# Therapeutic Immunization with HIV-1 Tat Reduces Immune Activation and Loss of Regulatory T-Cells and Improves Immune Function in Subjects on HAART

**DOI:** 10.1371/journal.pone.0013540

**Published:** 2010-11-11

**Authors:** Barbara Ensoli, Stefania Bellino, Antonella Tripiciano, Olimpia Longo, Vittorio Francavilla, Simone Marcotullio, Aurelio Cafaro, Orietta Picconi, Giovanni Paniccia, Arianna Scoglio, Angela Arancio, Cristina Ariola, Maria J. Ruiz Alvarez, Massimo Campagna, Donato Scaramuzzi, Cristina Iori, Roberto Esposito, Cristina Mussini, Florio Ghinelli, Laura Sighinolfi, Guido Palamara, Alessandra Latini, Gioacchino Angarano, Nicoletta Ladisa, Fabrizio Soscia, Vito S. Mercurio, Adriano Lazzarin, Giuseppe Tambussi, Raffaele Visintini, Francesco Mazzotta, Massimo Di Pietro, Massimo Galli, Stefano Rusconi, Giampiero Carosi, Carlo Torti, Giovanni Di Perri, Stefano Bonora, Fabrizio Ensoli, Enrico Garaci

**Affiliations:** 1 National AIDS Center, Istituto Superiore di Sanità, Rome, Italy; 2 Core Laboratory of Virology and Immunology, San Gallicano Hospital, “Istituti Fisioterapici Ospetalieri”, Rome, Italy; 3 Division of Infectious Diseases, University Policlinic of Modena, Modena, Italy; 4 Unit of Infectious Diseases, University Hospital of Ferrara, Ferrara, Italy; 5 Department of Infectious Dermatology, San Gallicano Hospital, Rome, Italy; 6 Division of Infectious Diseases, University of Bari, Policlinic Hospital, Bari, Italy; 7 Department of Infectious Diseases, S. Maria Goretti Hospital, Latina, Italy; 8 Division of Infectious Diseases, S. Raffaele Hospital, Milan, Italy; 9 Unit of Infectious Diseases, S.M. Annunziata Hospital, Florence, Italy; 10 Institute of Tropical and Infectious Diseases, University of Milan L. Sacco Hospital, Milan, Italy; 11 Division of Tropical and Infectious Diseases, Spedali Civili, Brescia, Italy; 12 Clinic of Infectious Diseases, Amedeo di Savoia Hospital, Turin, Italy; 13 Istituto Superiore di Sanità, Rome, Italy; National Institute of Allergy and Infectious Diseases, United States of America

## Abstract

Although HAART suppresses HIV replication, it is often unable to restore immune homeostasis. Consequently, non-AIDS-defining diseases are increasingly seen in treated individuals. This is attributed to persistent virus expression in reservoirs and to cell activation. Of note, in CD4^+^ T cells and monocyte-macrophages of virologically-suppressed individuals, there is continued expression of multi-spliced transcripts encoding HIV regulatory proteins. Among them, Tat is essential for virus gene expression and replication, either in primary infection or for virus reactivation during HAART, when Tat is expressed, released extracellularly and exerts, on both the virus and the immune system, effects that contribute to disease maintenance. Here we report results of an *ad hoc* exploratory interim analysis (up to 48 weeks) on 87 virologically-suppressed HAART-treated individuals enrolled in a phase II randomized open-label multicentric clinical trial of therapeutic immunization with Tat (ISS T-002). Eighty-eight virologically-suppressed HAART-treated individuals, enrolled in a parallel prospective observational study at the same sites (ISS OBS T-002), served for intergroup comparison. Immunization with Tat was safe, induced durable immune responses, and modified the pattern of CD4^+^ and CD8^+^ cellular activation (CD38 and HLA-DR) together with reduction of biochemical activation markers and persistent increases of regulatory T cells. This was accompanied by a progressive increment of CD4^+^ T cells and B cells with reduction of CD8^+^ T cells and NK cells, which were independent from the type of antiretroviral regimen. Increase in central and effector memory and reduction in terminally-differentiated effector memory CD4^+^ and CD8^+^ T cells were accompanied by increases of CD4^+^ and CD8^+^ T cell responses against Env and recall antigens. Of note, more immune-compromised individuals experienced greater therapeutic effects. In contrast, these changes were opposite, absent or partial in the OBS population. These findings support the use of Tat immunization to intensify HAART efficacy and to restore immune homeostasis.

**Trial registration:**

ClinicalTrials.gov NCT00751595

## Introduction

The use of antiretroviral drugs has changed the quality and expectancy of life of HIV-infected individuals [1]. However, in spite of viral-suppressing drug intervention, immune activation and loss of regulatory T-cells (T-reg), of CD4^+^ T cells, B cells, central memory CD4^+^ and CD8^+^ T cells and of immune functions are only partially reverted by HAART [1]–[8]. These dysfunctions are associated with an increased risk of non-AIDS-defining illnesses, including atherosclerosis, liver and kidney diseases, tumors and accelerated aging, that are now seen in HIV-treated disease [3].

To block these effects, novel non virus-targeting interventions, such as CCR5 antagonists, are being explored in association with conventional drugs [9], [10]. However, this approach appears to be only partially effective, suggesting that pathogenetic factors that maintain HIV disease should be targeted for restoring immune functions.

In this respect, residual virus replication is detected in most patients receiving HAART, likely originating from viral reservoirs, including latently infected CD4^+^ T cells, monocyte-macrophages, dendritic cells, NK cells, hematopoietic stem cells, mast cells and several cell types in the central nervous system [11]–[21]. This finding implies that viral gene products are still produced even under a “successful” therapy. Indeed, multi-spliced transcripts encoding HIV regulatory proteins are persistently expressed in viral reservoirs by unintegrated proviral DNA [22], [23], and are detected in resting CD4^+^ T cells, monocytes, and hematopoietic stem cells of HAART-treated individuals in the absence of detectable viremia [12],[13],[18],[22],[24]–[28]. Thus, HIV regulatory proteins are produced in latently infected cells [29], and can contribute to the persistent immune activation, immune system dysfunction, and disease observed in many HAART recipients [2], [4], [5], [17], [23], [30]–[32].

In particular, production of the Tat protein in virologically-suppressed individuals is confirmed by evidence of anti-Tat antibody (Ab) seroconversion and increases of Tat-specific T cell responses in HAART-treated patients (B. Ensoli et al., unpublished data).

Tat is the transactivator of HIV gene expression, which is essential for viral replication [33]–[35] and, therefore, for establishment of infection or virus reactivation [36]–[39]. Upon virus entry into cells, Tat is expressed by proviral DNA prior to virus integration [23], and it is released extracellularly early during acute infection or virus reactivation [37], [38], [40]–[42] by a leaderless secretory pathway similar to that used by bFGF and IL-Iβ to exit cells [40], [42], [43]. Upon release Tat binds heparan sulphate proteoglycans of the extracellular-matrix and is detected in tissues of infected individuals [40], [44]. Extracellular Tat exerts activities on both viral infection and immune activation that are key in acquisition of infection, as well as for virus reactivation and for HIV disease maintenance in HAART treated individuals [23], [31], [32], [38], [40], [42]–[51].

By targeting cells expressing RGD-binding integrin receptors such as dendritic cells, macrophages and activated endothelial cells via its RGD-binding site, extracellular Tat enters them very efficiently [44], [47], [52]. In these cells, Tat activates the proteasome leading to increased antigen processing and presentation thus contributing to Th-1 cell activation [48], [53], [54]. At the same time, via induction of TNFα, Tat induces the maturation of dendritic cells toward a Th-1 phenotype, again increasing T cell responses [31], [47], [52]. Tat also activates expression of cytokines with key immunomodulatory effects and/or capable of activating HIV gene expression [31], [45], [55]–[60]. Extracellular Tat also induces HIV co-receptor expression [61], [62] and can activate virus replication, rescue defective provirus, and facilitate virus transmission to neighbour cells [40], [43], [50]. Of note, the Tat protein is detected in highly purified virions [63], further supporting its key role in virus transmission and establishment of infection.

Thus, Tat plays key roles any time the virus needs to establish or to reactivate infection, i.e. at the acquisition of infection or under HAART-mediated viral suppression, both of which are accompanied by the presence of unintegrated proviral DNA expressing regulatory gene products and RGD-containing Tat protein isoforms [64]–[66].

Consistently with the roles of Tat in HIV pathogenesis, the presence of anti-Tat immune responses correlates with low or no progression to AIDS. In fact, when present, cellular and Ab anti-Tat responses exert protective roles to control virus replication and to delay disease progression, both in humans and monkeys [67]–[72]. Recently, a retrospective analysis on 112 monkeys with 67 vaccinees and 45 controls indicated that vaccination with Tat has statistically significant protective effects against acquisition of infection, and, in viremic monkeys, reduces significantly set-point viral load and CD4^+^ T cell decline [73]. Not surprisingly, anti-Tat Ab are produced by a small fraction (20%) of HIV-infected individuals in the asymptomatic phase and are lost during progression [69], [71]. In contrast, high Ab titers are produced against all viral products at all infection stages [74].

With these observations on Tat in mind, and after successful completion of preclinical [75]–[77] as well as preventative and therapeutic phase I studies [78]–[81] (http://www.hiv1tat-vaccines.info/), a phase II multicentric open-label clinical trial of immunization with the active Tat protein (ISS T-002, ClinicalTrials.gov NCT00751595) was initiated in anti-Tat Ab negative, HAART-treated and virologically-suppressed individuals. The primary endpoint of the trial was immunogenicity and the secondary endpoint was safety evaluation. In addition, the effect of Tat immunization on the immune activation and dysfunction seen in treated HIV disease was explored as second-line testing.

A parallel and prospective observational study (ISS OBS T-002, ClinicalTrials.gov NCT01024556) conducted on HAART-treated and virologically-suppressed individuals by the same clinical and laboratory platforms of the trial, and stratified to match the trial inclusions criteria served for intergroup comparison (data are shown in Supplementary material).

The open-label design of the phase II trial allowed us to follow up in a “real time” fashion all incoming data and to fine tune-up the second-line testing focused at assessing biomarkers of HAART efficacy. This exploratory testing was then included also in the observational study (OBS) to verify prospectively the presence or not of the same modifications found in the immunized population.

Due to the encouraging results, an *ad hoc* exploratory interim analysis was conducted on 87 subjects which completed the treatment phase. The results indicated that therapeutic immunization with Tat is safe, immunogenic and reverts biomarkers of HIV disease that persist under virologically-suppressing antiretroviral treatment [1]–[5]. In addition, greater therapeutic effects were seen in more immune compromised individuals. After reviewing of these data by the Investigators, the Data Safety Monitoring Board (DSMB), the International Advisory Board (IAB), and the Community Advisory Board (CAB), and in view of the urgency to improve HIV treatment, an amendment was proposed to and approved by the Ethical Committees. This amendment extends the trial to include also more immune compromised individuals and to expand the total sample size from 128 to 160 volunteers. Thus, the trial is still continuing and it is now recruiting according to the broader inclusion criteria (ClinicalTrials.gov NCT00751595; Supporting information).

The results of the pre-amendment trial population (87 subjects) are reported here and they proof the role of Tat in HIV pathogenesis and disease maintenance under HAART, providing encouragement for combining Tat immunization with conventional virus-targeting drugs for an improved treatment of HIV disease.

## Methods

The protocol for the ISS T-002 clinical trial and supporting CONSORT checklist are available as supporting information; see Checklist S1 and Protocol S1. The protocol for the ISS OBS T-002 observational study is available as Supporting information; see Protocol S2.

### ISS T-002 trial design, conduction and duration

The study “*A phase II randomized, open label, immunogenicity and safety trial of the vaccine based on the recombinant biologically active HIV-1 Tat protein in anti-Tat antibody negative HIV-1 infected HAART treated adult subjects*”, ISS T-002 (EudraCT No. 2007−007200−16; ClinicalTrials.gov NCT00751595) is a randomized, open-label, phase II multicentric clinical trial directed at evaluating the immunogenicity (primary end-point) and the safety (secondary end-point) of the HIV-1 Tat protein in HIV-1 infected adult subjects, anti-Tat Ab negative, of either gender, 18-55 years-old, HAART-treated with chronic suppressed infection and levels of plasma viremia <50 copies/mL in the last 6 months prior to screening and without a history of virologic rebound, with CD4^+^ T cell counts ≥400 cells/µL and with pre-HAART CD4 nadir >250 cells/µL.

The study was approved by the national regulatory body and by the Ethics Committees of each clinical center. All subjects signed the written informed consent prior to enrollment. The trial was conducted in 10 clinical sites in Italy (Policlinico of Modena, Modena; *Arcispedale S. Anna*, Ferrara; Istituti Fiosterapici Ospitalieri San Gallicano, Rome; Policlinico of Bari, Bari; *Ospedale S.M. Goretti* Latina; Fondazione S. Raffaele, Milan; *Ospedale S. Maria Annunziata* Florence; Ospedale Luigi Sacco, Milan; Spedali Civili, Brescia; *Ospedale A. di Savoia*, Turin). Subjects were randomized, with a competitive enrolment, to one of the four immunization regimens represented by 3 or 5 intradermal administrations of Tat at two doses (7.5 µg or 30 µg). The study includes 3-weeks screening period, 8-weeks or 16-weeks treatment period, 40-weeks or 32-weeks of follow-up for the 3 or 5 immunizations, respectively, and foresees an extended follow-up of 3 years from the first immunization (Supporting information).

Blood samples from clinical sites were shipped by a certified courier to the Core Laboratory of Immunology and Virology (Ospedale S. Gallicano IFO, Rome, Italy) where all the immunological and virological testing was performed according to the Standard Operating Procedures (SOP) developed within the AIDS Vaccine Integrated Program (AVIP) [82], funded within the FP6 program of the European Community, and implemented in the corresponding phase I preventative (Clinicaltrials.gov Identifier NCT00529698) and therapeutic (Clinicaltrials.gov Identifier NCT00505401) clinical trials [78]–[81].

All safety assessments were performed at the clinical sites according to the study schedule (see Protocol S1 in Supporting information). The study was monitored and quality assured by an accredited Contract Research Organization (CRO). The study enrolment started on July 2008 and is still open since an amendment has been approved by the DSMB and Ethical Committees to include individuals with a more advanced immune compromission and to expand the sample size from 128 to 160 volunteers (www.clinicaltrials.gov) (Supporting information).

The data shown here refer to the protocol prior to the amendment (Protocol S1) and include all the pre-amendment enrolled volunteers (87).

### Study Medication

The study medication is the biologically active recombinant Tat protein administered intradermally in two doses (7.5 µg and 30 µg) according to two regimens (3 or 5 immunizations) at week 0, 4, 8 or 0, 4, 8, 12, 16, respectively. Before administration, the study medication (Tat 7.5 µg/0.5 mL or Tat 30 µg/0.5 mL) was thawed, diluted with 1.5 mL of sterile water, swirled gently and then administered by two intradermal injections into the right and left deltoid regions of the upper arms.

### Study Outcomes

The primary pre-specified outcome of the study (immunogenicity) was measured by the induction, magnitude and persistence of the humoral immune responses to Tat, and by comparing the immunogenicity of the 3 or 5 immunization schedule of the two different vaccine doses (7.5 µg and 30 µg) at each scheduled time point (see Protocol S1 in Supporting information). “Responders” were defined as those subjects with at least a positive anti-Tat Ab response at any given time point after the first immunization. Cellular immune responses to Tat were a co-primary endpoint.

The anti-Tat humoral immune response was evaluated by determination and titration of IgM, IgG and IgA anti-Tat Ab in sera, while the anti-Tat cellular immune response was evaluated by the assessment of CD4^+^ and CD8^+^ lymphoproliferative responses and *in vitro* IFN-γ, IL-4 and IL-2 production in response to Tat, as detailed below.

The secondary pre-specified outcome of the study (safety) was assessed by the collection of all adverse events occurred during the study, including any significant change in haematological (including coagulation assessment), biochemical (with liver and kidney functional parameters) and immunological parameters (including CD4^+^, CD8^+^, CD3^+^ T cells, NK, B cells and monocytes). All the AEs were reported according to the Medical Dictionary for Regulatory Activities (MedDRA) and classified on the basis of the drug relationship as well as by the grade of severity.

All the safety data were periodically (every 3 months) evaluated by the DSMB and Annual Safety reports were submitted to the Regulatory Bodies at scheduled times, as by regulatory guidelines (DLgs 211/2003).

Finally, a second-line exploratory testing was performed to characterize in-depth biochemical and immunological biomarkers of disease progression used to assess HAART efficacy, including determination of cellular and biochemical markers of immune activation, regulatory T cells, cell viability, CD4^+^ T cells, CD8^+^ T cells, B cells and NK cells, central and effector memory CD4^+^ and CD8^+^ T cells as well as cellular responses to HIV Env and to recall antigens, as detailed below.

All data have been reviewed by the Investigators, the DSMB, the IAB and the CAB.

### Sample size

The primary objective of the trial was to assess the immunogenicity of Tat immunization. In particular, “responders” were defined as subjects with at least a positive anti-Tat Ab response at any given time point after the first immunization, as indicated in the Protocol S1 (Supporting information).

To observe a proportion of at least 80% of subjects with Ab responses to vaccination, taking into account a maximum margin of error of 7% and a confidence level of 95%, 112 valuable subjects were found to be required, 28 for each treatment group. In this hypothesis the width of the confidence interval within each of the 4 randomization arms is 15%.

With this sample size a difference of at least 35% of the responders between the two Tat doses with each immunization schedule (3 or 5 inocula, respectively) is detected as statistically significant.

Considering a drop-out rate no greater than 10%, the calculated sample size is 128 subjects, randomized in 4 treatment groups by 32 subjects each.

### Randomization

Since the study design foresees different immunization schedules, subjects were randomized into 4 treatment groups to ensure unbiased patient allocation.

The Randomization list was generated by the CRO using a block size of 4, according to a randomization scheme of 1∶1∶1∶1 (RND PLUS 2.10 software).

The randomization assignment was carried out by the CRO via web. Each clinical site received a block of 4 numbered treatments (according to the randomization list). A code was assigned at screening at each subject. The code is constituted by the clinical trial code (T2) followed by the clinical site number (01 to 10) and by the progressive patient number within each site. The code was assigned to each volunteer at screening, irrespectively of enrolment. Once eligibility was established, the clinical staff contacted the CRO via web to randomize the subject. The drug kit number to be assigned to the subject was provided via web within the block's numbers that the clinical site had received. The assigned drug kit number was recorded on the e-CRF.

### ISS OBS T-002 observational study enrollment and conduction

The ISS OBS T-002 (ClinicalTrials.gov NCT01024556) is a 5-years prospective observational study, which started before the therapeutic trial and is conducted in parallel at the same clinical centers, and follows the same procedures of the therapeutic trial ISS T-002 (sample collection and certified transportation, centralized immune and virologic testing, certified CRO management). The primary objective of this study is to prospectively evaluating the clinical, immunological and virological parameters to determine the impact of naturally occurring (i.e., not induced by vaccination) anti-Tat immunity in HIV-infected individuals, of either gender, ≥18 years-old, with HAART-suppressed HIV replication (plasma viremia <50 copies/mL for 6 months prior to screening) without a history of virologic rebound and with known CD4^+^ T cell number and nadir. In addition, the study had the secondary objective of harmonizing the multicentric clinical platform on common SOPs and procedures in order to both prepare cohorts for trials and to optimize all the operations required for the therapeutic trial conduction. The study is still recruiting. To date 127 patients have been enrolled and are being followed-up, 25 of them are anti-Tat Ab positive and 91 are anti-Tat Ab negative (Fig. S1). Eighty-eight anti-Tat Ab negative (out of the 91) are evaluable subjects (Total OBS Subjects), and 32 out of them meet all the immunological (CD4^+^ T cell counts ≥400 cells/μL, pre-HAART CD4 nadir >250 cells/μL) and virological criteria for eligibility in the ISS T-002 clinical trial, representing therefore the appropriate Reference Group for a comparative assessment of the results with the trial subjects (Fig. S1, Table S1). For clarity, data from the observational study are reported in the Supplementary material section and the protocol is available in Supporting information (Protocol S2).

### Anti-Tat antibodies

Ab were assessed as described [76], [79], [81], [83]. Titers are expressed as the reciprocal of sample dilution. Ab titers equal or higher than 25 for IgM and IgA, or 100 for IgG were considered as positive.

### IFN-γ, IL-2 and IL-4 Elispot

Elispot was performed as previously described [79]–[81] using commercial kits (EL285, EL202, EL204, R&D Systems), with 4 pools of overlapping 15mer-Tat peptides (5 µg/mL each) (UFP Service, Ferrara, Italy), 2 pools of Env peptides (5 µg/mL each) (Neosystem), Candida (5 µg/mL) (Nanogen Advanced Diagnostics), or a combination of Cytomegalovirus, Epstein-Barr and influenza virus (CEF) peptide pool (2 µg/mL each) (Anaspec, 01036–05). PHA, (2 µg/mL) or medium were the positive and the negative controls, respectively. Tests were considered valid when Spot Forming Cells (SFC)/well were ≥100 in positive controls. IFN-γ Elispot was considered positive when SFC/10^6^ cells were ≥30, and fold-increase over control was ≥3. The IL-2 and IL-4 Elispot were considered positive when fold-increase was ≥3.

### T cell proliferation

Responses to Tat (1–5 µg/mL) or Tat peptides (2 µg/mL), Env (5 µg/mL) (Fitzgerald), Candida (5 µg/mL), or CEF peptide pool (2 µg/mL each) were assessed by CellTrace CFSE Cell Proliferation kit (Molecular probesTM, Invitrogen), elaborated by ModFit software (Verity Software House, INC.), and expressed as Proliferation Index (PI). Fold-increase (FI), calculated as ratio of PI with antigens versus PI of controls, was considered positive when was ≥2.

### Peripheral Blood Mononuclear Cell (PBMC) viability and lymphocyte subsets

Cell viability was determined by Trypan Blue Dye Exclusion using the Vi-CELLTM XR Counter (Beckman Coulter) [84], [85]. Phenotyping was performed with BD Multitest 6-color TBNK reagent with BD Truecount tubes, (BD Biosciences). Samples were processed by FACSCanto flow cytometer (BD Biosciences) and data analyzed by FACSCanto clinical software. CD4^+^ T cell counts were also performed in parallel at each clinical center, and results were highly consistent with those generated by the Core lab.

### Immune activation markers and T-reg

Whole blood was stained with anti-CD8 FITC/CD38 PE/CD3 PerCP/HLA-DR APC (MultiTEST™ BD Biosciences) plus anti-CD4 APC-Cy7 Ab (BD Pharmingen). Collective quadrant gates, based on HLA-DR and CD38 expression on CD4^+^ or CD8^+^ T cells, were established.

Neopterin, β2-microglobulin and total immunoglobulins (Ig) were determined as described [86].

For CD25^+^ and T-reg, PBMCs were stained with anti-human APC-Cy7-labeled CD4, APC-labeled CD25 (BD Biosciences) and PE-labeled FOXP3 Ab (Bioscience, USA). Gating was performed on CD4^+^ and CD4^-^ for CD25 expression on total T cells, and on CD4^+^ T cells for CD25/FOXP3 doubly-positive cells, and then on CD4/CD25 for FOXP3^+^ lymphocytes.

### Naïve, central and effector memory CD4^+^ or CD8^+^ T cells

PBMC were stained with anti-human CD3 (PerCP), CD4 (APC-Cy7), CD8 (APC), CD45RA (FITC), CD62L (PE) Ab (MultiTEST™ BD Biosciences), analyzed by FACSCanto flow cytometer (BD Biosciences) with the FlowJo (Tree Star, Ashland, OR) software.

T cell subsets were identified by hierarchical gating (morphological, on CD3^+^, and then on CD4^+^ or CD8^+^ T cells). Collective quadrant gates based on CD45RA and CD62L expression on CD3^+^/CD4^+^ or CD3^+^/CD8^+^ T cells identified naïve (CD45RA+/CD62L+), central memory (CD45RA-/CD62L+, Tcm), effector memory (CD45RA-/CD62L-, Temro), and terminally-differentiated effector memory (CD45RA+/CD62L-, Temra) subsets.

### HIV-1 viral load

HIV-l RNA was determined with COBAS AmpliPrep/COBAS TaqMan HIV-1 Test, version 2.0, (Roche Diagnostics) [84], [87].

### Statistical methods

The percentage of “responders” was estimated both for total vaccinees and for each randomization group with a 95% confidence interval.

Cochran-Armitage Trend test was used to compare frequencies of humoral responses. McNemar's test was used to compare pre-post immunization frequencies of cellular responses within treatment groups. Wilcoxon signed-rank test was applied to evaluate increase of cellular responses intensity. Student's t-test for paired data was used to assess the mean changes from baseline of activation markers, lymphocytic phenotypes and cell viability, after controlling normality assumption of variables distribution (Saphiro-wilk test). Multivariate regression model for repeated measures, stratified by Tat dose, was applied to CD38^+^/CD8^+^ T cells (%), including anti-Tat Ab titers, cell viability and CD25^+^ FOXP3^+^ expression on CD4^+^ lymphocytes as explicative factors. The same model was applied on CD38^+^/CD8^+^ T cells (%) to all immunized subjects, to assess the potential relationships with the anti-Tat Ab titers (IgM, IgG, IgA), CD8^+^ central memory (%) and anti-Tat induced cytokines (IFN-γ, IL-2 and IL-4).

Since phase II studies are usually descriptive and are not designed to quantify treatment effects, the statistical analyses were not focused to determine differences in term of efficacy among three or more treatment groups and no adjustments were used for multiple comparisons. Therefore, exploratory analyses were applied to many variables to investigate the potential effect of the Tat immunization on the immunological status of vaccinees.

All statistical tests were carried out at a two-sided 5% significance level. Analyses and data processing were performed using SAS® software (SAS Institute, Cary, NC, USA).

## Results

### Study participants

**Figure 1:**
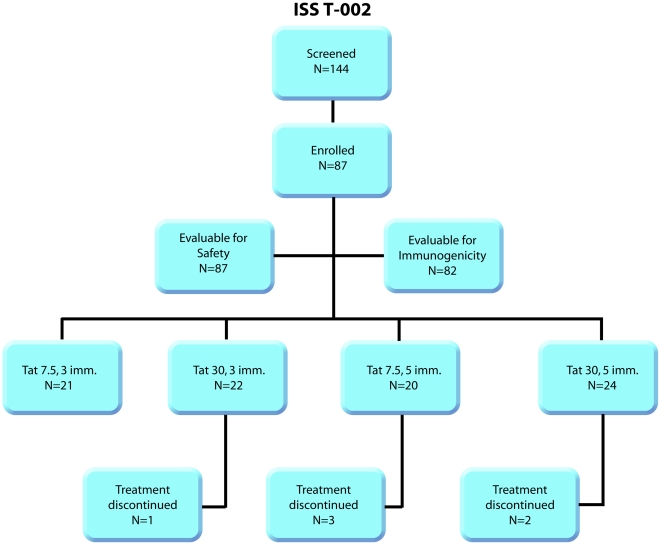
Flow diagram of the study participants. One hundred and forty-four HAART-treated patients were screened for enrollment. Of them, 87 met the inclusion criteria and were randomized on schedule and dose of immunization. This represents all the study population prior to the protocol amendment. All recruited individuals were included in the safety analysis (n = 87). Six subjects discontinued the immunization schedule. Of them, 5 were evaluated only for safety, since received at least one immunization, and 1 was evaluated also for immunogenicity since received 3 immunizations out of 5 (as indicated in the Protocol S1). A total of 82 individuals completed the 20-weeks period of the study and 68 have completed the 48-weeks period after the first immunizations.

Study participants were randomized by immunization schedule (3 or 5 times monthly) and Tat doses (7.5 or 30 µg) (Fig. 1 and Table 1 and Methods). The results refer to the full pre-amendment trial population (n = 87). Sixty-eight trial participants had also a 48-weeks of follow-up.

**Table 1:** Baseline characteristics of the study participants.

	ISS T-002 (*n* = 87)^a^
**Age (yr)**	
Mean ± s.d.^b^	41±7
Range	26–54
**Sex (%)**	
Male	78.2
Female	21.8
**CD4^+^ nadir (cells/µL)**	
Mean ± s.d.	352±114
Range	252–846
**CD4^+^ (cells/µL)**	
Mean ± s.d.	693±210
Range	425–1490
**CD4^+^ (%)**	
Mean ± s.d.	34±7
Range	19–53
**HIV RNA (copies/mL)**	<50
**Time since diagnosis of HIV (yr)**	
Mean ± s.d.	9±6
Range	1–23
**Time since HAART initiation (yr)^c^**	
Mean ± s.d.	6±4
Range	0–19
**Current HAART regimen (%)**	
Includes PI	25.3
Includes NNRTI	65.5
Includes NRTI	9.2

a Number of evaluable individuals; ^b^Standard deviation; ^c^Based on 76 individuals.

All study participants enrolled prior to the protocol amendment are shown.

Eighty-eight HAART-treated anti-Tat Ab negative and virologically-suppressed individuals (for at last 6 months and without a history of virological rebound) with a known number of CD4^+^ T cells and CD4 T cell nadir, were enrolled in the prospective observational study (ISS OBS T-002, ClinicalTrials.gov NCT00751595). The results of this study served for intergroup comparison. In particular, 32 of these subjects fully matched the baseline characteristics of the immunized patients and were, therefore, considered as the Reference Group (Fig. S1, Table S1 and Methods). However, since no major differences were seen between the Reference Group and the full OBS population (Total OBS Subjects), results from both these groups are reported (Supplementary material).

### Safety Data of therapeutic immunization with the biologically active Tat protein

Safety was assessed in all trial (n = 87) volunteers by monitoring local and systemic adverse events (AEs) as well as hematological, biochemical and immunological laboratory parameters as performed previously in phase I trials [79]–[81]. Since in immunized subjects no differences in the incidence of adverse events were detected between the number of inocula (3 or 5) with the same Tat dose, the results were stratified by Tat dosages.

No relevant AEs occurred during the study, and most of them were expected both in frequency and type for HIV-infected subjects (Table 2). In particular 51/87 subjects (59%) experienced AEs during the study. Out of them, 26/87 (30%) presented a *certain, probable* or *possible* relationship to the study treatment and were not related to the Tat dosage. These events were mostly local, related to the injection site and mild in severity (Table 2).

**Table 2:** Adverse Events (AEs) defined as *certainly, probably* or *possibly* related to the study medication.

Incidence and Severity of Treatment-Related AEs by Preferred Term		Tat 7.5 µg (N = 41)		Severity^c^			Tat 30 µg (N = 46)		Severity^c^		
System Organ Class (SOC)	MedDRA Preferred Term	n^a^	n/N^b^ (%)	1	2	3	n^a^	n/N^b^ (%)	1	2	3
**Blood and lymphatic system disorders**	Lymphocytic infiltration	1	2.4	1	0	0	0	0.0	0	0	0
**Cardiac disorders**	Chest Discomfort	1	2.4	0	0	1	0	0.0	0	0	0
	Bradycardia	1	2.4	0	1	0	0	0.0	0	0	0
**Eye disorders**	Photophobia	1	2.4	1	0	0	0	0.0	0	0	0
	Visual impairment	1	2.4	1	0	0	0	0.0	0	0	0
	Conjunctivitis	1	2.4	1	0	0	0	0.0	0	0	0
**Gastrointestinal disorders**	Nausea	0	0.0	0	0	0	1	2.2	1	0	0
**General disorders**	Asthenia	1	2.4	1	0	0	1	2.2	1	0	0
	Fatigue	1	2.4	1	0	0	0	0.0	0	0	0
	Hyperidrosis	1	2.4	1	0	0	0	0.0	0	0	0
	Injection site discomfort	0	0.0	0	0	0	1	2.2	1	0	0
	Injection site erythema	0	0.0	0	0	0	1	2.2	1	0	0
	Injection Site Irritation	4	9.8	4	0	0	2	4.3	2	0	0
	Injection Site Pain	10	24.4	10	0	0	13	28.3	13	0	0
	Malaise	1	2.4	1	0	0	0	0.0	0	0	0
	Pyrexia	0	0.0	0	0	0	4	8.7	4	0	0
**Musculoskeletal and connective tissue disorders**	Muscular weakness	0	0.0	0	0	0	1	2.2	1	0	0
**Nervous System Disorders**	Agitation	1	2.4	1	0	0	0	0.0	0	0	0
	Aphasia	1	2.4	0	0	1	0	0.0	0	0	0
	Disturbance in attention	1	2.4	1	0	0	0	0.0	0	0	0
	Dysarthria	1	2.4	0	0	1	0	0.0	0	0	0
	Headache	3	7.3	3	0	0	1	2.2	1	0	0
	Paraesthesia oral	1	2.4	0	0	1	0	0.0	0	0	0
	Trigeminal neuralgia	1	2.4	0	1	0	0	0.0	0	0	0
**Skin and subcutaneous tissue disorders**	Erythema	0	0.0	0	0	0	1	2.2	1	0	0
	Pruritus generalised	1	2.4	1	0	0	0	0.0	0	0	0

a n = number of subjects reporting the events; ^b^ N = number of evaluable subjects (%); ^c^ Severity grade: 1 = mild, 2 = moderate, 3 = severe.

All the study participants prior to the protocol amendment (n = 87) have been evaluated for safety. The Medical Dictionary for Regulatory Activities (MedDRA) was used to classify the adverse events occurred during the study.

Seven serious adverse events (SAE) occurred after Tat immunization, but only one was indicated as *possibly* related to the study treatment (Table 3).

**Table 3:** *Related* or *unrelated* serious adverse events occurred during the ISS T-002 trial.

Volunteer	Randomization	Description	Relationship	Severity	Action taken	Outcome
	group		to treatment			
#1	30 µg, 5 imm.	Hepatitis A	Unrelated	Severe	Hospitalization	Resolved
					Treatment discontinued	
#2	7.5 µg, 5 imm.	Disarthria and	Possible	Severe	Hospitalization	Resolved
		Paresthesia			Treatment discontinued	
		of the tongue				
#3	7.5 µg, 3 imm.	Neurosyphilis	Unrelated	Mild	Hospitalization	Ongoing
#4	30 µg, 3 imm.	Uterin fibroma	Unrelated	Severe	Medication required	Resolved
					Treatment discontinued	
#5	30 µg, 5 imm.	Hodgkin's	Unrelated	Severe	Medication required	Ongoing
		lymphoma				
#6	7.5 µg, 3 imm.	Transaminase	Unrelated	Moderate	Hospitalization	Resolved
		increase				
#7	30 µg, 3 imm.	Right occipital	Unrelated	Severe	Medication required	Ongoing
		lesion				
		(ischemic event)				

All study participants prior to the protocol amendment (n = 87) have been evaluated.

Based on the first and second year Annual Safety Report and on the periodic meetings and reports, the DSMB of the ISS T-002 deliberated that the immunization with Tat is safe and well tolerated.

### Therapeutic immunization with Tat induces specific humoral and cellular immune responses in HAART-treated individuals

Responders were defined by the induction of anti-Tat Ab. Total responders were 79% (95%C.I. 70–88%), with a higher frequency at the Tat 30 µg dose (86%; 95%C.I. 76–96%) as compared to the Tat 7.5 µg dose (72%; 95%C.I. 58–86%). After stratification by the randomization groups, responders were 76% (95%C.I. 58–94%) at 7.5 µg, 3 inocula, and 67% (95%C.I. 45–88%) after 5 inocula, respectively. At the 30 µg Tat dose responders were 86% (95%C.I. 71–100%) after 3 inocula and 86% (95%C.I. 72–100%) after 5 inocula, respectively.

**Figure 2:**
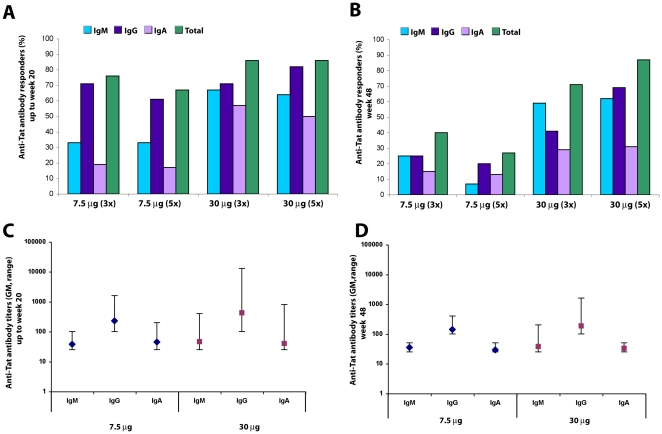
Anti-Tat humoral immune responses. (**A**) Percentage of subjects developing anti-Tat IgM (blue bar), IgG (purple bar), IgA (violet bar), or total anti-Tat Ab (green bar), stratified by Tat dose and treatment groups. Responder frequency up to week 20 among groups was analyzed by the Cochran-Armitage Trend test (p = 0.0117 and p = 0.0051 for IgM and IgA, respectively). Tat 7.5 µg, 3x, n = 21; Tat 7.5 µg, 5x, n = 18; Tat 30 µg, 3x, n = 21; Tat 30 µg, 5x, n = 22 subjects, respectively. (**B**) Percentage of subjects positive for anti-Tat Ab at week 48 (Tat 7.5 µg, 3x, n = 20; Tat 7.5 µg, 5x, n = 15; Tat 30 µg, 3x, n = 17; Tat 30 µg, 5x, n = 16 subjects, respectively). Responder frequency among groups was analyzed as described above (p = 0.0024 and p = 0.0048 for IgM and IgG, respectively, p = 0.0007 for total Ig). (**C**) Peak of anti-Tat IgM, IgG or IgA titers (geometric mean and range) up to 20 weeks after the first immunization from subjects positive for anti-Tat Ab (blue diamond Tat 7.5 µg and red square Tat 30 µg). Anti-Tat IgM: Tat 7.5 µg, n = 14; Tat 30 µg, n = 28. Anti-Tat IgG: Tat 7.5 µg, n = 26; Tat 30 µg, n = 33. Anti-Tat IgA: Tat 7.5 µg, n = 7; Tat 30 µg, n = 24 subjects, respectively. (**D**) Anti-Tat IgM, IgG or IgA titers at 48 weeks after the first immunization from subjects positive for anti-Tat Ab. Anti-Tat IgM: Tat 7.5 µg, n = 6; Tat 30 µg, n = 20. Anti-Tat IgG: Tat 7.5 µg, n = 8; Tat 30 µg, n = 18. Anti-Tat IgA: Tat 7.5 µg, n = 5; Tat 30 µg, n = 10 subjects, respectively.

The 30 µg dose was, therefore, more potent at inducing anti-Tat Ab and at maintaining long-term humoral responses with little or no differences between the 3 or 5 inoculation regimens (Fig. 2 A–B). Specifically, during the immunization phase (up to week 20) the 30 µg Tat dose was the most effective at inducing anti-Tat IgM and IgA subclasses (p = 0.0117 and p = 0.0051, respectively) (Fig. 2A). This Tat dose was also the most effective at inducing a durable Ab response (p = 0.0007), which was still present at approximately 1 year post-immunization (48 weeks), for both IgM and IgG subclasses (p = 0.0024 and p = 0.0048, respectively) (Fig. 2B). In contrast, peak Ab titers did not significantly differ between doses and only slightly decreased at 48 weeks (Fig. 2 C, D).

**Figure 3:**
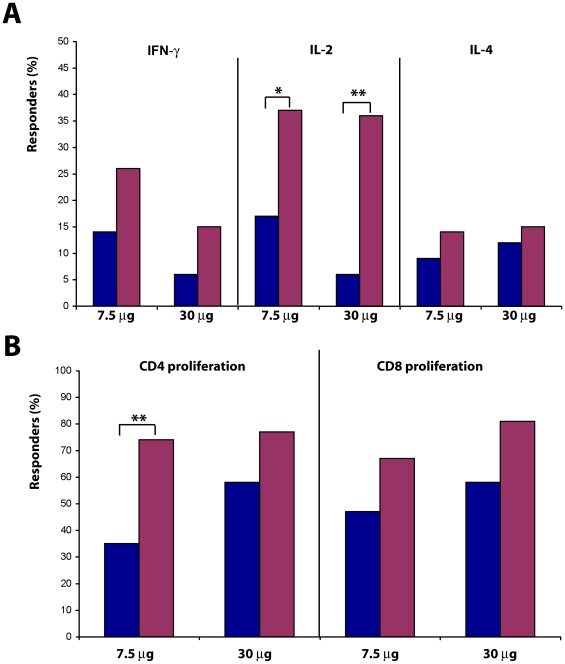
Anti-Tat cellular immune responses. (**A**) Percentage of subjects showing anti-Tat specific production of IFN-γ, IL-2 or IL-4, respectively, measured at baseline (blue bar) and up to week 48 after the first immunization (red bar) and stratified by Tat dose (Tat 7.5 µg, n = 35; Tat 30 µg, n = 33 subjects, respectively). (**B**) Percentage of subjects showing anti-Tat CD4^+^ or CD8^+^ lymphoproliferative responses measured at baseline and up to week 48 and stratified by Tat dose (Tat 7.5 µg, n = 31; Tat 30 µg, n = 31). The analysis was performed using the McNemar's test: *p<0.05, **p<0.01.

Both Tat doses induced specific cellular responses (Fig. 3 A–B and Table 4). A cumulative analysis of the T cell responses (up to 48 weeks after immunization) indicated an increase in the percentage of responders for production of IL-2 (p = 0.0348 and p = 0.0039 at Tat 7.5 or 30 µg, respectively) and, to a lesser extent, IFN-γ and IL-4, as well as of Tat-specific CD4^+^ (p = 0.0013 at Tat 7.5 µg) and CD8^+^ T cell proliferation. No relevant differences were observed between 3 or 5 immunizations.

**Table 4:** Tat-specific cellular immune responses in subjects with a positive response after immunization with Tat.

ISS T-002		Tat 7.5 µg^b^			Tat 30 µg^c^	
	*n*	Baseline	Up to week 48	*n*	Baseline	Up to week 48
**IFN-γ**						
Peak^a^ (SFC/10^6^ cells)	9	30 (10–48)	142 (92–208)**	5	14 (12–16)	50 (42–100)
**IL-2**						
Peak^a^ (SFC/10^6^ cells)	13	8 (2–30)	60 (30–104)*	12	9 (3–19)	29 (20–38)**
**IL-4**						
Peak^a^ (SFC/10^6^ cells)	5	6 (0–10)	40 (36–46)	5	2 (0–2)	44 (24–58)
**CD4 Proliferation**						
Peak^a^ (fold increase)	23	1.9 (1.4–2.9)	2.5 (2.1–4.5)**	24	2.2 (1.6–3.0)	3.8 (2.3–5.5)**
**CD8 Proliferation**						
Peak^a^ (fold increase)	20	2.1 (1.4–3.4)	4.0 (2.6–8.5)**	25	2.7 (1.6–6.5)	5.4 (2.5–8.2)**

The median intensity, with interquartile range of peak of responses, is shown for subjects with at least a positive cellular response at any given time point after the first immunization and up to week 48. Pre-post vaccination median change was evaluated by the Wilcoxon signed-rank test. IFN-γ, IL-2, IL-4 production by PBMC and CD4^+^ or CD8^+^ lymphoproliferative responses were measured at baseline and up to week 48 after the first immunization. Results are stratified by Tat doses, (7.5 and 30 µg). *n* indicates the number of responders.

a Median (interquartile range) of peak of responses, weeks 8, 12, 20, 48.

b Total subject tested for cytokines: 35; for proliferation: 31.

c Total subjects tested for proliferation: 33; for proliferation: 31.

*P<0.05, ** *P*<0.01.

Further, increased peak values were detected for both cytokines and CD4 ^+^ and CD8^+^ T cell proliferation in response to Tat, which reached significance with Tat at 7.5 µg for IFN-γ (p = 0.0078), and IL-2 (p = 0.0327), and at Tat 30 µg for IL-2 (p = 0.0005), and with both Tat doses for CD4^+^ and CD8^+^ T cell proliferation (p<0.001) (Table 4).

### Immunization with Tat downregulates phenotypic and biochemical markers of immune activation and increases regulatory T cells

To further investigate the effect of immunization with Tat, key biomarkers of AIDS pathogenesis and progression used to evaluate HAART efficacy were determined as second-line exploratory testing. These included phenotypic (CD38, HLA-DR) and biochemical (serum β2-microglobulin, neopterin and total immunoglobulins) immune activation markers. In addition, T-reg lymphocytes, that are markedly reduced in HIV-infected individuals even under HAART, were monitored since these cells are key for controlling immune activation and both the generation and termination of adaptive immune responses. Baseline values of these parameters were determined at study entry (Table 5). Since no differences were detected in immunized subjects between the number of inocula (3 or 5) with the same Tat dose, results were stratified by Tat dosage.

**Table 5:** Immune activation markers and T-reg at baseline in study participants.

ISS T-002	*n*	Mean ± s.e.
CD38^+^HLA-DR^−^ on CD8^+^ T cells (%)	38	31.5±2.0
HLA-DR^+^CD38^−^ on CD8^+^ T cells (%)	38	5.5±0.8
CD38^+^HLA-DR^+^ on CD8^+^ T cells (%)	38	6.0±0.7
CD38^+^ HLA-DR^−^ on CD4^+^ T cells (%)	37	49.7±2.2
HLA-DR^+^CD38^−^ on CD4^+^ T cells (%)	37	4.5±0.5
CD38^+^HLA-DR^+^ on CD4^+^T cells (%)	37	3.8±0.5
β2-microglobulin (mg/L)	77	1.7±0.1
Neopterin (nmol/L)	77	7.5±1.0
Total IgM (mg/dL)	77	77±5
Total IgG (mg/dL)	77	1025±29
Total IgA (mg/dL)	77	196±11
CD25^+^ on CD4^+^ T cells (%)	66	8.6±0.3
FOXP3^+^ on CD4^+^CD25^+^ T cells (%)	60	31.2±1.4
CD25^+^FOXP3^+^ on CD4^+^ T cells (%)	60	2.7±0.2
CD25^+^FOXP3^+^ on CD4^+^ T cells (cells/µL)	59	19.4±11.7

Mean values (± standard error) of phenotypic and biochemical immune activation markers and T-regs of the study participants at baseline. *n* indicates the number of individuals tested for each parameter.

### CD38 and HLA-DR expression

**Figure 4:**
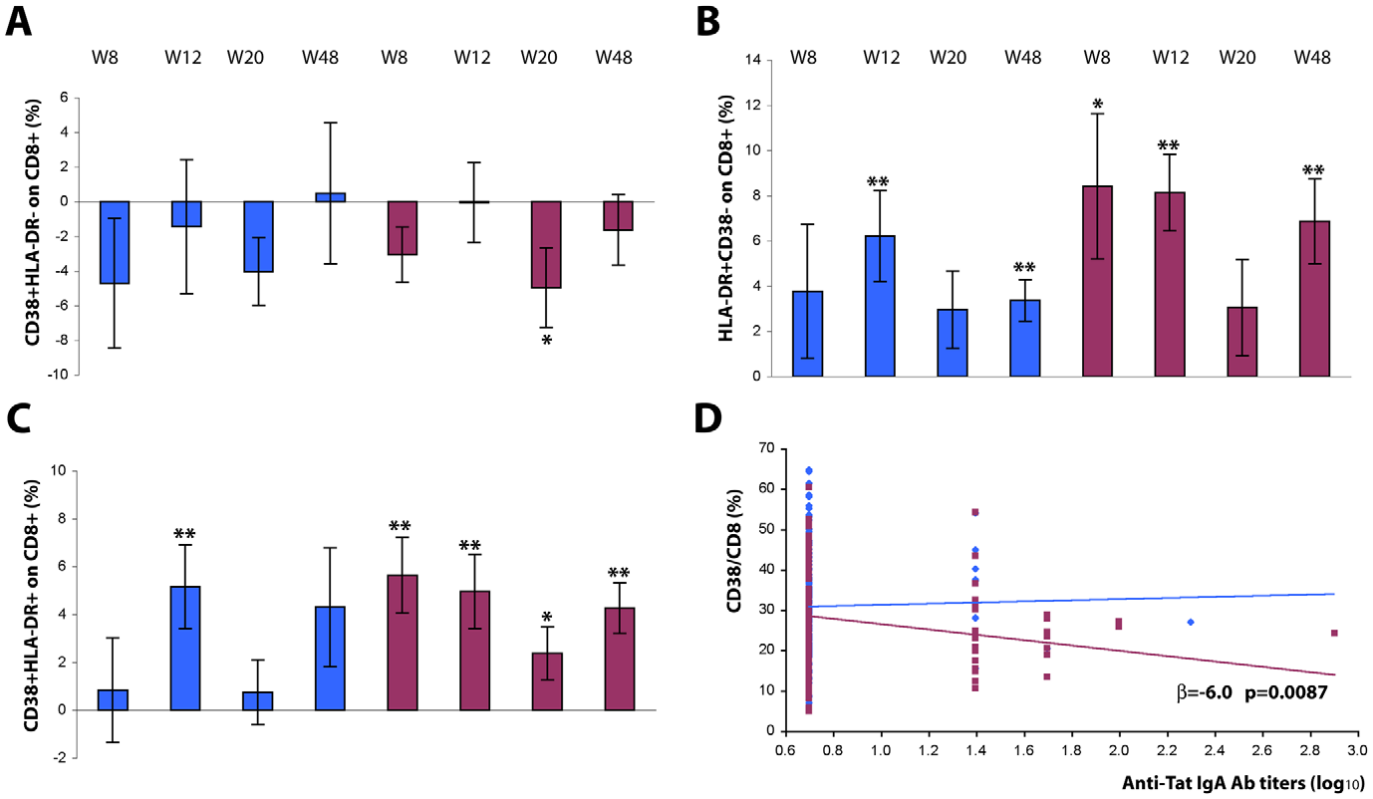
Expression of activation markers on CD8^+^ T cells after Tat immunization. Changes from baseline of CD8^+^ T cells (gating on CD8^+^ T cells) expressing (**A**) CD38, (**B**) HLA-DR, or (**C**) both CD38 and HLA-DR. Results are shown according to Tat dose and time after the first immunization. Data are presented as the mean % changes (± standard error) at week 8, 12, 20 and 48. Blue bars: Tat 7.5 µg, n = 17 up to week 20 and n = 12 at week 48; red bars: Tat 30 µg, n = 21 up to week 20, n = 16 at week 48, respectively. The t-Test for paired data was used for the analyses: *p<0.05, **p<0.01. (**D**) Correlation between CD38^+^/CD8^+^ T cells (%) and anti-Tat IgA antibody titers (Multivariate regression model for repeated measures). Blue diamond: Tat 7.5 µg, n = 39; red square: Tat 30 µg, n = 43, respectively.

Downregulation of CD38 expression was observed on CD8^+^ T cells from subjects immunized with both Tat doses, and was more pronounced and persistent at Tat 30 µg, reaching statistical significance at week 20 (p = 0.0436) to decline thereafter (week 48) (Fig. 4 A and Table 5). At the same time, a significant increase of HLA-DR expression on CD8^+^ T cells, either alone or with CD38, was also observed in subjects immunized with both Tat doses with a peak at week 12 (Tat 7.5 µg, p = 0.0071; Tat 30 µg, p = 0.0001), remaining significantly higher at week 48 (Tat 7.5 µg, p = 0.0039; Tat 30 µg, p = 0.0023) (Fig. 4 B, C). The most evident effects were again observed with Tat 30 µg, for which the average frequency of CD38^+^/HLA-DR^+^ doubly-positive CD8^+^ T lymphocytes remained significantly higher up to week 48 (p = 0.0010).

Of note, a longitudinal analysis showed a significant correlation of CD38 downregulation on CD8^+^ T cells with increasing anti-Tat IgA titers following the Tat 30 µg immunization

[log10 IgA: β = -6.0% (95% CI −10.5%; −1.5%) p = 0.0087].

**Figure 5:**
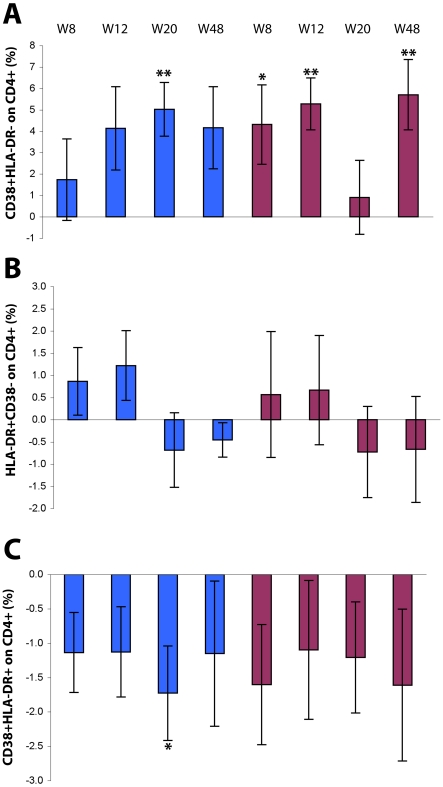
Expression of activation markers on CD4^+^ T cells after Tat immunization. Changes from baseline of CD4^+^ T cells (gating on CD4^+^ T cells) expressing (**A**) CD38, (**B**) HLA-DR, or (**C**) both CD38 and HLA-DR. Results are shown according to Tat dose and time after the first immunization. Data are presented as the mean % changes (± standard error) at week 8, 12, 20 and 48. Blue bars: Tat 7.5 µg, n = 17 up to week 20 and n = 12 at week 48; red bars: Tat 30 µg, n = 20 up to week 20, n = 15 at week 48, respectively; The t-Test for paired data was used for the analyses: *p<0.05, **p<0.01.

The modulation of the expression of these markers was somewhat opposite on CD4^+^ T cells (Fig. 5 A–C). In particular, the percentage of CD38 singly-positive CD4^+^ T cells was increased in immunized subjects with both Tat doses and up to week 48, remaining significant for Tat 30 µg (p = 0.0038), whereas HLA-DR expression had little changes after immunization. In contrast, the frequencies of CD38^+^/HLA-DR^+^ doubly-positive CD4^+^ T lymphocytes were reduced in immunized subjects at all time points and at both Tat doses.

### Biochemical markers of immune activation

**Figure 6:**
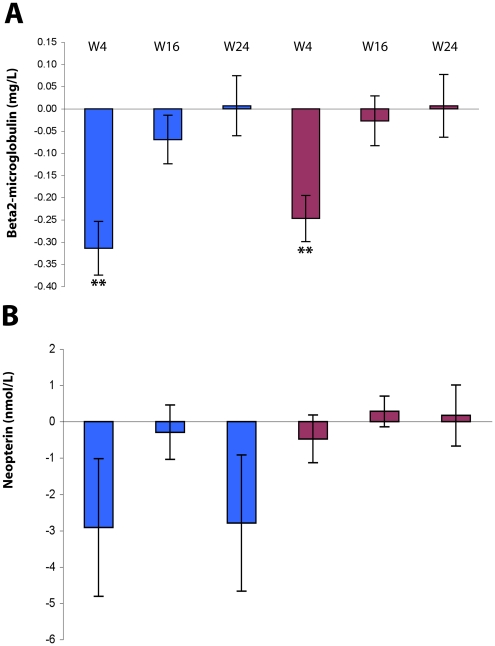
Production of β2-microglobulin and neopterin after Tat immunization. Changes from baseline, according to Tat dose, of (**A**) β2-microglobulin serum levels (mg/L) and (**B**) Neopterin (nmol/L). Blue bars: Tat 7.5 µg; red bars: Tat 30 µg. Data are presented as the mean changes (± standard error) at 4, 16 and 24 weeks (Tat 7.5 µg, n = 37, Tat 30 µg, n = 40 subjects, respectively). The t-Test for paired data was used for the analyses: **p<0.01.

The down-regulation of cellular markers of immune activation following Tat immunization was associated with an early decrease (week 4) in the serum levels of β2-microglobulin (p<0.0001) (Fig. 6 A), and neopterin (Fig. 6 B).

**Figure 7:**
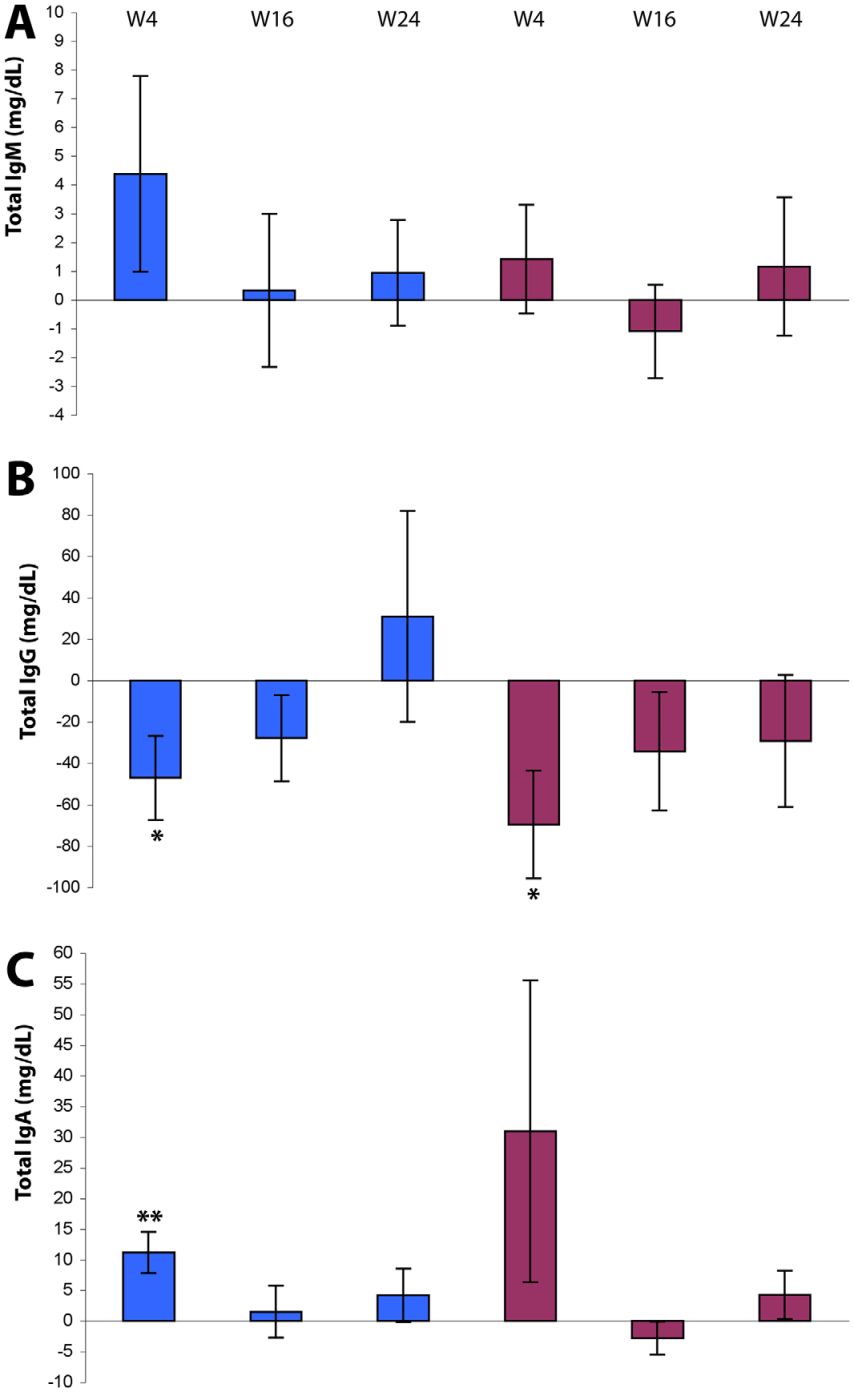
Production of total Ig after Tat immunization. Total IgM (**A**), IgG (**B**) and IgA (**C**) serum levels (mg/dL) are shown. Blue bars: Tat 7.5 µg, n = 37; red bars: Tat 30 µg, n = 40. Data are presented as the mean changes (± standard error). The t-Test for paired data was used for the analyses: *p<0.05, **p<0.01.

Different changes were observed for total Ig according to the isotype (Fig. 7 A–C). In particular, only total IgG were significantly decreased early (week 4) post-immunization and at both Tat doses (Tat 7.5 µg, p = 0.0258; Tat 30 µg, p = 0.0111), remaining thereafter below baseline levels only for Tat 30 µg.

### CD25 expression and T-regulatory Cells

**Figure 8:**
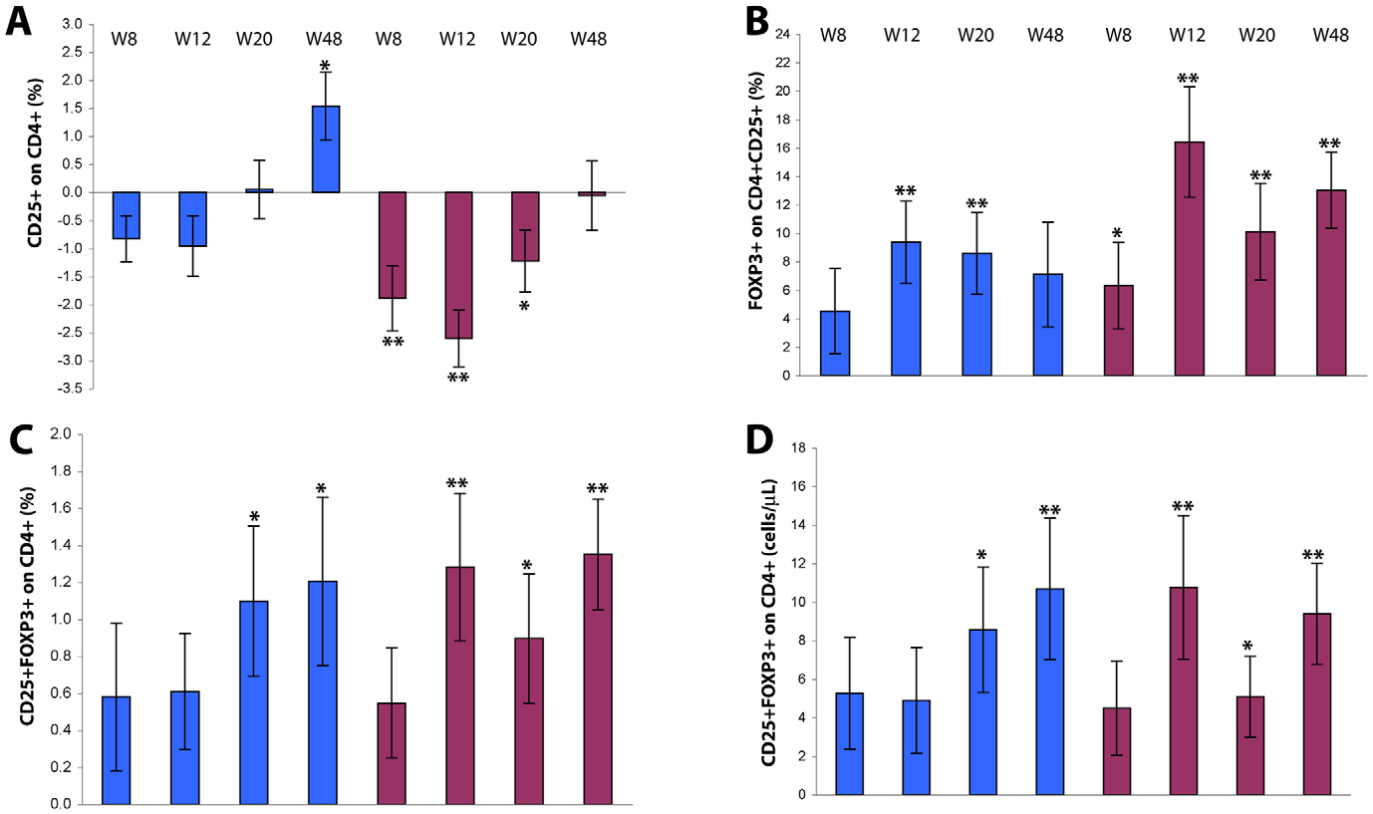
CD25 and FOXP3 expression on CD4^+^ T cells after Tat immunization. (**A**) Changes from baseline of CD4^+^ lymphocytes expressing CD25 are shown according to Tat dose and time after the first immunization (Tat 7.5 µg, n = 32 up to week 20 and n = 24 at week 48; Tat 30 µg, n = 34 up to week 20 and n = 28 at week 48, respectively). (**B**) Changes from baseline of the percentage of CD4^+^CD25^+^ lymphocytes expressing FOXP3^+^ (Tat 7.5 µg, n = 31 up to week 20 and n = 23 at week 48; Tat 30 µg, n = 29 up to week 20 and n = 28 at week 48, respectively). (**C**) Changes from baseline of the percentage of CD4^+^ T cells expressing CD25^+^FOXP3^+^ (Tat 7.5 µg, n = 31 up to week 20 and n = 23 at week 48; Tat 30 µg, n = 29 up to week 20 and n = 24 at week 48). (**D**) Changes from baseline of the absolute number of CD4^+^ lymphocytes expressing CD25^+^ FOXP3^+^ (Tat 7.5 µg, n = 30 up to week 20 and n = 20 at week 48; Tat 30 µg, n = 29 up to week 20 and n = 22 at week 48, respectively). Blue bars: Tat 7.5 µg; red bars: Tat 30 µg. Data are presented as the mean changes (± standard error) evaluated at 8, 12, 20 and 48 weeks after the first immunization. The t-Test for paired data was used for the analyses: *p<0.05, **p<0.01.

After Tat immunization, the percentage of total CD25^+^/CD4^+^ T cells markedly diminished returning to baseline values at week 48. This reduction was greater with Tat 30 µg, reaching a nadir at 12 weeks post-immunization and remaining significant at week 20 (p = 0.0351) (Fig. 8 A), before returning to baseline levels at week 48.

This finding was paralleled by a significant and persistent increase of the percentage of FOXP3 expression in the CD4^+^/CD25^+^ T cell subset as well as of the percentage and absolute number of CD4^+^/CD25^+^/FOXP3^+^ T-reg, which had a significant and progressive increase at both Tat doses and persisted up to week 48 (T-regs % at Tat 7.5 µg, p = 0.0146, at Tat 30 µg, p = 0.0002; T-regs number at Tat 7.5 µg, p = 0.0090, at Tat 30 µg, p = 0.0018) (Fig. 8 C, D).

The changes of immune activation markers and T-reg observed upon immunization were significantly and inversely related to baseline values (Table 6), in that the reduction in immune activation markers was more pronounced in subjects with the highest values at baseline. Conversely, the largest increase in T-reg cell number was seen in individuals with the lowest baseline values.

**Table 6:** Inverse correlation between baseline values and changes after Tat immunization of immune activation markers and T-regs.

ISS T-002	*n*	Pearson correlation	p-value
		coefficient	
CD38^+^HLA-DR^−^ on CD8^+^ T cells (%)	38	r = −0.5	0.0005
CD38^−^HLA-DR^+^ on CD8^+^ T cells (%)	38	r = −0.5	0.0023
CD38^+^HLA-DR^+^ on CD8^+^ T cells (%)	38	r = −0.4	0.0190
CD38^+^ HLA-DR^−^ on CD4^+^ T cells (%)	37	r = −0.3	0.0623
HLA-DR^+^CD38^−^ on CD4^+^ T cells (%)	37	r = −0.7	<0.0001
CD38^+^HLA-DR^+^ on CD4^+^T cells (%)	37	r = −0.8	<0.0001
β2-microglobulin (mg/L)	77	r = −0.3	0.0017
Neopterin (nmol/L)	77	r = −0.9	<0.0001
Total IgM (mg/dL)	77	r = −0.1	0.5371
Total IgG (mg/dL)	77	r = −0.4	0.0001
Total IgA (mg/dL)	77	r = 0.0	0.7519
CD25^+^ on CD4^+^ T cells (%)	66	r = −0.5	<0.0001
FOXP3^+^ on CD4^+^CD25^+^ T cells (%)	60	r = −0.5	0.0001
CD25^+^FOXP3^+^ on CD4^+^ T cells (%)	60	r = −0.3	0.0171

The relationship between baseline values and changes at week 20 or week 24 for all immunized subjects was evaluated by the Pearson correlation coefficient (r) after cumulating both Tat doses. *n* indicates the number of individuals evaluated for each parameter.

### Immunization with Tat increases PBMC viability and the number of CD4^+^ T cells and B cells

**Figure 9:**
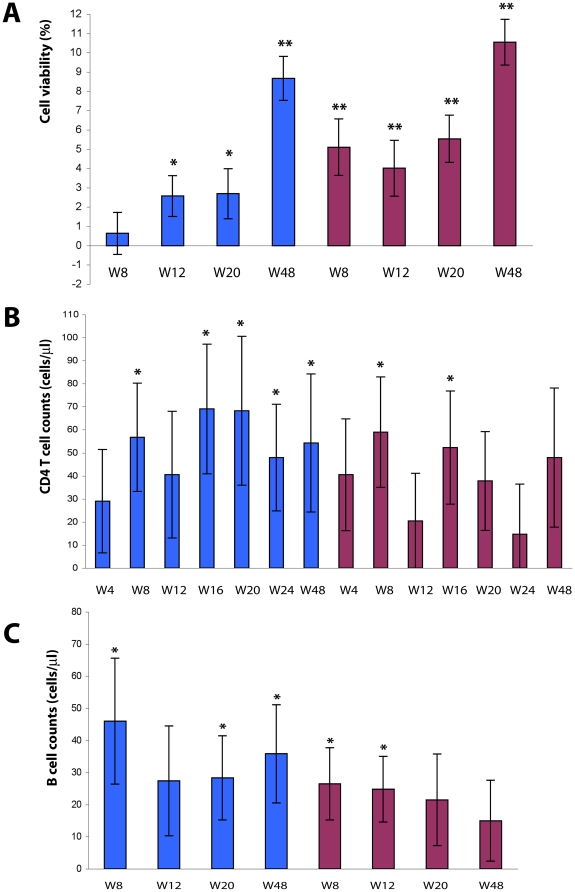
Evaluation of PBMC viability, CD4^+^ T cell and B cell counts after Tat immunization. (**A**) Changes from baseline of *in vitro* PBMC viability, stratified by Tat dose. Blue bars: Tat 7.5 µg, n = 35 up to week 20 and n = 32 at week 48; red bars: Tat 30 µg, n = 40 up to week 20 and n = 33 at week 48, respectively. (**B**) Changes from baseline of CD4^+^ T cells/µL (data from clinical sites), stratified by Tat dose. Blue bars: Tat 7.5 µg, n = 39 up to week 24 and n = 30 at week 48; red bars Tat 30 µg, n = 43 up to week 24 and n = 34 at week 48. (**C**) Changes from baseline of B cells/µL, stratified by Tat dose. Blue bars: Tat 7.5 µg, n = 38 up to week 20 and n = 30 at week 48; red bars Tat 30 µg, n = 40 up to week 20 and n = 30 at week 48, respectively. The t-Test for paired data was used for the analyses: *p<0.05, **p<0.01.

PBMC viability in vitro is reduced in HIV infection [88]. In contrast, progressive and significant increments of cell viability were seen in PBMC early after immunization (since week 8) with both Tat doses and particularly with Tat 30 µg. Cell viability continued to increase up to week 48 with both Tat doses (p<0.0001, Fig. 9 A).

The CD4^+^ T cell number increased after Tat immunization, at all time-points and for both Tat doses (Fig. 9 B), reaching statistical significance with the 7.5 µg Tat dose at week 8 (57 cells/µL, p = 0.0206), week 16 (69 cells/µL, p = 0.0188), week 20 (68 cells/µL, p = 0.0418), week 24 (48 cells/µL, p = 0.045) and week 48 (54 cells/µL, p = 0.0806), and for the 30 µg Tat dose at week 8 (59 cells/µL, p = 0.0181) and week 16 (52 cells/µL, p = 0.0388).

Similarly, the B cell number also increased upon immunization with both Tat doses and at all time-points (Fig. 9 C), reaching statistical significance with Tat 7.5 µg at week 8 (46 cells/µL, p = 0.0250), week 20 (28 cells/µL, p = 0.0368) and week 48 (66 cells/µL, p = 0.0261), as well as with Tat 30 µg at week 8 (26 cells/µL, p = 0.0241) and week 12 (25 cells/µL, p = 0.0200).

### Immunization with Tat increases the percentage of CD4^+^ T cells and B cells and reduces the percentage of CD8^+^ and NK cells independently from the type of HAART regimen

**Figure 10:**
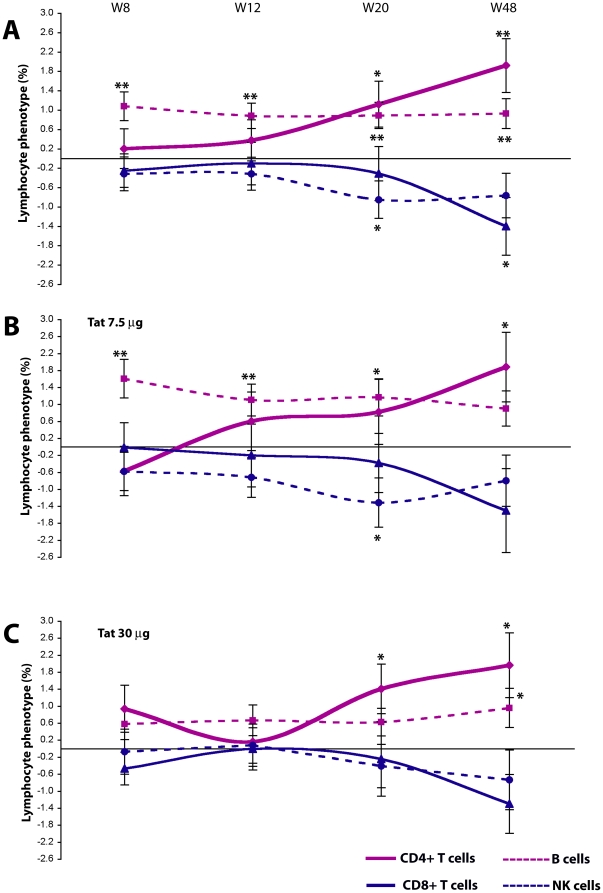
Evaluation of the percentage of CD4^+^, CD8^+^, NK and B cells in all Tat-immunized subjects and after stratification by Tat dose. (**A**) Changes from baseline of CD4^+^, CD8^+^, NK and B cells (percentage) for all immunized subjects (n = 78 up to week 20, n = 60 at week 48). (**B, C**) Changes from baseline of CD4^+^, CD8+, NK and B cells (percentage), stratified by Tat doses. (**B**) Tat 7.5 µg, n = 38 up to week 20 and n = 30 at week 48; (**C**) Tat 30 µg, n = 40 up to week 20 and n = 30 at week 48, respectively. The t-Test for paired data was used for the analyses: *p<0.05, **p<0.01.

The determination of the percentage of lymphocyte subsets confirmed the increase of CD4^+^ T cells and B cells observed for the absolute values, showing significant increases of CD4^+^ T cells, particularly at week 20 (1%, p = 0.0218) and 48 (2%, p = 0.0010), as well as significant increases of B cells at all time-points starting from week 8 (1%, p<0.01) (Fig. 10 A). In contrast, the percentage of NK and CD8^+^ T cells were significantly reduced at week 20 (−1%, p = 0.0288) and 48 (−1.4%, p = 0.0217), respectively. These effects were observed with both Tat doses (Fig. 10 B, C).

As a consequence, the CD4/CD8 ratio progressively increased in immunized subjects with the most evident effects at week 20 and 48 from the first immunization, and, particularly, for the 30 µg Tat dose (week 20, p = 0.0117; week 48, p = 0.0011).

**Figure 11:**
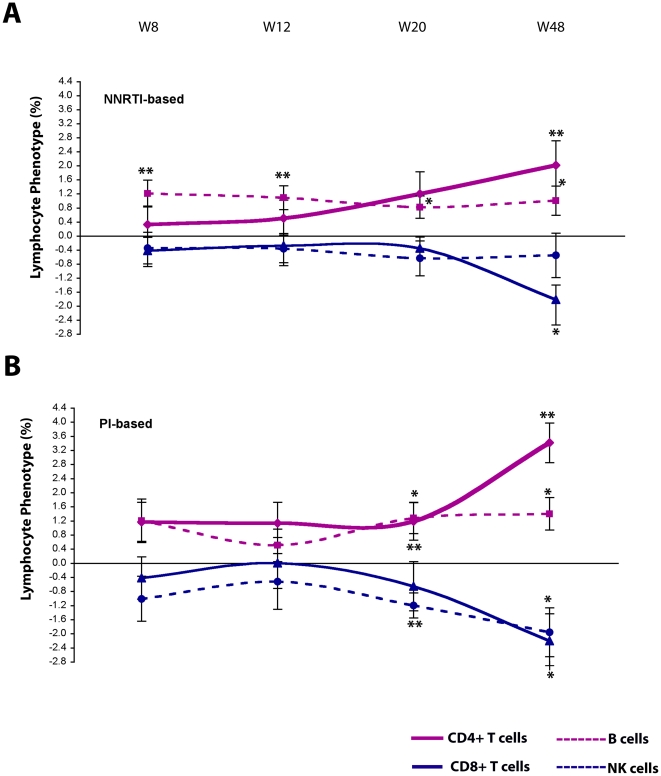
Evaluation of the percentage of CD4^+^, CD8^+^, NK and B cells in all Tat-immunized subjects after stratification by HAART regimens. Changes from baseline of CD4^+^, CD8^+^, NK and B cells (percentage) from all immunized patients are shown for NNRTI-based (**A**) and PI-based (**B**) treatments. NNRTI-based: n = 51 up to week 20 and n = 41 at week 48; PI-based: n = 19 up to week 20 and n = 12 at week 48, respectively. The t-Test for paired data was used for the analyses: *p<0.05, **p<0.01.

Stratification of these results according to the type of HAART regimen (NNRTI-based or PI-based) indicated that Tat immunization can overcome the differences seen with different drugs regimens on lymphocyte subsets, suggesting that the effects of Tat immunization are independent from the type of drugs combination (Fig. 11 A, B).

### Immunization with Tat increases central memory and reduces terminally- differentiated effector memory (Temra) CD4^+^ and CD8^+^ T cells

**Figure 12:**
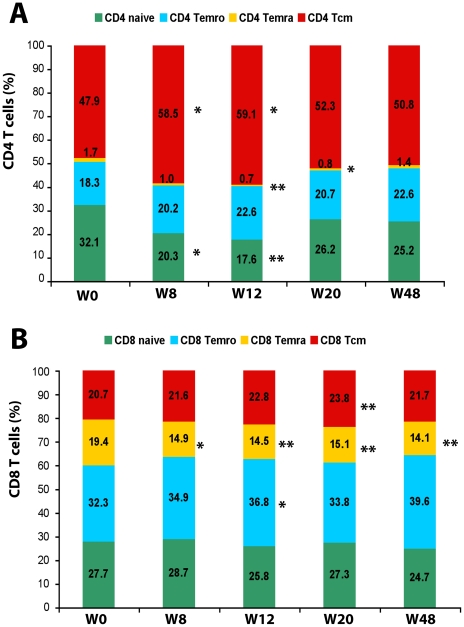
Effect of Tat immunization on naïve, central and effector memory CD4^+^ and CD8^+^ T cells. Percentage of naïve (CD45RA^+^/CD62L^+^), effector RA^+^ (CD45RA^+^/CD62L^−^, Temra) or RA- (CD45RA^−^/CD62L^−^, Temro) and central memory (CD45RA^−^/CD62L^+^, Tcm) CD4^+^ (**A**) or CD8^+^ (**B**) T cells at baseline and at week 8, 12, 20 and 48 after the first immunization for subjects immunized with both Tat doses (n = 18 up to week 20 and n = 10 at week 48). Asterisk indicates significant changes from baseline. The t-Test for paired data was used for the analyses: *p<0.05, **p<0.01.

Since reduction of central memory T cells (Tcm) with a concomitant increase of effector memory cells (Tem) is commonly observed in HIV infection and this unbalance is only partially restored under effective HAART [5], these T cell subsets were evaluated upon immunization with Tat. The percentage of central (CD45RA^−^/CD62L^+^) and, to a lesser extent, effector (CD45RA^−^/CD62L^+^, Temro) memory CD4^+^ and CD8^+^ T cells increased upon Tat immunization (Fig. 12).

For CD4^+^ T cells, these findings were concomitant with a reduction of both terminally-differentiated Tem (CD45RA+/CD62L^−^, Temra) and naïve (CD45RA^+^/CD62L^+^) T cells (Fig. 12 A). These changes were evident 8 weeks after the first immunization, peaked at week 12 (Tcm, p = 0.0176; Temra, p = 0.0080; naïve T cells, p = 0.0018) and declined thereafter but did not return to baseline values (Fig. 12 A).

For CD8^+^ T cells the earliest and most prominent changes were observed for Temra, which were significantly reduced at all time-points post-immunization, reaching a nadir at week 48 (Fig. 12 B). In contrast, the increase in Tcm was less pronounced and slower for CD8^+^ cells as compared to CD4^+^ cells, reaching statistical significance only at week 20 (p<0.0001). As for CD4^+^ T cells, also the percentage of CD8^+^ Temro increased after immunization, reaching statistical significance at week 12 (p = 0.0378). Unlike the CD4^+^ counterpart, the CD8^+^ naïve subset remained largely unaffected by the treatment (Fig. 12 B).

### Immunization with Tat increases cellular responses to HIV-Env and to recall antigens

**Figure 13:**
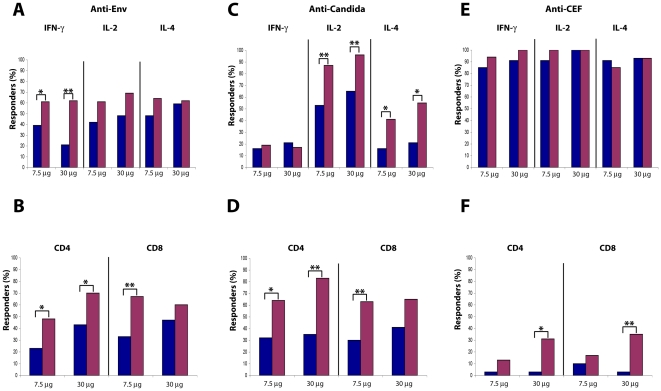
Cellular immune responses against Env or recall antigens after Tat immunization. Percentage of responders at baseline (blue bar) and up to week 48 (red bar) are stratified by Tat dose. Percentage of subjects showing (**A**) anti-Env production of IFN-γ, IL-2 and IL-4 (Tat 7.5 µg, n = 31; Tat 30 µg, n = 29) and (**B**) CD4^+^ or CD8^+^ lymphoproliferative responses (Tat 7.5 µg, n = 31; Tat 30 µg, n = 30); (**C**) anti-Candida cytokines production (Tat 7.5 µg, n = 32; Tat 30 µg, n = 29), and (**D**) CD4^+^ or CD8^+^ lymphoproliferative responses (Tat 7.5 µg, n = 31; Tat 30 µg, n = 29); (**E**) anti-CEF production of IFN-γ, IL-2 and IL-4 (Tat 7.5 µg, n = 34; Tat 30 µg, n = 32), and (**F**) CD4^+^ or CD8^+^ lymphoproliferative responses (Tat 7.5 µg, n = 31; Tat 30 µg, n = 29). The analysis was performed using the McNemar's test: *p<0.05, **p<0.01.

To verify whether the effects of Tat-immunization were accompanied by changes in adaptive immunity against heterologous antigens, T cell responses against HIV-Env, Candida, or CEF antigens were determined by monitoring Th1 and Th2 cytokine production and CD4^+^ and CD8^+^ T cell proliferation. An increase in both the percentage of responders and the intensity of responses was found in subjects immunized with both Tat doses (Fig. 13 A–F and Table 7–9). In particular, statistically significant increases in the percentage of responders were detected against Env for IFN-γ (Tat 7.5 µg, p = 0.0348; Tat 30 µg, p = 0.0005), and against Candida for IL-2 (Tat 7.5 µg, p = 0.0076; Tat 30 µg, p = 0.0027) and IL-4 (Tat 7.5 µg, p = 0.0114; Tat 30 µg, p = 0.0184). Statistically significant increases of CD4^+^ and CD8^+^ T cell proliferation were detected against Env (for CD4^+^ at Tat 7.5 µg, p = 0.0114, at Tat 30 µg, p = 0.0455; for CD8^+^ at Tat 7.5 µg, p = 0.0016), and Candida (for CD4^+^ at Tat 7.5 µg, p = 0.0253, at Tat 30 µg, p = 0.0010; for CD8^+^ at Tat 7.5 µg, p = 0.0039) (Fig. 13 A–D). Cytokine production in response to CEF was already present in most subjects with no differences after immunization (Fig. 13 E). In contrast, CD4^+^ and CD8^+^ T cell proliferation to CEF were very low at baseline and increased after immunization particularly upon vaccination with 30 µg of Tat for both CD4^+^ (p = 0.0114) and CD8^+^ (p = 0.0067) T cell subsets (Fig. 13 F).

**Table 7:** Cellular immune responses against Env after Tat immunization.

ISS T-002		Tat 7.5 µg^b^			Tat 30 µg^c^	
	*n*	Baseline	Up to week 48	*n*	Baseline	Up to week 48
**IFN-γ**						
Peak^a^ (SFC/10^6^ cells)	19	44 (0–394)	382 (86–834)**	18	53 (12–226)	236 (86–920)**
**IL-2**						
Peak^a^ (SFC/10^6^ cells)	19	52 (18–76)	94 (52–166)**	20	26 (14–49)	49 (38–95)**
**IL-4**						
Peak^a^ (SFC/10^6^ cells)	20	52 (16–122)	137 (44–464)**	18	24 (11–64)	80 (28–132)
**CD4 Proliferation**						
Peak^a^ (fold increase)	15	1.5 (0.8–3.8)	2.4 (2.1–4.7)*	21	1.5 (0.8–2.8)	2.9 (2.2–4.5)**
**CD8 Proliferation**						
Peak^a^ (fold increase)	20	2.0 (1.1–4.1)	3.1 (2.5–7.2)*	18	2.7 (1.1–5.0)	5.6 (3.9–7.0)*

IFN-γ, IL-2, IL-4 production by PBMC, and CD4 or CD8 lymphoproliferative responses to Env were measured at baseline and up to week 48 after the first immunization, respectively. Results are stratified by Tat doses, (7.5 and 30 µg). *n* indicates the number of responders for cytokines production and CD4 or CD8 T cell proliferation, respectively. The median intensity with interquartile range of peak of responses is shown. Pre-post vaccination median change was evaluated by Wilcoxon signed-rank test.

a Median (interquartile range) of peak of responses, weeks 8, 12, 20, 48.

b Total subject tested for cytokines: 31; for proliferation: 31.

c Total subjects tested for cytokines: 29; for proliferation: 30.

*P<0.05, ** *P*<0.01.

**Table 8 pone-0013540-t008:** Cellular immune responses against Candida after Tat immunization.

ISS T-002		Tat 7.5 µg^b^			Tat 30 µg^c^	
	*n*	Baseline	Up to week 48	*n*	Baseline	Up to week 48
**IFN-γ**						
Peak^a^ (SFC/10^6^ cells)	6	40 (0–70)	69 (46–150)	5	82 (82–118)	132 (82–266)
**IL-2**						
Peak^a^ (SFC/10^6^ cells)	28	98 (28–181)	175 (101–346)**	28	73 (48–111)	133 (93–259)**
**IL-4**						
Peak^a^ (SFC/10^6^ cells)	13	8 (0–34)	66 (36–124)**	16	6 (0–13)	60 (34–110)**
**CD4 Proliferation**						
Peak^a^ (fold increase)	20	1.6 (1.2–2.0)	4.8 (2.3–6.1)**	24	1.7 (1.2–2.8)	4.1 (2.8–4.9)**
**CD8 Proliferation**						
Peak^a^ (fold increase)	19	1.8 (1.3–4.6)	4.9 (2.8–9.9)**	19	1.5 (1.2–3.8)	5.7 (3.9–8.9)**

IFN-γ, IL-2, IL-4 production by PBMC, and CD4 or CD8 lymphoproliferative responses to Candida were measured at baseline and up to week 48 after the first immunization, respectively. Results are stratified by Tat doses, (7.5 and 30 µg). *n* indicates the number of responders for cytokines production and CD4 or CD8 T cell proliferation, respectively. The median intensity with interquartile range of peak of responses is shown. Pre-post vaccination median change was evaluated by Wilcoxon signed-rank test.

a Median (interquartile range) of peak of responses, weeks 8, 12, 20, 48.

b Total subject tested for cytokines: 32; for proliferation: 29.

c Total subjects tested for cytokines: 29; for proliferation: 29.

**P<0.05, ** *P*<0.01.

**Table 9:** Cellular immune responses against CEF after Tat immunization.

ISS T-002		Tat 7.5 µg^b^			Tat 30 µg^c^	
	*n*	Baseline	Up to week 48	*n*	Baseline	Up to week 48
**IFN-γ**						
Peak^a^ (SFC/10^6^ cells)	32	713 (332–1144)	1244 (750–1684)**	32	1054 (326–1659)	1754 (1064–3947)**
**IL-2**						
Peak^a^ (SFC/10^6^ cells)	34	270 (152–500)	525 (372–638)**	31	266 (142–752)	706 (496–1108)**
**IL-4**						
Peak^a^ (SFC/10^6^ cells)	29	298 (140–736)	728 (312–1132)**	29	504 (148–660)	678 (518–986)**
**CD4 Proliferation**						
Peak^a^ (fold increase)	4	0.9 (0.8–1.2)	4.2 (3.4–4.9)	9	0.7 (0.7–1.1)	2.8 (2.6–3.0)**
**CD8 Proliferation**						
Peak^a^ (fold increase)	5	2.3 (1.6–2.7)	2.9 (2.0–3.2)	10	1.0 (0.7–1.2)	3.4 (2.2–4.8)**

IFN-γ, IL-2, IL-4 production by PBMC, and CD4 or CD8 lymphoproliferative responses to CEF were measured at baseline and up to week 48 after the first immunization, respectively. Results are stratified by Tat doses, (7.5 and 30 µg). *n* indicates the number of responders for cytokines production and CD4 or CD8 T cell proliferation, respectively. The median intensity with interquartile range of peak of responses is shown. Pre-post vaccination median change was evaluated by Wilcoxon signed-rank test.

a Median (interquartile range) of peak of responses, weeks 8, 12, 20, 48.

b Total subject tested for cytokines: 34; for proliferation: 31.

c Total subjects tested for cytokines: 32; for proliferation: 29.

**P<0.01.

The intensity (peak values) of both cytokine production and proliferative responses to Env as well as to recall antigens including CEF were significantly increased after immunization (p<0.05, Table 7–9).

### Correlations of Tat immunization with changes in the T cell compartments

**Figure 14:**
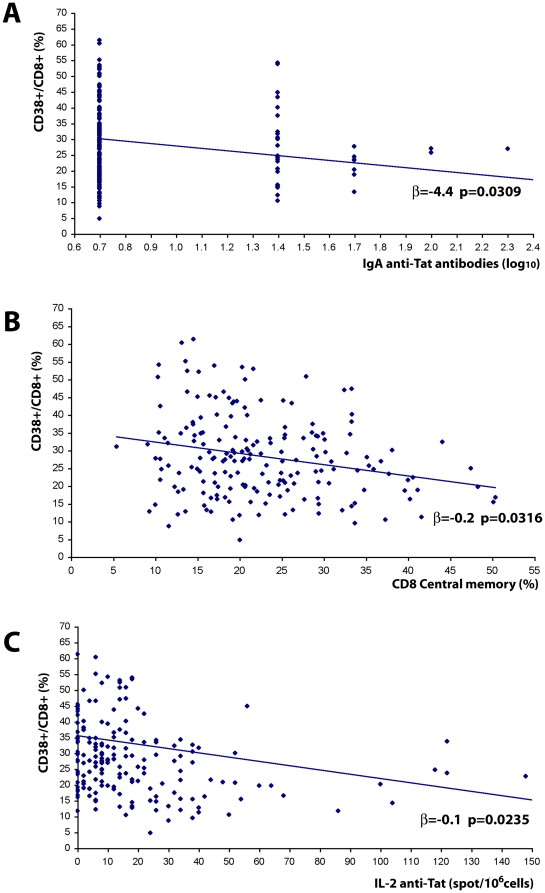
Correlation of the reduction of the percentage of CD38^+^/CD8^+^T cells with the increase of anti-Tat IgA antibody titers, CD8^+^ T central memory and anti-Tat specific IL-2 production after Tat immunization. Multivariate regression model for repeated measures was applied to CD38^+^/CD8^+^ T cells (%), including as explicative factors anti-Tat antibody titers (IgM, IgG, IgA), CD8^+^ central memory (%) and anti-Tat IFN-γ, IL-2 and IL-4 cytokines, for immunized subjects (n = 70).

A multivariate regression analysis was used to assess the presence of potential correlations among the different parameters investigated after therapeutic immunization. A statistically significant inverse correlation was found between the percentage of CD38^+^/CD8^+^ T cells with anti-Tat IgA titers (p = 0.0309), CD8^+^ Tcm lymphocytes (p = 0.0316), and with IL-2 production in response to Tat (p = 0.0235) (Fig. 14 A–C), suggesting a direct relationship between the induction of anti-Tat specific IL-2 producing cells and increasing anti-Tat IgA titers with the expansion of CD8**^+^** Tcm and the reduction of activated CD8^+^ effector T cells.

### Comparison of Tat-immunized subjects with patients enrolled in the observational Study (ISS OBS T-002)

Although the effects and the limits of a successful HAART are well known [89], an intergroup comparison between trial subjects and those enrolled in the parallel ISS OBS T-002 study was made to better evaluate the effect of Tat immunization on virologically-suppressed patients (see Methods section, Protocol S2 and Supplementary material). Out of 88 evaluable patients (Total OBS Subjects) enrolled in this study (see Methods section), 32 subjects fully matched the baseline characteristics of the trial participants (Fig. S1 and Table S1). This group was considered as the Reference Group, and comparison of immunized subjects was made with both this Reference Group and the Total OBS Subjects for all assessed parameters (Table 10, 11). The baseline values of the OBS subjects for activation markers and T-reg lymphocytes were determined at study entry (Table S2).

**Table 10:** Qualitative comparison of the results of the ISS T-002 Clinical Trial and the ISS OBS T-002 Observational Study up to 48 weeks of follow-up: activation markers, T-regs, cell viability and lymphocyte phenotype.

	ISS T-002^a^	Reference Group^b^	Total OBS Subjects^c^
**Activation markers**			
*Phenotypic* (%)			
CD38^+^HLA-DR^−^ on CD8^+^ T cells	− (∗)	−	− (∗)
HLA-DR^+^CD38^−^ on CD8^+^ T cells	+ (∗)	− (∗)	− (∗)
CD38^+^HLA-DR^+^ on CD8^+^ T cells	+ (∗)	−	− (∗)
CD38^+^ HLA-DR^−^ on CD4^+^ T cells	+ (∗)	− (∗)	− (∗)
HLA-DR^+^CD38^−^ on CD4^+^ T cells	+/−	− (∗)	− (∗)
CD38^+^HLA-DR^+^ on CD4^+^T cells	− (∗)	−	− (∗)
CD25^+^ on CD4^+^ T cells	− (∗)	+	+
*Biochemical*			
β2-microglobulin (mg/L)	− (∗)	+/−	n.d.
Neopterin (nmol/L)	−	+	n.d.
Total IgM (mg/dL)	+/−	+	n.d.
Total IgG (mg/dL)	− (∗)	+	n.d.
Total IgA (mg/dL)	+ (∗)	+	n.d.
**T-regs**			
FOXP3^+^ on CD4^+^CD25^+^ T cells (%)	+ (∗)	− (∗)	− (∗)
CD25^+^FOXP3^+^ on CD4^+^ T cells (%)	+ (∗)	−	−
CD25^+^FOXP3^+^ on CD4^+^ T cells (cells/µL)	+ (∗)	−	− (∗)
**Cell viability (%)**	+ (∗)	+ (∗)	+ (∗)
**Lymphocyte cell counts** (cells/µL)			
CD4^+^ T cell counts	+ (∗)	+/−	+
B cell counts	+ (∗)	−	−
**Lymphocyte Phenotype** (%)			
CD4^+^ T cell	+ (∗)	+	+
CD8^+^ T cell	− (∗)	+	−
NK	− (∗)	+/−	=
B	+ (∗)	−	+/−
CD4/CD8 ratio	+ (∗)	+	+

a Subjects enrolled in the clinical trial; ^b^subjects enrolled in the OBS study with the same characteristics at baseline of the trial patients; ^c^ all evaluable subjects enrolled in the OBS study. Shown are the qualitative changes from baseline of the different study populations for all evaluated parameters. +, increase; −, decrease; +/−, fluctuations (higher and lower levels as compared to baseline at different time points);  =  stable; (∗), statistically significant in at least one time point; n.d., not done.

**Table 11:** Qualitative comparison of the results of the ISS T-002 and the ISS OBS T-002 up to 48 weeks: central and effector memory T cell phenotype and cellular immune responses.

	ISS T-002^a^	Reference Group^b^	Total OBS Subjects^c^
**T cell phenotype** (%)			
CD4^+^ naïve	− (∗)	+/−	−
CD4^+^ Tcm	+ (∗)	+/−	+
CD4^+^ Temro	+	+	+
CD4^+^ Temra	− (∗)	+/−	+/−
CD8^+^ naïve	+/−	−	+/−
CD8^+^ Tcm	+ (∗)	+/−	+
CD8^+^ Temro	+ (∗)	+	+
CD8^+^ Temra	− (∗)	+/−	+/−
**Anti-Tat (% responders)**			
IFN-γ production	+	+	+ (∗)
IL-2 production	+ (∗)	=	+ (∗)
IL-4 production	+	+ (∗)	+
CD4^+^ proliferation	+ (∗)	+	+
CD8^+^ proliferation	+	+ (∗)	+ (∗)
**Anti-Env (% responders)**			
IFN-γ production	+ (∗)	+	+
IL-2 production	+	−	−
IL-4 production	+	=	=
CD4^+^ proliferation	+ (∗)	+	+ (∗)
CD8^+^ proliferation	+ (∗)	+	+ (∗)
**Anti-Candida (% responders)**			
IFN-γ production	=	=	+
IL-2 production	+ (∗)	−	−
IL-4 production	+ (∗)	+	+ (∗)
CD4^+^ proliferation	+ (∗)	+	+ (∗)
CD8^+^ proliferation	+ (∗)	+	+ (∗)
**Anti-CEF (% responders)**			
IFN-γ production	=	+ (∗)	+ (∗)
IL-2 production	=	+	+ (∗)
IL-4 production	=	+ (∗)	+ (∗)
CD4^+^ proliferation	+ (∗)	+	+
CD8^+^ proliferation	+ (∗)	+	+

a Subjects enrolled in the clinical trial; ^b^subjects enrolled in the OBS study with the same characteristics at baseline of the trial patients; ^c^ all evaluable subjects enrolled in the OBS study. +, increase; −, decrease; +/−, fluctuations (higher and lower levels as compared to baseline at different time points);  =  stable; (∗), statistically significant in at least one time point.

A decrease of CD38 and HLA-DR expression on CD8^+^ T cells either alone or in combination was observed during the follow up for both Total OBS Subjects and Reference Group (Fig. S2, A–F). These results are in sharp contrast with the upregulation of HLA–DR expression, alone or combined with CD38, observed on CD8^+^ T cells from immunized subjects (Fig. 4 A–C).

The modulation of CD38 and HLA–DR expression on CD4^+^ T cells of OBS subjects was similar to that observed on CD8^+^ T cells (Fig. S2 G–L). Thus, the pattern of expression of these two activation markers differed from what observed in the immunized subjects in whom an opposite and statistically significant upregulation of CD38 expression on CD4^+^ T cells was observed (Fig. 5 A–C).

The biochemical markers of immune activation were scarcely modified in the Reference Group throughout follow-up (Fig. S3 A–E). Specifically, β2-microglobulin showed an early increase and then a gradual but slight decrease, while neopterin was slightly but persistently increased respect to baseline values (Fig. S3 A, B). In contrast, both these activation markers were reduced at most time-points in immunized patients (Fig. 6 A, B).

Total IgM, IgG and IgA remained substantially stable in OBS subjects for most of the follow up (Fig. S3 C–E), whereas a significant reduction of total IgG was recorded early after immunization in trial subjects (Fig. 7 A–C).

Overall, in the OBS patients the percentage of total CD25^+^/CD4^+^ T cells was stable, with an increase at week 48 (Fig. S4 A and Fig. S5 A). Conversely, the percentage of CD4^+^/CD25^+^ T cells expressing FOXP3 gradually decreased in the Total OBS Subjects (Fig. S4 B) as well as in the Reference Group (Fig. S5 B). In addition, a decrease was observed in the percentage and absolute number of the CD4^+^/CD25^+^/FOXP3^+^ T-reg subset in the Total OBS Subjects (Fig. S4 C, D), whereas the decline was less pronounced in the Reference Group (Fig. S5 C, D). The opposite was observed in the patients immunized with Tat, in which an overall reduction of CD25 expression and a concomitant and persistent statistically significant increase of T-reg were detected (Fig. 8 A–D).

Cell viability increased at late time-points (36 and 48 weeks) both in the Total OBS Subjects and in the Reference Groups (Fig. S6 A, B), as opposed to immunized individuals in whom a significant and steady increase of cell viability was detected as early as 8 weeks after the first immunization, particularly with the 30 µg Tat dose (Fig. 9 A).

No significant changes of the CD4^+^ T cell number were detected in individuals from either the Total OBS (Fig. S6 C) or the Reference Group (Fig. S6 D). This is in contrast with the statistically significant and persistent increase of CD4^+^ T cells observed in the subjects immunized with Tat (Fig. 9 B).

Further, a progressive loss of B cells was observed in both OBS groups (Fig. S6 E, F), a finding in striking contrast with the early, durable and statistically significant increments of B cells observed in the Tat immunized subjects (Fig. 9 C).

These differences between the trial and OBS groups were also reflected by the percentage of lymphocyte subsets. In fact, as compared to baseline levels, in the OBS study were detected a moderate increase of the CD4^+^ T cell percentage, an overall stability (Total OBS Subjects) or decrease (Reference Group) of the B cell subset (Fig. S7 A, B), a stable (Total OBS Subjects) or further increased (Reference Group) percentage of CD8^+^ T cells, and a substantial stability of the NK subset. Again, opposite trends were apparent in immunized patients in whom a persistent increase of the percentage of CD4^+^ T and B cells and a progressive decrease of CD8^+^ T cells and NK cells were recorded, irrespective of the type of HAART regimen (Fig. 11 A, B). In this regard, analysis of OBS subjects after stratification by NNRTI-based or PI-based treatment showed a different profile of lymphocytes subsets, particularly for B cells (Fig. S7 C–E), indicating a positive effect of Tat immunization in HAART intensification independently from the type of drugs combination.

The percentage of central memory (CD45RA^−^/CD62L^+^) CD4^+^ (Fig. S8 A, C) and CD8^+^ (Fig. S8 B, D) T cells was stable or moderately increased, though not significantly, at week 24 both for the Total OBS and Reference Group, as opposed to the more pronounced increments that were recorded in immunized subjects (Fig. 12 A, B). In the effector memory CD4^+^ and CD8^+^ compartment a reduction of the Temra (CD62L^−^/CD45RA+) subset and a concomitant increase of the Temro (CD62L^−^/CD45RA-) subpopulation were observed at week 24 in the OBS subjects (Fig. S8, A–D), while similar but earlier and more pronounced changes were evident in the immunized patients (Fig. 12 A, B). Finally, individuals from both the trial and the OBS study experienced a decline of naïve (CD45RA^+^/CD62L^+^) CD4^+^ and CD8^+^ T cells (Fig. 12 and Fig. S8 A–D).

The cumulative assessment of the cellular immune responses to Tat (up to 48 weeks of follow up) revealed an increase of the percentage of responders in terms of both specific cytokines production, except for IL-2 production in the Reference Group, and particularly of CD8^+^ T cell proliferation in both the Total OBS Subjects and the Reference Group (Fig. S9 A, B and Fig. S10 A, B). These changes were also quantitative, in that an increase in the peak values of cytokine production and CD8^+^ T cell proliferation was also detected for both groups (Table S3). In contrast, greater and statistically significant increments were seen for IL-2 production and CD4^+^ T cell proliferation in subjects immunized with Tat.

The percentage of responders and the intensity of responses were also evaluated for HIV-Env, Candida and CEF antigens in both the Total OBS Subjects and in the Reference Group (Fig. S9 C–H, Fig. S10 C–H and Tables S4, S5, S6). No changes were observed in cytokines production to Env, while the number of responders for CD4^+^ and CD8^+^ T cell proliferation against Env increased in the follow-up period in both the Total OBS Subjects (Fig. S9 D) and in the Reference Group (Fig. S10 D). In Total OBS Subjects, the percentage of responders to Candida increased for IL-4 production as well as for CD4^+^ and CD8^+^ T cell proliferation (Fig. S9 E, F). Similar trends were observed for the Reference Group (Fig. S10 E, F). Increases in the percentage of responders to CEF were found for all cytokines, while no relevant changes were observed for lymphoproliferative responses for both Total OBS Subjects and the Reference Group (Fig. S9 G, H and Fig. S10 G, H).

In addition, in both Total OBS Subjects and in the Reference Group, the intensity (peak values) of proliferative responses but not cytokine production to Env were increased, whereas for recall antigens both cytokine production and T cell proliferation were increased (Tables S4, S5, S6).

Overall, and in contrast to OBS subjects, in immunized subjects there was an increase of cytokine production to Env and Candida and of CD4^+^ and CD8^+^ proliferation to CEF (Fig. 13 B, D, F).

## Discussion

HAART represents a major virus-targeting intervention against HIV [1]. However, in spite of its success at suppressing HIV replication, HAART can only partially reduce chronic immune activation, and revert the immune dysregulation seen in treated individuals [1]–[8], [19]. Since Tat is persistently expressed in cell reservoirs also under HAART and has important effects on the virus and on the immune system, we hypothesized that Tat was a key factor for disease maintenance in drug-treated individuals.

Here we show that therapeutic immunization with Tat further reduces the immune activation still present under HAART [1]–[8], [19], and found also here in OBS subjects. In particular, Tat-immunized individuals, particularly at the 30 µg dose, experienced a significant reduction of CD38^+^/CD8^+^ T cells, which was inversely related to the anti-Tat IgA titers, central memory CD8^+^ T cells, and IL-2 production in response to Tat.

The decrease of CD38^+^/CD8^+^ T cells observed after immunization was associated with upregulation of HLA-DR expression on CD8^+^ T cells, either alone or in association with CD38, and with an increase of CD38 expression on CD4^+^ T cells. The pattern of the expression of HLA-DR, either alone or in association with CD38, on CD8^+^ T cells in response to immunization was opposite to what was observed in OBS subjects and it is consistent with that induced in healthy individuals by other vaccinations [90]. Notably, CD8^+^ T cells expressing HLA-DR and CD38 have been reported to possess optimal antiviral functionality [91], [92], whereas expression of CD38 on CD4^+^ T cells has been associated with reduced susceptibility to productive HIV infection in vitro [93] and in lymph nodes [94].

Further, although co-expression of DR and CD38 on CD8^+^ T cells has been extensively reported to be associated with immune activation in HIV progression [95], it is also found in immature and rapidly cycling T cells [96]–[99]. This suggests that co-expression of DR and CD38 may also result from restoration of homeostatic mechanisms upon therapeutic vaccination with Tat. Such a scenario is consistent with the increase of CD4^+^ and CD8^+^ central memory T cells and with the recovery of T cell responses to HIV and recall antigens, which were observed in vaccinated individuals. It is therefore tempting to speculate that Tat immunization reduces the HIV-driven immune dysregulation and associated anergy, and promotes restoration of proper and effective immune responses. This may explain the apparently paradoxical increment of doubly positive (CD38^+^/DR^+^) activated CD8^+^ T cells despite the concomitant reduction of the other soluble and cellular inflammation and immune activation markers, including CD38^+^/DR^+^ CD4^+^ T cells, and the increase of both T-regs and central memory CD8^+^ T cells.

In particular, the frequency and number of T-reg lymphocytes, which are known to suppress immune activation [100], were stably increased upon Tat immunization, as observed in elite controllers [101]. In contrast, they are progressively reduced in HIV-infected individuals, even under successful HAART [102], as observed also here in OBS subjects.

The increase of T-reg and the reduction of immune activation were associated with stable increases of the percentage and absolute numbers of both CD4^+^ T cells and B lymphocytes, and with the reduction of the percentage of CD8^+^ T cells and NK lymphocytes. As a result the CD4/CD8 T cell ratio progressively increased. Such a pattern of T and B cell “repopulation” differs markedly from that reported to occur during HAART [8] and observed here in the OBS group.

Of note, although differences were observed in the percentage of lymphocyte subsets with different HAART regimens (NNRTI-based versus PI-based) in the OBS subjects, Tat immunization had the same effects independently from the type of therapy, suggesting that Tat can intensify different HAART regimens.

In such a scenario, the increment or *de novo* appearance of cellular adaptive immune responses against HIV Env or to recall antigens suggests that restoration of key immune parameters takes place upon Tat immunization. In fact, these results were associated with increases in CD4^+^ and CD8^+^ central memory and functional effector memory T cells [103], [104], and with a reduction of terminally-differentiated, and functionally exhausted, effector counterparts. This is different from the trend seen in HIV-treated disease (and also observed in OBS), where central memory CD4^+^ and CD8^+^ T cell subsets are incompletely restored and effector memory T cells remain persistently increased (Table 10, 11) [5].

Except for the reduction of CD38 on CD8^+^ T cells and CD25 on CD4^+^ T cells, the effects of vaccination were long-lived since they were still present or even increased after 48 weeks from the first immunization.

The data indicate that immunization with Tat acts in synergy with HAART to help restoring immune homeostasis. Indeed, the therapeutic effects of Tat immunization were more pronounced in the patients that were more immune dysregulated at baseline.

The extended follow-up of the vaccinees will provide information on the need or timing of vaccine boosting.

## Supporting Information

Figure S1 Flow diagram of ISS OBS T-002 study participants. One hundred and twenty-seven subjects were recruited in the observational study ISS OBS T-002. Among them, 25 individuals were anti-Tat Ab positive and 91 anti-Tat Ab negative, respectively. Evaluable subjects were constituted by 88 anti-Tat Ab negative (Total OBS Subjects) and 32 Reference Group subjects, respectively. The Total OBS Subjects included anti-Tat Ab negative volunteers of either gender, ≥18 years old, under successful HAART (chronic suppression of HIV infection with a plasma viremia <50 copies/ml in the last 6 months and without a history of virologic rebound), a known nadir level of CD4+ T cells and CD4+ T cell number at study entry. The Reference Group included subjects having at baseline the same characteristics of volunteers enrolled in the ISS T-002 clinical trial: anti-Tat Ab negative (18–55 years of age), HAART-treated with chronic suppressed HIV infection, with levels of plasma viremia <50 copies/ml in the last 6 months prior to the screening and without a history of virologic rebound, CD4+ T cell counts ≥400 cells/µL and pre-HAART CD4 nadir >250 cells/µL.
(2.39 MB TIF)

Figure S2 Expression of activation markers on CD8+ and CD4+ T cells in subjects of ISS OBS T-002. Changes from baseline of CD8+ T cells (gating on CD8+ cells) expressing (A, B) CD38, (C, D) HLA-DR, or (E, F) both CD38 and HLA-DR in the Total Subjects (A, C, E) and in the Reference Group (B, D, F), respectively. Data are presented as the mean % changes (±standard error) at week 12, 24 and 36. Blue bars: Total Subjects n = 16 at week 12, n = 6 at week 24 and n = 6 at week 36; light violet bar: Reference Group, n = 6 at week 12, n = 3 at week 24. The t-Test for paired data was used for the analyses: *p<0.05, **p<0.01. Total Subjects: CD38+HLA-DR- at week 24, p = 0.0121; HLA-DR+CD38- at week 12, p = 0.0019; HLA-DR+CD38+ at week 12, p = 0.0019. Reference Group: HLA-DR+CD38- at week 12, p = 0.0256. Changes from baseline of CD4+ T cells (gating on CD4+ cells) expressing (G, H) CD38, (I, J) HLA-DR, or (K, L) both CD38 and HLA-DR in the Total Subjects (G, I, K) and in Reference Group (H, J, L), respectively. Data are presented as the mean % changes (±standard error) at week 12, 24 and 36. Blue bars: Total Subjects n = 16 at week 12, n = 6 at week 24 and n = 6 at week 36; light violet bar: Reference Group, n = 6 at week 12, n = 3 at week 24. Total Subjects: CD38+HLA-DR- at week 24, p = 0.0048; HLA-DR+CD38- at week 12, p = 0.0014 and at week 36, p = 0.0114; HLA-DR+CD38+ at week 12, p = 0.0139. Reference Group: CD38+ HLA-DR- at week 24, p = 0.0239; HLA-DR+CD38- at week 12, p = 0.0060.
(2.08 MB TIF)

Figure S3 Production of β2-microglobulin, neopterin and total Ig in subjects of the Reference Group of ISS OBS T-002. Changes from baseline of (A) β2-microglobulin serum levels (mg/L), (B) Neopterin (nmol/L), Total (C) IgM, (D) IgG and (E) IgA serum levels (mg/dL), respectively. Data are presented as the mean changes (± standard error) at 12, 24 and 36 weeks (n = 30 at week 12; n = 19 at week 24; n = 10 at week 36).
(1.62 MB TIF)

Figure S4 CD25 and FOXP3 expression on CD4+ T cells in Total Subjects of ISS OBS T-002. (A) Changes from baseline of CD4+ lymphocytes expressing CD25 are shown for Total Subjects (n = 34 at week 12; n = 10 at week 24; n = 8 at week 36 and n = 8 at week 48). (B) Changes from baseline of the percentage of CD4+CD25+ lymphocytes expressing FOXP3+ in Total Subjects (n = 31 at w12; n = 10 at week 24; n = 8 at week 36 and n = 8 at week 48). (C) Changes from baseline of the percentage of CD4+ T cells expressing CD25+FOXP3+ in Total Subjects (n = 31 at week12; n = 10 at week 24; n = 8 at week 36 and n = 8 at week 48). (D) Changes from baseline of the absolute number of CD4+ lymphocytes expressing CD25+FOXP3+ in Total Subjects (n = 25 at week 12; n = 6 at week 24; n = 2 at week 36 and n = 7 at week 48). Data are presented as the mean changes (± standard error). The t-Test for paired data was used for the analyses: *p<0.05, **p<0.01. CD4+CD25+ lymphocytes expressing FOXP3+ at week 36, p = 0.0220; at week 48, p = 0.0017. CD4+/CD25+/FOXP3+ T-reg number at week 36, p = 0.0467.
(1.72 MB TIF)

Figure S5 CD25 and FOXP3 expression on CD4+ T cells in subjects of the Reference Group of ISS OBS T-002 study. (A) Changes from baseline of CD4+ lymphocytes expressing CD25 are shown in subjects of the Reference Group (n = 20 at week 12; n = 8 at week 24; n = 4 at week 36 and n = 4 at week 48). (B) Changes from baseline of the percentage of CD4+CD25+ lymphocytes expressing FOXP3+ in subjects of the Reference Group (n = 19 at week 12; n = 8 at week 24; n = 4 at week 36 and n = 4 at week 48). (C) Changes from baseline of the percentage of CD4+ T cells expressing CD25+FOXP3+ in subjects of the Reference Group (n = 19 at week 12; n = 8 at week 24; n = 4 at week 36 and n = 4 at week 48). (D) Changes from baseline of the absolute number of CD4+ lymphocytes expressing CD25+FOXP3+ in subjects of the Reference Group (n = 15 at week 12; n = 5 at week 24 and n = 4 at week 48). Data are presented as the mean changes (± standard error). The t-Test for paired data was used for the analyses: *p<0.05. CD4+CD25+ lymphocytes expressing FOXP3+ at week 48, p = 0.0290.
(1.81 MB TIF)

Figure S6 Evaluation of PBMC viability, CD4+ T cell and B cell counts in subjects of ISS OBS. Changes from baseline of in vitro PBMC viability in Total Subjects (A) and the Reference Group (B); n = 88 at week 12; n = 62 at week 24; n = 46 at week 36 and n = 30 at week 48 for the Total Subjects; n = 32 at week 12; n = 20 at week 24; n = 11 at week 36 and n = 6 at week 48 for the Reference Group. The t-Test for paired data was used for the analyses: **p<0.01. Total Subjects: at 36 and 48 weeks, p<0.0001; Reference Group: at week 36, p = 0.0003. Changes from baseline of CD4+ T cells/µl (data from clinical sites) for Total Subjects (C) and the Reference Group subjects (D); n = 76 at week 12; n = 54 at week 24; n = 37 at week 36 and n = 25 at week 48 for the Total Subjects; n = 29 at week 12; n = 19 at week 24; n = 10 at week 36 and n = 5 at week 48 for subjects of the Reference Group. Changes from baseline of B cells/µL, for Total Subjects (E) and the Reference Group subjects (F), n = 73 at week 12; n = 20 at week 24; n = 10 at week 36 for the Total Subjects; n = 27 at week 12; n = 8 at week 24; n = 3 at week 36 for subjects of the Reference Group.
(1.99 MB TIF)

Figure S7 Evaluation of the percentage of CD4+, CD8+, NK and B cells in subjects of ISS OBS T-002 prior or after stratification by HAART regimen. Changes from baseline of CD4+, CD8+, NK and B cells (percentage) for Total Subjects (A) and Reference Group subjects (B); n = 73 at week 12; n = 20 at week 24; n = 10 at week 36 for Total Subjects; n = 27 at week 12; n = 8 at week 24; n = 3 at week 36 for subjects of the Reference Group. Changes from baseline of CD4+, CD8+, NK and B cells (percentage) for NNRTI-based (C, D) in Total Subjects (C) and in the Reference Group subjects (D), respectively, and for PI-based (E) in Total Subjects. NNRTI-based: n = 43 at week 12, n = 10 at week 24, n = 6 at week 36 for Total Subjects, and n = 16 at week 12, n = 4 at week 24, n = 3 at week 36 for the Reference Group. PI-based: n = 25 at week 12, n = 6 at week 24, n = 3 at week 36 for Total Subjects. The t-Test for paired data was used for the analyses: *p<0.05. Total Subjects, PI-based: CD8+ T cells at week 24, p = 0.0339; B cells at week 36, p = 0.0291.
(1.94 MB TIF)

Figure S8 Characterization of naïve, central and effector memory CD4+ and CD8+ T cells in Total Subjects and in the Reference Group of ISS OBS T-002. Percentage of naïve (CD45RA+/CD62L+), effector RA+ (CD45RA+/CD62L-, Temra) or RA- (CD45RA-/CD62L-, Temro) and central memory (CD45RA-/CD62L+, Tcm) CD4+ (A) or CD8+ (B) T cells for total OBS subjects at baseline and at week 12 and 24 (n = 6 at week 0; n = 6 at week 12, n = 2 at week 24), and for CD4+ (C) or CD8+ (D) T cells for the Reference Group at baseline and at week 12 and 24 (n = 5 at week 0; n = 5 at week 12, n = 2 at week 24).
(2.36 MB TIF)

Figure S9 Cellular immune responses against Tat, Env or recall antigens in Total Subjects of ISS OBS T-002. Percentage of responders at baseline (green bar) and up to week 48 (orange bar). (A) Percentage of subjects (n = 87) showing anti-Tat production of IFN-γ, IL-2 and IL-4, and (B) percentage of subjects (n = 67) showing anti-Tat CD4+ or CD8+ lymphoproliferative responses. (C) Percentage of subjects (n = 72) showing anti-Env production of IFN-γ, IL-2 and IL-4, and (D) percentage of subjects (n = 64) showing anti-Env CD4+ or CD8+ lymphoproliferative responses. (E) Percentage of subjects (n = 74) showing anti-Candida cytokines production, and (F) percentage of subjects (n = 64) showing anti-Candida CD4+ or CD8+ lymphoproliferative responses. (G) Percentage of subjects (n = 78) showing anti-CEF production of IFN-γ, IL-2 and IL-4, and (H) percentage of subjects (n = 64) showing anti-CEF CD4+ or CD8+ lymphoproliferative responses. The McNemar's test was used for the analyses: *p<0.05, **p<0.01. Anti-Tat response: IFN-γ, p = 0.0270; IL-2, p = 0.0411; CD8+ proliferation, p = 0.0017. Anti-Env response: CD4+ proliferation, p = 0.0184; CD8+ proliferation, p = 0.0007. Anti-Candida response: IL-4, p = 0.0003; CD4+ proliferation, p = 0.0158; CD8+ proliferation, p = 0.0411. Anti-CEF response: IFN-γ,p = 0.0016; IL-2, p = 0.0008; IL-4, p<0.0001.
(2.71 MB TIF)

Figure S10 Cellular immune responses against Tat, Env or recall antigens in the Reference Group of ISS OBS T-002. Percentage of responders at baseline (green bar) and up to week 48 (orange bar). (A) Percentage of subjects (n = 31) showing IFN-γ, IL-2 and IL-4 production against Tat, and (B) percentage of subjects (n = 26) showing anti-Tat CD4+ or CD8+ lymphoproliferative responses. (C) Percentage of subjects (n = 29) showing anti-Env production of IFN-γ, IL-2 and IL-4, and (D) percentage of subjects (n = 23) showing anti-Env CD4+ or CD8+ lymphoproliferative responses. (E) Percentage of subjects (n = 29) showing anti-Candida production of IFN-γ, IL-2 and IL-4, and (F) percentage of subjects (n = 23) showing anti-Candida CD4+ or CD8+ lymphoproliferative responses. (G) Percentage of subjects (n = 28) showing anti-CEF production of IFN-γ, IL-2 and IL-4, and (H) percentage of subjects (n = 23) showing anti-CEF CD4+ or CD8+ lymphoproliferative responses. The McNemar's test was used for the analyses: *p<0.05, **p<0.01. Anti-Tat response: IL-4, p = 0.0339; CD8+ proliferation, p = 0.0348. Anti-CEF response: IFN-γ, p = 0.0253; IL-4, p = 0.0339.
(1.37 MB TIF)

Table S1 Baseline characteristics of study participants in ISS OBS T-002 for the Total Subjects and the Reference Group.
(0.04 MB DOC)

Table S2 Immune activation markers and T-regs at baseline in subjects of ISS OBS T-002.
(0.04 MB DOC)

Table S3 Tat-specific cellular immune responses in subjects of ISS OBS T-002.
(0.04 MB DOC)

Table S4 Cellular immune responses against Env in subjects of ISS OBS T-002.
(0.04 MB DOC)

Table S5 Cellular immune responses against Candida in subjects of ISS OBS T-002.
(0.04 MB DOC)

Table S6 Cellular immune responses against CEF in subjects of ISS OBS T-002.
(0.04 MB DOC)

Protocol S1 Trial Protocol.
(0.89 MB PDF)

Protocol S2 ISS OBS T-002 Protocol.
(0.15 MB PDF)

Checklist S1 CONSORT Checklist.
(0.23 MB DOC)
