# Supplementary figures and images for: Therapeutic Immunization with HIV-1 Tat Reduces Immune Activation and Loss of Regulatory T-Cells and Improves Immune Function in Subjects on HAART
Source: PLoS One. 2010 Nov 11;5(11):e13540. doi: 10.1371/journal.pone.0013540 (PMC2978690; doi:10.1371/journal.pone.0013540)

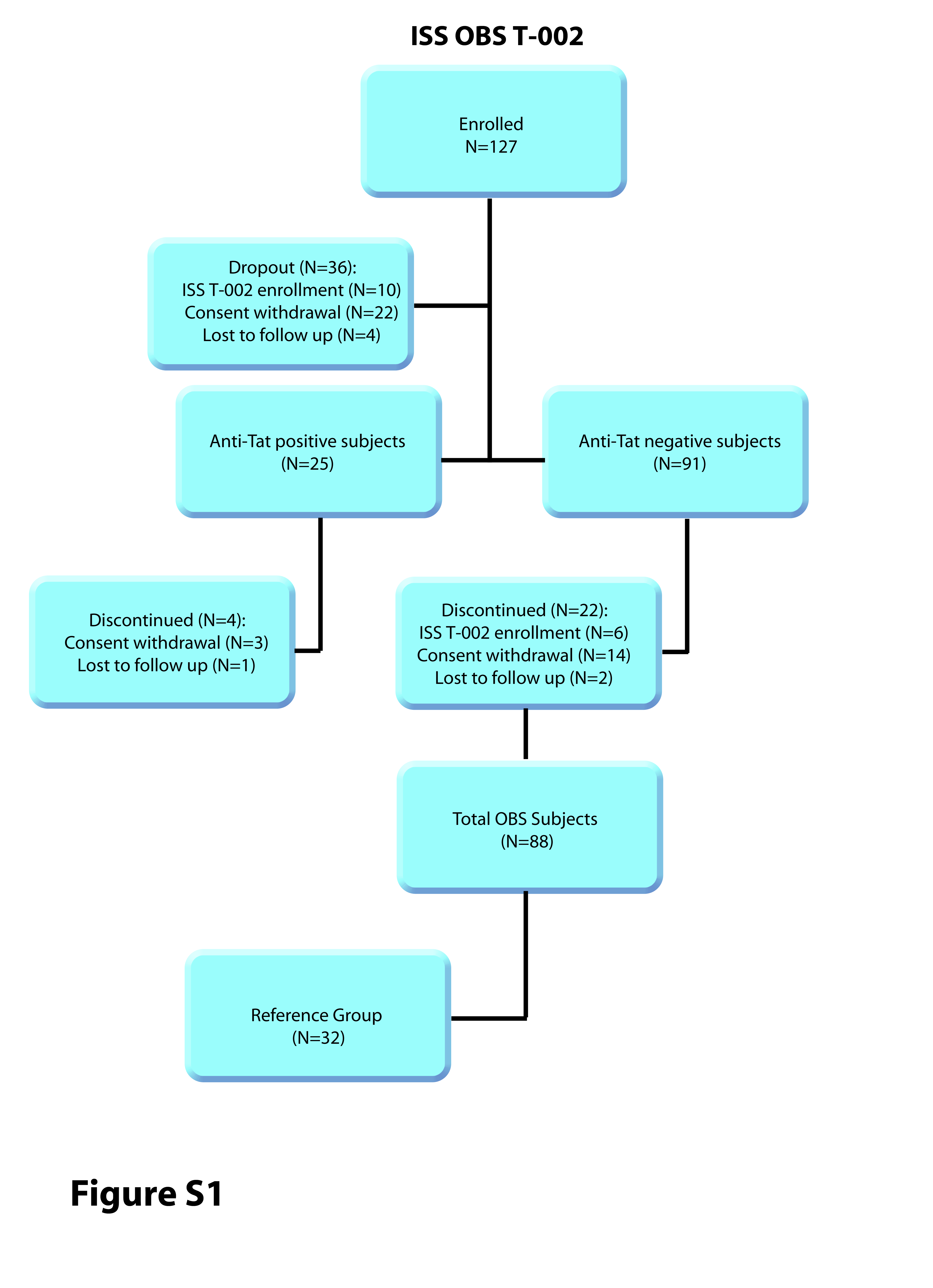

Supplement: Figure S1 — Flow diagram of ISS OBS T-002 study participants. One hundred and twenty-seven subjects were recruited in the observational study ISS OBS T-002. Among them, 25 individuals were anti-Tat Ab positive and 91 anti-Tat Ab negative, respectively. Evaluable subjects were constituted by 88 anti-Tat Ab negative (Total OBS Subjects) and 32 Reference Group subjects, respectively. The Total OBS Subjects included anti-Tat Ab negative volunteers of either gender, ≥18 years old, under successful HAART (chronic suppression of HIV infection with a plasma viremia <50 copies/ml in the last 6 months and without a history of virologic rebound), a known nadir level of CD4+ T cells and CD4+ T cell number at study entry. The Reference Group included subjects having at baseline the same characteristics of volunteers enrolled in the ISS T-002 clinical trial: anti-Tat Ab negative (18–55 years of age), HAART-treated with chronic suppressed HIV infection, with levels of plasma viremia <50 copies/ml in the last 6 months prior to the screening and without a history of virologic rebound, CD4+ T cell counts ≥400 cells/µL and pre-HAART CD4 nadir >250 cells/µL. (2.39 MB TIF) [file pone.0013540.s001.tif]

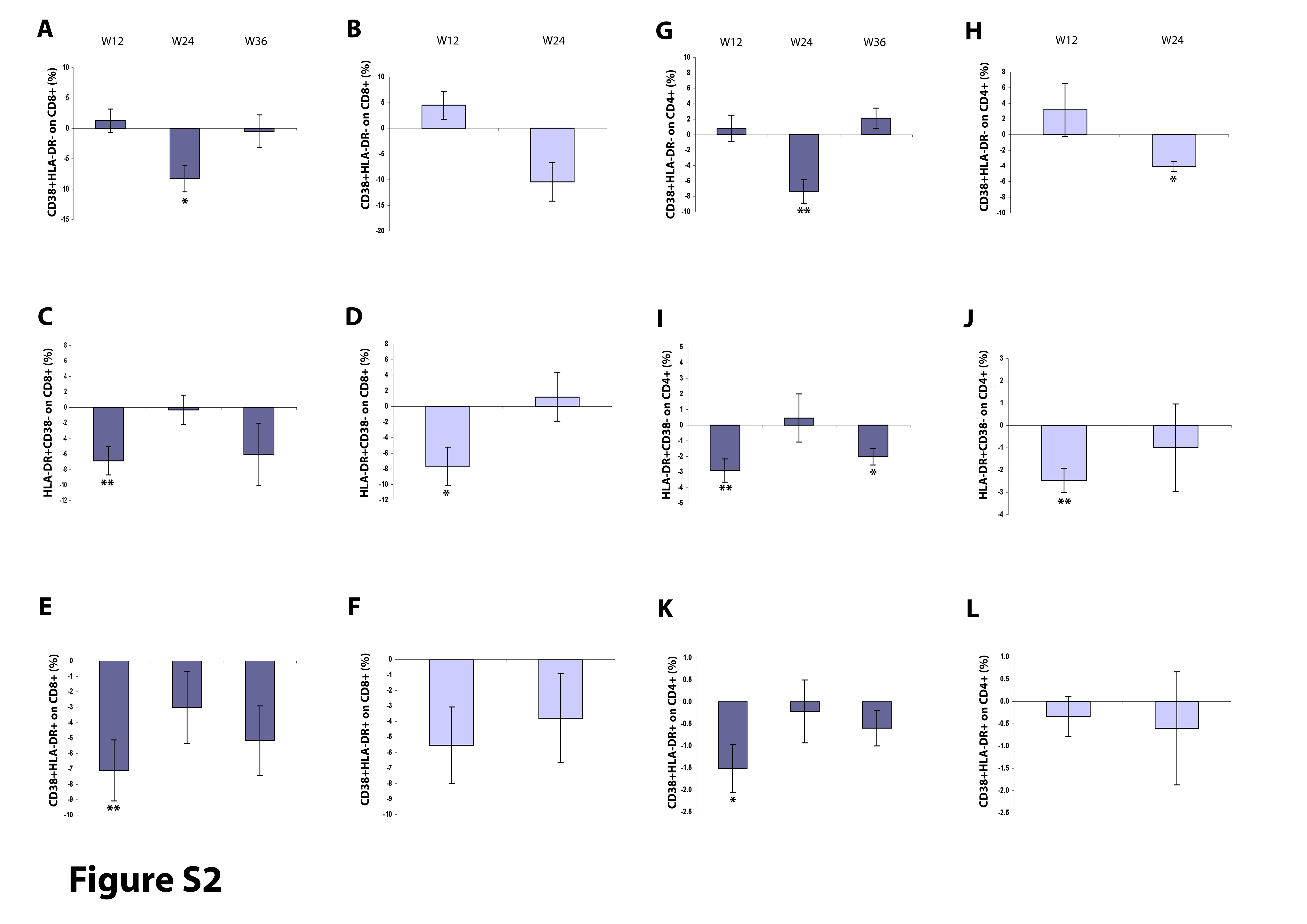

Supplement: Figure S2 — Expression of activation markers on CD8+ and CD4+ T cells in subjects of ISS OBS T-002. Changes from baseline of CD8+ T cells (gating on CD8+ cells) expressing (A, B) CD38, (C, D) HLA-DR, or (E, F) both CD38 and HLA-DR in the Total Subjects (A, C, E) and in the Reference Group (B, D, F), respectively. Data are presented as the mean % changes (±standard error) at week 12, 24 and 36. Blue bars: Total Subjects n = 16 at week 12, n = 6 at week 24 and n = 6 at week 36; light violet bar: Reference Group, n = 6 at week 12, n = 3 at week 24. The t-Test for paired data was used for the analyses: *p<0.05, **p<0.01. Total Subjects: CD38+HLA-DR- at week 24, p = 0.0121; HLA-DR+CD38- at week 12, p = 0.0019; HLA-DR+CD38+ at week 12, p = 0.0019. Reference Group: HLA-DR+CD38- at week 12, p = 0.0256. Changes from baseline of CD4+ T cells (gating on CD4+ cells) expressing (G, H) CD38, (I, J) HLA-DR, or (K, L) both CD38 and HLA-DR in the Total Subjects (G, I, K) and in Reference Group (H, J, L), respectively. Data are presented as the mean % changes (±standard error) at week 12, 24 and 36. Blue bars: Total Subjects n = 16 at week 12, n = 6 at week 24 and n = 6 at week 36; light violet bar: Reference Group, n = 6 at week 12, n = 3 at week 24. Total Subjects: CD38+HLA-DR- at week 24, p = 0.0048; HLA-DR+CD38- at week 12, p = 0.0014 and at week 36, p = 0.0114; HLA-DR+CD38+ at week 12, p = 0.0139. Reference Group: CD38+ HLA-DR- at week 24, p = 0.0239; HLA-DR+CD38- at week 12, p = 0.0060. (2.08 MB TIF) [file pone.0013540.s002.tif]

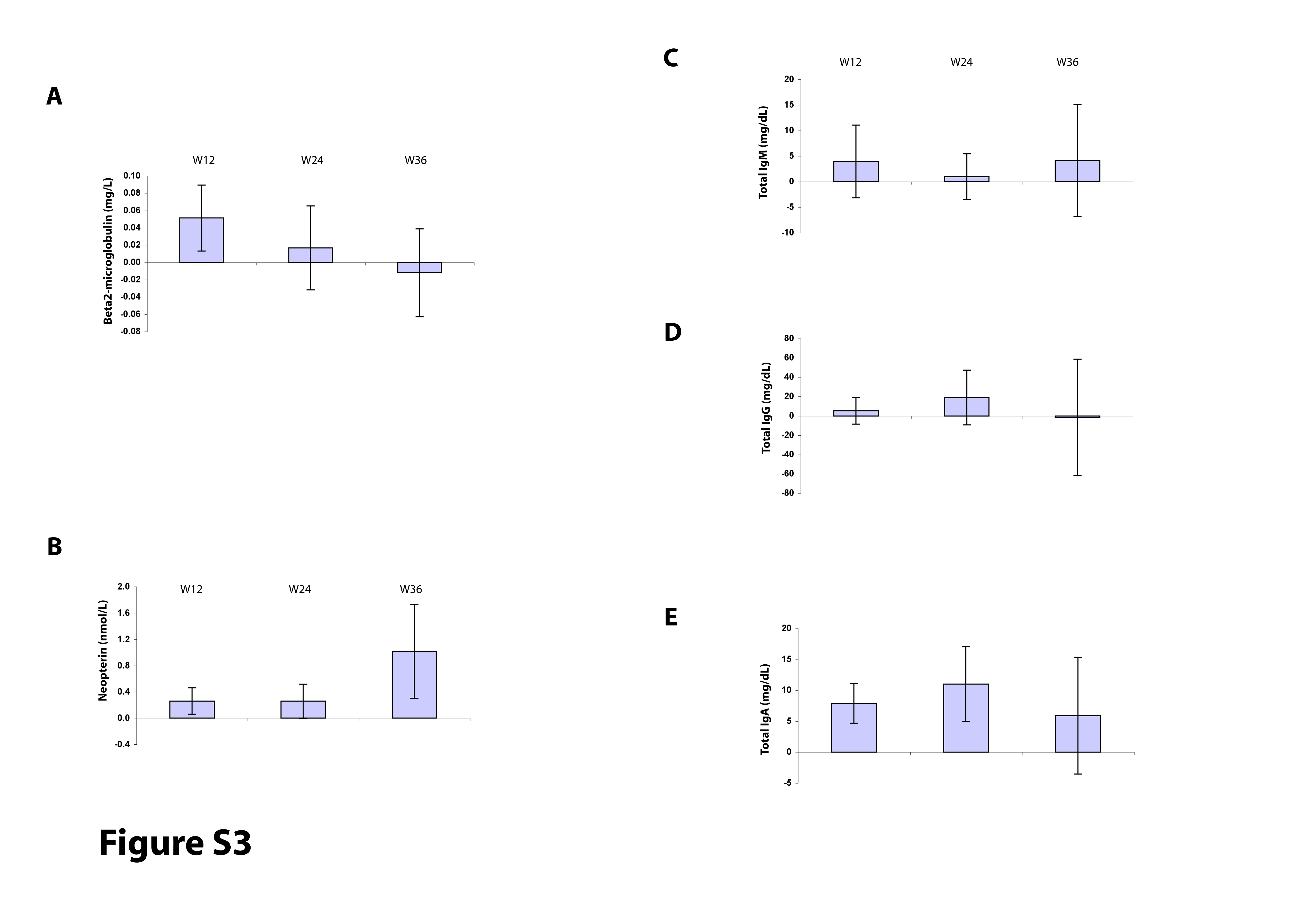

Supplement: Figure S3 — Production of β2-microglobulin, neopterin and total Ig in subjects of the Reference Group of ISS OBS T-002. Changes from baseline of (A) β2-microglobulin serum levels (mg/L), (B) Neopterin (nmol/L), Total (C) IgM, (D) IgG and (E) IgA serum levels (mg/dL), respectively. Data are presented as the mean changes (± standard error) at 12, 24 and 36 weeks (n = 30 at week 12; n = 19 at week 24; n = 10 at week 36). (1.62 MB TIF) [file pone.0013540.s003.tif]

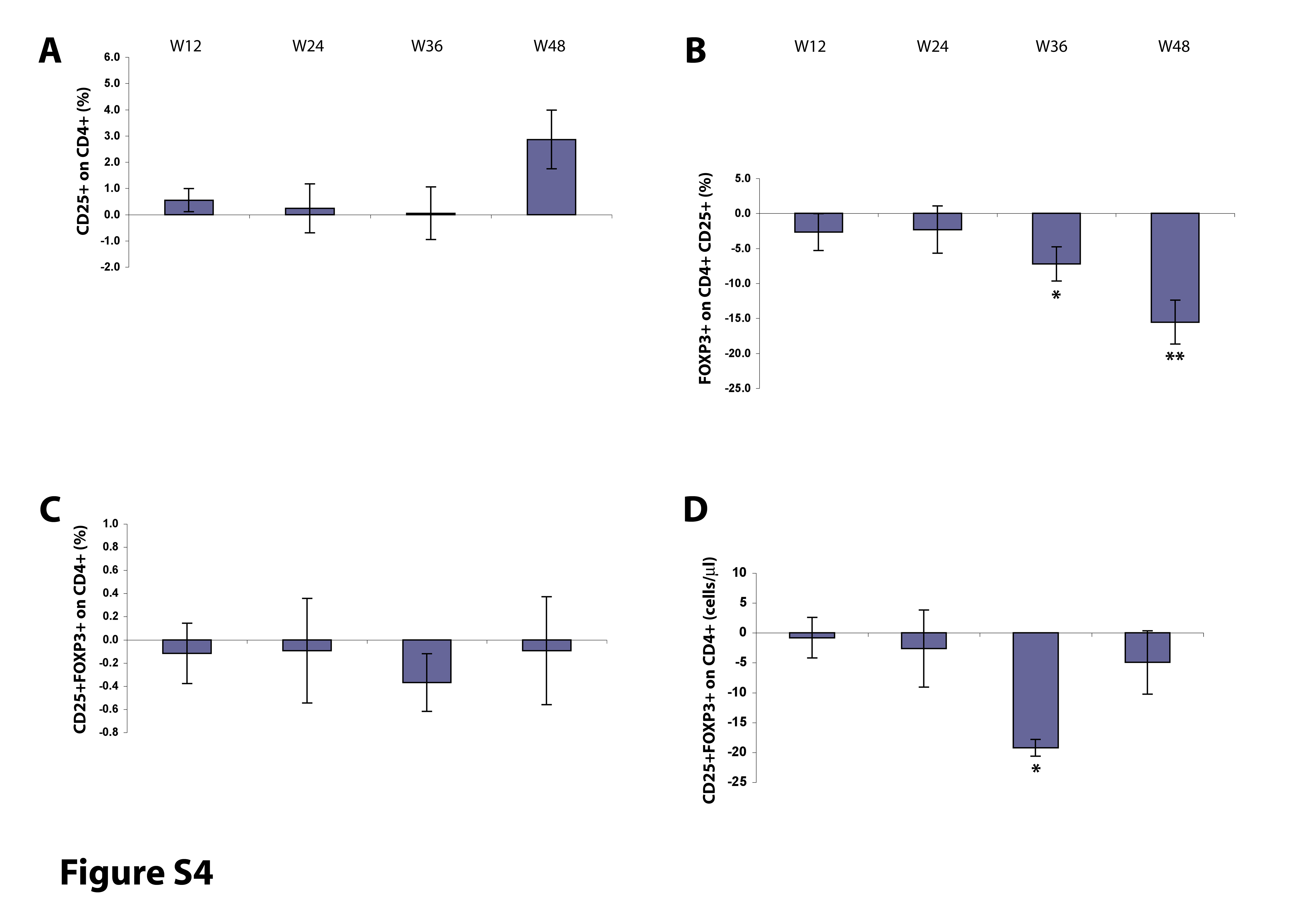

Supplement: Figure S4 — CD25 and FOXP3 expression on CD4+ T cells in Total Subjects of ISS OBS T-002. (A) Changes from baseline of CD4+ lymphocytes expressing CD25 are shown for Total Subjects (n = 34 at week 12; n = 10 at week 24; n = 8 at week 36 and n = 8 at week 48). (B) Changes from baseline of the percentage of CD4+CD25+ lymphocytes expressing FOXP3+ in Total Subjects (n = 31 at w12; n = 10 at week 24; n = 8 at week 36 and n = 8 at week 48). (C) Changes from baseline of the percentage of CD4+ T cells expressing CD25+FOXP3+ in Total Subjects (n = 31 at week12; n = 10 at week 24; n = 8 at week 36 and n = 8 at week 48). (D) Changes from baseline of the absolute number of CD4+ lymphocytes expressing CD25+FOXP3+ in Total Subjects (n = 25 at week 12; n = 6 at week 24; n = 2 at week 36 and n = 7 at week 48). Data are presented as the mean changes (± standard error). The t-Test for paired data was used for the analyses: *p<0.05, **p<0.01. CD4+CD25+ lymphocytes expressing FOXP3+ at week 36, p = 0.0220; at week 48, p = 0.0017. CD4+/CD25+/FOXP3+ T-reg number at week 36, p = 0.0467. (1.72 MB TIF) [file pone.0013540.s004.tif]

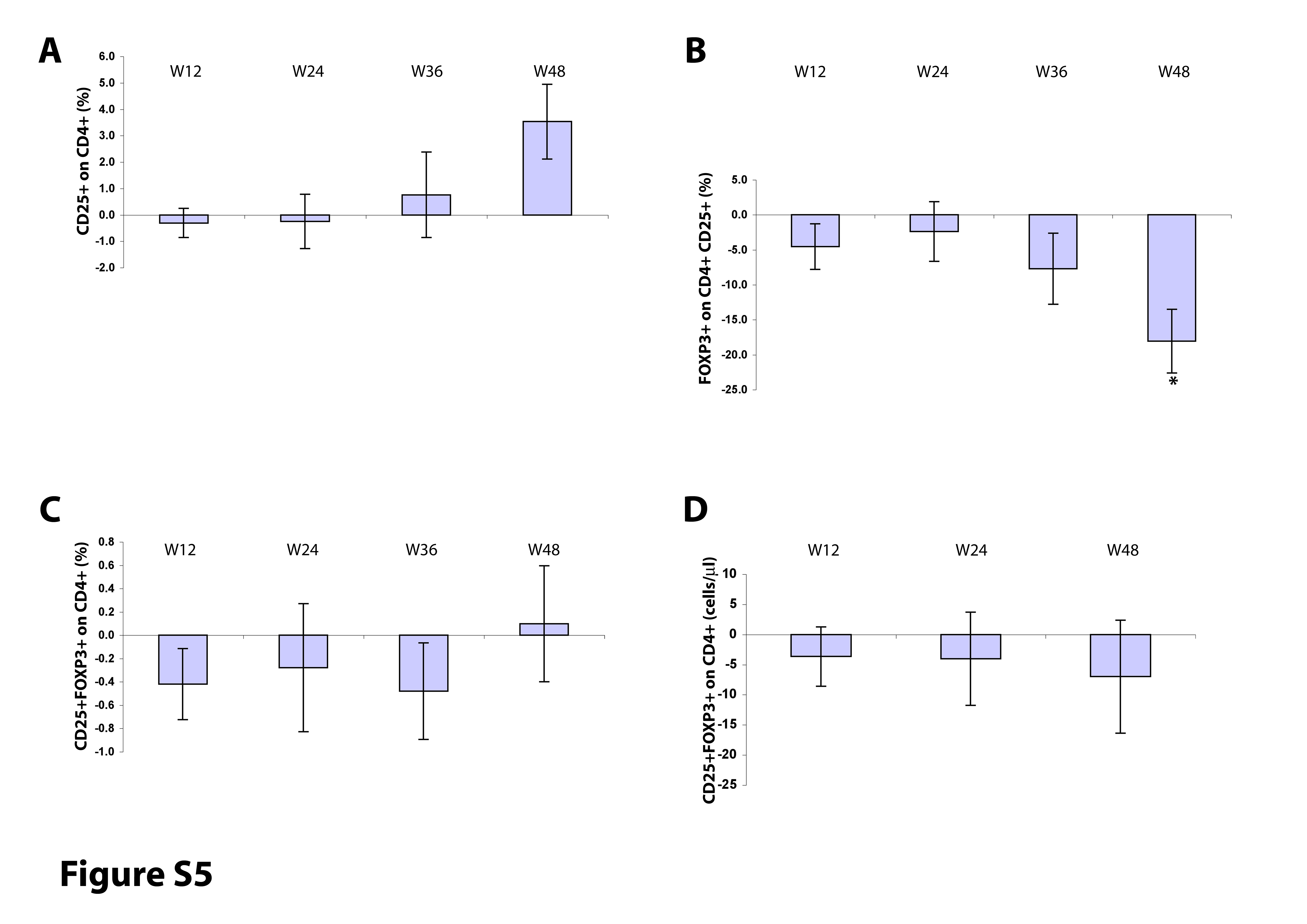

Supplement: Figure S5 — CD25 and FOXP3 expression on CD4+ T cells in subjects of the Reference Group of ISS OBS T-002 study. (A) Changes from baseline of CD4+ lymphocytes expressing CD25 are shown in subjects of the Reference Group (n = 20 at week 12; n = 8 at week 24; n = 4 at week 36 and n = 4 at week 48). (B) Changes from baseline of the percentage of CD4+CD25+ lymphocytes expressing FOXP3+ in subjects of the Reference Group (n = 19 at week 12; n = 8 at week 24; n = 4 at week 36 and n = 4 at week 48). (C) Changes from baseline of the percentage of CD4+ T cells expressing CD25+FOXP3+ in subjects of the Reference Group (n = 19 at week 12; n = 8 at week 24; n = 4 at week 36 and n = 4 at week 48). (D) Changes from baseline of the absolute number of CD4+ lymphocytes expressing CD25+FOXP3+ in subjects of the Reference Group (n = 15 at week 12; n = 5 at week 24 and n = 4 at week 48). Data are presented as the mean changes (± standard error). The t-Test for paired data was used for the analyses: *p<0.05. CD4+CD25+ lymphocytes expressing FOXP3+ at week 48, p = 0.0290. (1.81 MB TIF) [file pone.0013540.s005.tif]

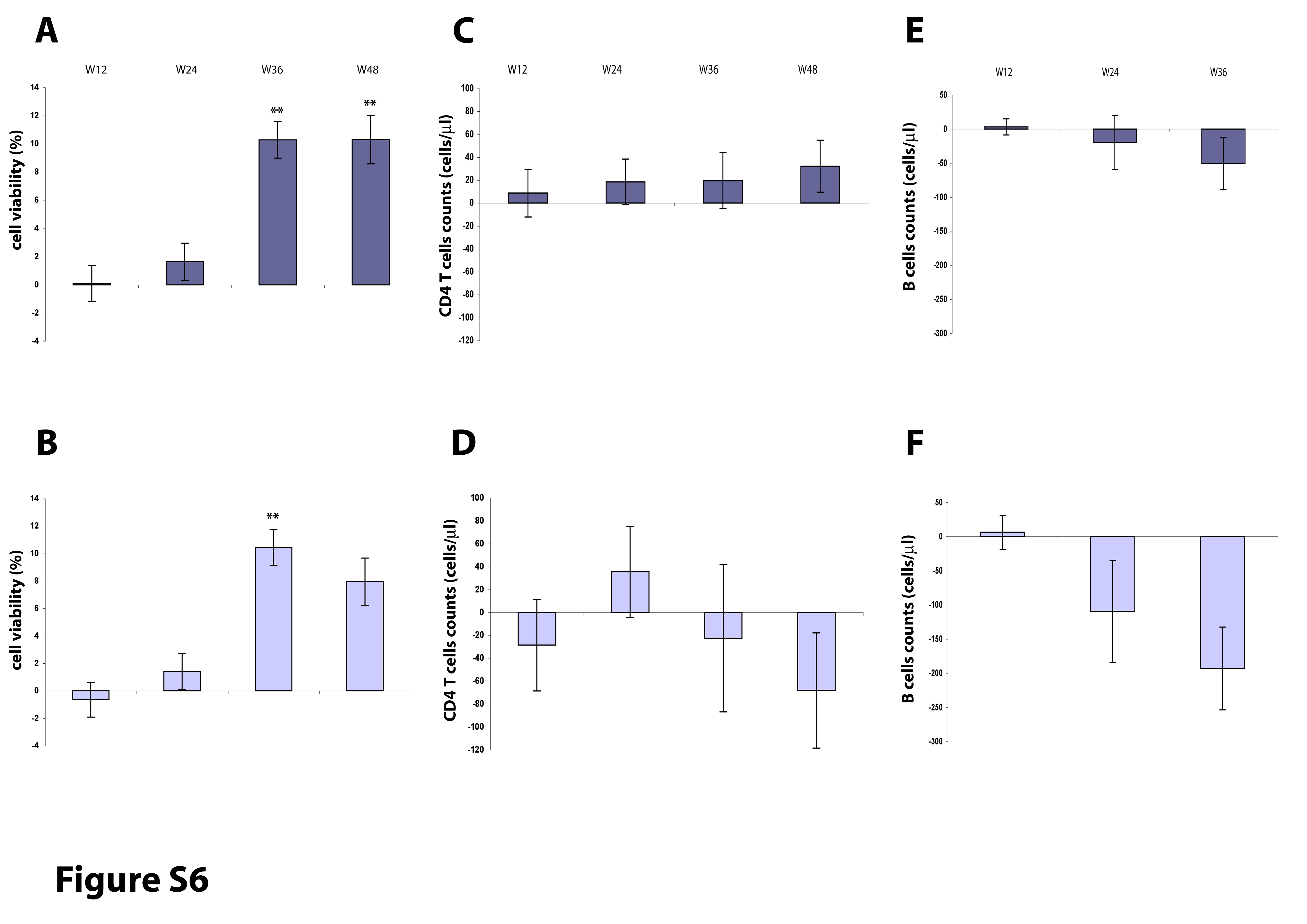

Supplement: Figure S6 — Evaluation of PBMC viability, CD4+ T cell and B cell counts in subjects of ISS OBS. Changes from baseline of in vitro PBMC viability in Total Subjects (A) and the Reference Group (B); n = 88 at week 12; n = 62 at week 24; n = 46 at week 36 and n = 30 at week 48 for the Total Subjects; n = 32 at week 12; n = 20 at week 24; n = 11 at week 36 and n = 6 at week 48 for the Reference Group. The t-Test for paired data was used for the analyses: **p<0.01. Total Subjects: at 36 and 48 weeks, p<0.0001; Reference Group: at week 36, p = 0.0003. Changes from baseline of CD4+ T cells/µl (data from clinical sites) for Total Subjects (C) and the Reference Group subjects (D); n = 76 at week 12; n = 54 at week 24; n = 37 at week 36 and n = 25 at week 48 for the Total Subjects; n = 29 at week 12; n = 19 at week 24; n = 10 at week 36 and n = 5 at week 48 for subjects of the Reference Group. Changes from baseline of B cells/µL, for Total Subjects (E) and the Reference Group subjects (F), n = 73 at week 12; n = 20 at week 24; n = 10 at week 36 for the Total Subjects; n = 27 at week 12; n = 8 at week 24; n = 3 at week 36 for subjects of the Reference Group. (1.99 MB TIF) [file pone.0013540.s006.tif]

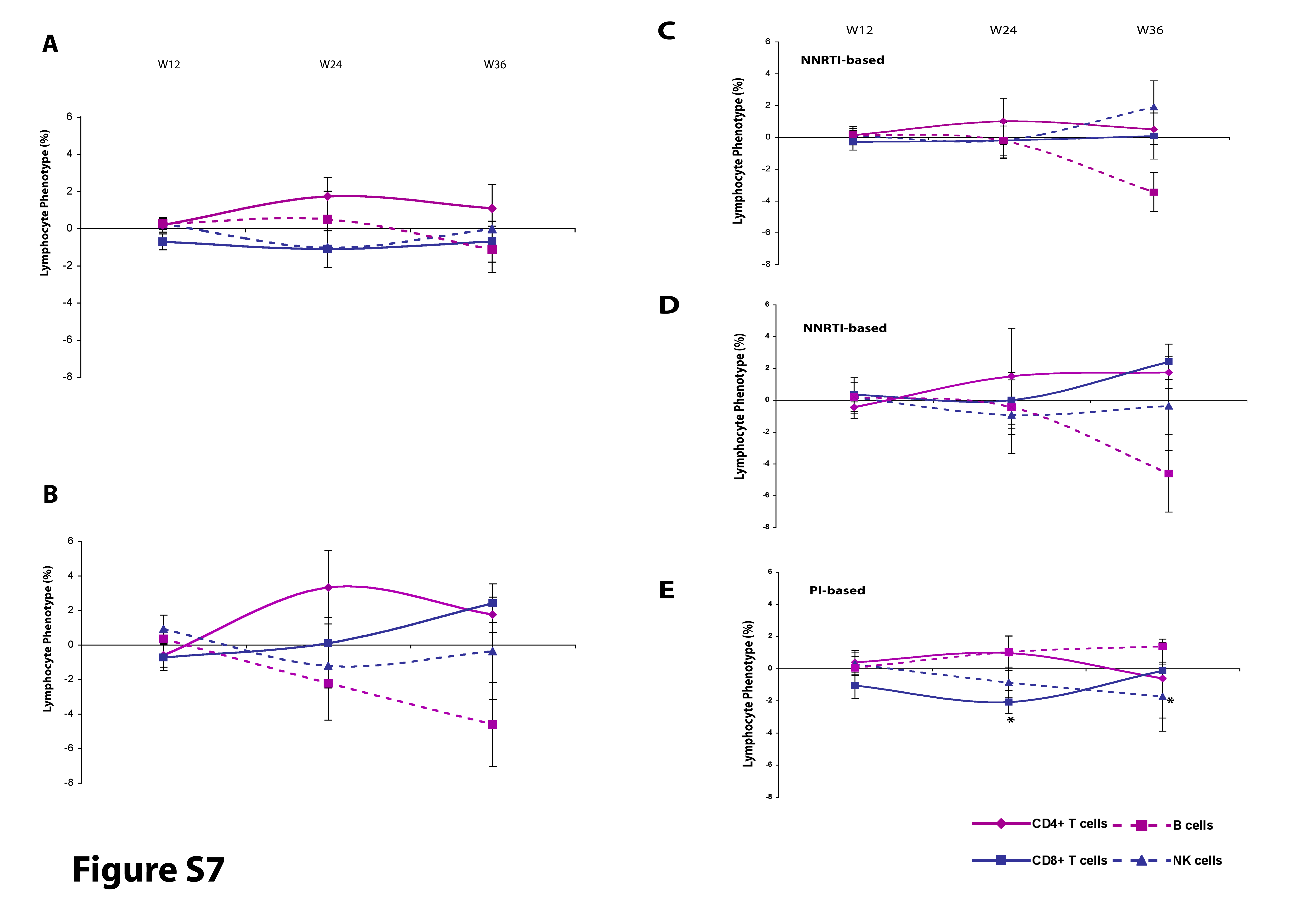

Supplement: Figure S7 — Evaluation of the percentage of CD4+, CD8+, NK and B cells in subjects of ISS OBS T-002 prior or after stratification by HAART regimen. Changes from baseline of CD4+, CD8+, NK and B cells (percentage) for Total Subjects (A) and Reference Group subjects (B); n = 73 at week 12; n = 20 at week 24; n = 10 at week 36 for Total Subjects; n = 27 at week 12; n = 8 at week 24; n = 3 at week 36 for subjects of the Reference Group. Changes from baseline of CD4+, CD8+, NK and B cells (percentage) for NNRTI-based (C, D) in Total Subjects (C) and in the Reference Group subjects (D), respectively, and for PI-based (E) in Total Subjects. NNRTI-based: n = 43 at week 12, n = 10 at week 24, n = 6 at week 36 for Total Subjects, and n = 16 at week 12, n = 4 at week 24, n = 3 at week 36 for the Reference Group. PI-based: n = 25 at week 12, n = 6 at week 24, n = 3 at week 36 for Total Subjects. The t-Test for paired data was used for the analyses: *p<0.05. Total Subjects, PI-based: CD8+ T cells at week 24, p = 0.0339; B cells at week 36, p = 0.0291. (1.94 MB TIF) [file pone.0013540.s007.tif]

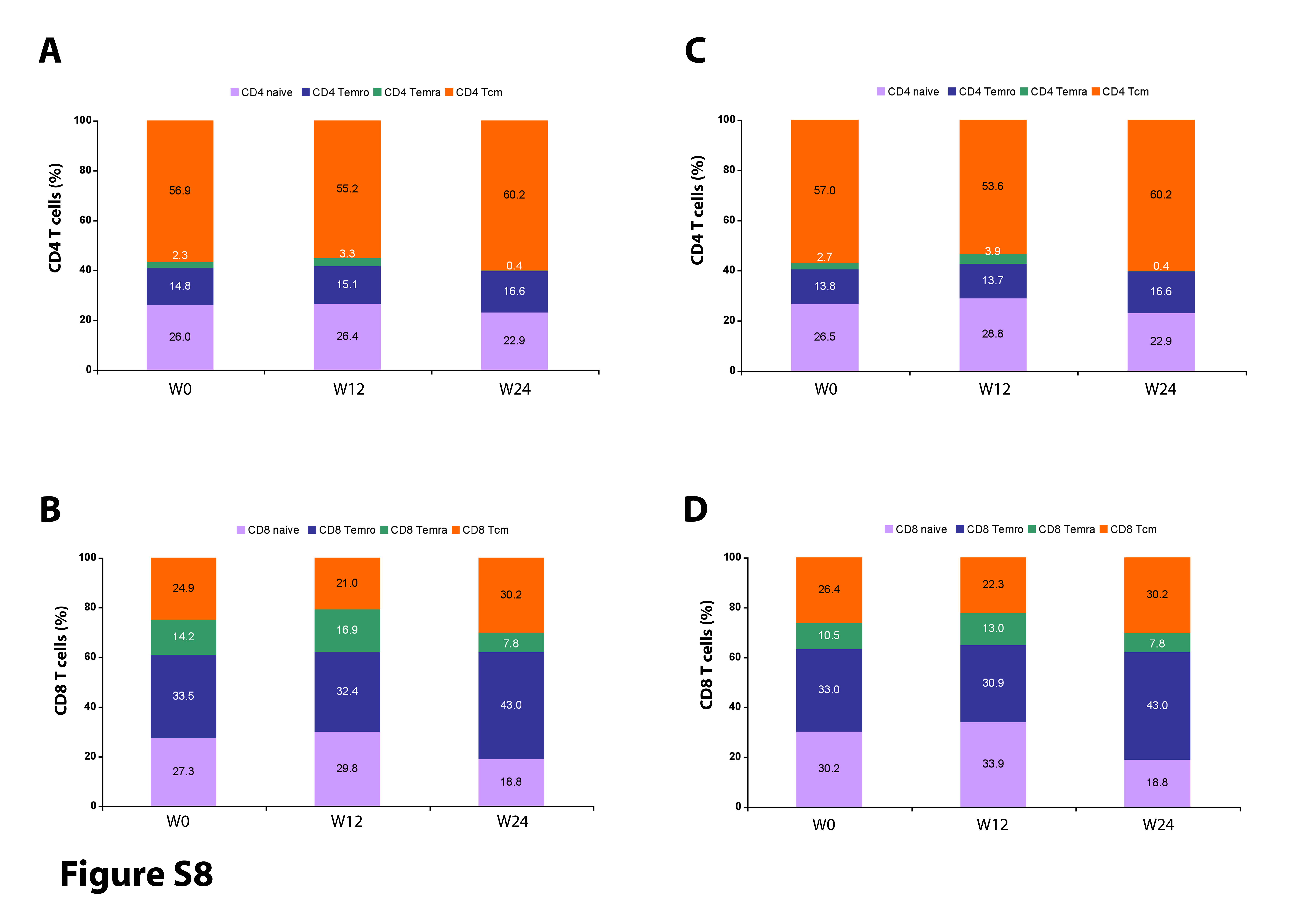

Supplement: Figure S8 — Characterization of naïve, central and effector memory CD4+ and CD8+ T cells in Total Subjects and in the Reference Group of ISS OBS T-002. Percentage of naïve (CD45RA+/CD62L+), effector RA+ (CD45RA+/CD62L-, Temra) or RA- (CD45RA-/CD62L-, Temro) and central memory (CD45RA-/CD62L+, Tcm) CD4+ (A) or CD8+ (B) T cells for total OBS subjects at baseline and at week 12 and 24 (n = 6 at week 0; n = 6 at week 12, n = 2 at week 24), and for CD4+ (C) or CD8+ (D) T cells for the Reference Group at baseline and at week 12 and 24 (n = 5 at week 0; n = 5 at week 12, n = 2 at week 24). (2.36 MB TIF) [file pone.0013540.s008.tif]

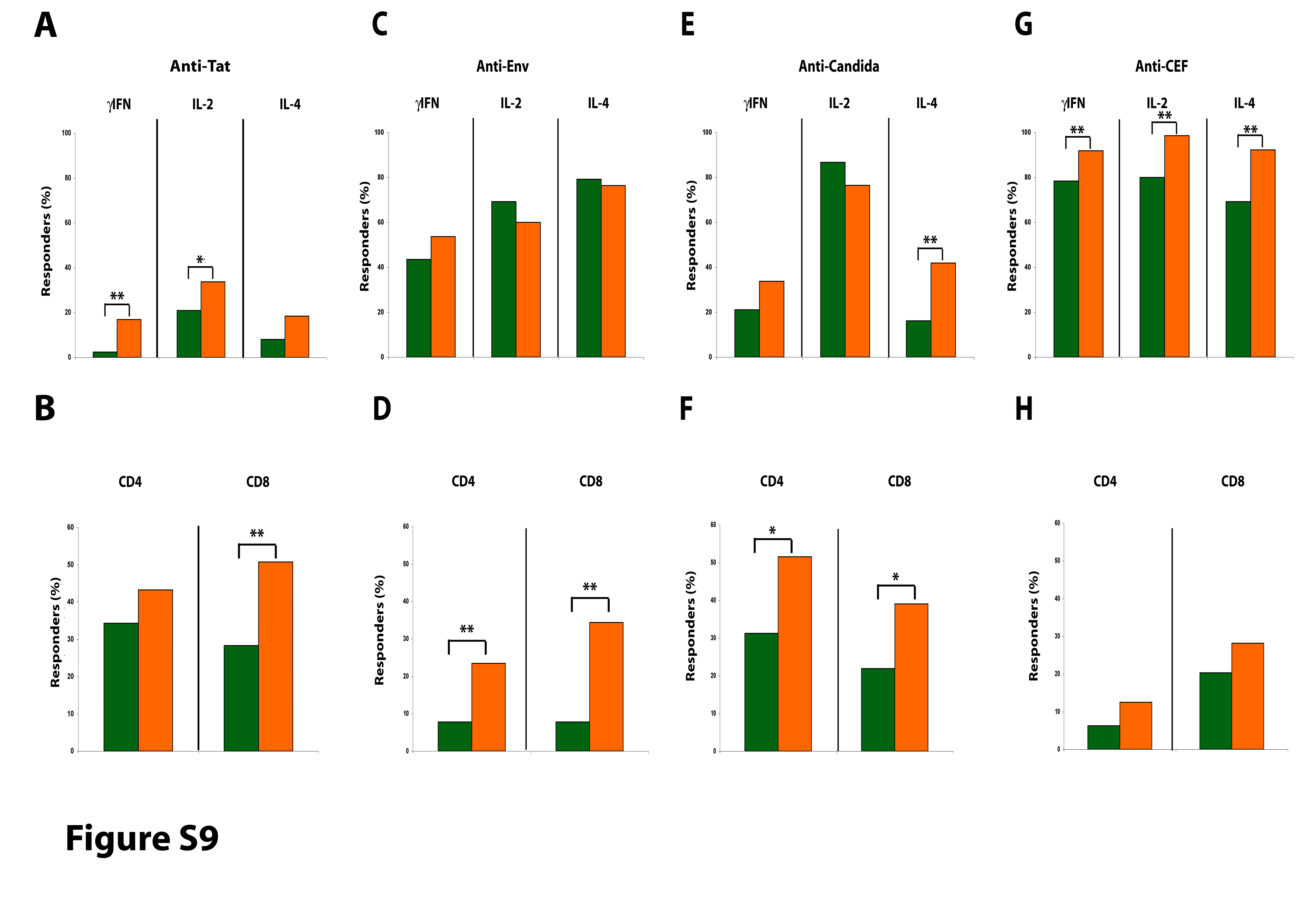

Supplement: Figure S9 — Cellular immune responses against Tat, Env or recall antigens in Total Subjects of ISS OBS T-002. Percentage of responders at baseline (green bar) and up to week 48 (orange bar). (A) Percentage of subjects (n = 87) showing anti-Tat production of IFN-γ, IL-2 and IL-4, and (B) percentage of subjects (n = 67) showing anti-Tat CD4+ or CD8+ lymphoproliferative responses. (C) Percentage of subjects (n = 72) showing anti-Env production of IFN-γ, IL-2 and IL-4, and (D) percentage of subjects (n = 64) showing anti-Env CD4+ or CD8+ lymphoproliferative responses. (E) Percentage of subjects (n = 74) showing anti-Candida cytokines production, and (F) percentage of subjects (n = 64) showing anti-Candida CD4+ or CD8+ lymphoproliferative responses. (G) Percentage of subjects (n = 78) showing anti-CEF production of IFN-γ, IL-2 and IL-4, and (H) percentage of subjects (n = 64) showing anti-CEF CD4+ or CD8+ lymphoproliferative responses. The McNemar's test was used for the analyses: *p<0.05, **p<0.01. Anti-Tat response: IFN-γ, p = 0.0270; IL-2, p = 0.0411; CD8+ proliferation, p = 0.0017. Anti-Env response: CD4+ proliferation, p = 0.0184; CD8+ proliferation, p = 0.0007. Anti-Candida response: IL-4, p = 0.0003; CD4+ proliferation, p = 0.0158; CD8+ proliferation, p = 0.0411. Anti-CEF response: IFN-γ,p = 0.0016; IL-2, p = 0.0008; IL-4, p<0.0001. (2.71 MB TIF) [file pone.0013540.s009.tif]

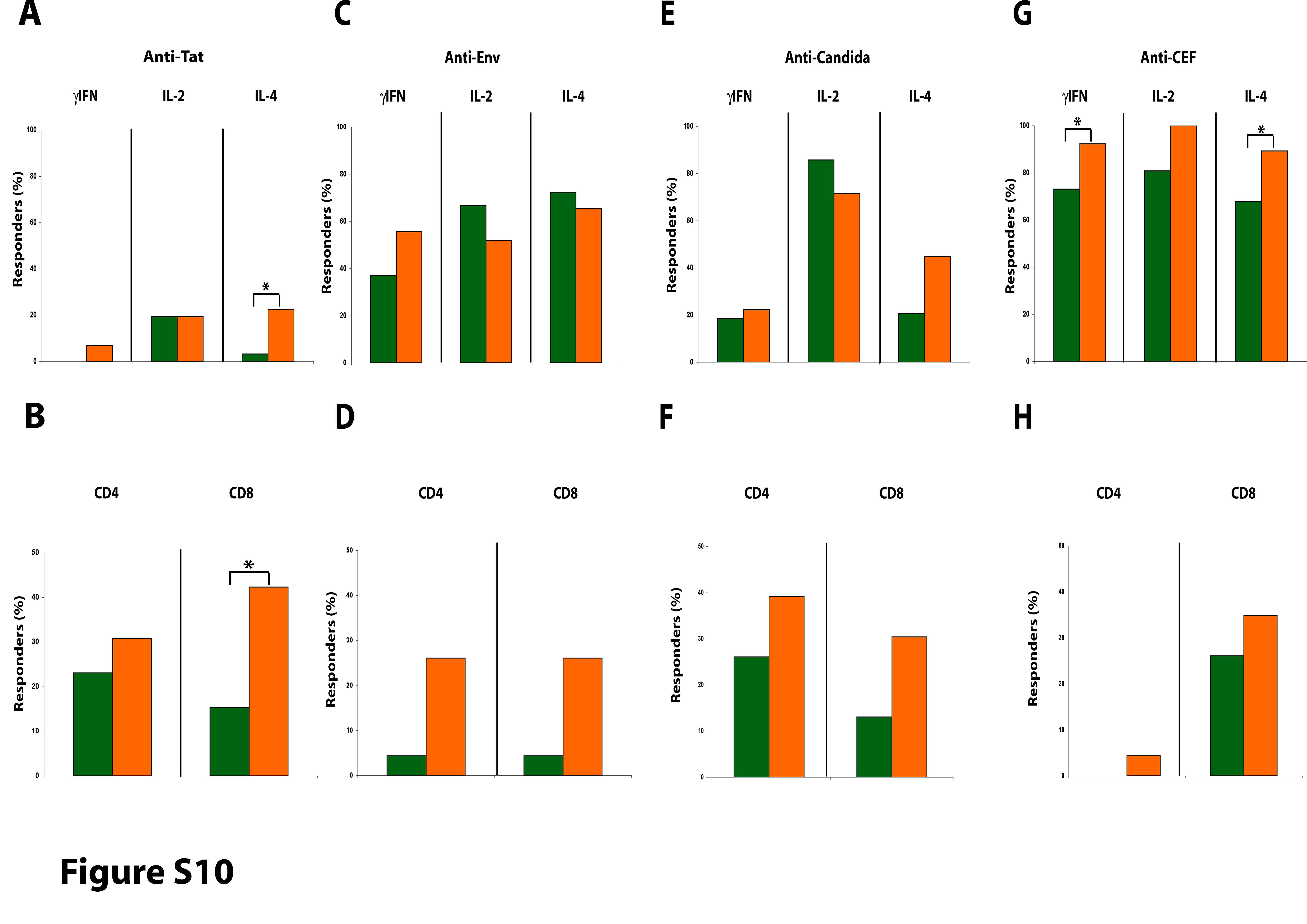

Supplement: Figure S10 — Cellular immune responses against Tat, Env or recall antigens in the Reference Group of ISS OBS T-002. Percentage of responders at baseline (green bar) and up to week 48 (orange bar). (A) Percentage of subjects (n = 31) showing IFN-γ, IL-2 and IL-4 production against Tat, and (B) percentage of subjects (n = 26) showing anti-Tat CD4+ or CD8+ lymphoproliferative responses. (C) Percentage of subjects (n = 29) showing anti-Env production of IFN-γ, IL-2 and IL-4, and (D) percentage of subjects (n = 23) showing anti-Env CD4+ or CD8+ lymphoproliferative responses. (E) Percentage of subjects (n = 29) showing anti-Candida production of IFN-γ, IL-2 and IL-4, and (F) percentage of subjects (n = 23) showing anti-Candida CD4+ or CD8+ lymphoproliferative responses. (G) Percentage of subjects (n = 28) showing anti-CEF production of IFN-γ, IL-2 and IL-4, and (H) percentage of subjects (n = 23) showing anti-CEF CD4+ or CD8+ lymphoproliferative responses. The McNemar's test was used for the analyses: *p<0.05, **p<0.01. Anti-Tat response: IL-4, p = 0.0339; CD8+ proliferation, p = 0.0348. Anti-CEF response: IFN-γ, p = 0.0253; IL-4, p = 0.0339. (1.37 MB TIF) [file pone.0013540.s010.tif]
