# Supplementary material for: Therapeutic Immunization with HIV-1 Tat Reduces Immune Activation and Loss of Regulatory T-Cells and Improves Immune Function in Subjects on HAART
Source: PLoS One. 2010 Nov 11;5(11):e13540. doi: 10.1371/journal.pone.0013540 (PMC2978690; doi:10.1371/journal.pone.0013540)
Supplement: Table S1 — Baseline characteristics of study participants in ISS OBS T-002 for the Total Subjects and the Reference Group. (0.04 MB DOC) [file pone.0013540.s011.doc]

**Table S1.** Baseline characteristics of study participants in ISS OBS T-002 for the Total Subjects and the Reference Group.

|  | **Total Subjects** | **Reference Group** |
| --- | --- | --- |
|  | (*n* = 88)^a^ | (*n* = 32)^a^ |
| Age (yr)^b^ |  |  |
| Mean ± s.d. | 44 ± 8 | 42 ± 6 |
| Range | 26-65 | 30-55 |
| Sex (%) |  |  |
| Male | 75.6 | 68.8 |
| Female | 24.4 | 31.2 |
| CD4+ nadir (cells/μl) |  |  |
| Mean ± s.d. | 205 ± 141 | 356 ± 102 |
| Range | 2-612 | 253-612 |
| Time since diagnosis of HIV (yr)^c^ |  |  |
| Mean ± s.d. | 10 ± 7 | 12 ± 6 |
| Range | 1-23 | 2-21 |
| Time since HAART initiation (yr) |  |  |
| Mean ± s.d. | 6 ± 5 | 7 ± 5 |
| Range | 0-21 | 1-19 |
| Current HAART regimen (%)^d^ |  |  |
| Includes PI | 35.6 | 25.8 |
| Includes NNRTI | 56.3 | 58.1 |
| Includes NRTI | 8.1 | 16.1 |

^a^**Number of individuals.**

^b^Based on 87 individuals for the Total Subjects.

^c^Based on 77 individuals for the Total Subjects and on 27 individuals for the Reference Group.

^d^Based on 87 individuals for the Total Subjects and on 31 individuals for the Reference Group.
