# Supplementary material for: Therapeutic Immunization with HIV-1 Tat Reduces Immune Activation and Loss of Regulatory T-Cells and Improves Immune Function in Subjects on HAART
Source: PLoS One. 2010 Nov 11;5(11):e13540. doi: 10.1371/journal.pone.0013540 (PMC2978690; doi:10.1371/journal.pone.0013540)
Supplement: Table S2 — Immune activation markers and T-regs at baseline in subjects of ISS OBS T-002. (0.04 MB DOC) [file pone.0013540.s012.doc]

**Table S2.** Immune activation markers and T-regs at baseline in subjects of ISS OBS T-002.

|  |  | **Total Subjects** |  | **Reference Group** |
| --- | --- | --- | --- | --- |
|  | *n* | Mean ± s.e. | *n* | Mean ± s.e. |
| CD38^+^HLA-DR^-^ on CD8^+^ T cells (%) | 16 | 32.8 ± 2.8 | 6 | 34.3 ± 4.7 |
| HLA-DR^+^CD38^-^ on CD8^+^ T cells (%) | 16 | 14.4 ± 1.6 | 6 | 15.6 ± 2.6 |
| CD38^+^HLA-DR^+^ on CD8^+^ T cells (%) | 16 | 16.7 ± 2.6 | 6 | 15.3 ± 2.9 |
| CD38^+^ HLA-DR^-^ on CD4^+^ T cells (%) | 16 | 51.6 ± 4.1 | 6 | 54.4 ± 2.3 |
| HLA-DR^+^CD38^-^ on CD4^+^ T cells (%) | 16 | 6.6 ± 1.1 | 6 | 6.3 ± 1.0 |
| CD38^+^HLA-DR^+^ on CD4^+^T cells (%) | 16 | 4.6 ± 1.0 | 6 | 3.9 ± 0.8 |
| β2-microglobulin (mg/L) | n.d. | n.d. | 30 | 1.9 ± 0.1 |
| Neopterin (nmol/L) | n.d. | n.d. | 30 | 6.0 ± 0.3 |
| Total IgM (mg/dL) | n.d. | n.d. | 30 | 110 ± 9 |
| Total IgG (mg/dL) | n.d. | n.d. | 30 | 1308 ± 51 |
| Total IgA (mg/dL) | n.d. | n.d. | 30 | 283 ± 29 |
| CD25^+^ on CD4^+^ T cells (%) | 34 | 9.0 ± 0.4 | 20 | 9.0 ± 0.4 |
| FOXP3^+^ on CD4^+^CD25^+^ T cells (%) | 31 | 34.3 ± 2.2 | 19 | 36.6 ± 2.8 |
| CD25^+^FOXP3^+^ on CD4^+^ T cells (%) | 31 | 2.8 ± 0.2 | 19 | 2.9 ± 0.2 |
| CD25^+^FOXP3^+^ on CD4^+^ T cells (cells/μl) | 25 | 22.6 ± 2.2 | 15 | 27.6 ± 2.7 |

Mean values (± standard error) of immune activation markers and T-regs up to at week 48. *n* indicates the number of individuals tested for each parameter. n.d. not done.
