# Supplementary material for: Therapeutic Immunization with HIV-1 Tat Reduces Immune Activation and Loss of Regulatory T-Cells and Improves Immune Function in Subjects on HAART
Source: PLoS One. 2010 Nov 11;5(11):e13540. doi: 10.1371/journal.pone.0013540 (PMC2978690; doi:10.1371/journal.pone.0013540)
Supplement: Table S3 — Tat-specific cellular immune responses in subjects of ISS OBS T-002. (0.04 MB DOC) [file pone.0013540.s013.doc]

**Table S3.** Tat-specific cellular immune responses in subjects of ISS OBS T-002.

|  |  | **Total Subjects^b^** | |  | **Reference Group^c^** | |
| --- | --- | --- | --- | --- | --- | --- |
|  | *n* | **Baseline** | **Up to week 48** | *n* | **Baseline** | **Up to week 48** |
| **IFN-γ** |  |  |  |  |  |  |
| Peak^a^ (SFC/10^6^ cells) | 14 | 36 (14-62) | 96 (40-136)** | 2 | 47 (26-68) | 71 (40-102) |
| **IL-2** |  |  |  |  |  |  |
| Peak^a^ (SFC/10^6^ cells) | 29 | 8 (2-16) | 38 (16-68)** | 6 | 14 (0-56) | 39 (12-76) |
| **IL-4** |  |  |  |  |  |  |
| Peak^a^ (SFC/10^6^ cells) | 16 | 0 (0-7) | 21 (13-49)** | 7 | 0 (0-12) | 18 (12-28)* |
| **CD4 Proliferation** |  |  |  |  |  |  |
| Peak^a^ (fold increase) | 29 | 1.7 (1.3-2.5) | 3.1 (2.7-4.2)** | 8 | 1.4 (1.0-2.2) | 3.0 (2.7-3.9)* |
| **CD8 Proliferation** |  |  |  |  |  |  |
| Peak^a^ (fold increase) | 34 | 1.5 (1.1-2.9) | 2.9 (2.4-6.0)** | 11 | 1.2 (1.1-1.9) | 2.8 (2.1-4.0)* |

^a^Median (interquartile range) of peak of positive responses, weeks 12, 24, 36, 48.

^b^ Subject tested for cytokines: 87; for proliferation: 67.

^c^ Subject tested for cytokines: 31; for proliferation: 26.

* *P* < 0.05, ** *P* < 0.01
