# Supplementary material for: Therapeutic Immunization with HIV-1 Tat Reduces Immune Activation and Loss of Regulatory T-Cells and Improves Immune Function in Subjects on HAART
Source: PLoS One. 2010 Nov 11;5(11):e13540. doi: 10.1371/journal.pone.0013540 (PMC2978690; doi:10.1371/journal.pone.0013540)
Supplement: Table S4 — Cellular immune responses against Env in subjects of ISS OBS T-002. (0.04 MB DOC) [file pone.0013540.s014.doc]

**Table S4.** Cellular immune responses against Env in subjects of ISS OBS T-002.

|  |  | **Total Subjects^b^** | |  | **Reference Group^c^** | |
| --- | --- | --- | --- | --- | --- | --- |
|  | *n* | **Baseline** | **Up to week 48** | *n* | **Baseline** | **Up to week 48** |
| **IFN-γ** |  |  |  |  |  |  |
| Peak^a^ (SFC/10^6^ cells) | 37 | 132 (94-278) | 166 (82-282) | 15 | 118 (42-440) | 166 (110-310) |
| **IL-2** |  |  |  |  |  |  |
| Peak^a^ (SFC/10^6^ cells) | 39 | 54 (24-134) | 62 (46-92) | 14 | 71 (38-226) | 52 (34-82) |
| **IL-4** |  |  |  |  |  |  |
| Peak^a^ (SFC/10^6^ cells) | 55 | 146 (82-484) | 150 (70-304) | 19 | 160 (112-514) | 114 (60-310) |
| **CD4 Proliferation** |  |  |  |  |  |  |
| Peak^a^ (fold increase) | 15 | 0.3 (0.2-0.5) | 2.4 (2.0-4.5)** | 6 | 0.2 (0.1-0.3) | 2.8 (2.0-4.1)* |
| **CD8 Proliferation** |  |  |  |  |  |  |
| Peak^a^ (fold increase) | 22 | 0.6 (0.5-0.9) | 4.3 (2.6-6.3)** | 6 | 0.7 (0.6-0.8) | 2.8 (2.6-5.5)* |

^a^Median (interquartile range) of peak of positive responses, weeks 12, 24, 36, 48.

^b^ Subject tested for cytokines: 72; for proliferation:64.

^c^ Subject tested for cytokines: 29; for proliferation: 23.

* *P* < 0.05, ** *P* < 0.01
