# Supplementary material for: Therapeutic Immunization with HIV-1 Tat Reduces Immune Activation and Loss of Regulatory T-Cells and Improves Immune Function in Subjects on HAART
Source: PLoS One. 2010 Nov 11;5(11):e13540. doi: 10.1371/journal.pone.0013540 (PMC2978690; doi:10.1371/journal.pone.0013540)
Supplement: Table S5 — Cellular immune responses against Candida in subjects of ISS OBS T-002. (0.04 MB DOC) [file pone.0013540.s015.doc]

**Table S5.** Cellular immune responses against Candida in subjects of ISS OBS T-002.

|  |  | **Total Subjects^b^** | |  | **Reference Group^c^** | |
| --- | --- | --- | --- | --- | --- | --- |
|  | *n* | **Baseline** | **Up to week 48** | *n* | **Baseline** | **Up to week 48** |
| **IFN-γ** |  |  |  |  |  |  |
| Peak^a^ (SFC/10^6^ cells) | 24 | 43 (4-128) | 73 (27-180)** | 6 | 104 (36-238) | 139 (92-270) |
| **IL-2** |  |  |  |  |  |  |
| Peak^a^ (SFC/10^6^ cells) | 52 | 143 (78-289) | 160 (77-274) | 20 | 218 (97-316) | 223 (77-343) |
| **IL-4** |  |  |  |  |  |  |
| Peak^a^ (SFC/10^6^ cells) | 31 | 12 (0-52) | 62 (38-122)** | 13 | 18 (4-46) | 62 (48-130)* |
| **CD4 Proliferation** |  |  |  |  |  |  |
| Peak^a^ (fold increase) | 33 | 1.4 (1.0-3.2) | 3.0 (2.4-5.1)** | 9 | 1.4 (0.9-2.1) | 3.7 (2.7-4.5)* |
| **CD8 Proliferation** |  |  |  |  |  |  |
| Peak^a^ (fold increase) | 25 | 1.3 (0.8-1.9) | 3.7 (2.7-7.2)** | 7 | 1.3 (0.3-1.6) | 3.7 (3.2-4.0) |

^a^Median (interquartile range) of peak of positive responses, weeks 12, 24, 36, 48.

^b^ Subject tested for cytokines: 74; for proliferation: 64.

^c^ Subject tested for cytokines: 29; for proliferation: 23.

* *P* < 0.05, ** *P* < 0.01
