# Supplementary material for: Therapeutic Immunization with HIV-1 Tat Reduces Immune Activation and Loss of Regulatory T-Cells and Improves Immune Function in Subjects on HAART
Source: PLoS One. 2010 Nov 11;5(11):e13540. doi: 10.1371/journal.pone.0013540 (PMC2978690; doi:10.1371/journal.pone.0013540)
Supplement: Table S6 — Cellular immune responses against CEF in subjects of ISS OBS T-002. (0.04 MB DOC) [file pone.0013540.s016.doc]

**Table S6.** Cellular immune responses against CEF in subjects of ISS OBS T-002.

|  |  | **Total Subjects^b^** | |  | **Reference Group^c^** | |
| --- | --- | --- | --- | --- | --- | --- |
|  | *n* | **Baseline** | **Up to week 48** | *n* | **Baseline** | **Up to week 48** |
| **IFN-γ** |  |  |  |  |  |  |
| Peak^a^ (SFC/10^6^ cells) | 68 | 844 (214-1422) | 1216 (454-2016)** | 24 | 1053 (571-1573) | 1348 (1014-3960)** |
| **IL-2** |  |  |  |  |  |  |
| Peak^a^ (SFC/10^6^ cells) | 69 | 110 (50-468) | 510 (284-726)** | 26 | 143 (48-468) | 464 (170-724)** |
| **IL-4** |  |  |  |  |  |  |
| Peak^a^ (SFC/10^6^ cells) | 72 | 101 (28-215) | 459 (154-995)** | 28 | 86 (28-174) | 386 (152-648)** |
| **CD4 Proliferation** |  |  |  |  |  |  |
| Peak^a^ (fold increase) | 8 | 1.3 (1.1-1.7) | 3.0 (2.1-5.4)** | 1 | 1.2 (1.2-1.2) | 3.0 (3.0-3.0) |
| **CD8 Proliferation** |  |  |  |  |  |  |
| Peak^a^ (fold increase) | 18 | 1.6 (1.2-2.3) | 2.8 (2.2-3.9)* | 8 | 1.8 (0.9-2.6) | 2.2 (2.0-3.7) |

^a^Median (interquartile range) of peak of positive responses, weeks 12, 24, 36, 48.

^b^ Subject tested for cytokines: 78; for proliferation: 64.

^c^ Subject tested for cytokines: 28; for proliferation: 23.

* *P* < 0.05, ** *P* < 0.01
