# Supplementary material for: Therapeutic Immunization with HIV-1 Tat Reduces Immune Activation and Loss of Regulatory T-Cells and Improves Immune Function in Subjects on HAART
Source: PLoS One. 2010 Nov 11;5(11):e13540. doi: 10.1371/journal.pone.0013540 (PMC2978690; doi:10.1371/journal.pone.0013540)
Supplement: Protocol S1 — Trial Protocol. (0.89 MB PDF) [file pone.0013540.s017.pdf]

Sponsor:
AIDS National Center
Istituto Superiore di Sanità (ISS)
Viale Regina Elena, 299
00161 - Rome, Italy

Coordinating Center:
Policlinico di Modena

Principal Investigator:
Prof. Roberto Esposito

Protocol Number: ISS T-002
EudraCT number: 2007-007200-16

A PHASE II RANDOMIZED, OPEN LABEL, IMMUNOGENICITY AND SAFETY TRIAL OF THE VACCINE BASED ON THE RECOMBINANT BIOLOGICALLY ACTIVE HIV-1 TAT PROTEIN IN ANTI-TAT NEGATIVE HIV-1 INFECTED HAART-TREATED ADULT SUBJECTS

Version: dated **12th February 2008**

Study phase: II

Contract Research Organization:
Opera Srl
Via Sampierdarena, 33/2
16149 - Genoa, Italy

The information contained in this protocol are confidential and will not be disclosed to anyone without written authorization from Istituto Superiore di Sanità, except to the extent necessary to obtain Informed Consent from those subjects to whom investigational drug may be administered or for eventual discussions with regulatory authorities, Ethical Committees or persons participating in the conduct of the study.

CONFIDENTIAL

LIST OF CLINICAL SITES AND PRINCIPAL INVESTIGATORS

1) Policlinico di Modena<br>Prof. R. Esposito<br>L.go del Pozzo, 71<br>41100 Modena	***7) Ospedale S. Maria Annunziata***<br>Prof. F. Mazzotta<br>Via dell'Antella, 58<br>50011 Antella, Firenze
2) Ospedale S. Raffaele<br>Prof. A. Lazzarin<br>Via Stamira d'Ancona, 20<br>20127 Milano	***8) IFO-San Gallicano***<br>Prof. G. Palamara<br>Via di S. Gallicano, 25/a<br>00153 Roma
3) Ospedale Sacco<br>Prof. M. Galli<br>Via G.B. Grassi, 74<br>20157 Milano	***9) Policlinico di Bari***<br>Prof. G. Pastore<br>Piazza Giulio Cesare, 11<br>70124 Bari
4) Ospedale A. di Savoia<br>Prof. G. Di Perri<br>C.so Svizzera, 164<br>10149 Torino	***10) Ospedale S.M. Goretti***<br>Prof. F. Soscia<br>Via Guido Reni, 1<br>04100 Latina
5) Spedali Civili di Brescia<br>Prof. G. Carosi<br>Piazzale Spedali Civili, 1<br>25123 Brescia	***11) INMI "L. Spallanzani"***<br>IV Divisione Malattie Infettive<br>Prof. P. Narciso<br>Via Portuense, 292<br>00149 Roma
6) Arcispedale S. Anna<br>Prof. F. Ghinelli<br>Corso Giovecca, 203<br>44100 Ferrara	***12) INMI "L. Spallanzani"***<br>III Divisione Malattie Infettive<br>Prof. A. Antinori<br>Via Portuense, 292<br>00149 Roma

LIST OF OTHER PARTICIPANTS

Sponsor
AIDS National Center
Istituto Superiore di Sanità (ISS)
Viale Regina Elena, 299
00161 Rome, Italy
Tel. +39 06 49903209
Fax +39 06 49903002
Sponsor Responsible – Dr. Barbara Ensoli

Core Laboratory
Ospedale S. Gallicano
Istituti Fisioterapici Ospitalieri
Via San Gallicano, 25 A
00153 Rome, Italy
Tel. +39 06 58543682
Fax +39 06 58543680
Laboratory Responsible – Dr. Fabrizio Ensoli

Opera Srl - CRO
Via Sampierdarena, 33/2
16149 Genoa, Italy
Tel. +39 010 4699623
Fax +39 010 6591120
President – Dr. Dionisio Franco Barattini
Scientific Responsible – Dr. Laura Michellini

EMERGENCY CONTACTS

Role in the study	Name	Telephone/Fax/e-mail contacts
Sponsor<br>AIDS National Center<br>Istituto Superiore di Sanità	Dr. Barbara Ensoli	Tel. +39 06 49903209<br>Fax +39 06 49903002<br>E-mail: barbara.ensoli@iss.it
CRO<br>Opera Srl	Dr. Dionisio Franco Barattini<br><br><br>Dr. Laura Michellini	Tel. +39 010 4699623<br>Fax +39 010 6591120<br>E-mail: barattini@operacro.com<br>E-mail: michellini@operacro.com

TABLE OF CONTENTS

	**PROTOCOL SYNOPSIS**	
	Title	Page 7
	Study Objectives	Page 7
	Study Design	Page 7
	Planned Duration of the Study	Page 7
	Number of Subjects	Page 7
	Study Population	Page 7
	Inclusion Criteria	Page 7
	Exclusion Criteria	Page 8
	Study Medication, Administration and Dosage Regimens	Page 9
	Study Duration	Page 9
	Population for Statistical Evaluation	Page 9
	Outcome Variables	Page 9
**1.**	**INTRODUCTION AND RATIONALE**	Page 11
1.1	Rationale	Page 11
1.2	Background on Vaccine Preparation	Page 12
1.3	Preclinical Studies	Page 15
1.4	Clinical Studies	Page 16
1.5	Safety	Page 19
1.5.1	Preventive Protocol (ISS P-001)	Page 20
1.5.2	Therapeutic Protocol (ISS T-001)	Page 27
1.6	Immunogenicity	Page 35
1.6.1	Preventive Protocol (ISS P-001)	Page 35
1.6.2	Therapeutic Protocol (ISS T-001)	Page 37
**2.**	**STUDY OBJECTIVES**	Page 40
2.1	Primary Objectives	Page 40
2.2	Secondary Objectives	Page 41
**3.**	**STUDY DESIGN**	Page 41
3.1	Rationale for the Selection of Vaccine Dosage and Schedule of Immunization	Page 41
**4.**	**STUDY MEDICATION AND DOSAGE REGIMENS**	Page 42
4.1	Study Medication – Background on Study Medication Preparation	Page 42
4.1.1	Administration	Page 42
4.1.2	Dosage Regimens	Page 43
4.2	Study Duration	Page 43
4.3	Study Medication Delivery	Page 44
4.4	Packaging and Labeling of Study Medication	Page 44
4.5	Study Medication Storage and Accountability	Page 45
4.6	Randomization Codes	Page 45
4.7	Concomitant Treatments	Page 45
4.7.1	Prior Treatments	Page 45
4.7.2	Antiretroviral Treatments	Page 46

**5.**	**METHODS**	Page 46
5.1	Diagnosis	Page 46
5.2	Psychological Evaluation	Page 46
5.3	Inclusion Criteria	Page 46
5.4	Exclusion Criteria	Page 47
5.5	Screening Failures	Page 48
5.6	Randomization	Page 48
5.7	Blinding	Page 48
5.8	Study Assessments	Page 48
5.8.1	Immunological Evaluations	Page 49
5.8.2	Virological Evaluations	Page 50
5.8.3	Sample Collection, Storage and Shipment	Page 50
5.9	Schedule of Study Procedures	Page 51
5.10	Compliance	Page 57
**6.**	**STUDY INTERRUPTION**	Page 57
6.1	Premature Discontinuation of the Treatment/Study per Subject	Page 57
6.2	Premature Discontinuation of the Study in a Clinical Site	Page 58
6.3	Premature Discontinuation of the Whole Study	Page 58
**7.**	**ENDPOINTS**	Page 59
7.1	Primary Endpoint	Page 59
7.2	Primary Endpoint Variables and Measurements	Page 59
7.3	Secondary Endpoints	Page 60
7.4	Secondary Endpoints Variables and Measurements	Page 60
**8.**	**TOLERABILITY**	Page 60
8.1	Adverse Events Definitions	Page 60
8.1.1	Adverse Event (AE)	Page 60
8.1.2	Serious Adverse Event (SAE)	Page 61
8.1.3	Adverse Drug Reaction (ADR)	Page 61
8.1.4	Serious Adverse Drug Reaction (SADR)	Page 61
8.2	Reporting of Adverse Events/Adverse Drug Reactions	Page 61
8.3	Reporting of Serious Adverse Events/Serious Adverse Drug Reactions	Page 62
8.4	Subjects' Protection	Page 63
**9.**	**DATA RECORDING, MONITORING AND DATA MANAGEMENT**	Page 64
9.1	Data Recording	Page 64
9.2	Data Monitoring	Page 64
9.3	Data Management	Page 64
**10.**	**STATISTICAL ANALYSIS**	Page 65
10.1	Statistical Program	Page 65
10.2	Parameters	Page 65
10.3	Sample Size Determination	Page 65
10.4	Population for Statistical Evaluation	Page 66
10.5	Analytical Methods	Page 66
10.6	Primary Endpoint Analysis	Page 66
10.7	Secondary Endpoints Analysis	Page 66

11. ETHICAL AND ADMINISTRATIVE PROCEDURES Page 67
11.1 Informed Consent Page 67
11.2 Ethical and Administrative Issues Page 67
11.3 Subject Data Protection - Direct Access to Source Data Page 68
11.4 Insurance, Indemnity and Refunds Page 68
11.5 Audits and Inspections Page 69
11.6 Training to Study Staff Page 69
11.7 Records Retention Page 69

12. CASE REPORT FORMS Page 69

13. PUBLICATION OF RESULTS Page 69

14. STUDY TIMETABLE Page 70

15. INVESTIGATOR'S AGREEMENT Page 71

16. REFERENCES Page 72

APPENDICES

Appendix I WHO - Division of AIDS - Table for Grading Severity of Adult Adverse Experiences Page 76
Appendix II Flow-Chart Page 79
Appendix III Facsimile of drug labels Page 80
Appendix IV Declaration of Helsinki Page 81
Appendix V Psychological Evaluation Page 85
Appendix VI List of Abbreviations and Symbols Page 100

PROTOCOL SYNOPSIS

Title:
A Phase II randomized, open label, immunogenicity and safety trial of the vaccine based on the recombinant biologically active HIV-1 Tat protein in anti-Tat negative HIV-1 infected HAART-treated adult subjects.

Study Objectives:
The primary objective of this study is to demonstrate that the Tat vaccine is immunogenic in HIV-1 infected, anti-Tat antibody negative subjects treated with antiretroviral therapy. The secondary objective is to monitor the safety of the Tat vaccine in immunized subjects.

Study Design:
Phase II, randomized, open label study.

Planned Duration of the Study:
The enrolment is planned to start on April 2008 and the enrolment period will be extended to ensure completion of the case number.
The study duration for each subject enrolled will be of 48 weeks, unless premature discontinuation.

Number of Subjects:
Totally 128 subjects will be randomized into 2 arms and each arm will be divided in two groups:

Arm A:
Group I: 32 subjects with 5 intradermal administrations of the Tat vaccine (7.5 μg)
Group II: 32 subjects with 5 intradermal administrations of the Tat vaccine (30 μg)

Arm B:
Group I: 32 subjects with 3 intradermal administrations of the Tat vaccine (7.5 μg)
Group II: 32 subjects with 3 intradermal administrations of the Tat vaccine (30 μg)

Study Population:
HIV-1 infected adult subjects of either gender, 18-55 years of age, anti-Tat antibody negative, HAART-treated with chronic suppressed HIV-1 infection: $CD4^+$ T cell counts ≥ 400 cells/μl, levels of plasma viremia < 50 copies/ml in the last 6 months prior to the screening and without a history of virologic rebound. The pre-HAART CD4 nadir must be > 250 cells/μl.

Inclusion Criteria:
1) Age 18-55 years;
2) Anti-Tat antibody negative subjects;
3) HIV-1 infected subjects under successful HAART treatment (with chronic suppressed HIV-1 infection) with $CD4^+$ T cell counts ≥ 400 cells/μl determinated by 2 separate evaluations within the 3 weeks pre-study screening period (at day -21 and then between day -14 and -7);

4) HIV plasma viremia < 50 copies/ml in the last 6 months prior to the screening and without a history of virological rebound;
5) Subjects with pre-HAART CD4 nadir > 250 cells/μl;
6) Availability for the planned study duration;
7) Negative pregnancy test for women of childbearing potential (to be performed during the screening phase and just before the immunizations) and use of an acceptable mean of contraception (condom, hormonal or mechanical methods) for one month prior to immunization and for the all duration of the study;
8) Signed informed consent.

Exclusion Criteria:

1) History of AIDS-related opportunistic or neoplastic disease;
2) History of encephalopathy, neuropathy, or unstable CNS pathology (HIV or non-HIV related);
3) History of non-HIV related neoplastic diseases, autoimmune diseases, severe and/or persistent angina or cardiac arithymias, or severe or uncontrolled systemic disease (e.g., unstable or uncompensated respiratory, cardiac, hepatic, thyroid gland or renal disease);
4) Any evidence, as judged by the investigator, of unstable cardio-vascular disease (e.g. unstable hypertensive disease needing modification or introduction of an anti-hypertensive treatment);
5) Laboratory findings exceeding the normal range adopted by each clinical site laboratory for haematology and biochemistry assessments will make undesirable for the subject the participation to the study. In particular, subjects presenting AST/ALT > 3 x the upper limit of normal will be excluded, as well as AST/ALT > 5 x the upper limit of normal in case of subjects having co-infections HIV/HCV related;
6) Chest radiography, within 6 months prior to study screening visit, showing evidence of active or acute cardiac or pulmonary disease;
7) History of anaphylaxis or serious adverse reactions to vaccines as well as serum IgE levels exceeding 1000 U.I./ml;
8) History of serious allergic reaction to any substance, requiring hospitalization or emergent medical care (e.g. Steven-Johnson syndrome, bronchospasm, or hypotension);
9) Early syphilis documented by Syphilis serology test [*NOTE: if serology is documented to be a false positive or due to a remote (> 6 months), successfully treated infection, the subject is eligible*];
10) Active tuberculosis documented PPD skin test within one year [*NOTE: if the PPD skin test is positive, then a chest x-ray will be done and if no findings consistent with active pulmonary tuberculosis and no indications exist for prophylaxis or treatment, the subject is eligible for participation in this trial*];
11) Medical or psychiatric condition which preclude subject compliance with the protocol. Specifically, persons with psychotic disorders, major affective disorders, suicidal ideation are to be excluded;
12) Current use of psychotrophic drugs prescribed for major psychotic disorders;
13) Use of any experimental HIV therapy or participation in another experimental protocol;
14) Current or prior therapy with immunomodulators or immunosuppressive drugs and anticoagulant drugs within 30 days prior to study medication administration;
15) Concomitant treatment for HBV or HCV infections;
16) Live attenuated vaccines within 60 days of study inclusion [*NOTE: Medically indicated sub-unit or killed vaccines (e.g., influenza, pneumococcal, hepatitis A and B) are not exclusionary, but should be given at least 4 weeks away from HIV immunizations*];
17) Receipt of blood products or immunoglobulin in the past year;

18) Previous participation in an HIV-1 vaccine trial;
19) Drug and/or alcohol abuse;
20) Pregnant or lactating women.

Study Medication, Administration and Dosage Regimens:
The study medication is the biologically active recombinant Tat protein. Four vaccination regimens will be tested by intradermal administration of the Tat vaccine at two different doses (7.5 μg or 30 μg) in 5 or 3 immunizations, according to the following immunization schedule.

			**Immunization Schedule**				
**Arm A**	**Dose Levels**	**Accrual**	**Week 0**	**Week 4**	**Week 8**	**Week 12**	**Week 16**
Group I	Tat 7.5 μg (id)	32	X	X	X	X	X
Group II	Tat 30 μg (id)	32	X	X	X	X	X
	Subtotal	n = 64					
**Arm B**	**Dose Levels**	**Accrual**	**Week 0**	**Week 4**	**Week 8**	**Week 12**	**Week 16**
Group I	Tat 7.5 μg (id)	32	X	X	X	-	-
Group II	Tat 30 μg (id)	32	X	X	X	-	-
	Subtotal	n = 64					
	**Total**	**128**					

Study Duration:
The treatment duration will be of 16 weeks for Arm A and of 8 weeks for Arm B, with immunizations every 4 weeks.
To evaluate the eligibility, subjects will be screened before treatment initiation (screening phase) and then monitored until week 48 from the first immunization (for a total of a 32-weeks period after the treatment completion for subjects in Arm A and a 40-weeks period after the treatment completion for subjects in Arm B).

Population for Statistical Evaluation:
Two groups of subject populations will be considered for statistical analysis.
The immunogenicity population, representing all randomized subjects who received at least 3 immunizations and have at least one post-baseline immunogenicity evaluation.
The safety population, representing all randomized subjects who received at least one administration of the vaccine.

Outcome Variables:
Primary Efficacy Variables
- Induction of IgM, IgG, IgA anti-Tat antibodies as specific anti-Tat humoral immune response; neutralization of Tat activity by *in vitro* tests (inhibition of Tat-induced HIV rescue assay)

- Induction or increase of lymphoproliferation (CFSE staining) and/or *in vitro* γIFN, IL-4, IL-2 production (Elispot) in response to Tat, as specific anti-Tat cellular immune response.

Safety Variables

- adverse events, including any significant change in hematological/biochemical laboratory parameters.

First interim analysis: at week-24 of study conduction.
Final analysis: at week 48, after study conclusion.

1. INTRODUCTION AND RATIONALE

This Phase II study is directed at evaluating the immunogenicity (as a primary end-point) and the safety (as a secondary end-point), of the recombinant HIV-1 Tat vaccine in HIV-1 infected adult subjects, anti-Tat antibody negative, HAART-treated with chronic suppressed HIV-1 infection, $CD4^+$ T cell counts $\geq$ 400 cells/µl, levels of plasma viremia < 50 copies/ml in the last 6 months prior to the screening and without a history of virologic rebound.
This vaccine strategy has been evaluated in preclinical studies in cynomolgus monkeys and in phase I preventive and therapeutic clinical trials, as described below.

1.1 Rationale

Over the last 20 years most of the efforts in HIV vaccine development have been focused on achieving sterilizing immunity by targeting the Envelope protein (Env) of HIV, that is responsible for the binding and entry of the virus, with the rationale of generating neutralizing antibodies (NA) capable of protecting from infection (Wahren, 2002). Alternatively, more recent approaches have been attempted, by combining multiple HIV antigens with the rationale of generating strong antiviral cellular immune responses capable of preventing infection and/or reducing virus replication and progression to disease. However, results from pre-clinical and clinical trials, including the first phase III trial (AIDSVAX by VaxGen) and the first phase II "Proof-of-Concept" trial (HVTN 502/Merck 023) have been largely disappointing since no protection from primary infection and no effect on viral load have been observed. The inability of such vaccines to elicit protective immune responses can be, at least in part, explained by the high Env variability that hampers recognition of relevant epitopes by NA, and by the heavy glycosylation of gp120 that contributes in hiding critical (neutralizing) Env-epitopes (reviewed in Burton, 1997). On the other hand, the induction of strong cellular responses against a combination of different HIV antigens (Gag, Pol and Nef) failed both in preventing and in controlling infection and virus replication (news release Merck/HVTN, 2007) suggesting that a more balanced induction of humoral and cellular immune responses might be necessary to increase the chance of success of the candidate vaccines. New and alternative vaccine strategies have been therefore developed aimed at blocking disease onset by targeting viral regulatory genes, which are essential for virus replication and infectivity. Control of infection, which is achieved in the absence of sterilizing immunity, should provide protection from disease progression and reduce virus transmission to healthy individuals. Thus, this approach may be effective for both preventive and therapeutic vaccine strategies.

Being a very early regulatory protein and playing a major role in HIV-1 replication and pathogenesis, Tat represents an optimal candidate for such vaccine strategies (Ensoli, 1990, 1993 and 1994; Chang, 1997; Ensoli, 2006). Tat is a key viral regulatory protein produced very early after infection, even prior to HIV integration, and necessary for viral gene expression (Arya, 1985; Fisher, 1986; Ensoli, 1993; Wu, 2001), cell-to-cell virus transmission and disease progression. In fact, in the absence of Tat no or negligible amounts of structural proteins are expressed and, therefore, no infectious virus is made. Further, Tat is released by the infected T lymphocytes in the extracellular milieu (Ensoli, 1990 and 1993; Chang, 1997) and enters both infected cells, in which promotes HIV-1 replication, while exerting multiple effects on uninfected cells, which facilitate, directly or indirectly, cell recruitment and activation, thus providing new cell targets for HIV tissue propagation and systemic spreading of the infection (Ensoli, 1990 and 1993; Chang, 1997; Shutt and Soll, 1999; Koedel, 1999; Arora, 2002; Caputo, 2004; James, 2004; Ferrantelli, 2004).

Several studies suggest that an immune response to Tat has a protective role and may control the progression of the disease *in vivo* (Reiss, 1990; Rodman, 1993; Re, 1995; Zagury, 1998; Re, 2001). In particular, a higher prevalence of anti-Tat antibodies has been shown in asymptomatic

HIV-infected individuals as compared to patients in advanced stages of the disease (Krone, 1988; Demirhan, 2000; Re, 2001) and in non-progressors as compared to fast progressors (Zagury, 1998).

A cross-sectional and longitudinal study has been recently performed in a cohort of 252 individuals with known dates of seroconversion and a medium follow-up of 7.2 years (Rezza, 2005). The risk of developing AIDS or severe immunodeficiency was 60% lower for anti-Tat positive individuals as compared to anti-Tat negative individuals. A longitudinal analysis performed on 139 individuals (with at least two serum samples) indicated no progression to disease in persistently anti-Tat positive individuals (n = 10). Even individuals with a transient anti-Tat positive determination had a slower progression, as compared to persistently anti-Tat negative individuals. In particular, none of the persistently anti-Tat positive individuals developed AIDS, whereas AIDS or severe immunodeficiency occurred in 53 individuals among those who were anti-Tat negative. These results indicate that presence of anti-Tat antibodies is predictive of a slower progression to AIDS and/or severe immunodeficiency (Rezza, 2005).

Moreover, Tat is conserved in its immunogenic regions (both B and T cell) among all subtypes. Recent data, in fact, indicate an effective cross-clade recognition of clade B strain-derived (BH-10) Tat protein from the HTLV-IIIB lab-adapted virus strain (Buttò, 2003), which was isolated about 20 years ago (Ratner, 1985), by sera from individuals infected with viruses circulating at the present in Italy and in Africa, thus reflecting the high degree of conservation of the corresponding Tat regions. Specifically, sera from Italian, Ugandan and South African patients who are mainly infected with A, B, C and D and to a lesser extent, with F and G HIV-1 subtypes, recognize the BH-10 Tat protein at similar levels (e.g. prevalence and titers of anti-Tat antibodies) (Buttò, 2003). This observation is reinforced by the results of sequence conservation analysis, demonstrating that the predicted amino acidic sequence of Tat is well conserved among the different circulating viruses belonging to distinct HIV-1 clades and presents a relatively high degree of homology with the BH-10 Tat sequence (Buttò, 2003). These findings indicate that the overall identity of Tat is preserved and provide strong formal evidence that a Tat-based vaccine may indeed be used in the different geographic areas of the world, since it is capable of inducing a broad immune response against different virus clades.

The Tat vaccine has also the advantage of maintaining the vaccinees HIV-negative according to the current serological tests for diagnosis of HIV infection, since it does not contain structural HIV proteins on which these tests are based. This greatly facilitates recruitment and trial participation as well as vaccinees monitoring by avoiding immunization-induced seroconversion.

Preclinical studies performed in different animal models, including mice and cynomolgus monkeys, demonstrated that vaccination with a biologically active Tat protein or <u>tat</u> DNA is safe, elicits a broad and specific immune response and, most importantly, induces a long-term protection against infection with a highly pathogenic virus (SHIV 89.6P), which rapidly causes AIDS and death in these monkeys (Cafaro, 1999, 2000 and 2001). Based on these results Phase I preventive and therapeutic trials have been sponsored by ISS and conducted in 4 clinical centers in Italy. The results of the phase I clinical trials indicate that the Tat vaccine is safe and immunogenic in both uninfected and infected individuals, having efficiently induced both humoral and cellular immune responses against the Tat protein.

1.2 Background on Vaccine Preparation

The biologically active Tat utilized for vaccination has been produced under Good Manufacturing Practice (GMP) conditions in order to be administered in humans. Tat biologically active protein is a recombinant protein obtained from a lysate of *E. coli* cells engineered with the pET-tat plasmid, constructed for Tat expression. Briefly, the pET3c-tat and pLysS plasmids were constructed and

optimized for Tat expression. The pET-tat was constructed by cloning the 261 bp tat into the 5' *NdeI* and 3' *BamH1* site of pET-3c. The plasmid was transferred in an *E. coli* strain, BL21(DE3), selected for optimal growth and protein expression. The lysates obtained from the engineered *E. coli* cells are filtered and purified by DEAE Sepharose Fast Flow column and Heparin Sepharose CL-6B column. Following purification, the Tat protein has been formulated in 0.5 mL of potassium phosphate saline buffer, pH 7.4, containing 1% sucrose and 1% human serum albumin (HSA), in two doses: 7.5 µg/dose and 30 µg/dose. This formulation has been defined in order to maintain the biological activity of the protein in a liquid form, when the vaccine is stored at -80°C (described in "HIV-1 recombinant Tat protein: Evaluation of Comparability" submitted to the Italian Ministry of Health for the past phase I trials). The storage conditions of the vaccine have been accurately detailed in the clinical protocols, and the cold chain, from the manufacturer to the clinical sites, is being strictly monitored.
During the phase I clinical trials (ISS P-001 and ISS T-001) it was also performed a long term *Stability Study* to evaluate the range of time in which the biological activity and the quality of the drug substance did not result modified from the storage process of the Tat protein. The protocol included the evaluation of physicochemical properties, immunochemical properties, biological activity and sterility performed after 3, 6, 12, 18 months. Being unmodified the protein stored until 18 months, additional time points at 24 and 30 months were evaluated.
The results indicated that no or little modifications resulted from the process of storage until 24 months.
A similar Stability Study will be performed to confirm stability data on the Tat protein produced for the phase II clinical trial.

The biological activity of Tat is assayed at picomolar to nanomolar concentrations and it is defined by the ability of the protein of entering dendritic cells according to specific parameters of doses and time (Fanales-Belasio, 2002). The assessment of the biological activity properties constitutes an equally essential step in establishing a complete characterization profile. The quantitative measure of biological activity, based on the attribute of the active substance, which is linked to the relevant biological properties, is defined as <u>potency test</u>. The biological activity of the recombinant Tat protein can be evaluated by using several tests, such as the uptake by MDDCs, the rescue of a Tat-defective provirus, the transactivation of the HIV-1 LTR (Ensoli, 1992 and 1993; Barillari 1993 and 1999; Fiorelli, 1995; Fanales-Belasio, 2002). Due to its high levels of reproducibility and sensitivity, the uptake by MDDC has been selected as the potency test for the recombinant Tat protein.
Extracellular Tat is taken up by cells (Frankel and Pabo, 1988; Ensoli 1993; Chang 1997; Tyagi 2001), and, unlike most soluble proteins, enters the MHC class I pathway of presentation and elicits CTL activity (Moy 1996; Kim 1997). MDDCs, among the most potent APCs, take up Tat much more efficiently than other cell types such as T cell blasts and B-lymphoblastoid cell lines and in a time-, dose- and maturation-dependent fashion (Fanales-Belasio 2002) (Fig. 1). This process is markedly hampered by the oxidation/inactivation of the protein (Fig. 1), and by low temperature. Moreover, Tat also induces MDDCs maturation, as indicated by the increase of both the surface expression of MHC and costimulatory (CD40, CD80, CD86) molecules and by the production of interleukin-12 (IL-12) and tumor necrosis factor-α (TNF-α) or the β-chemokines MIP-1α, MIP-1β, and RANTES (Fanales-Belasio 2002). In contrast, oxidized Tat has no effects.

Fig. 1

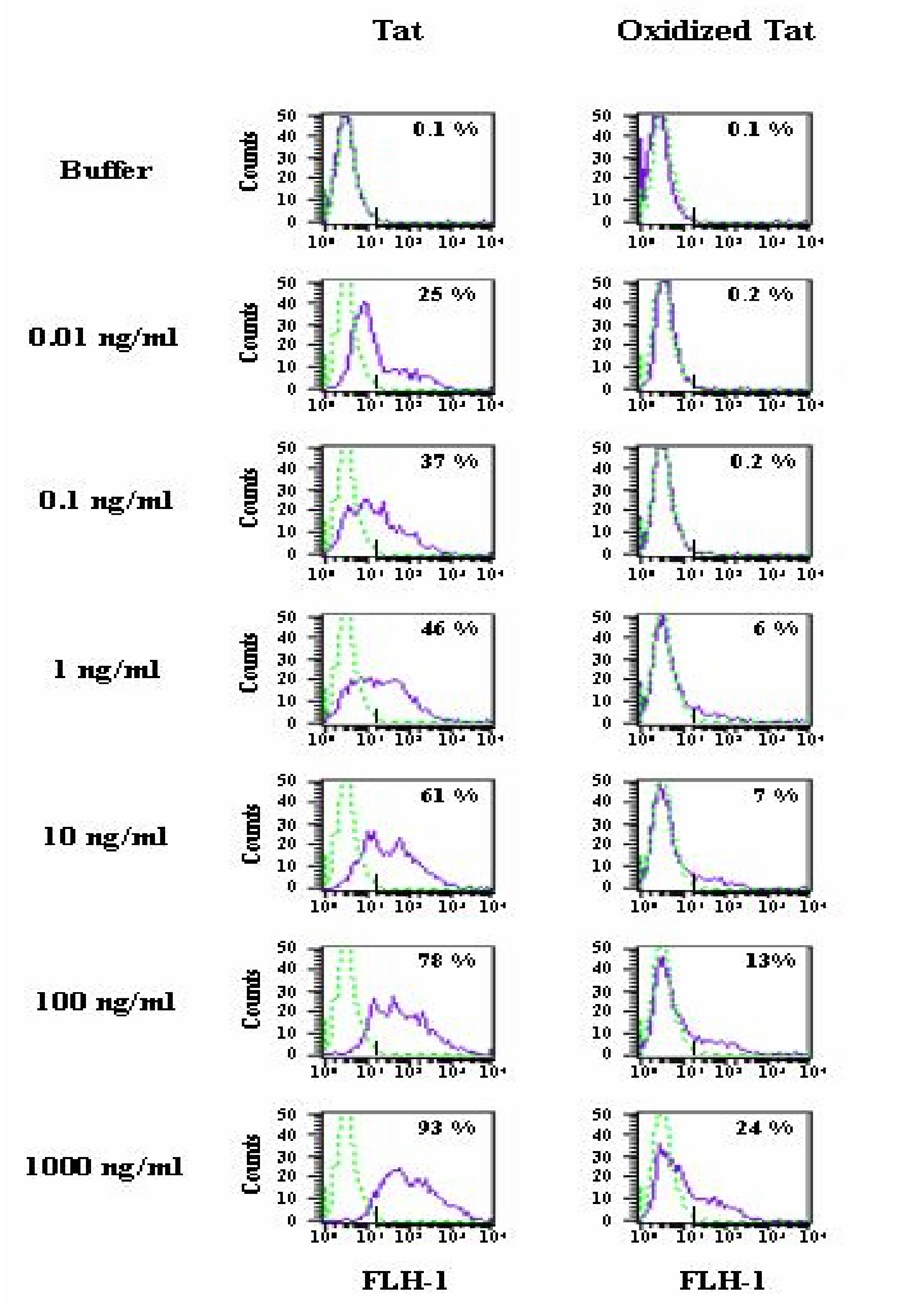

The assessment of the biological activity of Tat can be performed also by using a physicochemical assay, such as the HPLC. As indicated in Figure 2, the loss of biological activity induced by oxidation of the protein, corresponds to the loss of the Tat protein peak and to the appearance of smaller peaks corresponding to the oxidized forms of the protein, as evidenced by loading a higher amount of oxidized protein (20 µg vs. 10 µg, Fig. 2) in the HPLC test. This observation confirmed

the reliability of the HPLC assay in the evaluation of protein content after the GMP fermentation and the purification process.

Fig. 2

HPLC

Abs at 280 nm

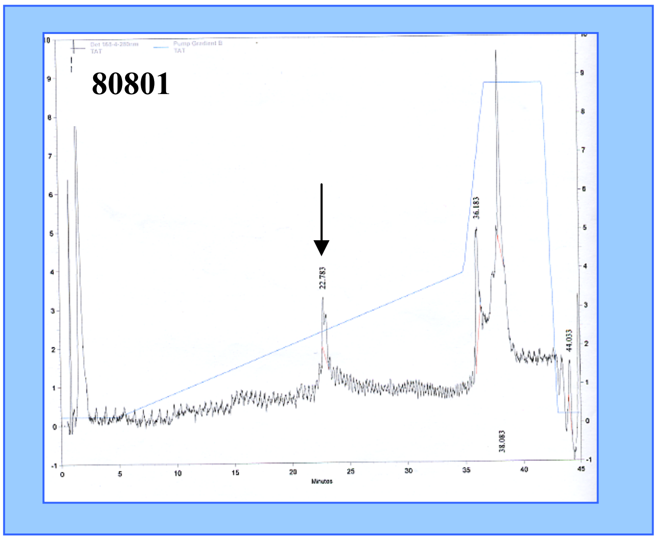

Oxidized Tat

20 µg loaded

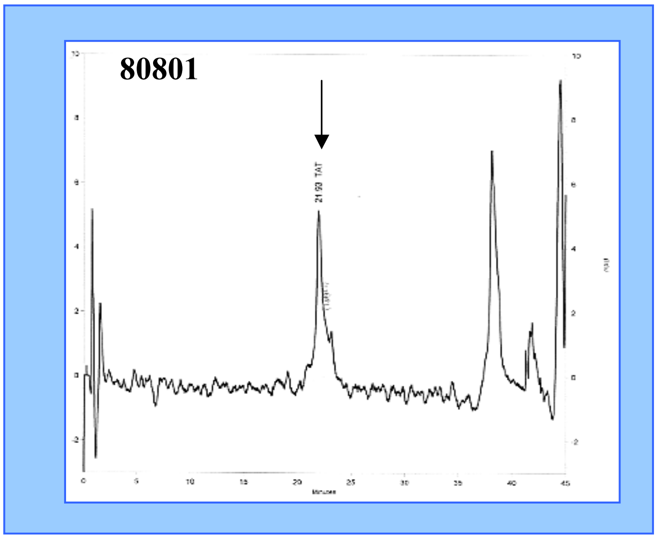

Biologically Active Tat
10 µg loaded

For this reason the protein will be thawed at the moment of injection in the absence of light and to be handled carefully.

1.3 Preclinical Studies

The biologically active Tat protein has been tested in mice (149) and in monkeys (12) to evaluate the safety and the immunogenicity. The results demonstrated that vaccination with the Tat protein, at different doses and administration routes, is not toxic, since no signs of both local or systemic toxicity were ever observed, and it is immunogenic, since it elicits both humoral and cell-

mediated Tat-specific immune responses (Caselli, 1999; Cafaro, 1999, 2000 and 2001). Similar data were obtained by vaccinating animals with tat DNA (Cafaro, 1999, 2000 and 2001).

Comparable data on safety, immunogenicity and efficacy of Tat vaccination have been obtained in other laboratories. DNA vaccination with Tat, Rev and Nef has also been investigated in mice (Hinkula, 1997) providing evidence of safety and immunogenicity of these HIV-1 regulatory genes. Finally, protection from homologous challenge with a pathogenic SIV has been demonstrated in monkeys vaccinated with viral vectors expressing Tat and Rev of SIV (Osterhaus, 1999).

Unpublished data obtained from several Tat-based studies (immunization with Tat protein, DNA, adenovirus vector or microparticles-protein) on 112 Rhesus Monkeys (67 vaccinated and 45 controls) indicated that the 10 MID_{50} with the SHIV89.6P_{cy243} appeared to be the best challenge dose to assess protective immunity by Tat-based vaccines. Monkeys challenged with 10 $MID_{50,}$ had a higher probability to be aviremic as compared to 15-20 MID_{50}. In particular only for 10 MID_{50} challenge dose an association was observed between vaccination and the virological outcome both in acute phase (aviremic versus infected) and in chronic phase (aviremic versus controllers versus viremic). In acute phase vaccination was associated with lower CD4 decline as compared to controls, but this result was confirmed only for 10 MID_{50}.

In conclusion, results from pre-clinical trials indicate that vaccination with Tat is safe, immunogenic and is effective in controlling HIV infection and disease progression. Since Tat is highly conserved among the different virus clades Tat may represent an optimal target for AIDS vaccine development for both preventive and therapeutic applications in different geographical areas of the world.

1.4 Clinical Studies

On the basis of the pre-clinical studies, Phase I preventive and therapeutic trials have been sponsored by ISS and conducted in 4 clinical centers in Italy [San Raffaele Hospital (Milan), San Gallicano Hospital (Rome), Policlinico Umberto I (Rome), Spallanzani Hospital (Rome)]. These studies were directed at evaluating the safety profile (as a primary end-point) and the immunogenicity (as a secondary end-point) of the recombinant, biologically active HIV-1 Tat protein vaccine in healthy, immunologically competent adult volunteers without identifiable risk of HIV-1 infection (preventive protocol, ISS P-001), and in HIV-1 infected adult volunteers with mild immune deficiency (Clinical category A according to CDC) characterized by $CD4^+$ T cell counts ≥ 400/µl and levels of plasma viremia ≤ 50,000 copies/ml (therapeutic protocol, ISS T-001).

Both studies were randomized, placebo-controlled, and double-blinded. Volunteers were randomized to one of the two treatment arms, according to the two preparations and routes of administration, and blinded to dosage group. Specifically, in arm A, Tat protein was given subcutaneously (sc) in association with the Alum adjuvant in three dosage groups (7.5 µg, 15 µg or 30 µg) at weeks 0, 4, 8, 12, 16; three groups of volunteers were randomized to receive the vaccine and one group of volunteers to receive Alum plus the vaccine formulation buffer as placebo. In arm B, Tat protein was administered intradermally (id) without adjuvant in three dosage groups (7.5 µg, 15 µg or 30 µg) at weeks 0, 4, 8, 12, 16; three groups of volunteers were randomized to receive the vaccine and one group of volunteers to receive the vaccine formulation buffer as placebo.
In addition to the clinical protocols, a psychological protocol was also implemented to the aim of providing support for enrolment and promoting the adherence to the study, ensure the identification of major psychotic disorders, and identify psychosocial profiles in need of additional psychological

support during the most critical steps of the trial (exclusion, enrolment, occurrence of adverse events, drop out). The psychological protocol was also aimed at evaluating the motivation, anxiety, quality of life and social impact experienced by the trial participants.

For the preventive protocol, 20 volunteers were randomized as follows:

Arm A - Tat + Alum, sc	7.5 µg: 3 subjects<br>15 µg: 3 subjects<br>30 µg: 2 subjects<br>placebo: 2 subjects
Arm B - Tat, id	7.5 µg: 2 subjects<br>15 µg: 3 subjects<br>30 µg: 2 subjects<br>placebo: 3 subjects

Demographic and baseline assessment of the safety population are summarized in Table 1. There were no notable differences in the characteristics of each of the treatment groups. The majority of subjects were "male" and all were Caucasian.

Table 1. Demographic Characteristics: ISS P-001 Safety Population

	Treatment Arm A				Treatment Arm B				
	**7.5µg**	**15µg**	**30µg**	**Plb**	**7.5µg**	**15µg**	**30µg**	**Plb**	**Overall**
N	3	3	2	2	2	3	2	3	20
**Age at time of consent (years)**									
Median	42	40	41	36	21	46	32	37	38
Min	36	35	38	32	20	36	28	23	20
Max	49	50	43	39	21	50	36	49	50
**Weight (kg)**									
Median	78	85	91	68	64	76	58	60	69
Min	54	67	89	56	62	67	58	52	52
Max	95	109	92	80	66	89	59	70	109
**Height (cm)**									
Median	176	180	183	174	177	172	167	166	174
Min	165	174	178	170	171	166	160	164	160
Max	180	185	188	177	183	177	174	170	188
**Gender, n (%)**									
Male	2 (66.7)	3 (100.0)	2 (100.0)	2 (100.0)	2 (100.0)	3 (100.0)	1 (50.0)	2 (66.7)	17 (85.0)
Female	1 (33.3)	0 (0.0)	0 (0.0)	0 (0.0)	0 (0.0)	0 (0.0)	1 (50.0)	1 (33.3)	3 (15.0)
**Race, n (%)**									
Caucasian	3 (100.0)	3 (100.0)	2 (100.0)	2 (100.0)	2 (100.0)	3 (100.0)	2 (100.0)	3 (100.0)	20 (100.0)

N = total number of subjects per treatment group; n = number of subjects

For the therapeutic protocol, 27 volunteers were randomized as follows:

Arm A - Tat + Alum, sc
- 7.5 µg: 4 subjects
- 15 µg: 3 subjects
- 30 µg: 4 subjects
- placebo: 4 subjects

Arm B - Tat, id
- 7.5 µg: 4 subjects
- 15 µg: 2 subjects
- 30 µg: 3 subjects
- placebo: 3 subjects

Demographic and baseline assessment of the safety population from therapeutic protocol are summarized in Table 2. There were no differences in the characteristics of each of the treatment groups.

Table 2. Demographic Characteristics: ISS T-001 Safety Population

	Arm A				Arm B				
	**7.5µg**	**15µg**	**30µg**	**Plb**	**7.5µg**	**15µg**	**30µg**	**Plb**	**Overall**
N	4	3	4	4	4	2	3	3	27
**Age at time of consent (years)**									
Median	41	33	33	38	35	35	39	38	38
Min	38	30	28	32	24	31	30	29	24
Max	50	40	38	45	43	39	43	39	50
**Weight (kg)**									
Median	56	72	62	77	70	58	61	73	67
Min	55	66	50	61	68	50	57	62	50
Max	86	76	63	89	98	67	64	79	98
**Height (cm)**									
Median	167	173	164	178	171	169	157	174	172
Min	144	172	155	158	165	163	152	173	144
Max	180	181	170	180	175	174	171	175	181
**Gender, n (%)**									
Male	1 (25.0)	2 (66.7)	1 (25.0)	4 (100.0)	4 (100.0)	1 (50.0)	2 (66.7)	3 (100.0)	18 (66.7)
Female	3 (75.0)	1 (33.3)	3 (75.0)	0 (0.0)	0 (0.0)	1 (50.0)	1 (33.3)	0 (0.0)	9 (33.3)
**Race, n (%)**									
Caucasian	4 (100.0)	3 (100.0)	4 (100.0)	4 (100.0)	4 (100.0)	2 (100.0)	3 (100.0)	3 (100.0)	27 (100.0)

N = total number of patients per treatment group; n = number of patients; Plb = placebo

The studies were conducted in accordance with the ethical principles that have their origin in the Declaration of Helsinki amended by the 52nd General Assembly in October 2000 with the Note of Clarification on Paragraph 29 added by the WMA General Assembly, Washington 2002 and consistently with Good Clinical Practice (GCP) and applicable regulatory requirements.
The safety and immunogenicity results obtained after immunization with the recombinant biologically active HIV-1 Tat vaccine in HIV-1 infected adult volunteers (therapeutic protocol, ISS T-001) and HIV-1 uninfected adult volunteers (preventive protocol, ISS P-001) are described in the following sections.

1.5 Safety

The primary end-point of the Phase I preventive and therapeutic vaccine trials was to qualify the active Tat protein as safe for its further evaluation in Phase II clinical trials. Safety was evaluated by monitoring the volunteers for local and systemic adverse reactions during the course of the trials. Clinical evaluation of safety also included monitoring of hematological (including coagulation assessment), biochemical (including liver and kidney functional parameters) and immunological parameters (including CD4, CD8, CD3 T cells, NK, B cells and monocytes) (Table 3). The safety profile assessment also included the evaluation of plasma viremia levels.

Table 3. Laboratory Safety

**CLINICAL CHEMISTRY**	**Sodium<br>Potassium<br>Calcium<br>Total Protein<br>Albumin**
**HEMATOLOGY**	**RBC<br>HB<br>HCT<br>MCV<br>Platelets<br>WBC<br>Neutrophils<br>Lymphocytes<br>Monocytes<br>Eosinophils<br>Basophils**
**COAGULATION ASSESSMENT**	**Thrombine time<br>Prothrombine time<br>APTT**
**HEPATIC FUNCTION**	**Total bilirubin<br>Alkaline Phosphatase<br>AST<br>ALT<br>γGT**
**RENAL FUNCTION**	**Urea<br>Creatinine**
**LYMPHOCYTE PHENOTYPE**	**CD3+ (T cells)<br>CD3+ CD4+ (T cells)<br>CD3+ CD8+ (T cells)<br>CD3- CD56+ CD16+ (NK cells)<br>CD19+ (B cells)**

Assessment of safety was performed at baseline and at several time points during the study. A detailed calendar of these evaluations is reported in Table 4 (A: Calendar for protocol ISS P-001; B: calendar for protocol ISS T-001). Overall, no clinically significant alterations in laboratory values have been identified in any treatment group. AEs were coded according to the MedDRA dictionary and grouped in accordance to the MedDRA System Organ Classes (SOC).
The AEs were classified according to the relationship to drug (RD) and to the grade of severity. In particular, the relationship to the immunization was defined as follows: RD 1: Unrelated, the AE is clearly not related to the investigational agent; RD 2: Unlikely, the AE is doubtfully related to the investigational agent; RD 3: Possible, the AE may be related to the investigational agent; RD 4:

Probable, the AE is likely related to the investigational agent; RD 5: Definite, the AE is clearly related to the investigational agent.
An independent, "ad hoc" safety monitoring board (DSMB) periodically evaluated all safety documentation including the frequency and characteristics of AEs. Safety monitoring confirmed that the vaccine based on the recombinant Tat protein is safe and well tolerated. The most frequent AEs were mild and did not appear to be clearly dose related, including injection site reactions, asthenia, fever, headache and transient blood disorders (mainly leukocytosis and neutrophilia) as detailed below.

Table 4. Safety evaluations performed during the treatment phase (24 weeks)

A: protocol ISS P-001

**WEEK**	**Pre**		**0**			**4**			**8**			**12**			**16**			**24**
**DAY**	**-28 -7**		**0**	**1**	**7**	**28**	**29**	**35**	**56**	**57**	**63**	**84**	**85**	**91**	**112**	**113**	**119**	**168**
**STUDY VISIT**	01	02	03	04	05	06	07	08	09	10	11	12	13	14	15	16	17	18
Immunization			X			X			X			X			X			
Hematology	X	X	X	X	X	X	X	X	X	X	X	X	X	X	X	X	X	X
Coagulation	X		X	X	X	X	X	X	X	X	X	X	X	X	X	X	X	X
Blood chemistry	X		X	X	X	X	X	X	X	X	X	X	X	X	X	X	X	X
Urine dipstick	X		X	X	X	X	X	X	X	X	X	X	X	X	X	X	X	X
$CD4^+$ T cell count	X	X	X	X	X	X	X	X	X	X	X	X	X	X	X	X	X	X
Lymphocyte phenotype	X					X			X			X			X			X
Assessment of AEs				X	X	X	X	X	X	X	X	X	X	X	X	X	X	X

B: protocol ISS T-001

**WEEK**	**Pre**		**0**			**4**			**8**			**12**			**16**			**24**
**DAY**	**-28 -7**		**0**	**1**	**7**	**28**	**29**	**35**	**56**	**57**	**63**	**84**	**85**	**91**	**112**	**113**	**119**	**168**
**STUDY VISIT**	01	02	03	04	05	06	07	08	09	10	11	12	13	14	15	16	17	18
Immunization			X			X			X			X			X			
Hematology	X	X	X	X	X	X	X	X	X	X	X	X	X	X	X	X	X	X
Coagulation	X		X	X	X	X	X	X	X	X	X	X	X	X	X	X	X	X
Blood chemistry	X		X	X	X	X	X	X	X	X	X	X	X	X	X	X	X	X
Urine dipstick	X		X	X	X	X	X	X	X	X	X	X	X	X	X	X	X	X
$CD4^+$ T cell count	X	X	X	X	X	X	X	X	X	X	X	X	X	X	X	X	X	X
Lymphocyte phenotype	X					X			X			X			X			X
Assessment of AEs				X	X	X	X	X	X	X	X	X	X	X	X	X	X	X

1.5.1 Preventive Protocol (ISS P-001)

Non Serious Adverse Events

A total of 166 local AEs were reported for the preventive protocol (ISS P-001) (Table 5). No events with a grade of severity 3 were reported and most of the events (92.2%) were mild (grade1). The

mean number of local AEs per subject is indicated in Figure 3. No significant differences were observed in the number of the AE reported in the dosage groups.

Table 5. Total Local Adverse Events reported for protocol ISS P-001

Arm A																
**Tat 7.5 µg (N=3)**				**Tat 15 µg (N=3)**				**Tat 30 µg (N=2)**				**Pla (N=2)**				**Total AEs (N=10)**
	**Grade of Severity**				**Grade of Severity**				**Grade of Severity**				**Grade of Severity**			
**Total**	**1**	**2**	**3**	**Total**	**1**	**2**	**3**	**Total**	**1**	**2**	**3**	**Total**	**1**	**2**	**3**	
34	31	3	0	30	28	2	0	22	21	1	0	1	1	0	0	87
**Arm B**																
**Tat 7.5 µg (N=2)**				**Tat 15 µg (N=3)**				**Tat 30 µg (N=2)**				**Pla (N=3)**				**Total AEs (N=10)**
	**Grade of Severity**				**Grade of Severity**				**Grade of Severity**				**Grade of Severity**			
**Total**	**1**	**2**	**3**	**Total**	**1**	**2**	**3**	**Total**	**1**	**2**	**3**	**Total**	**1**	**2**	**3**	
18	17	1	0	27	25	2	0	27	27	0	0	7	3	4	0	79

Figure 3. Mean number of total Local AEs/subject (protocol ISS P-001)

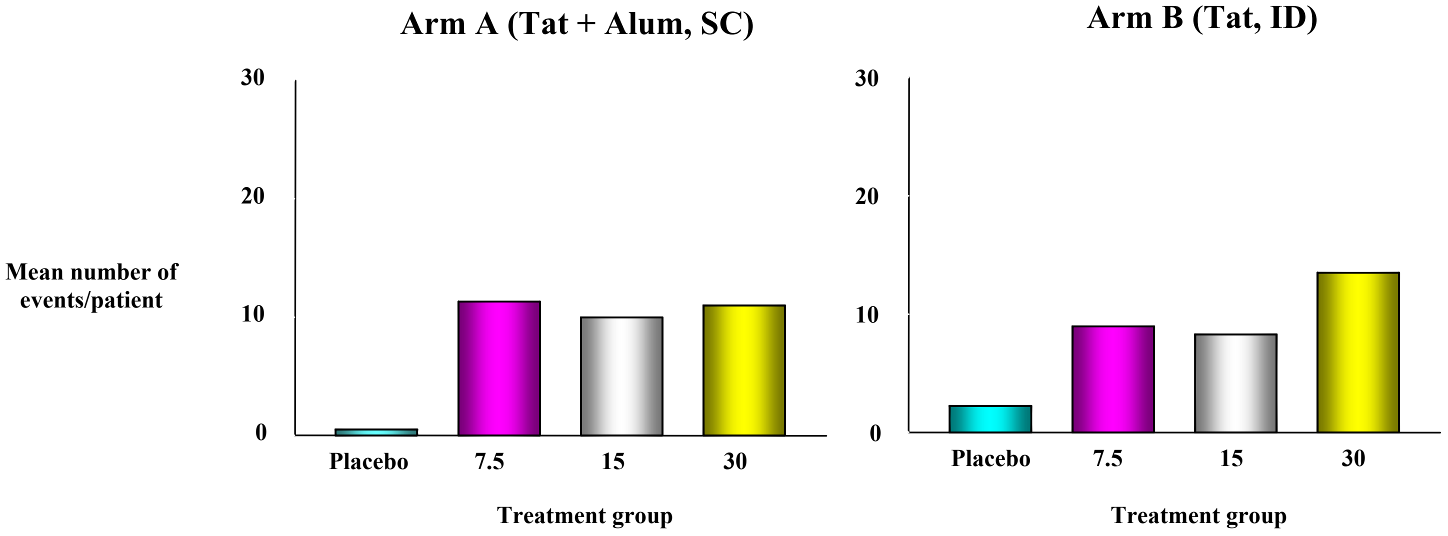

A total of 200 non-local AEs were reported (Table 6). For one event severity was not recorded on CRF and therefore a grade of severity =3 was attributed. No other events with a grade of severity > 2 were reported and most of the events (87%) were mild (grade 1). The mean number of non-local events is indicated in Figure 4. No statistical significant differences were observed among treatment groups.

Table 6. Total Non-local Adverse Events reported for protocol ISS P-001

Arm A																
Tat 7.5 µg (N=3)				Tat 15 µg (N=3)				Tat 30 µg (N=2)				Pla (N=2)				Total AEs (N=10)
	Grade of Severity				Grade of Severity				Grade of Severity				Grade of Severity			
Total	1	2	3	Total	1	2	3	Total	1	2	3	Total	1	2	3	
37	33	3	1	33	31	2	0	42	34	8	0	10	10	0	0	122
**Arm B**																
Tat 7.5 µg (N=2)				Tat 15 µg (N=3)				Tat 30 µg (N=2)				Pla (N=3)				Total AEs (N=10)
	Grade of Severity				Grade of Severity				Grade of Severity				Grade of Severity			
Total	1	2	3	Total	1	2	3	Total	1	2	3	Total	1	2	3	
20	18	2	0	30	24	6	0	12	11	1	0	16	13	3	0	78

Figure 4. Mean number of total non local AEs/subject (protocol ISS P-001)

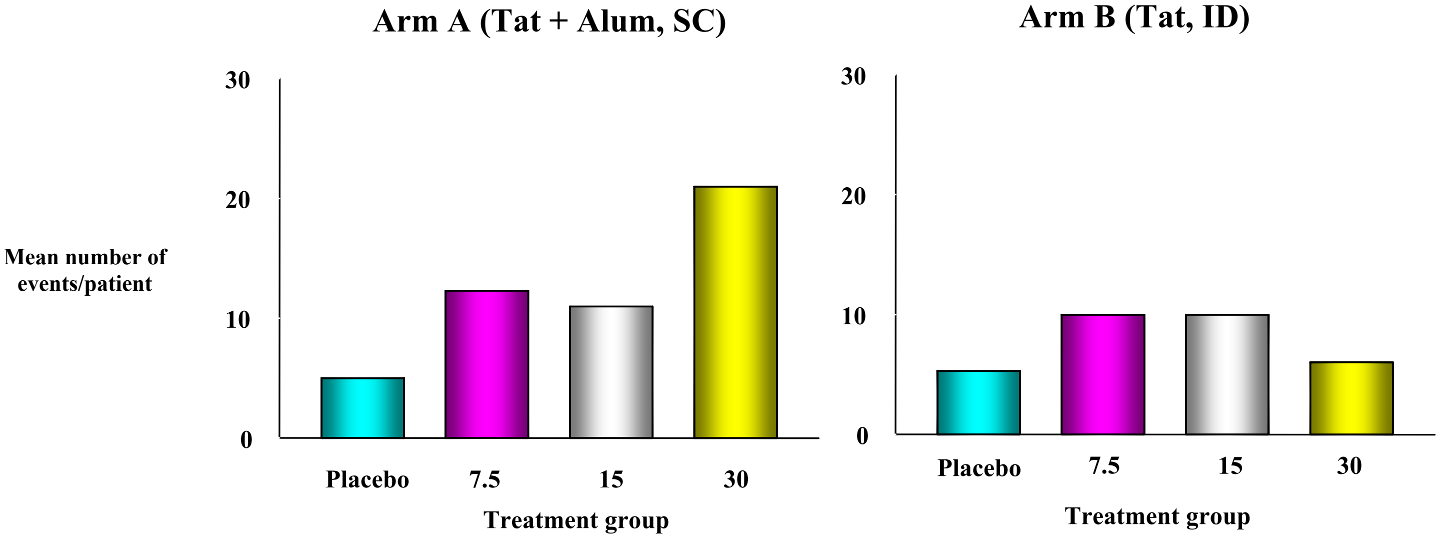

AEs which were reported as “possibly”, “likely” or “clearly” related to the immunization are described according to the MedDRA SOC and preferred terms in Table 7. In particular, 157 local AEs and 137 non local AEs were reported (Arm A: 78 local AEs, 96 non local AEs; Arm B: 79 local AEs, 41 non local AEs); for one event (fever) in Arm A the severity was not indicated by the investigator and therefore, a grade of severity = 3 was attributed. No other events were recorded. No events with a grade of severity > 2 were reported in Arm B. Injection site related AEs were reported in the volunteers immunized with the Tat protein, with no relationship with the dosage. These events were reported, at lower frequency, also in the placebo groups. Systemic AEs events were reported with all dosage groups, and were mostly represented by transient blood disorders (mainly leucocytosis with neutrophilia and transient lymphocytopenia), asthenia, fever, myalgia. No significant differences were observed among the three vaccine dosages.

The average number of AEs per volunteer, according to the MedDRA SOC, is reported in Table 8.

Table 7. Number of volunteers reporting AEs "possibly", "likely" or "clearly" related to the immunization in Arm A (A) and in Arm B (B) for protocol ISS P-001

A.

	**Tat 7.5 µg (N=3)**					**Tat 15 µg (N=3)**					**Tat 30 µg (N=2)**					**Placebo (N=2)**					**Total (N=10)**
**Body System/Apparatus**	**N**	**%**	**1**	**2**	**3**	**N**	**%**	**1**	**2**	**3**	**N**	**%**	**1**	**2**	**3**	**N**	**%**	**1**	**2**	**3**	**N**
**Local AEs**																					
Local administration site disorders	3	100%	2	1	0	3	100%	1	2	0	2	100%	1	1	0	1	50%	1	0	0	**9**
**Non Local AEs**																					
General disorders	2	67%	1	0	1	3	100%	3	0	0	2	100%	0	2	0	0	0%	0	0	0	**7**
Blood and lymphatic system disorders	2	67%	2	0	0	2	67%	1	1	0	1	50%	0	1	0	0	0%	0	0	0	**5**
Nervous System disorders	1	33%	1	0	0	2	67%	2	0	0	1	50%	0	1	0	1	50%	1	0	0	**5**
Skeletal muscle and connective tissue disorders	1	33%	0	1	0	1	33%	1	0	0	1	50%	1	0	0	0	0%	0	0	0	**3**
Gastrointestinal disorders	0	0%	0	0	0	2	67%	2	0	0	0	0%	0	0	0	0	0%	0	0	0	**2**
Cardio-vascular disorders	0	0%	0	0	0	0	0%	0	0	0	0	0%	0	0	0	1	50%	1	0	0	**1**
Ear and labyrinth disorders	0	0%	0	0	0	1	33%	1	0	0	0	0%	0	0	0	0	0%	0	0	0	**1**
Eye disorders	0	0%	0	0	0	1	33%	1	0	0	0	0%	0	0	0	0	0%	0	0	0	**1**
Kidney and urinary disorders	1	33%	0	1	0	0	0%	0	0	0	0	0%	0	0	0	0	0%	0	0	0	**1**
Psychiatric disorders	0	0%	0	0	0	1	33%	1	0	0	0	0%	0	0	0	0	0%	0	0	0	**1**
Skin and subcutaneous tissue disorders	0	0%	0	0	0	1	33%	0	1	0	0	0%	0	0	0	0	0%	0	0	0	**1**

N: number of volunteers; 1: grade of severity 1 (mild); 2: grade of severity 2 (moderate); grade 3: grade of severity 3 (severe).

B.

Body System/Apparatus	Tat 7.5 µg (N=2)					Tat 15 µg (N=3)					Tat 30 µg (N=2)					Placebo (N=3)					Total (N=10)
	**N**	**%**	**1**	**2**	**3**	**N**	**%**	**1**	**2**	**3**	**N**	**%**	**1**	**2**	**3**	**N**	**%**	**1**	**2**	**3**	**N**
**Local AEs**																					
Local administration site disorders	2	100%	1	1	0	3	100%	2	1	0	2	100%	2	0	0	2	67%	1	1	0	**9**
**Non Local AEs**																					
General disorders	0	0%	0	0	0	2	67%	2	0	0	2	100%	1	1	0	1	33%	1	0	0	**5**
Blood and lymphatic system disorders	1	50%	1	0	0	2	67%	1	1	0	0	0%	0	0	0	0	0%	0	0	0	**3**
Nervous System disorders	2	100%	2	0	0	1	33%	1	0	0	0	0%	0	0	0	0	0%	0	0	0	**3**
Cardio-vascular disorders	0	0%	0	0	0	1	33%	1	0	0	1	50%	1	0	0	0	0%	0	0	0	**2**
Skeletal muscle and connective tissue disorders	0	0%	0	0	0	1	33%	0	1	0	0	0%	0	0	0	1	33%	0	1	0	**2**
Gastrointestinal disorders	0	0%	0	0	0	1	33%	0	1	0	0	0%	0	0	0	0	0%	0	0	0	**1**
Skin and subcutaneous tissue disorders	0	0%	0	0	0	1	33%	1	0	0	0	0%	0	0	0	0	0%	0	0	0	**1**

N: number of volunteers. 1: grade of severity 1 (mild); 2: grade of severity 2 (moderate); grade 3: grade of severity 3 (severe).

Table 8. Average number of AEs per volunteer "possibly", "likely" or "clearly" related to the immunization for protocol ISS P-001

	Arm A				Arm B				TOTAL
	**7.5 µg**	**15 µg**	**30 µg**	**Placebo**	**7.5 µg**	**15 µg**	**30 µg**	**Placebo**	
**NUMBER OF VOLUNTEERS**	3	3	2	2	2	3	2	3	20
Local administration site disorders	9.3	9.0	11.0	0.5	9.0	9.0	13.5	2.3	7.9
Blood and lymphatic system disorders	5.7	4.3	9.0	0.0	4.0	2.0	0.0	0.0	3.1
General disorders	2.7	2.0	5.0	0.0	0.0	0.7	2.0	0.7	1.6
Nervous System Disorders	1.0	1.0	1.0	0.5	1.5	1.0	0.0	0.0	0.8
Skeletal muscle and connective tissue disorders	1.0	0.3	1.0	0.0	0.0	1.7	0.0	1.7	0.8
Cardio-vascular disorders	0.0	0.0	0.0	0.5	0.0	0.3	0.5	0.0	0.2
Gastrointestinal disorders	0.0	1.0	0.0	0.0	0.0	0.3	0.0	0.0	0.2
Ear and labyrinth disorders	0.0	0.3	0.0	0.0	0.0	0.0	0.0	0.0	0.1
Eye disorders	0.0	0.3	0.0	0.0	0.0	0.0	0.0	0.0	0.1
Kidney and urinary disorders	0.3	0.0	0.0	0.0	0.0	0.0	0.0	0.0	0.1
Psychiatric disorders	0.0	0.3	0.0	0.0	0.0	0.0	0.0	0.0	0.1
Skin and subcutaneous tissue disorders	0.0	0.3	0.0	0.0	0.0	0.3	0.0	0.0	0.1
TOTAL	20.0	19.0	27.0	1.5	14.5	15.3	16.0	4.7	14.8

Fever was a frequently reported AE after immunization. The increase in body temperature occurred within 24 hours after injection and lasted 12-24 hours. As indicated in Table 9, this event occurred more frequently (13/18 events) in the individuals vaccinated subcutaneously (in association with the Alum adjuvant). In particular, the event was reported in 67% of the volunteers immunized with 7.5 µg of Tat, and in 100% of the volunteers immunized with 15 and 30 µg of Tat. Five events were reported among the groups vaccinated intradermally (in the absence of adjuvant), in the volunteers immunized with 15 µg (67%) and 30 µg (100%) of Tat. No events of fever were reported with a grade of severity > 3. For one of these events (Arm A), the severity was not indicated by the investigator and therefore, a grade of severity = 3 was attributed. Events of fever were not observed in the placebo groups.

Table 9. Summary of the events of fever "possibly", "likely" or "clearly" related to the immunization, reported for protocol ISS P-001

**Treatment Groups**	**FEVER**		
	**Arm A (N=10)**	**Arm B (N=10)**	**Total (N= 20)**
**Placebo**			
Number of volunteers reporting the event (% of total)	0/2 (0%)	0/3 (0%)	0/5 (0%)
Number of events	0	0	0
**Tat 7.5 µg**			
Number of volunteers reporting the event (% of total)	2/3 (67%)	0/2 (0%)	2/5 (40%)
Number of events	4	0	4
**Tat 15 µg**			
Number of volunteers reporting the event (% of total)	3/3 (100%)	2/3 (67%)	5/6 (83%)
Number of events	3	2	5
**Tat 30 µg**			
Number of volunteers reporting the event (% of total)	2/2 (100%)	2/2 (100%)	4/4 (100%)
Number of events	6	3	9
**Total events**	**13**	**5**	**18**

N: number of volunteers.

Transient changes in hematologic parameters are known to occur after exposure to vaccines and are generally not associated with clinical sequelae. Within this trial, blood disorders were mostly represented by transient leukocytosis with neutrophilia and lymphocytopenia, which occurred within 24 hours after injection and did not appear to be dose related. In most cases, normal hematological values were restored at the following time point, 7 days after immunization. As indicated in Table 10, these events occurred mostly (48/62 events) in the individuals vaccinated subcutaneously (in 67% of the volunteers immunized with 7.5 and 15 µg of Tat, and in 50% of the volunteers immunized with 30 µg of Tat). Fourteen events were reported among the groups vaccinated intradermally (in 100% of the volunteers immunized with 7.5 µg and in 67% of the volunteers immunized with 15 µg of Tat). No events were reported in the placebo groups.

Table 10. Summary of blood disorders events "possibly", "likely" or "clearly" related to the immunization, reported for protocol ISS P-001

Treatment groups	BLOOD DISORDERS		
	Arm A (N=10)	Arm B (N=10)	Total (N= 20)
**Placebo**			
Number of volunteers reporting the events (% of total)	0/2 (0%)	0/3 (0%)	0/5 (0%)
Number of events	0	0	0
**Tat 7.5 μg**			
Number of volunteers reporting the events (% of total)	2/3 (67%)	2/2 (100%)	4/5 (80%)
Number of events	17	8	25
**Tat 15 μg**			
Number of volunteers reporting the events (% of total)	2/3 (67%)	2/3 (67%)	4/6 (67%)
Number of events	13	6	19
**Tat 30 μg**			
Number of volunteers reporting the events (% of total)	1/2 (50%)	0/2 (0%)	1/4 (25%)
Number of events	18	0	18
**Total events**	**48**	**14**	**62**

N: number of volunteers. No events with a grade of severity > 2 were reported.

Assessment of safety included an extensive monitoring of hematological, biochemical and immunological parameters. Laboratory parameters were collected at the day of immunization and 24 hours and 7 days after each injection. Overall, no clinically significant alterations of laboratory values have been identified in any treatment group of the protocol ISS P-001. An increase of number of WBC and neutrophils (which mostly accounted for the WBC increase) was observed in all the individuals 24 hours after immunization with the Tat vaccine. Normal WBC values were completely restored at the following evaluation, 7 days after immunization. Due to the transient nature of the variations observed, and the well known association with the administration of vaccines against infectious or allergic agents, the general consensus of the DSMB was that these events were not clinically significant.

Serious Adverse Events

No SAE were reported for subjects immunized with the Tat protein.

1.5.2 Therapeutic Protocol (ISS T-001)

Non Serious Adverse Events

A total of 266 local AEs were reported as shown in Table 11. Most of the events (93.6%) were mild (grade 1); only one event was defined as grade 3 of severity. The mean number of local AEs per patient is indicated in Figure 5.

Table 11. Number of total local AEs reported by dosage group and grade of severity for protocol ISS T-001

Arm A																
**Tat 7.5 µg (N=4)**				**Tat 15 µg (N=3)**				**Tat 30 µg (N=4)**				**Pla (N=4)**				**Total AEs (N=15)**
	**Grade of Severity**				**Grade of Severity**				**Grade of Severity**				**Grade of Severity**			
**Total**	**1**	**2**	**3**	**Total**	**1**	**2**	**3**	**Total**	**1**	**2**	**3**	**Total**	**1**	**2**	**3**	
50	44	5	1	20	17	3	0	56	52	4	0	8	6	2	0	**134**
**Arm B**																
**Tat 7.5 µg (N=4)**				**Tat 15 µg (N=2)**				**Tat 30 µg (N=3)**				**Pla (N=3)**				**Total AEs (N=12)**
	**Grade of Severity**				**Grade of Severity**				**Grade of Severity**				**Grade of Severity**			
**Total**	**1**	**2**	**3**	**Total**	**1**	**2**	**3**	**Total**	**1**	**2**	**3**	**Total**	**1**	**2**	**3**	
53	53	0	0	21	21	0	0	54	53	1	0	4	3	1	0	**132**

Figure 5. Mean number of total local AEs/ subject (protocol ISS T-001)

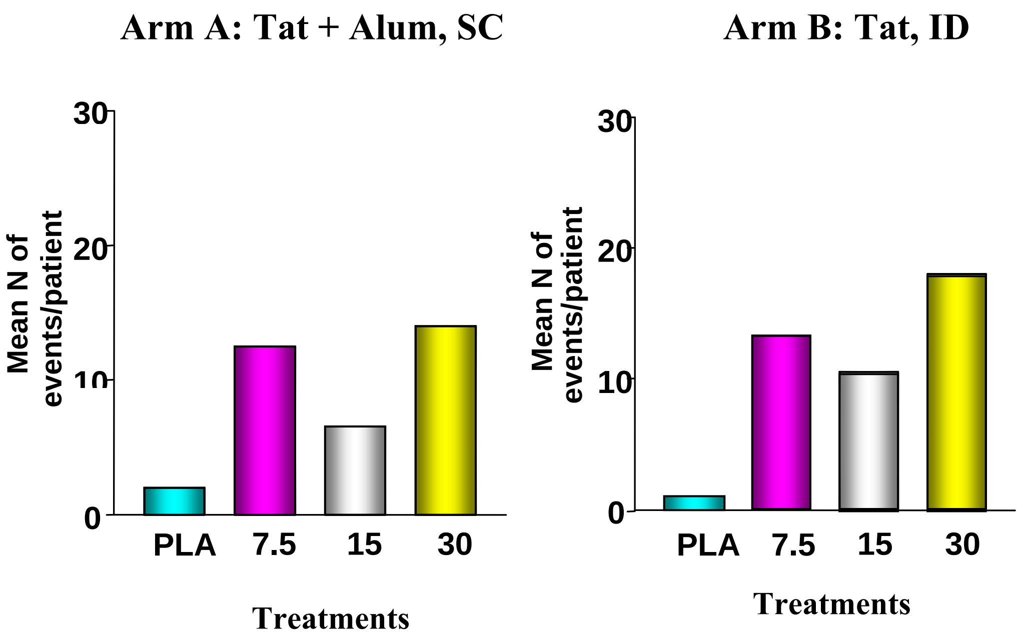

A total of 295 non-local AEs were reported (Table 12). Most of the events (78%) were mild; only one event was defined as grade 3 of severity. The mean number of non-local AEs per patient is indicated in Figure 6.

Table 12. Number of total non-local AEs reported by dosage group and grade of severity for protocol ISS T-001

Arm A																
Tat 7.5 µg (N=4)				Tat 15 µg (N=3)				Tat 30 µg (N=4)				Pla (N=4)				Total AEs (N=15)
	Grade of Severity				Grade of Severity				Grade of Severity				Grade of Severity			
Total	1	2	3	Total	1	2	3	Total	1	2	3	Total	1	2	3	
26	22	4	0	34	24	10	0	72	49	22	1	47	40	7	0	179
**Arm B**																
Tat 7.5 µg (N=4)				Tat 15 µg (N=2)				Tat 30 µg (N=3)				Pla (N=3)				Total AEs (N=12)
	Grade of Severity				Grade of Severity				Grade of Severity				Grade of Severity			
Total	1	2	3	Total	1	2	3	Total	1	2	3	Total	1	2	3	
24	21	3	0	30	22	8	0	44	37	7	0	18	15	3	0	116

N= number of volunteers

Figure 6. Mean number of total non-local AEs/subject (protocol ISS T-001)

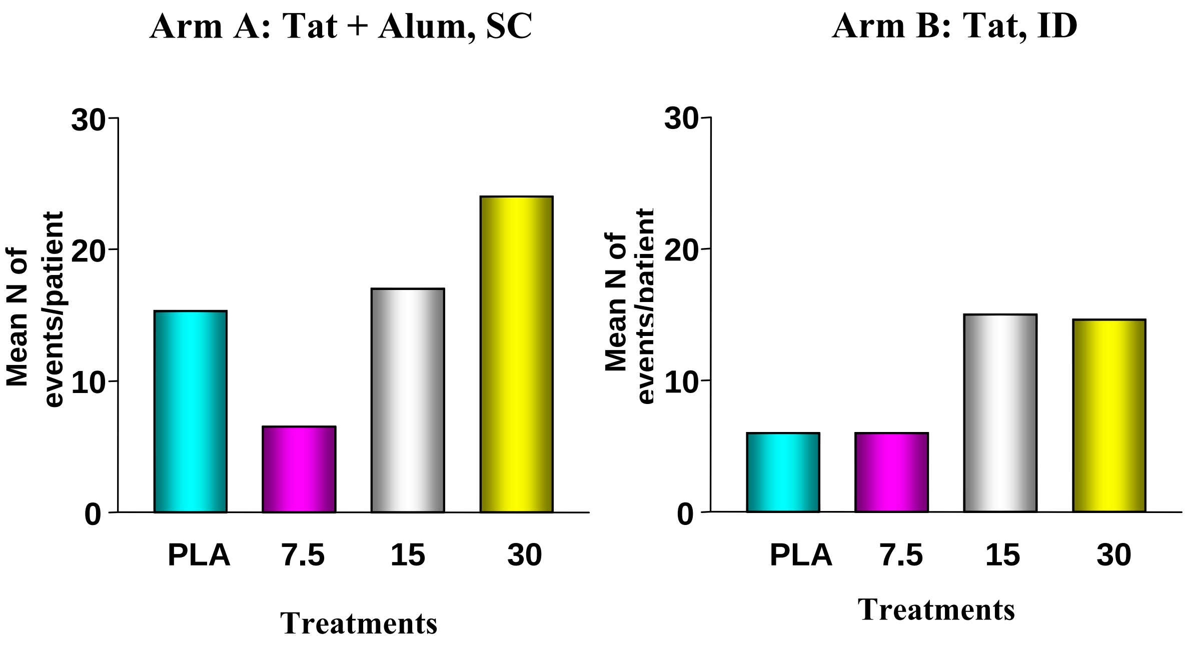

Injection site related AEs were reported in the volunteers immunized with the Tat protein, with no relationship with the dosage. These events were reported, at lower frequency, also in the placebo groups. Systemic AEs were reported with all dosage groups, and were mostly represented by asthenia, fever, headache, transient blood disorders (mainly leukocytosis and neutrophilia).

The number of volunteers reporting AEs which were "possibly", "likely" or "clearly" related to treatment, classified according to the MedDRA SOC, is reported in Table 13. Local, administration site disorders were reported in 24/27 volunteers, including 3/4 volunteers (Arm A) and 2/3 volunteers (Arm B) randomized in the placebo groups. Among non local AEs, 15/27 volunteers (including 1 placebo in Arm A) reported general disorders; 12/27 volunteers reported blood and lymphatic system disorders (including 2 placebos in Arm A and 1 placebo in Arm B).

The average number of AEs per volunteer, according to the MedDRA SOC, is reported in Table 14.

Table 13. Number of volunteers reporting AEs "possibly", "likely" or "clearly" related to the immunization in Arm A (A) and in Arm B (B) for protocol ISS T-001

A.

Body System/Apparatus	Tat 7.5 µg (N=4)					Tat 15 µg (N=3)					Tat 30 µg (N=4)					Placebo (N=4)					Total (N=15)
	N	%	1	2	3	N	%	1	2	3	N	%	1	2	3	N	%	1	2	3	N
**Local AEs**																					
Local administration site disorders	4	100%	0	3	1	2	67%	0	2	0	4	100%	1	3	0	3	75%	1	2	0	**13**
**Non Local AEs**																					
General disorders	3	75%	3	0	0	2	67%	0	2	0	4	100%	0	3	1	1	25%	0	1	0	**10**
Blood and lymphatic system disorders	1	25%	0	1	0	1	33%	1	0	0	2	50%	1	1	0	2	50%	1	1	0	**6**
Skeletal muscle and connective tissue disorders	0	0%	0	0	0	2	67%	1	1	0	3	75%	2	1	0	1	25%	1	0	0	**6**
Nervous System Disorders	1	25%	1	0	0	1	33%	1	0	0	1	25%	1	0	0	0	0%	0	0	0	**3**
Psychiatric disorders	0	0%	0	0	0	1	33%	0	1	0	0	0%	0	0	0	1	25%	1	0	0	**2**
Gastrointestinal disorders	0	0%	0	0	0	1	33%	1	0	0	1	25%	1	0	0	0	0%	0	0	0	**2**
Ear and labyrinth disorders	0	0%	0	0	0	1	33%	1	0	0	0	0%	0	0	0	0	0%	0	0	0	**1**
Skin and subcutaneous tissue disorders	0	0%	0	0	0	0	0%	0	0	0	0	0%	0	0	0	1	25%	0	1	0	**1**

N: number of volunteers; 1: grade of severity 1 (mild); 2: grade of severity 2 (moderate); 3: grade of severity 3 (severe).

B.

**Body System/Apparatus**	**Tat 7.5 µg (N=4)**					**Tat 15 µg (N=2)**					**Tat 30 µg (N=3)**					**Placebo (N=3)**					**Total (N=12)**
	**N**	**%**	**1**	**2**	**3**	**N**	**%**	**1**	**2**	**3**	**N**	**%**	**1**	**2**	**3**	**N**	**%**	**1**	**2**	**3**	**N**
**Local AEs**																					
Local administration site disorders	4	100%	3	1	0	2	100%	0	2	0	3	100%	1	2	0	2	67%	2	0	0	**11**
**Non Local AEs**																					
Blood and lymphatic system disorders	3	75%	3	0	0	2	100%	2	0	0	0	0%	0	0	0	1	33%	1	0	0	**6**
General disorders	2	50%	1	1	0	1	50%	0	1	0	2	67%	2	0	0	0	0%	0	0	0	**5**
Nervous System disorders	0	0%	0	0	0	1	50%	1	0	0	2	67%	2	0	0	0	0%	0	0	0	**3**
Infection and infestation	0	0%	0	0	0	1	50%	1	0	0	0	0%	0	0	0	0	0%	0	0	0	**1**
Skeletal muscle and connective tissue disorders	0	0%	0	0	0	1	50%	0	1	0	0	0%	0	0	0	0	0%	0	0	0	**1**
Skin and subcutaneous tissue disorders	0	0%	0	0	0	1	50%	1	0	0	0	0%	0	0	0	0	0%	0	0	0	**1**
Hepato-biliary disorders	0	0%	0	0	0	0	0%	0	0	0	1	33%	1	0	0	0	0%	0	0	0	**1**

N: number of volunteers; 1: grade of severity 1 (mild); 2: grade of severity 2 (moderate); 3: grade of severity 3 (severe).

Table 14. Average number of AEs per volunteer "possibly", "likely" or "clearly" related to the immunization for protocol ISS T-001

	Arm A				Arm B				TOTAL
	**7.5 µg**	**15 µg**	**30 µg**	**Placebo**	**7.5 µg**	**15 µg**	**30 µg**	**Placebo**	
**NUMBER OF VOLUNTEERS**	4	3	4	4	4	2	3	3	27
Local administration site disorders	12,5	6,7	13,5	2,0	13,3	10,0	18,0	1,0	9,7
General disorders	2,3	3,0	7,0	1,5	0,8	6,5	3,0	0,0	2,9
Blood and lymphatic system disorders	0,3	0,7	3,0	0,8	3,0	1,0	0,0	0,3	1,2
Skeletal muscle and connective tissue disorders	0,0	2,0	2,8	0,3	0,0	1,0	0,0	0,0	0,7
Nervous System Disorders	0,3	0,3	0,3	0,0	0,0	0,5	1,3	0,0	0,3
Gastrointestinal disorders	0,0	0,7	0,5	0,0	0,0	0,0	0,0	0,0	0,1
Psychiatric disorders	0,0	0,7	0,0	0,3	0,0	0,0	0,0	0,0	0,1
Skin and subcutaneous tissue disorders	0,0	0,0	0,0	0,3	0,0	1,0	0,0	0,0	0,1
Ear and labyrinth disorders	0,0	0,3	0,0	0,0	0,0	0,0	0,0	0,0	0,0
Hepato-biliary disorders	0,0	0,0	0,0	0,0	0,0	0,0	0,3	0,0	0,0
Infection and infestation	0,0	0,0	0,0	0,0	0,0	0,5	0,0	0,0	0,0
Total	15,3	14,3	27,0	5,0	17,0	20,5	22,7	1,3	15,3

Fever was a frequently reported AE after immunization. In most cases, the increase in body temperature occurred within 24 hours after injection and lasted 12-24 hours. As indicated in Table 15, this event occurred more frequently (31/38 events) in the individuals vaccinated subcutaneously (in association with the Alum adjuvant). In particular, the event was reported in 75% of the volunteers immunized with 7.5 μg of Tat, in 33% of the volunteers immunized with 15 μg of Tat, and in 100% of the volunteers immunized with 30 μg of Tat. Seven events were reported among the groups vaccinated intradermally (in the absence of adjuvant), in 50% of the volunteers immunized with 7.5 μg and 15 μg of Tat, and in 33% of the volunteers immunized with 30 μg of Tat. Four events of fever were reported in 1/4 individuals within the placebo group of Arm A (the placebo contains the Alum adjuvant). One event of fever with a grade of severity = 3 was reported in Arm A.

Table 15. Summary of the events of fever "possibly", "likely" or "clearly" related to the immunization, reported for protocol ISS T-001

**Treatment Groups**	**FEVER**		
	**Arm A (N=15)**	**Arm B (N=12)**	**Total (N=27)**
**Placebo**			
Number of volunteers reporting the event (% of total)	1/4 (25%)	0/3 (0%)	1/7 (14%)
Number of events	4	0	4
**Tat 7.5 μg**			
Number of volunteers reporting the event (% of total)	3/4 (75%)	2/4 (50%)	5/8 (62%)
Number of events	5	2	7
**Tat 15 μg**			
Number of volunteers reporting the event (% of total)	1/3 (33%)	1/2 (50%)	2/5 (40%)
Number of events	4	3	7
**Tat 30 μg**			
Number of volunteers reporting the event (% of total)	4/4 (100%)	1/3 (33%)	5/7 (71%)
Number of events	18	2	20
**Total events**	**31**	**7**	**38**

N: number of volunteers.

Transient changes in hematologic parameters are known to occur after exposure to vaccines and are not associated with clinical sequelae. Within this trial, blood alterations were mostly represented by transient leukocytosis and neutrophilia, which occurred within 24 hours after injection. Normal hematological values were restored at the following visit, 7 days after immunization. As indicated in Table 16, 18/33 events occurred in the individuals vaccinated subcutaneously (in 25% of the volunteers immunized with 7.5 μg, in 33% of the individuals immunized with 15 μg, and in 50% of the volunteers immunized with 30 μg of Tat). Fifteen events were reported among the groups vaccinated intradermally (in 75% of the volunteers immunized with 7.5 μg and in 100% of the individual immunized with 15 μg of Tat). Four events were reported in the placebo groups, 3 events in 50% of the individuals belonging to Arm A, 1 event in 33% of the individuals belonging to Arm B.

Table 16. Summary of blood disorders events "possibly", "likely" or "clearly" related to the immunization reported for protocol ISS T-001

Treatment Groups	BLOOD DISORDERS		
	**Arm A (N=15)**	**Arm B (N=12)**	**Total (N=27)**
**Placebo**			
Number of volunteers reporting the events (% of total)	2/4 (50%)	1/3 (33%)	3/7 (43%)
Number of events	3	1	4
**Tat 7.5 μg**			
Number of volunteers reporting the events (% of total)	1/4 (25%)	3/4 (75%)	4/8 (50%)
Number of events	1	12	13
**Tat 15 μg**			
Number of volunteers reporting the events (% of total)	1/3 (33%)	2/2 (100%)	3/5 (60%)
Number of events	2	2	4
**Tat 30 μg**			
Number of volunteers reporting the events (% of total)	2/4 (50%)	0/3 (0%)	2/7 (29%)
Number of events	12	0	12
**Total events**	**18**	**15**	**33**

N: number of volunteers.

<u>*Serious Adverse Events*</u>

Two SAEs were reported in the therapeutic protocol. One event was described as paralysis of the left VII nerve. It occurred 22 days after the II immunization (Arm A, Tat 15 μg) and was completely resolved at clinical examination performed after 2 weeks. Nevertheless, after that date the patient was hospitalised to perform the following instrumental and laboratory evaluation: NMR, BAER, virologic testing, CSF testing. The event was discussed by the experts of the DSMB. On the basis of the temporal interval with respect to the immunization, and the observation that facial paralysis has been reported to occur more frequently in HIV infected patients (Doutre, 1992; Casanova-Sotolongo, 2001), the association to the immunization was defined as "possible". No request of unblinding was submitted by the investigator.
The second event was described as lipothymia-nausea. The event occurred 50 min after the II immunization (Arm A, Tat 7.5 μg). The episode lasted for 10 min only, and then resolved without residual effects. The event was reported as "serious" because the clinical investigator directed the volunteer to the Hospital Emergency. However, the volunteer decided not to comply with the investigator suggestion. The relationship to study drug was defined as "possible" in the SAE form. As a possible concomitant/alternative cause of the event, the investigators refer "possible gastric block after breakfast". Indeed, 30 min after the immunization (and then 20 min before the occurrence of the event), the patient had assumed a breakfast, although the investigator indicated not to assume any food within 2 hours after immunization. Due to the benign nature of the event, no request for unblinding was submitted by the investigator.

Overall, the AEs and safety parameters reported indicate that the Tat vaccine is safe and well-tolerated when administered either subcute or intradermally. No severe local or systemic reactions were observed by clinicians, although two events were defined severe, one is the local event "Injection site infiltration" which was defined as "severe" while having a toxicity of grade 1; the second is the non local event "fever" which was defined as "severe" while having a toxicity of grade 2. There were not notable dose-dependent effects or differences between routes of administration for both the protocols ISS P-001 and ISS T-001.

1.6 Immunogenicity

The secondary end-point of the Phase I vaccine trials was to qualify Tat protein as immunogenic for evaluation in Phase II clinical trials. Two primary immunogenicity endpoints have been evaluated: induction of anti-Tat humoral immune response (anti-Tat specific IgM, IgG and IgA) and induction of anti-Tat cellular immune response [lymphoproliferation and γIFN/IL-4 production (Elispot) in response to Tat]. These evaluations were performed on the immunogenicity population, consisting of all volunteers who received at least three doses of the study medication and who had at least one valid post-baseline assessment.

1.6.1 Preventive Protocol (ISS P-001)

For the preventive protocol, 18 volunteers were treated as follows:

Arm A - Tat + Alum sc	7.5 μg: 3 subjects<br>15 μg: 3 subjects<br>30 μg: 2 subjects<br>placebo: 1 subject
Arm B - Tat id	7.5 μg: 2 subjects<br>15 μg: 2 subjects<br>30 μg: 2 subjects<br>placebo: 3 subjects

Humoral immune response

Assessment of anti-Tat humoral immune response was performed by the detection and titration of anti-Tat specific IgM, IgG and IgA antibodies in sera.
The presence and titer of specific anti-Tat IgM, IgG and IgA were evaluated at the baseline, week 8 (28 days after the II immunization), at week 12 (28 days after the III immunization), at week 16 (28 days after the IV immunization), at week 24 (56 days after the V immunization) and at week 48 (224 days after the V immunization).
As shown in Table 17, specific anti-Tat IgM and IgG were induced by all vaccine doses in both arms in all the immunized volunteers [14/14 (100%)], while anti-Tat IgA were induced in 12/14 (86%) of the immunized volunteers [Arm A: 8/8 (100%); Arm B: 4/6 (67%)]. No anti-Tat antibodies were detected in sera from individuals randomized in the placebo groups [0/4 (0%)].

Table 17. Frequency of anti-Tat humoral responses until 48 weeks (ISS P-001)

	IgM	IgG	IgA
**TAT+ALUM, SC**			
**7.5**	3/3 (100%)	3/3 (100%)	3/3 (100%)
**15**	3/3 (100%)	3/3 (100%)	3/3 (100%)
**30**	2/2 (100%)	2/2 (100%)	2/2 (100%)
**TOTAL**	**8/8 (100%)**	**8/8 (100%)**	**8/8 (100%)**
**TAT, ID**			
**7.5**	2/2 (100%)	2/2 (100%)	1/2 (50%)
**15**	2/2 (100%)	2/2 (100%)	1/2 (50%)
**30**	2/2 (100%)	2/2 (100%)	2/2 (100%)
**TOTAL**	**6/6 (100%)**	**6/6 (100%)**	**4/6 (67%)**
**TOTAL VACCINEES**	**14/14 (100%)**	**14/14 (100%)**	**12/14 (86%)**
**PLACEBO SC**	0/1 (0%)	0/1 (0%)	0/1 (0%)
**PLACEBO ID**	0/3 (0%)	0/3 (0%)	0/3 (0%)
**TOTAL PLACEBO**	**0/4 (0%)**	**0/4 (0%)**	**0/4 (0%)**

Cellular immune response

Assessment of anti-Tat cellular immune response was performed by the evaluation of *in vitro* γIFN and IL-4 production in response to Tat (by Elispot assay), and lymphoproliferative response to Tat (by 3H-thymidine incorporation).
The presence of a specific anti-Tat cellular immune response was evaluated at the baseline, week 5 (7 days after the II immunization), at week 13 (7 days after the IV immunization), at week 17 (7 days after the V immunization) and at week 48. As indicated in Table 18, an anti-Tat specific cellular response was induced in 13/14 (93%) immunized individuals [7/8 (87%) in Arm A, 6/6 (100%) individuals in Arm B]. In particular, proliferation activity to Tat was induced in 9/14 (64%) immunized individuals [5/8 (62.5%) in Arm A, 4/6 (67%) individuals in Arm B]. γIFN production in response to Tat was induced after immunization in 5/14 (36%) individuals [2/8 (25%) in Arm A, 3/6 (50%) in Arm B]. IL-4 production in response to Tat was induced after immunization in 12/14 (86%) individuals [7/8 (87.5%) in Arm A, 5/6 (83%) in Arm B]. No anti-Tat cellular immune response was observed in the individuals randomized in the placebo groups.

Table 18. Frequency of anti-Tat cellular responses until 48 weeks (ISS P-001)

-	CUMULATIVE RESPONSE	PROLIFERATION	IFNγ ELISPOT	IL-4 ELISPOT
**TAT+ALUM, SC**				
**7.5**	3/3 (100%)	2/3 (67%)	0/3 (0%)	3/3 (100%)
**15**	2/3 (67%)	1/3 (33%)	1/3 (33%)	2/3 (67%)
**30**	2/2 (100%)	2/2 (100%)	1/2 (50%)	2/2 (100%)
**TOTAL**	**7/8 (87%)**	**5/8 (62.5%)**	**2/8 (25%)**	**7/8 (87.5%)**
**TAT, ID**				
**7.5**	2/2 (100%)	1/2 (50%)	1/2 (50%)	1/2 (50%)
**15**	2/2 (100%)	2/2 (100%)	0/2 (0%)	2/2 (100%)
**30**	2/2 (100%)	1/2 (50%)	2/2 (100%)	2/2 (100%)
**TOTAL**	**6/6 (100%)**	**4/6 (67%)**	**3/6 (50%)**	**5/6 (83%)**
**TOTAL VACCINEES**	**13/14 (93%)**	**9/14 (64%)**	**5/14 (36%)**	**12/14 (86%)**
**PLACEBO, SC**	0/1 (0%)	0/1 (0%)	0/1 (0%)	0/1 (0%)
**PLACEBO, ID**	0/3 (0%)	0/3 (0%)	0/3 (0%)	0/3 (0%)
**TOTAL PLACEBO**	**0/4 (0%)**	**0/4 (0%)**	**0/4 (0%)**	**0/4 (0%)**

1.6.2 Therapeutic Protocol (ISS T-001)

For the therapeutic protocol the immunogenicity population, consisting of all volunteers who received at least 3 doses of the study medication and who had at least 1 valid post-baseline assessment, included 25 volunteers treated as follows:

Arm A - Tat + Alum sc
7.5 µg: 3 subjects
15 µg: 2 subjects
30 µg: 4 subjects
placebo: 4 subjects

Arm B - Tat id
7.5 µg: 4 subjects
15 µg: 2 subjects
30 µg: 3 subjects
placebo: 3 subjects

Humoral immune response

Assessment of anti-Tat humoral immune response was performed by the detection and titration of anti-Tat specific IgM, IgG and IgA antibodies. These evaluations were performed at the baseline and at different timepoints until week 48.

Since subjects enrolled in the T-001 protocol were HIV infected, some of them had already generated an immune response (humoral and/or cellular) against Tat. Immunogenicity evaluation should therefore consider the anti Tat immune responses present at baseline, before Tat vaccination. As shown in Table 19, anti-Tat IgM were present in 3/18 (17%) subjects at baseline [3/9 (33%) in Arm A, 0/9 (0%) in Arm B] as a response to the natural infection and, after vaccination, were induced in 15/18 (83%) of the immunized individuals [7/9 (78%) in Arm A, 8/9 (89%) in Arm B)]. One subject out of seven (14%) was positive for IgM at baseline in the placebo group. A transient production (at 1 time point) of anti-Tat IgM was also seen in one volunteer randomized in the placebo group (Arm B). Anti-Tat IgG were present in 2/18 (11%) subjects at baseline [1/9 (11%) in Arm A, 1/9 (11%) in Arm B] as a response to the natural infection and, after vaccination, were induced in all immunized individuals [18/18 (100%)]. One subject out of seven (14%) was positive for IgG at baseline in the placebo group. No production of anti-Tat IgG was reported in this group. Anti-Tat IgA were present in 0/18 (0%) subjects at baseline and, after vaccination, were induced in 11/18 (61%) of the immunized individuals [6/9 (67%) in Arm A, 5/9 (55%) Arm B)]. One subject out of seven (14%) was positive for IgA at baseline in the placebo group. No production of anti-Tat IgA was reported in this group during the study.

Table 19. Frequency of anti-Tat humoral immune response until 48 weeks (ISS T-001)

	IgM		IgG		IgA	
	**Prior**	**After**	**Prior**	**After**	**Prior**	**After**
**Tat + Alum, sc**						
**7,5**	1/3 (33%)	2/3 (67%)	0/3 (0%)	3/3 (100%)	0/3 (0%)	2/3 (67%)
**15**	1/2 (50%)	1/2 (50%)	0/2 (0%)	2/2 (100%)	0/2 (0%)	2/2 (100%)
**30**	1/4 (25%)	4/4 (100%)	1/4 (25%)	4/4 (100%)	0/4 (0%)	2/4 (50%)
**Total**	**3/9 (33%)**	**7/9 (78%)**	**1/9 (11%)**	**9/9 (100%)**	**0/9 (0%)**	**6/9 (67%)**
**Tat, id**						
**7,5**	0/4 (0%)	4/4 (100%)	0/4 (0%)	4/4 (100%)	0/4 (0%)	3/4 (75%)
**15**	0/2 (0%)	2/2 (100%)	1/2 (50%)	2/2 (100%)	0/2 (0%)	0/2 (0%)
**30**	0/3 (0%)	2/3 (67%)	0/3 (0%)	3/3 (100%)	0/3 (0%)	2/3 (67%)
**Total**	**0/9 (0%)**	**8/9 (89%)**	**1/9 (11%)**	**9/9 (100%)**	**0/9 (0%)**	**5/9 (55%)**
**TOTAL VACCINEES**	**3/18 (17%)**	**15/18 (83%)**	**2/18 (11%)**	**18/18 (100%)**	**0/18 (0%)**	**11/18 (61%)**
**Placebo, sc**	1/4 (25%)	1/4 (25%)	1/4 (25%)	1/4 (25%)	1/4 (25%)	1/4 (25%)
**Placebo, id**	0/3 (0%)	1/3 (33%)	0/3 (0%)	0/3 (0%)	0/3 (0%)	0/3 (0%)
**TOTAL PLACEBO**	**1/7 (14%)**	**2/7 (29%)**	**1/7 (14%)**	**1/7 (14%)**	**1/7 (14%)**	**1/7 (14%)**

Cellular immune response

Assessment of anti-Tat cellular immune response was performed by the evaluation of *in vitro* γIFN production in response to Tat (by Elispot assay), *in vitro* IL-4 production in response to Tat (by Elispot assay) and lymphoproliferative response to Tat (by 3H-thymidine incorporation). These tests were performed with peripheral blood mononuclear cells (PBMC) obtained from immunized subjects. The presence of a specific anti-Tat cellular immune response was evaluated at the baseline and at different time points until week 48.

A cellular immune response to Tat (γIFN production and/or IL-4 production and/or proliferation) was present at baseline as response to the natural infection in 15/18 (83%) individuals randomized in the groups to be treated with the Tat vaccine [8/9 (89%) in Arm A, 7/9 (78%) individuals in Arm B], and in 6/7 (86%) individuals randomized in the placebo groups. After immunization, anti-Tat cellular responses were observed in 18/18 (100%) individuals, while a decrease was observed in the placebo groups [4/7 (57%)] (Table 20).

In particular, lymphoproliferation in response to Tat was present at baseline in 11/18 (61%) individuals randomized in the groups to be treated with the Tat vaccine [6/9 (67%) in Arm A, 5/9 (55%) individuals in Arm B], and in 2/7 (29%) individuals randomized in the placebo groups. After immunization, anti-Tat proliferation was observed in 16/18 (89%) individuals [7/9 (78%) in Arm A, 9/9 (100%) individuals in Arm B], while a decrease was observed in the placebo groups [1/7 (14%)].

γIFN production in response to Tat was present, at baseline, in 10/18 (55%) individuals randomized in the groups to be treated with the Tat vaccine [4/9 (44%) in Arm A, 6/9 (67%) individuals in Arm B], and in 5/7 (71%) individuals randomized in the placebo groups. After immunization, this response was observed in 15/18 (83%) individuals [7/9 (78%) in Arm A, 8/9 (89%) individuals in Arm B], while a decrease was observed in the placebo groups [4/7 (57%)]. IL-4 production in response to Tat was present, at baseline, in 3/18 (17%) individuals randomized in the groups to be treated with the Tat vaccine [1/9 (11%) in Arm A, 2/9 (22%) individuals in Arm B], and in 1/7 (14%) individuals randomized in the placebo groups. Similarly to that observed for γIFN production, after immunization IL-4 production increased in the vaccinated volunteers, with 9/18 (50%) positive individuals [2/9 (22%) in Arm A and 7/9 (78%) individuals in Arm B]. All the individuals in the placebo groups were negative for IL-4 production at the end of the study [0/7 (0%)].

Table 20. Frequency of anti-Tat cellular responses prior to (baseline) or after vaccination (48 weeks) (ISS T-001)

	Total Response		Proliferation		IFNγ Elispot		IL-4 Elispot	
	**Before**	**After**	**Before**	**After**	**Before**	**After**	**Before**	**After**
**Tat+Alum, SC**								
**7.5**	2/3 (67%)	3/3 (100%)	1/3 (33%)	2/3 (67%)	1/3 (33%)	2/3 (67%)	1/3 (33%)	0/3 (0%)
**15**	2/2 (100%)	2/2 (100%)	1/2 (50%)	2/2 (100%)	1/2 (50%)	1/2 (50%)	0/2 (0%)	1/2 (50%)
**30**	4/4 (100%)	4/4 (100%)	4/4 (100%)	3/4 (75%)	2/4 (50%)	4/4 (100%)	0/4 (0%)	1/4 (25%)
**Total**	**8/9 (89%)**	**9/9 (100%)**	**6/9 (67%)**	**7/9 (78%)**	**4/9 (44%)**	**7/9 (78%)**	**1/9 (11%)**	**2/9 (22%)**
**Tat, ID**								
**7.5**	3/4 (75%)	4/4 (100%)	2/4 (50%)	4/4 (100%)	2/4 (50%)	3/4 (75%)	1/4 (25%)	4/4 (100%)
**15**	1/2 (50%)	2/2 (100%)	1/2 (50%)	2/2 (100%)	1/2 (50%)	2/2 (100%)	1/2 (50%)	1/2 (50%)
**30**	3/3 (100%)	3/3 (100%)	2/3 (67%)	3/3 (100%)	3/3 (100%)	3/3 (100%)	0/3 (0%)	2/3 (67%)
**Total**	**7/9 (78%)**	**9/9 (100%)**	**5/9 (55%)**	**9/9 (100%)**	**6/9 (67%)**	**8/9 (89%)**	**2/9 (22%)**	**7/9 (78%)**
**TOTAL VACCINEES**	**15/18 (83%)**	**18/18 (100%)**	**11/18 (61%)**	**16/18 (89%)**	**10/18 (55%)**	**15/18 (83%)**	**3/18 (17%)**	**9/18 (50%)**
**Placebo, SC**	4/4 (100%)	3/4 (75%)	1/4 (25%)	1/4 (25%)	3/4 (75%)	3/4 (75%)	1/4 (25%)	0/4 (0%)
**Placebo, ID**	2/3 (67%)	1/3 (33%)	1/3 (33%)	0/3 (0%)	2/3 (67%)	1/3 (33%)	0/3 (0%)	0/3 (0%)
**TOTAL PLACEBO**	**6/7 (86%)**	**4/7 (57%)**	**2/7 (29%)**	**1/7 (14%)**	**5/7 (71%)**	**4/7 (57%)**	**1/7 (14%)**	**0/7 (0%)**

2. STUDY OBJECTIVES

2.1 Primary Objectives

- To investigate the immunogenicity of 5 vaccine injections with 7.5 μg of recombinant, biologically active, HIV-1 Tat protein given intradermally (id), compared to 3 injections with the same dose;

- To investigate the immunogenicity of 5 vaccine injections with 30 μg of recombinant, biologically active, HIV-1 Tat protein given id, compared to 3 injections with the same dose;

- To compare the immunogenicity of 3 and 5 vaccinations with 7.5 μg of recombinant, biologically active, HIV-1 Tat protein given id, with that induced by 3 and 5 vaccinations with 30 μg given id, respectively.

2.2 Secondary Objectives

- To compare the safety of the four vaccination regimens based on the administration of 7.5 μg and 30 μg of recombinant, biologically active, HIV-1 Tat protein given id in 3 or 5 injections, by monitoring the incidence of adverse events, including any significant change in hematological/biochemical laboratory parameters.

3. STUDY DESIGN

The study is a randomized, open label, phase II clinical trial directed to evaluate the immunogenicity and the safety of the HIV-1 Tat protein-based vaccine. HIV-1 positive subjects will be recruited and screened. Volunteers will be eligible to enrolment based on the inclusion and exclusion criteria described in Section 5, Paragraphs 5.3 and 5.4. The study will last 48-weeks, including a period of 16 or 8 weeks treatment phase and a period of 32 or 40 weeks follow-up phase, respectively, in arm A and B.
This study will be conducted in 12 clinical sites in Italy.
The enrolment will be competitive, therefore no specific number of enrolled subjects is established per site *a priori*.
One hundred twenty-eight subjects will be randomized 1:1:1:1 to one of the four treatment groups, according to the immunization schedule showed in the following paragraph 4.1.2.

3.1 Rationale for the Selection of Vaccine Dosage and Schedule of Immunization

As described above, the safety and immunogenicity of three dosages (7.5 μg, 15 μg or 30 μg) of the Tat vaccine administered sc (in association with the Alum adjuvant) or id, have been investigated in a Phase I trial, in HIV-1 infected adult volunteers with mild immune deficiency characterized by $CD4^+$ T cell counts ≥ 400/μL and levels of plasma viremia ≤ 50,000 copies/ml.
Overall, the AEs and safety parameters reported indicate that the Tat vaccine is safe and well tolerated when administered both subcute or intradermally. In particular, there were not notable dose-dependent effects or differences between routes of administration.
The Tat vaccine resulted highly immunogenic having induced or increased specific humoral and cellular immune responses in the vaccinated subjects. Optimal humoral immune responses were induced after immunization with 7.5 and 30 μg of Tat, by both routes of administration. In particular 30 μg of Tat induced a more persistent humoral immune response. In addition, Tat-specific cellular responses, particularly IL-4 and lymphoproliferation, were more frequently observed in subjects randomized to intradermic administration (Arm B of the phase I clinical trial, § 1.6.2).
Therefore, id administration of the vaccine was chosen for the present study. With respect to other routes of administration, it does not require adjuvant and is, therefore, easier to handle and less expensive to produce and deliver. This is a relevant advantage in the setting of large scale vaccination campaigns in populations living in poor health conditions like African and Asian populations. No dose-dependent effects were observed in terms of safety and tolerability, therefore, five immunizations with 7.5 μg or 30 μg of Tat will be administered (Arm A), as performed for the Arm B of the Phase I clinical trial. In addition, since peak anti-Tat antibody titers were observed

after three immunizations, the immunogenicity of a five-immunizations schedule (Arm A), used also in the phase I trial, will be compared to a three-immunizations schedule (Arm B), which is both less expensive and favoring a better compliance to the treatment for the enrolled subjects.

4. STUDY MEDICATION AND DOSAGE REGIMENS

4.1 Study Medication - Background on Study Medication preparation

The study medication is the biologically active recombinant Tat protein to be tested in two different doses and two administration regimens. Research products for this protocol will be supplied by ISS. Numbered kits, one per subject, will be provided.
ISS will keep one reference sample of each of the two dosages (Tat Vaccine 7.5 μg and Tat Vaccine 30 μg) into his samples archive, according to GCP regulations.

The biologically active Tat utilized for vaccination is a recombinant protein obtained as described in paragraph 1.2.
Three or five doses of Tat 7.5 μg or 30 μg will be administered intradermally.
The vaccine contains only the Tat antigen.
Tat is produced according to GMP by Diatheva-Avitech Srl.
The officine responsible for Tat vialing and labeling is Injectalia Srl.

Tat 7.5 μg
Batch number: T2051007
Expiration date: 10/2009
Active substance: Biologically active Tat protein
Buffer composition: Phosphate saline buffer, pH 7.4, 1% sucrose, 1% Human Serum Albumine

Tat 30 μg
Batch number: T3051007
Expiration date: 10/2009
Active substance: Biologically active Tat protein
Buffer composition: Phosphate saline buffer, pH 7.4, 1% sucrose, 1% Human Serum Albumine

ISS will also provide the sterile water (Industria Farmaceutica Galenica Senese, Lot number 03H07, Expiration date 08/2010) for the dilution of Tat.

4.1.1 Administration

Each kit contains 3 or 5 vials, according to the randomization list, representing the complete treatment for one subject. At the moment of the vaccine administration the Investigator will have to complete the label's vial and then to attach the tear-off label on the appropriate form. Also the Investigator will have to record the sterile water code and batch number on the appropriate form.
All the treatment kits have to be stored at -80°C and protected from light until the time of injection.
For the administration of the study medication, the vial's content (0.5 ml), needs to be thawed and diluted with 1.5 ml of sterile water right before the moment of injection which should occur within a maximum of 40 minutes from the preparation.
When ready for administration, the mixture must be swirled gently for 10 seconds and then the assigned volume (2.0 ml) of the vaccine preparation must be administered by two intradermal injections into the right and left deltoid regions of the upper arms.

The real volume that can be drawn from each vial, is 0.5 ml and it is the drawing volume certified by the manufacturer. To guarantee this volume, and in consideration of the residual liquid in the vial after withdrawal, an "overfilling volume" (2%) was introduced during manufacturing process to permit the correct withdrawing of the volume required for the immunization.

4.1.2 Dosage Regimens

Tat (7.5 μg or 30 μg) will be administered intradermally according to the following schedule:

- Arm A - Group I: 5 immunizations with Tat (7.5 μg) at weeks 0, 4, 8, 12, 16;
- Arm A - Group II: 5 immunizations with Tat (30 μg) at weeks 0, 4, 8, 12, 16;
- Arm B - Group I: 3 immunizations with Tat (7.5 μg) at weeks 0, 4, 8;
- Arm B - Group II: 3 immunizations with Tat (30 μg) at weeks 0, 4, 8.

			**Immunization Schedule**				
**Arm A**	**Dose Levels**	**Accrual**	**Week 0**	**Week 4**	**Week 8**	**Week 12**	**Week 16**
Group I	Tat 7.5 μg (id)	32	X	X	X	X	X
Group II	Tat 30 μg (id)	32	X	X	X	X	X
	Subtotal	n = 64					
**Arm B**	**Dose Levels**	**Accrual**	**Week 0**	**Week 4**	**Week 8**	**Week 12**	**Week 16**
Group I	Tat 7.5 μg (id)	32	X	X	X	-	-
Group II	Tat 30 μg (id)	32	X	X	X	-	-
	Subtotal	n = 64					
	**Total**	**128**					

No increase/decrease of the study medication dosage is foreseen during the study.

4.2 Study Duration

The treatment phase will last 16 weeks for Arm A and 8 weeks for Arm B, with immunizations administered every 4 weeks.
To evaluate eligibility, subjects will be screened before the treatment initiation (screening phase) and then monitored until week 48 from the first immunization (thus corresponding to a 32-weeks period after treatment completion for Arm A subjects, and to a 40-weeks period after treatment completion for Arm B subjects, respectively).

4.3 Study Medication Delivery

Study medication will be supplied by ISS after the completion of all the Ethical and Administrative procedures. The study medication will be supplied through a dedicated courier specialized for refrigerated shipment of biologicals. At receipt, it will be responsibility of the clinical site pharmacist or a delegated person to verify immediately that the temperature conditions have been maintained during the shipment and to ensure that the study drug is stored at -80°C and in the absence of light.
Numbered kits, one per subject, will be provided. Each kit will contain the complete set of vials containing the vaccine preparations required for a single subject (three or five vials, according to the randomization list).
Sterile water for dilution will be provided by ISS.
Study medication will be delivered to clinical sites in sets of 4 treatment blocks, according to the randomization list. Each clinical site will be then re-supplied when the block of 4 has been completely allocated to enrolled subjects. Therefore re-supply will follow the rate of recruitment at each clinical site.
In case a vial of the assigned study medication results damaged during the process of preparation, the investigator should immediately inform the site monitor (Opera) to arrange for the replacement of the vial(s) from the Sponsor.

4.4 Packaging and Labeling of Study Medication

Each vaccine vial (7.5 μg or 30 μg) will be packaged in single boxes; five or three boxes, each containing one vial, will be packaged in one kit-box. Each kit-box will contain the complete treatment for one enrolled subject.
Blocks of 4 kit-boxes will be packaged in a bigger container, which will correspond to the minimum quantity of study drug treatments shipped from ISS to each clinical site.
The study medication will be packaged and labeled according to the pertinent guideline (Volume 4 - Good Manufacturing Practices - Annex 13, Manufacture of investigational medicinal products, July 2003).

The information included in labels is:

(a) name, address and telephone number of the Sponsor;
(b) pharmaceutical dosage form, route of administration, quantity of dosage units;
(c) the batch and/or code number to identify the contents and packaging operation;
(d) a trial reference code allowing identification of the trial, clinical site and investigator;
(e) the trial subject identification number, the treatment number and the visit number;
(f) directions for use;
(g) "For clinical trial use only";
(h) the storage conditions;
(i) period of use, expiration date in a month/year format.

The facsimile of labels is reported in Appendix III.
The drug label will be written in the language of the Country where the study takes place (Italian language).

4.5 Study Medication Storage and Accountability

The study medication has to be stored in a lockable storage area, at -80°C and protected from light.

It is the investigator/institution's responsibility to make all reasonable efforts to assure that:

- products delivered from ISS are correctly received (the CRO clinical trial monitor will collect the signed and dated receipt form)
- study medication is handled in a safely and properly manner and it is stored in a secured area
- study medication is administered to subjects only in accordance with the protocol requirements
- any unused study medication and residual used vials are returned to the Sponsor.

Each center will be requested to exhibit cryopreservation records that demonstrate the study medication is being adequately preserved. Records will be checked by the CTM in occasion of monitoring visits.
Study drugs will be administered to subjects only by authorized personnel (Principal Investigator, Co-Investigators and nurses under the Principal Investigator direct responsibility).
Drug inventory and accountability records will be kept by the Investigator or by the Pharmacist, or other authorized person.
To ensure adequate records, all study medication will be accounted for in the case report form and drug accountability inventory forms. The inventory will include details of the received and dispensed study medication.
At the conclusion of vaccine administration, all study medication supplies (including used, unused, and partially used vials of the vaccine), will be returned to the Injectalia Srl.

4.6 Randomization Codes

Being this study an open label study, the randomization process will be centralised and carried out via web, with the sole purpose of avoiding the potential bias that an open allocation to treatment could cause. Therefore no randomization codes will be used.
Each clinical site will receive a block of 4 numbered treatments (according to a randomization scheme 1:1:1:1 and to the randomization list) but the investigator will not be aware of the allocation of the 4 treatments until he randomizes the subject via web (that means just before the first dose is to be administered).

4.7 Concomitant Treatments

Subjects should receive standard therapy for any medical condition, including viral or bacterial or other infections that develop during the study. Chronic stable therapy (e.g., antihypertension therapy) will be permitted.
Subject in need of any therapy listed in the exclusion criteria, must withdraw from study treatment.
All other concomitant treatments will be recorded on the e-CRF, including the name of the procedure or the drug (both trade name and generic name should be recorded), medical indication, route, total daily dose and start/stop dates).

4.7.1 Prior Treatments

Reasonable efforts will be made to determine all relevant pharmacological and non pharmacological treatments received by the subject within the four months before screening visit. All relevant

information must be recorded on the e-CRF, including the name of the treatment (if pharmacological, trade name and generic name are required), indication, route, total daily dose and start/stop dates.
To be enrolled, subject must be compliant with all the requirements listed in the exclusion criteria that means he/she must not use/have used any of the listed therapies.

4.7.2 Antiretroviral Treatments

Subject should receive standard antiretroviral therapy according to his/her clinical conditions and current guidelines.
All antiretroviral treatments need to be recorded on the e-CRF, including the name of the drug (trade name and generic name), indication, route, total daily dose and start/stop dates.

5. METHODS

5.1 Diagnosis

Subjects will be HIV-1 infected adult of either gender, 18-55 years of age, anti-Tat antibody negative, HAART-treated with chronic suppressed HIV-1 infection: $CD4^+$ T cell counts $\geq$ 400 cells/µl, levels of plasma viremia < 50 copies/ml in last 6 months prior to the screening and without a history of virological rebound. The pre-HAART CD4 nadir must be > 250 cells/µl.

5.2 Psychological Evaluation

A psychological evaluation (Appendix V) has been prepared on the basis of the important support given by this approach in phase I studies. The aim of psychological evaluation is to identify psychological profiles which might require a particular support during the most critical steps of the trial (exclusion, enrolment, occurrence of adverse events, drop out). In addition, an evaluation by the psychologist/psychiatrist will support the investigators in confirming the eligibility of the volunteer for the study by identifying major psychotic disorders.
The psychological evaluation will also allow the assessment of motivation, anxiety, social impact and quality of life experienced by the trial participants, therefore alerting the psychologist/psychiatrist at the clinical site of specific needs which may require support to volunteers such as those arising at critical points during the study (screening failure, enrolment, treatment phase, conclusion of the study, adverse events and follow-up).

5.3 Inclusion Criteria

All the following criteria should be met for the subjects to be eligible for the study:

1) Age 18-55 years;
2) Anti-Tat antibody negative subjects;
3) HIV-1 infected subjects under successful HAART treatment (with chronic suppressed HIV-1 infection) with $CD4^+$ T cell counts $\geq$ 400 cells/µl determinated by 2 separate evaluations within the 3 weeks pre-study screening period (at day -21 and then between day -14 and -7);
4) HIV plasma viremia < 50 copies/ml in the last 6 months prior to the screening and without a history of virological rebound;

5) Subjects with pre-HAART CD4 nadir > 250 cells/µl;
6) Availability for the planned study duration;
7) Negative pregnancy test for women of childbearing potential (to be performed during the screening phase and just before the immunizations) and use of an acceptable mean of contraception (condom, hormonal or mechanical methods) for one month prior to immunization and for the all duration of the study;
8) Signed informed consent.

5.4 Exclusion Criteria

Any of the following criteria will exclude the subject from this study:

1) History of AIDS-related opportunistic or neoplastic disease;
2) History of encephalopathy, neuropathy, or unstable CNS pathology (HIV or non-HIV related);
3) History of non-HIV related neoplastic diseases, autoimmune diseases, severe and/or persistent angina or cardiac arithymias, or severe or uncontrolled systemic disease (e.g., unstable or uncompensated respiratory, cardiac, hepatic, thyroid gland or renal disease);
4) Any evidence, as judged by the investigator, of unstable cardio-vascular disease (e.g. unstable hypertensive disease needing modification or introduction of an anti-hypertensive treatment);
5) Laboratory findings exceeding the normal range adopted by each clinical site laboratory for haematology and biochemistry assessments will make undesirable for the subject the participation to the study. In particular, subjects presenting AST/ALT > 3 x the upper limit of normal will be excluded, as well as AST/ALT > 5 x the upper limit of normal in case of subjects having co-infections HIV/HCV related;
6) Chest radiography, within 6 months prior to study screening visit, showing evidence of active or acute cardiac or pulmonary disease;
7) History of anaphylaxis or serious adverse reactions to vaccines as well as serum IgE levels exceeding 1000 U.I./ml;
8) History of serious allergic reaction to any substance, requiring hospitalization or emergent medical care (e.g. Steven-Johnson syndrome, bronchospasm, or hypotension);
9) Early syphilis documented by Syphilis serology test [*NOTE: if serology is documented to be a false positive or due to a remote (> 6 months), successfully treated infection, the subject is eligible*];
10) Active tuberculosis documented PPD skin test within one year [*NOTE: if the PPD skin test is positive, then a chest x-ray will be done and if no findings consistent with active pulmonary tuberculosis and no indications exist for prophylaxis or treatment, the subject is eligible for participation in this trial*];
11) Medical or psychiatric condition which preclude subject compliance with the protocol. Specifically, persons with psychotic disorders, major affective disorders, suicidal ideation are to be excluded;
12) Current use of psychotrophic drugs prescribed for major psychotic disorders;
13) Use of any experimental HIV therapy or participation in another experimental protocol;
14) Current or prior therapy with immunomodulators or immunosuppressive drugs and anticoagulant drugs within 30 days prior to study medication administration;
15) Concomitant treatment for HBV or HCV infections;
16) Live attenuated vaccines within 60 days of study inclusion [*NOTE: Medically indicated sub-unit or killed vaccines (e.g., influenza, pneumococcal, hepatitis A and B) are not exclusionary, but should be given at least 4 weeks away from HIV immunizations*];

17) Receipt of blood products or immunoglobulin in the past year;
18) Previous participation in an HIV-1 vaccine trial;
19) Drug and/or alcohol abuse;
20) Pregnant or lactating women.

5.5 Screening Failures

The investigator will maintain a log of all screening failures. A screening failure is defined as any subject who signs the informed consent but does not receive any study medication due to the exclusion from the study.

5.6 Randomization

As subjects are screened for the study they must be provided with a screening code. The screening code is a code made up by the clinical trial code (T2) + the clinical site number (01 → 12) + the progressive number within a particular site: for example the first subject screened at clinical site n. 05 would be assigned the screening code T205001, the second subject screened would be T205002 and so on.
All screened subjects are assigned a screening code irrespective of whether or not they will receive the study treatment. If a subject discontinues from the study at any time (even before the first immunization), the screening code will not be reused.
Subjects will be randomly assigned to treatment regimens according to a computer generated list. The subjects will be allocated to treatment in balanced blocks.
Once eligibility is established (see eligibility criteria in Section 5, Paragraphs 5.3 and 5.4), the clinical staff will contact the CRO Opera via web, to randomize the subject. The drug kit number to be assigned to the subject will be provided via web within the block's numbers that the clinical site has received. The assigned drug kit number will be recorded on the e-CRF.
Original randomization list will be kept by the Sponsor and the CRO.
If a subject is randomized, but withdrawn from the study for any reason before the first immunization, the drug kit number can be associated to another subject upon informing Opera by phone and fax/e-mail. Records of such re-allocation will be traced and maintained.

5.7 Blinding

Not applicable, since the study is an open label study.

5.8 Study Assessments

The following section provides a detailed listing of the clinical, immunological and virological determinations to be performed in this protocol.
Clinical determinations will be made at each clinical site. Local sites will be responsible for the hematology (RBC, HB, HCT, MCV, Platelets, Total WBC, Neutrophils, Lymphocytes, Monocytes, Eosinophils, Basophils), blood chemistry (Sodium, Potassium, Calcium, Total Protein, Albumin, Total Bilirubin, Alkaline Phosphatase, AST, ALT, GGT, Urea, Creatinine), serum IgE levels, thyroid parameters, $CD4^+$ T cell counts, and HIV-1/2 ELISA determinations, the coagulation assessment (Thrombin Time, Prothrombin Time, APTT), the urinalysis (Protein, Glucose, Nitrate,

Albumin, by dispstick), the pregnancy test, the ECG as well as PPD test, chest X-ray (according to exclusion criteria requirements) and Syphilis serology test.
A Core Laboratory of Immunology and Virology (*Core Laboratory of Immunology and Virology Ospedale S. Gallicano IFO, Rome)* will centralize all the immunologic and virological assessment that go beyond the routine clinical monitoring of the patients applying Standard Operating Procedures (SOPs) based on a consensus panel of assays developed within the AIDS Vaccine Integrated Program (AVIP), funded within the FP6 program of the European Community and the Italian Concerted Action on HIV/AIDS Vaccine Development (ICAV), funded within the Italian AIDS National Program (Ministry of Health). The same SOPs were employed to determine the immunogenicity induced by the Tat-vaccine in phase I preventative (P-001) and therapeutic (T-001) clinical trials (Ensoli, 2006).

The amount of blood to be collected for the purposes of this study will be reduced to a minimum volume (about 50 ml) that will guarantee the immunological and virological assessments as well as all the primary safety determinations necessary to fulfill the needs of a first line of immunological and virological testing. A second line of immunological and virological testing will be performed according to the availability of residual specimens (PBMC, sera, plasma).
The first line of testing includes the minimal panel of assays required to identify the presence of an immune response to Tat, which represents the primary endpoint of the study. The second line of testing has been elaborated in order to allow a more detailed exploration of the immune response against Tat.

**Assessment**		**Blood sample volume (ml)**
Immunology/Virology		40
Safety	Clinical chemistry	7
	Hematology	3
**Total**		**50**

5.8.1 Immunological Evaluations

Immunological evaluations will be performed according to a “first line” and a “second line” laboratory testing by the Core Laboratory.

First line immunological testing
Assessment of anti-Tat humoral immune response:
- Determination of IgM, IgG and IgA anti-Tat antibodies in sera
- Titration of IgM, IgG and IgA anti-Tat antibodies
- Neutralization of Tat activity by in vitro assays (rescue inhibition assay).

Assessment of anti-Tat cellular immune response:
- Lymphoproliferative response to Tat (CFSE staining)
- *In vitro* γIFN, IL-4 and IL-2 production in response to Tat (Elispot).

Second line immunological testing
These studies will be performed, according to residual specimen (PBMC, sera, plasma) availability:
- Characterization of lymphocyte subsets (CD3, CD4, CD8, CD16, CD56, CD19)
- Anti-Tat IgG (IgG1, IgG2, IgG3, IgG4) subclasses
- Epitope mapping of IgM and IgG anti-Tat antibodies
- Anti-HIV regulatory and structural proteins antibodies

- Antibody-mediated cellular cytotoxicity (ADCC)
- Inhibition of Tat uptake by MDDCs
- Neutralization of primary isolates (all clades)
- Anti-CCR5 antibodies
- Anti-CD4 antibodies
- Lymphoproliferative response to mitogens and recall antigens
- Lymphoproliferative response to HIV-1 Env (CFSE staining)
- *In vitro* γIFN, IL-4 and IL-2 production in response to Env (ICS/Elispot)
- B cells phenotype (naïve and memory)
- Phenotype and functional characterization of regulatory T cells
- Intracellular PBMC staining for granzyme, perforin, cytokines and chemokines
- Analysis of Th1 and Th2 cytokines in sera and in PBMC supernatants
- Analysis of chemokines in sera and in PBMC supernatants
- Lymphocytes spontaneous cell death
- B cell cloning
- Functional and molecular characterization of clono-specific antibodies
- Serum/plasma determination of soluble CD4
- HLA typing.

5.8.2 Virological Evaluations

Virological evaluations will be performed according to a “first line” and a “second line” laboratory testing by the Core Laboratory by determining:

HIV-1 plasma viremia (viral RNA copies, first line).

Additional (second line) virologic studies will be performed, according to residual specimens (PBMC, sera, plasma) availability. These will include:

- HIV-1 sequencing and phylogenetic analysis
- Genotypic resistance
- Viral tropism
- Anti-HTLV-I antibodies
- Anti-HTLV-II antibodies
- Anti-HBV antibodies
- HBV antigens (HbsAg, HbeAg)
- Anti-HCV antibodies
- HHV-8 antibodies and plasma viremia
- HIV-1 Proviral DNA copies.

5.8.3 Sample Collection, Storage and Shipment

The volume of blood to be collected at each visit will be 50 ml (40 ml for immunologic and virologic evaluations, 10 ml for assessing safety parameters). Blood collection will be made by venipuncture on an empty stomach.
Primary safety tests will be made locally, while immunological and virological evaluations will be made by the Core Lab.

Whole blood samples will be sent at room temperature to the Core Lab within the collection day, at each visit, while plasma and sera will be frozen and stored at clinical site and will be sent in dry ice to the Core Lab (San Gallicano Hospital, Rome) as soon as possible (by the next shipping at room temperature at the following visit, anyway within two weeks since the collection day).

Each sample will be identified with the following information: protocol number, center number, subject number (screening code), visit number, sample type (plasma, serum or whole blood) and sample collection date.
In case of availability of residual PBMC, serum and plasma specimens (after the fulfillment of the first line tests described in Paragraphs 5.8.1 and 5.8.2) will be directly utilized or kept frozen until utilization for the additional second line tests indicated (Paragraphs 5.8.1 and 5.8.2).
The shipment of blood samples from the Clinical Sites to the Core Laboratory will be organized and performed directly by the Sponsor through a dedicated courier. Sample shipment and storage costs as well as samples examination costs for the analysis performed by the Core Laboratory will be covered by the Sponsor.
A specific manual with a complete description of procedures to be followed for samples collection and management will be provided to investigational sites before study initiation.

5.9 Schedule of Study Procedures

The following section provides a detailed listing of the safety and immunogenicity determinations to be performed in this protocol at the designated time points (see the Flow Chart in Appendix II).

Subjects randomized to Arm A will receive 5 immunizations and after that they will have 3 follow-up visits, respectively at day 140 (week 20), 168 (week 24) and 336 (week 48).

Subjects randomized to Arm B will receive 3 immunizations and after that they will have 5 follow-up visits, respectively at day 84 (week 12), 112 (week 16), 140 (week 20), 168 (week 24) and 336 (week 48).

It will be acceptable that follow-up visits are anticipated or delayed of 7 days, at maximum.
The timing of each follow-up visit will be scheduled according to the planned follow-up period.

DAY -21 SCREENING PERIOD (Prior to Vaccination)

Informed consent for the study will be obtained prior to any assessment of eligibility.

There will be a three-weeks pre-study screening period. During this period, pre-admission screening tests and evaluations will be performed to determine the eligibility of each subject for the study.
Investigators will proceed with the medical history, physical and instrumental examination and blood testing. Eligibility determination for the trial will also depend on the results of laboratory tests and on the score of the self-administered psychological assessment and/or the psychiatric interview.

At study entry, fulfillment of all the following points is strictly required:
- Signed informed consent
- Medical History; complete physical examination and vital signs: weight, height, sitting blood pressure, sitting respiratory rate, sitting heart rate, axillary temperature; history of previous (four months) and concomitant treatments

- 12-lead ECG
- Counseling on avoidance of pregnancy
- Pregnancy test for females
- Psychological assessments and tests (according to the psychological evaluation flow-chart)
- Hematological analysis, blood chemistry (included thyroid, kidney and hepatic routine analysis) and coagulation assessment
- Urine dipstick
- RPR (syphilis serology) [*NOTE: If serology is documented to be a false positive or due to a remote (> 6 months), successfully treated infection, the subject is eligible*]
- Documented PPD skin test within one year [*NOTE: If the PPD skin test is positive, then a chest x-ray will be done and if no findings consistent with active pulmonary tuberculosis and no indications exist for prophylaxis or treatment, the subject is eligible for participation in this trial*]
- Chest x-ray, if PPD skin test is positive or if an x-ray examination performed within 6 months prior to screening is not available to exclude active or acute cardiac or pulmonary disease
- Serum IgE levels
- $CD4^+$ T cell counts: the test must be repeated twice within the screening period (firstly at -21 and secondly between -14 and -7), to confirm the value as for inclusion criteria n. 3
- Virological evaluation: HIV1/HIV2 ELISA
- HIV-1 plasma viremia
- Immunological evaluations: Anti-Tat antibodies
- Samples collection and storage (-80°C) at clinical site
- Samples collection and shipment (whole blood).

All inclusion and exclusion criteria must be assessed and met prior to study medication administration.

DAY 0 (Baseline)

Prior to the vaccine administration:

- Physical examination and vital signs: weight, sitting blood pressure, sitting respiratory rate, sitting heart rate, axillary temperature
- Concomitant treatments (medications)
- Laboratory determinations: hematology, blood chemistry and coagulation assessments, urine dipstick
- Pregnancy test (females)
- Psychological assessments and tests
- $CD4^+$ T cell counts
- HIV-1 plasma viremia
- Anti-Tat antibodies (IgG, IgM, IgA)
- Neutralization of Tat activity
- Lymphoproliferative response to Tat
- γIFN, IL-4 and IL-2 production in response to Tat
- Assessment of adverse events
- Review of Inclusion and Exclusion Criteria (before the first immunization)
- Sample collection and shipment (whole blood).

VACCINATION
Study medications will be allocated to subjects according to the randomization performed via web. Instruction for administration are reported in Section 4, Paragraph 4.1.1.

After Day 0 (the first vaccination) the following 2 or 4 vaccination visits will be scheduled with a 4 weeks interval with the possibility to delay each vaccination of 1 week at maximum (+ 7 days).

Following To Vaccination
After administration of the study medication, subjects will remain at the study center for 2 hours for the following assessments:
- Physical examination and vital signs: weight, sitting blood pressure, sitting respiratory rate, sitting heart rate, axillary temperature
- Counseling on avoidance of pregnancy
- Assessment of adverse events.

During this period the subject will not assume any food.

DAY 28 (2nd immunization)

Prior to the vaccine administration:

- Physical examination and vital signs: weight, sitting blood pressure, sitting respiratory rate, sitting heart rate, axillary temperature
- Concomitant treatments (medications)
- Laboratory determinations: hematology, blood chemistry and coagulation assessments, urine dipstick
- Pregnancy test (females)
- $CD4^+$ T cell counts
- HIV-1 plasma viremia
- Anti-Tat antibodies (IgG, IgM, IgA)
- Assessment of adverse events
- Samples collection and storage (-80°C) at clinical site
- Samples collection and shipment (whole blood).

VACCINATION
The subject will be administered with the study medications allocated to him/her at randomization. Instruction for administration are reported in Section 4, Paragraph 4.1.1.
The following vaccination visit will be scheduled with a 4 weeks interval with the possibility to delay the vaccination of 1 week at maximum (+ 7 days).

Following To Vaccination
After administration of the study medication, subjects will remain at the study center for 2 hours for the following assessments:
- Physical examination and vital signs: weight, sitting blood pressure, sitting respiratory rate, sitting heart rate, axillary temperature
- Counseling on avoidance of pregnancy
- Assessment of adverse events.

During this period the subject will not assume any food.

DAY 56 (3rd immunization)

Prior to the vaccine administration:

- Physical examination and vital signs: weight, sitting blood pressure, sitting respiratory rate, sitting heart rate, axillary temperature
- Concomitant treatments (medications)
- Laboratory determinations: hematology, blood chemistry and coagulation assessments, urine dipstick
- Pregnancy test (females)
- Psychological assessments and tests
- $CD4^+$ T cell counts
- HIV-1 plasma viremia
- Anti-Tat antibodies (IgG, IgM, IgA)
- Lymphoproliferative response to Tat
- γIFN, IL-4 and IL-2 production in response to Tat
- Assessment of adverse events
- Samples collection and shipment (whole blood).

VACCINATION
The subject will be administered with the study medications allocated to him/her at randomization. Instruction for administration are reported in Section 4, Paragraph 4.1.1.
The following vaccination visit will be scheduled with a 4 weeks interval with the possibility to delay the vaccination of 1 week at maximum (+ 7 days).

Following To Vaccination
After administration of the study medication, subjects will remain at the study center for 2 hours for the following assessments:
- Physical examination and vital signs: weight, sitting blood pressure, sitting respiratory rate, sitting heart rate, axillary temperature
- Counseling on avoidance of pregnancy
- Assessment of adverse events.

During this period the subject will not assume any food.

DAY 84 (4th immunization for Arm A – first follow-up visit for Arm B)

The following activities will have to be performed for subjects randomized to both Arms A and B, except where specified.
Subjects randomized to Arm A, will perform the following determinations prior to immunization.

- Physical examination and vital signs: weight, sitting blood pressure, sitting respiratory rate, sitting heart rate, axillary temperature
- Concomitant treatments (medications)
- Laboratory determinations: hematology, blood chemistry and coagulation assessments, urine dipstick
- Pregnancy test (females, to be done only for Arm A)
- $CD4^+$ T cell counts
- HIV-1 plasma viremia
- Anti-Tat antibodies (IgG, IgM, IgA)

- Neutralization of Tat activity (the time-line of this determination could be rescheduled in relationship to the trend of the humoral immune response to Tat)
- Lymphoproliferative response to Tat
- γIFN, IL-4 and IL-2 production in response to Tat
- Assessment of adverse events
- Samples collection and shipment (whole blood).

<u>VACCINATION (only for Arm A)</u>
The subject will be administered with the study medications allocated to him/her at randomization. Instruction for administration are reported in Section 4, Paragraph 4.1.1.
The following vaccination visit will be scheduled with a 4 weeks interval with the possibility to delay the vaccination of 1 week at maximum (+ 7 days).

<u>Following To Vaccination (only for Arm A)</u>
After administration of the study medication, subjects will remain at the study center for 2 hours for the following assessments:
- Physical examination and vital signs: weight, sitting blood pressure, sitting respiratory rate, sitting heart rate, axillary temperature
- Counseling on avoidance of pregnancy
- Assessment of adverse events.

During this period the subject will not assume any food.

<u>DAY 112 (5th immunization only for Arm A – second follow-up visit for Arm B)</u>

The following activities will have to be performed for subjects randomized to both Arms A and B, except where specified.
<u>Subjects randomized to Arm A, will perform the following determinations prior to immunization.</u>

- Physical examination and vital signs: weight, sitting blood pressure, sitting respiratory rate, sitting heart rate, axillary temperature
- 12-lead ECG (to be done only for Arm B)
- Concomitant treatments (medications)
- Laboratory determinations: hematology, blood chemistry and coagulation assessments, urine dipstick
- Pregnancy test (females, to be done only for Arm A)
- Psychological assessments and tests
- $CD4^+$ T cell counts
- HIV-1 plasma viremia
- Anti-Tat antibodies (IgG, IgM, IgA)
- Assessment of adverse events
- Samples collection and storage (-80°C) at clinical site
- Samples collection and shipment (whole blood).

<u>VACCINATION (only for Arm A)</u>
The subject will be administered with the study medications allocated to him/her at randomization. Instruction for administration are reported in Section 4, Paragraph 4.1.1.

Following To Vaccination (only for Arm A)
After administration of the study medication, subjects will remain at the study center for 2 hours for the following assessments:
- Physical examination and vital signs: weight, sitting blood pressure, sitting respiratory rate, sitting heart rate, axillary temperature
- Counseling on avoidance of pregnancy
- Assessment of adverse events.

During this period the subject will not assume any food.

DAY 140 FOLLOW-UP POST-VACCINATIONS (Arm A and Arm B)

The following evaluations will be done:

- Physical examination and vital signs: weight, sitting blood pressure, sitting respiratory rate, sitting heart rate, axillary temperature
- Concomitant treatments (medications)
- Laboratory determinations: hematology, blood chemistry and coagulation assessments, urine dipstick
- $CD4^+$ T cell counts
- HIV-1 plasma viremia
- Anti-Tat antibodies (IgG, IgM, IgA)
- Lymphoproliferative response to Tat
- γIFN, IL-4 and IL-2 production in response to Tat
- Assessment of adverse events
- Samples collection and shipment (whole blood).

DAY 168 FOLLOW-UP POST-VACCINATIONS (Arm A and Arm B)

The following evaluations will be done:

- Physical examination and vital signs: weight, sitting blood pressure, sitting respiratory rate, sitting heart rate, axillary temperature
- Concomitant treatments (medications)
- Laboratory determinations: hematology, blood chemistry and coagulation assessments, urine dipstick
- 12-lead ECG (to be done only for Arm A)
- $CD4^+$ T cell counts
- HIV-1 plasma viremia
- Anti-Tat antibodies (IgG, IgM, IgA)
- Neutralization of Tat activity (the time-line of this determination could be rescheduled in relationship to the trend of the humoral immune response to Tat)
- Assessment of adverse events
- Samples collection and storage (-80°C) at clinical site
- Samples collection and shipment (whole blood).

DAY 336 FOLLOW-UP POST-VACCINATIONS (Arm A and Arm B)

The following evaluations will be done:

- Physical examination and vital signs: weight, sitting blood pressure, sitting respiratory rate, sitting heart rate, axillary temperature
- Concomitant treatments (medications)
- Laboratory determinations: hematology, blood chemistry and coagulation assessments, urine dipstick
- $CD4^+$ T cell counts
- HIV-1 plasma viremia
- Anti-Tat antibodies (IgG, IgM, IgA)
- Lymphoproliferative response to Tat
- γIFN, IL-4 and IL-2 production in response to Tat
- Assessment of adverse events
- Psychological assessments and tests
- Samples collection and shipment (whole blood).

5.10 Compliance

Study treatments will be administered only by study staff at clinical site.
The investigator will attach the vaccine tear-off label and to record the sterile water code and batch number on the appropriate form, recording the date and time of administration. It will be investigators' responsibility to conserve used, unused and partially used vials until they are returned to Injectalia Srl by the Clinical Trial Monitor.
The treatment compliance will be verified by the Clinical Trial Monitor in occasion of each monitoring visit and drug accountability records will be filled in accordance to the CRO SOP.
The subject will be reminded of the importance of attending the planned visit timeline as strictly as possible.

6. STUDY INTERRUPTION

6.1 Premature Discontinuation of the Treatment/Study per Subject

Treatment may be withdrawn at any time, when any of the following conditions occurs:
- voluntary discontinuation. Subjects are free at any time to discontinue study participation, without prejudice to further treatment
- safety reasons as judged by the Investigator and/or Sponsor and/or DSMB
- severe non-compliance to the protocol as judged by the Investigator and/or ISS
- incorrect enrolment of the subject (unless the Sponsor's agreement)
- subject lost to follow-up.

The Investigator should determine the primary reason for premature discontinuation of the treatment/study and record it in the case report form.

The following specific events will necessarily determinate the discontinuation of the study treatment: (1) Pregnancy; (2) Grade IV systemic event classified as "probably" or "definitely associated" with immunization; (3) Grade IV local adverse event classified as "probably" or "definitely associated" with immunization; (4) Type 1 hypersensitivity associated with immunization; (5) Serious intercurrent illness that is not expected to resolve prior to the next scheduled immunization.

Note: Grade IV systemic reactions are defined in Appendix I (http://www.who.int/hiv/pub/guidelines/artadultguidelines.pdf) - Annex 7 - Division of AIDS/Table for Grading Severity of Adult Adverse Experiences

In any case the investigator will make all efforts in order to maintain the subject in the study until the end of the follow-up period (week 48); if not possible, a final evaluation will be made at the time of study interruption. In case of treatment or study interruption, the subject will be treated in accordance with the investigator's clinical judgment.

In case a participant misses a scheduled study visit, the study staff will try to establish communication with the subject through all possible means (e.g., writing and telephoning the participants and his/her contacts) and to reschedule the vaccination visit within 7 days; after this period the subject will be excluded from the treatment, on the basis of a scarce compliance to the protocol.
The need to attend all scheduled study visits will be emphasized at each visit.

6.2 Premature Discontinuation of the Study in a Clinical Site

ISS may stop this trial in a clinical site for any of the following reasons:

- the site cannot include an adequate number of subjects
- serious and/or persistent non-compliance with the protocol
- careless or premeditated false documentation in the CRFs
- inadequate co-operation with the Sponsor
- non-compliance with GCP, SOPs or regulatory requirements
- the investigator asks to discontinue the trial
- lack of confidentiality and/or non compliance with the contract spread with the Sponsor.

If the trial is prematurely terminated or suspended for any reason, the subjects will be informed promptly, appropriate therapy and follow-up will be assured and relevant regulatory authorities will be informed. The Local Ethical Committee/Central Ethical Committee will be promptly informed and provided with a detailed written explanation.

6.3 Premature Discontinuation of the Whole Study

If the trial is prematurely terminated or suspended, ISS will promptly inform the investigators/institutions, and the regulatory authorities providing reasonable explanations according to the cGCP. A written explanation will be promptly sent to the Local Ethical Committee/Central Ethical Committee (LEC/CEC) by ISS or the investigator/institution, as specified in the regulatory requirements.
All the subjects involved in the study will be informed promptly if the whole trial is prematurely terminated or suspended and appropriate therapy and follow-up will be assured.

7. ENDPOINTS

7.1 Primary Endpoint

The primary endpoint of this Phase II vaccine trial is to qualify Tat protein as immunogenic in HIV-1 infected adult subjects, anti-Tat antibody negative, HAART-treated, with chronic suppressed HIV-1 infection: $CD4^+$ T cell counts $\geq$ 400 cells/µl, levels of plasma viremia < 50 copies/ml in last 6 months prior to the screening and without a history of virologic rebound.

7.2 Primary Endpoint Variables and Measurements

Evaluation of immunogenicity will include:

1. Induction of specific anti-Tat humoral immune response in terms of IgM, IgG, IgA anti-Tat antibodies. Assessment of antibody neutralization of Tat activity in vitro (inhibition of Tat-induced HIV rescue assay).
2. Induction or increase of anti-Tat cellular-mediated immune response, in terms of lymphoproliferative response to Tat (CFSE staining), in vitro γIFN, IL-4, IL-2 production (Elispot) by peripheral blood mononuclear cells (PBMC) in response to Tat.

The Primary Endpoint will be measured by the induction, magnitude and persistence of the humoral and cellular immune responses to Tat and by comparing the immunogenicity of a 3 or a 5 immunization schedule of the two different vaccine doses (7.5 µg and 30 µg) at week 24 and week 48.

The induction of the anti-Tat specific humoral immune response will be evaluated as follows:

a) Percentage of "responders" defined as those vaccinees that develop an immune response above the cut-off level;
b) Geometric mean antibody titers (GMT) and pre/post vaccination ratios.

Responders will be defined as those immunized subjects that develop at least one of the following responses, at one or more time point after immunization:

- Anti-Tat IgM titers ≥ 25
- Anti-Tat IgG titers ≥ 100
- Anti-Tat IgA titers ≥ 25.

The induction of the anti-Tat specific cellular immune response will be evaluated as follows:

a) Percentage of vaccinees with a positive cellular immune response to vaccination (responders);
b) Increase of anti-Tat cellular-mediated immune response, evaluated in terms of fold/spots/proliferation index.

Responders will be defined as those immunized subjects that develop immune responses, at one or more time points after immunization, above the following thresholds:

- γIFN ≥ 3 fold and 30 spots/10^6 cells
- IL-2 ≥ 3 fold
- IL-4 ≥ 3 fold
- Proliferation index ≥ 1.

The evaluation of Tat-specific immune responses will be performed by the Core Laboratory of Immunology and Virology (San Gallicano Hospital, Rome).
All data generated by immunologic testing performed by the core lab will be recorded on the Core Laboratory CRFs (CL-CRFs) and will be sent to the clinical sites.

The following laboratory results will be provided, within 15-20 days after blood withdrawal by the

Core Laboratory to the corresponding clinical site during the course of the study:

- determination of IgM, IgG and IgA anti-Tat antibodies in sera
- Titration of IgM, IgG and IgA anti-Tat antibodies
- Lymphoproliferative response to HIV-1 Tat (CFSE staining)
- *In vitro* γIFN, IL-4 and IL-2 production in response to Tat (Elispot)
- HIV-1 plasma viremia (viral RNA copies).

The following laboratory results will be provided by the Core Laboratory to the corresponding clinical site within 20 days after Study Visit 8:

- Neutralization of Tat activity by in vitro assays (rescue inhibition assay).

7.3 Secondary Endpoints

The secondary endpoints of this Phase II vaccine trial are to qualify the Tat protein as safe.

7.4 Secondary Endpoints Variables and Measurements

Assessment of vaccine safety will include clinical observation and monitoring of hematological, biochemical, virological and immunological parameters. Safety will be further evaluated by monitoring the local and systemic adverse reactions occurring during the course of the trial.
After administration of the study medication, subjects will remain at the clinical site for 2 hours. Local reactogenicity and early adverse events will be evaluated during the first 2 hours after each immunization. Vital signs and physical examination will be re-assessed just before subjects are allowed to leave the study center.

The evaluation of Secondary Endpoints will be performed at weeks 24 and 48 to compare subjects vaccinated in Arm A and at weeks 16 and 48 when comparing volunteers vaccinated in Arm B. Comparisons between study Arms will also be performed between Arm A-Group I (5 doses of Tat 7.5 μg) at week 24 versus the Arm B-Group I (3 doses of Tat 7.5 μg) at week 16 as well as between Arm A-Group II (5 doses of Tat 30 μg) at week 24 versus the Arm B-Group II (3 doses of Tat 30 μg) at week 16. Such comparison will be made with regard to frequency and grading of adverse events, including any significant change in hematological/biochemical laboratory parameters.

8. TOLERABILITY

Female participants will be cautioned of the unknown risk of study vaccines to the fetus and will be advised to use adequate birth control methods for one month prior to immunization and for the whole the duration of the study.

8.1 Adverse Events Definitions

8.1.1 Adverse Event (AE)

An AE is any untoward medical occurrence in a patient or clinical trial subject administered a medicinal product and which does not necessarily have a causal relationship with the experimental treatment.

AEs include any illness, sign (e.g. tachycardia, enlarged liver, etc.), symptom (e.g. nausea, chest pain, etc.) or clinically significant laboratory test abnormality (e.g. laboratory findings, electrocardiogram, etc.) that has appeared or worsened during the course of the clinical trial at any time, including run-in or wash out periods, regardless of causal relationship to the drug(s) under study, even if no study treatment has been administered.

8.1.2 Serious Adverse Event (SAE)

A SAE is any untoward medical occurrence that:

- Results in death
- Is life-threatening (defined as an event in which the subject or patient was at risk of death at the time of the event; it does not refer to an event which hypothetically might have caused death if it were more severe)
- Requires inpatient hospitalization or prolongation of existing hospitalization
- Results in persistent or significant disability/incapacity
- Is a congenital anomaly/birth defect
- Is an important medical event [defined as a medical event that may not be immediately life-threatening or result in death or hospitalization but, based upon appropriate medical and scientific judgment, may jeopardize the patient/subject or may require intervention (e.g. medical, surgical) to prevent one of the other serious outcomes listed in the definition above]. Examples of such events include, but are not limited to, intensive treatment in an emergency room or at home for allergic bronchospasm, blood dyscrasias or convulsions that do not result in hospitalization.

8.1.3 Adverse Drug Reaction (ADR)

An ADR is any untoward and unintended responses to an investigational medicinal product related to any dose administered in clinical trials that means that the Investigator or the Sponsor has judged that there is at least a reasonable possibility that the event was related to the product.

8.1.4 Serious Adverse Drug Reaction (SADR)

A SADR is a SAE suspected to be causally related to the investigational product.

8.2 Reporting of Adverse Events/Adverse Drug Reactions

The identification and reporting of AEs/ADRs is an Investigator's responsibility. The collection of AE/ADR information will begin as soon as the study starts at each investigational center (when the first subject is enrolled).

AEs/ADRs may be either spontaneously reported or elicited during questioning and examination of a subject. All identified AEs/ADRs must be recorded and described on the appropriate page of the CRF in occasion of each visit scheduled within the clinical trial. If known, the diagnosis of the underlying illness or disorder should be recorded, rather than its individual symptoms.

Any clinically significant changes noted during interim or final physical examinations, electrocardiograms, hematological-biochemical-coagulation analysis, urinalysis and any other potential safety assessments, whether or not these procedures are required by the protocol, should also be recorded on the appropriate AE page of the CRF.

Grades of severity will be recorded according to the National Institutes of Health (NIH) Division of AIDS Adult AIDS Clinical Trials Group - Table for Grading Severity of Adult Adverse Experiences provided in Appendix I.
The investigator will be requested to provide a relationship assessment between the AE and the study drug, using the following criteria:

Code	Descriptor	Definition
5	Certain	The AE is clearly related to the investigational product(s)
4	Probable	The AE is likely related to the investigational product(s)
3	Possible	The AE may be related to the investigational product(s)
2	Unlikely	The AE is doubtfully related to the investigational product(s)
1	Unrelated	The AE is clearly not related to the investigational product(s)

All AEs and ADRs should be followed until resolution or stabilization and reported as SAEs/SADRs if they become serious.
The investigator will provide or arrange for appropriate follow-up (if required) for such subjects, and document the course of the subject's condition.

It is requested to the Investigator to communicate to the CRO within 7 working days any Non Serious Adverse Event and Non Serious Adverse Drug Reaction which causes the subject withdrawal from the study or the interruption (temporary or definitive) of the study treatment.
Such AEs/ADRs have to be reported to the CRO by filling the appropriate section of the electronic CRF and by sending an e-mail or fax warning to the CRO Opera.

8.3 Reporting of Serious Adverse Events/Serious Adverse Drug Reactions

The Investigator must immediately notify Opera of any Serious Adverse Event/Serious Adverse Drug Reaction, which should occur while the study is ongoing (until week 48), as soon as he/she acknowledges it.
In case a subject discontinues the study during the 8 weeks or 16 weeks of treatment it will be responsibility of the Investigator to make all efforts to report any SAE/SADR which should occur to the subject within 30 days after treatment interruption, as soon as he/she acknowledges it. In addition, deaths which occur more than 30 days after stopping study drugs need to be reported immediately on an SAE form only if they are consequent to a SAE which developed during the study or within 30 days from treatment interruption, and which did not resolve before the death.
Exception is made for all Serious Adverse Events identified as strictly related to the progression of AIDS disease. All these Serious Adverse Events should be recorded on the CRF but do not need immediate notification.
SAE/SADR notifications have to be made, according to the training and procedures manual that will be provided to all Investigators, by telephone, fax or e-mail and has to be promptly followed by a detailed written report (Serious Adverse Event (SAE)/ Serious Adverse Drug Reaction (SADR) Report).

Emergency contacts for SAE/SADR notification:
OPERA Srl:
Tel: +39 010 4699623
Fax: +39 010 6591120
Mobile: +39 329 0557770
E-mail: trials@operacro.com

It will be Opera responsibility to inform immediately ISS and the DSMB of any SAE/SADR.

In case further information is available, after the first notification, the Investigator must provide a follow-up report to Opera as soon as such further information is available.
If an autopsy was performed, a copy of the autopsy report should be provided when it becomes available.
All Serious Adverse Events/Serious Adverse Drug Reactions shall be also registered in the CRF relevant section in occasion of each visit scheduled within the clinical trial.

As required, the Sponsor will notify all the Suspected Unexpected Serious Adverse Reactions (SUSARs) to the involved LECs in accordance with in force regulations.
Investigators will be informed about all AEs that are serious, unexpected, and certainly, probably, possibly, unlikely related to the investigational product, through periodic safety reports.

8.4 Subjects' Protection

The Sponsor will provide the overall operational directions for the study and will be responsible for the trial conduction according to the highest scientific and ethical standards.
The Sponsor will provide for the constitution of the Data Safety Monitoring Board (DSMB) and the Community Advisory Board (CAB) that will cooperate with the Sponsor for the study oversight and management.
It will be responsibility of the DSMB to assess the safety of the study vaccine during the trial. The members of the Committee will not have any direct involvement in the study. The DSMB will monitor the trial for evidence of adverse effects of the study vaccine and may recommend any action to ensure the safety of study participants.
The Sponsor and the DSMB will monitor the progress of the trial. They will review data from the trial with particular emphasis on adverse reactions, including also the following:

A. Local reactogenicity at the site of injection: e.g., pain, tenderness, erythema, inflammation, angiogenesis, induration, regional lymphadenopathy, limitation of limb movement;
B. Systemic symptoms: fever, myalgia, fatigue, and headache; anaphylaxis, immune complex disease, and other hypersensitivity reactions;
C. Hematology: WBC with differential, platelets;
D. Hepatic/renal: ALT, creatinine, urinalysis;
E. $CD4^+$ lymphocyte counts;
F. Plasmatic HIV-1 RNA;
G. Other reactions: dermatologic, neurologic, gastrointestinal (nausea/vomiting, diarrhea), psychological (stress, phobia);
H. Changes in attitudes with friends or family, or other unanticipated social impact;
I. Risk behavior.

The DSMB will evaluate all SAEs occurred during the trial, in particular those "clearly" or "probably" related to vaccination, and will have the faculty of requesting changes to the protocol or the study interruption, in order to preserve subjects' safety.

The CAB will represent the interests and concerns of all communities of individuals affected by HIV/AIDS. The primary role of the CAB will be to integrate patients community into the clinical trial process in order to advance HIV/AIDS research. The CAB will also contribute in educating the public about the importance of the participation in clinical trials.

9. DATA RECORDING, MONITORING AND DATA MANAGEMENT

9.1 Data Recording

Investigators are required to prepare and maintain adequate and accurate case histories designed to record all observations and other data pertinent to the investigation on each individual entered into the study. Data reported on the electronic Case Report Form (e-CRF) must be consistent with the source documents.
The subjects will be asked to participate to a psychological assessment. The evaluations provided by the psychological protocol could be performed both by electronic or paper questionnaires according to the site facilities. In any case such questionnaires have to be considered as source documents.
Core Laboratory data will be recorded on a CL-CRF and then printed, signed and dated in original by the Core Lab responsible. After that, Core Laboratory reports will be sent to the Investigators, who will have the responsibility of recording the necessary data on the e-CRF.

9.2 Data Monitoring

This trial will be conducted in accordance with the principles of Good Clinical Practice.
Before clinical trial initiation, Opera personnel will visit the clinical sites to:

- determine the adequacy of the facilities
- discuss with the investigator(s) (and other personnel involved in the study) their responsibilities with regard to protocol adherence, and the responsibilities of Sponsor or its representatives.

During the study, a Clinical Trial Monitor will have regular contacts with clinical sites, including visits to:

- provide information and support to the investigator(s)
- confirm that facilities remain acceptable
- confirm that the investigational team is adhering to the protocol, that data are being accurately recorded in the electronic case report forms, and that investigational product accountability checks are being done
- perform source data verification (a cross-check between data recorded in e-CRF and subject's medical records at the hospital, and/or other records relevant to the study). This will require direct access to all original data for each subject (e.g. clinical records).

9.3 Data Management

Data verification will be performed by Opera.

Subjects will be identified by initials, birth date and subject number. All requested information must be entered on the e-CRF in the provided spaces. If an item is not available or is not applicable, it must be documented as such.
The confidentiality of records that could identify subjects must be protected, respecting the privacy and confidentiality rules in accordance with the applicable regulatory requirement(s).

The Investigator will maintain a Signature Sheet to document signatures and initials of all persons authorized to make entries and/or corrections on e-CRFs. A data correction will be made only by the authorized persons (persons who are authorized to access to the e-CRF by a specific user ID and

password). The e-CRF system will record the date of any correction, and the name of the person making the correction. The system will not obscure the original entry.

The e-CRFs for this protocol will be formatted in a sequence of modules, which will correspond to the various study periods through which study subjects will progress. Upon completion of each scheduled study visit, the appropriate e-CRF module will be accessible to ISS and Opera through the Remote Data Capture system, for rapid data processing.

10. STATISTICAL ANALYSIS

10.1 Statistical Program

Statistical analyses and data processing will be performed using SAS software for Windows™.

10.2 Parameters

Primary Efficacy Variables

- IgM, IgG, IgA anti-Tat antibodies induction as specific anti-Tat humoral response; neutralization of Tat activity by in vitro tests (inhibition of Tat-induced HIV rescue assay)
- lymphoproliferative response to Tat (CFSE staining), in vitro γIFN, IL-4, IL-2 production in response to Tat (Elispot).

Safety Variables

- adverse events, including any significant change in hematological/biochemical laboratory parameters.

10.3 Sample Size Determination

As the primary objective is to assess the immunogenicity of the Tat vaccine, sample size has been estimated separately in each vaccination arm. To observe a proportion of at least 80% of subjects with positive humoral immune responses to vaccination, taking into account a maximum margin of error of 15% and a confidence level of 95%, 28 valuable subjects are required in each vaccination group. With this sample size a difference of at least 35% of positive immune response between vaccination groups will be detected as statistically significant.
In the hypothesis that the drop-out rate will be no greater then nearly 10%, 128 subjects will be enrolled, 32 for each vaccination group.

Such a sample size will allow to test some additional hypothesis. In fact, with 28 subjects in each vaccination group, a difference of 0.5 $\log_{10}$ anti-Tat antibodies after the treatment period between each of the two vaccination groups, assuming a common standard deviation of 0.65 $\log_{10}$, will be detectable with a significance level of 5% and a power of 80%.
Analogously, a difference of 50 CD4+ T-cell counts/μl after the treatment period between each of the two vaccination groups, assuming a common standard deviation of 65 cells/μl, will be detectable with a significance level of 5% and a power of 80%.

10.4 Population for Statistical Evaluation

Two groups of subject populations will be considered for statistical analysis.
The immunogenicity population, representing all randomized subjects who received at least 3 immunizations and have at least one post-baseline immunogenicity evaluation.
The safety population, representing all randomized subjects who received at least one administration of the vaccine.

10.5 Analytical Methods

All descriptive statistics used to summarize numeric data will include mean values, standard deviation, median, minimum and maximum. Frequency distributions will be presented for categorical variables.
95% confidence intervals will be determined for all data.
All statistical tests will be performed at two-sided with a 5% significance level.
One interim analysis (week 24) and a final analysis (week 48) will be carried out.

10.6 Primary Endpoint Analysis

To evaluate the humoral immune response to vaccination, "responders" will be defined as those immunized subjects that develop an immune response above the cut-off level. Risk to be "responder" will be compared between groups using a logistic regression model (Odds ratio).
In addition geometric mean antibody titers (GMT) and pre/post-vaccination ratios will be calculated and compared between treatment groups.
Mean changes from baseline of antibody titers will be evaluated within and between treatment groups by the ANOVA model.
Subjects with a cellular immune response will be analyzed and compared between arms using the Mantel-Haenszel Chi-Square test.

10.7 Secondary Endpoints Analysis

Safety will be assessed in terms of adverse events and laboratory measurements.
MedDRA dictionary will be used for coding adverse events. Adverse events will be summarized by body system, preferred term, severity and relationship with the vaccination; the incidence of adverse events "possibly", "likely" or "clearly" related to vaccination will be computed and compared between the four vaccination regimens to estimate the relative risk. All serious adverse events (SAEs) will be summarized by subject, including gender, age, duration of the event, action taken, investigator's assessment of causality and outcome.
Statistics on changes from baseline at the several time points during the study will be provided for each hematological and biochemical parameter. Incidence of clinically significant changes in laboratory values will also be evaluated and compared by arms.

In addition, mean changes from baseline of $CD4^+$ T cells and HIV viral load will be evaluated within and between treatment groups by the ANOVA model; mean changes from baseline of lymphocytes phenotype values will also be performed.

11. ETHICAL AND ADMINISTRATIVE PROCEDURES

11.1 Informed Consent

Preparation of the consent form is the responsibility of the Sponsor in cooperation with CAB and must include all elements required by ICH, GCP and applicable regulatory requirements, and must adhere to GCP and to the ethical principles that have their origin in the Declaration of Helsinki (Appendix IV). The consent form must also include a statement that ISS, its delegates, and regulatory authorities have direct access to subject records. Prior to the beginning of the study, the Investigator must have the CEC/LEC's written approval/favorable opinion of the written informed consent form and any other information to be provided to the subjects.

The Investigator must provide the subject with a copy of the consent form and written information about the study in a format that is non-technical and it easily understood. The Investigator should allow the necessary time to the subject to inquire about the details of the study, then informed consent must be signed and personally dated by the subject and by the person who conducted the informed consent discussion. The subject should receive a copy of the signed informed consent and any other written information provided for the study subjects prior to subject's participation to the trial.

The informed consent and any other information provided to the subjects, should be revised whenever important new information becomes available that is relevant to the subject's consent, and should receive CEC/LEC approval/favorable opinion prior to use. The Investigator, or a person designated by the Investigator, should fully inform the subject of all pertinent aspects of the study and of any new information relevant to the subject's willingness to continue participation in the study. This communication should be documented.
During a subject's participation in the trial, any updates to the consent form and any updates to the written information will be provided to the subjects.

11.2 Ethical and Administrative Issues

This study will be conducted in accordance with the ethical principles that have their origin in the Declaration of Helsinki (as amended by the 52nd World Medical Association (WMA) General Assembly in October 2000 with Note of Clarification on paragraph 29 added by the WMA General Assembly, Washinghton 2002 and Note of Clarification on paragraph 30 added by the WMA General Assembly, Tokyo 2004) and will be consistent with Good Clinical Practice (GCP) and applicable regulatory requirements.

The study will be conducted in compliance with the protocol.
All revisions to the protocol must be discussed with, and prepared by, the Sponsor. The Investigator should not implement any deviation or change to the protocol without prior review and documented approval/favorable opinion from the CEC/LEC of an Amendment, except where necessary to eliminate an immediate hazard(s) to study subjects. Any significant deviation must be documented in the e-CRF.
If a deviation or change to the protocol is implemented to eliminate an immediate hazard(s), as soon as possible the deviation or change will be communicated to DSMB for its advice and submitted to:

- ISS Ethical Committee;
- Local Ethical Committee/Central Ethical Committee for review and approval/favorable opinion.

Documentation of approval signed by the chairperson or designee of the CEC/LEC must be sent to ISS.
If an Amendment substantially alters the study design or increases the potential risk to the subject: (1) the consent form must be revised and submitted to the CEC/LEC for review and approval/favorable opinion; (2) the revised form must be used to obtain consent from subjects currently enrolled in the study if they are affected by the Amendment; and (3) the new form must be used to obtain consent from new subjects prior to enrolment.

Neither study drug nor study materials will be provided to clinical sites until all the necessary authorizations are obtained.
All the ethical and administrative procedures for study initiation will be managed by the CRO.

11.3 Subjects Data Protection – Direct Access to Source Data

The right, safety and well-being of the trial subjects are the most important considerations and should prevail over interests of science and society.
Study personnel involved in conducting this trial will be qualified by education, training, and experience to perform their respective tasks.
This trial will not use the services of study personnel where sanctions have been invoked or where there has been scientific misconduct or fraud (e.g., loss of medical licensure, debarment).
Systems with procedures will be implemented to assure the quality of every aspect of the study.
ISS, its delegates, and regulatory authorities have direct access to subject records and for the protection of the enrolled subjects, this study will be conducted respecting the privacy and confidentiality rules, in accordance with the applicable regulatory requirements.

11.4 Insurance, Indemnity and Refunds

ISS's liability will be covered by a liability insurance policy with HDI GERLING, Policy number: 63/108172/16.

With respect to any liability directly or indirectly caused by the investigational products in connection with this clinical study, ISS assumes liability by law on behalf of the investigator(s) and his assistants for possible injury to the subject provided the investigator(s) and his/her assistants have followed the instructions of ISS in accordance with this protocol and any amendments thereto, that the investigational products administered to the subject in this clinical study have been supplied by ISS and that the investigator and his/her assistants have in general performed this clinical study in accordance with good scientific and clinical practice and currently acceptable techniques and know-how.

All medical costs (including new tests ordered as a result of abnormal results) will be covered by the Public Health System.
ISS will cover costs for the centralized blood analysis, samples shipments and study materials (study drugs, e-CRF, kits for blood withdrawal and sample collection).

11.5 Audits and Inspections

Authorized representatives of ISS, a regulatory authority, CEC/LEC may visit the center to perform audits or inspections, including source data verification. The purpose of an ISS audit or inspection is to systematically and independently examine all study related activities and documents to determine whether these activities were properly conducted, and data were recorded, analyzed, and accurately reported according to the protocol, Good Clinical Practice (GCP), the guidelines of the International Conference on Harmonisation (ICH), and any applicable regulatory requirements. The investigator should contact ISS immediately if contacted by a regulatory agency about an inspection at his/her center.

11.6 Training to Study Staff

The principal investigator will maintain a record of all individuals involved in the study (medical, nursing and other staff). He/She will ensure the appropriate training relevant to the study is given to all of the staff, and that any new information of relevance to the performance of this study is forwarded to the staff involved.

11.7 Records Retention

The Investigator must retain investigational product disposition records, copies of CRFs (or electronic files), and source documents for the maximum period required by applicable regulations and guidelines, or Institution procedures, or for the period specified by the ISS, whichever is longer.
The Investigator must contact ISS prior to destroy any records associated with the study.
ISS will notify the Investigator when the trial records are no longer needed.
If the Investigator withdraws from the study (e.g., relocation, retirement), the records shall be transferred to a mutually agreed designee (e.g., another Investigator, LEC). Notice of such transfer will be given in writing to ISS.

12. CASE REPORT FORMS

This study will use electronic Case Report Forms based on Oracle Clinical data base.
Different access levels will be allowed to Sponsor/CRO and to clinical sites.
Each clinical site will be allowed to see its own data and not to see data from other participating sites.
Users will identify themselves by signing on with a database user ID and password.
The database user ID determines the user's security role such as "Data Manager", "CTM", "Investigator".

13. PUBLICATION OF RESULTS

All information regarding this trial obtained as a result of the study will be regarded as confidential.
The Investigators agree that scientific results from this study are to be considered the property of ISS.
Information regarding the operations and procedures of ISS obtained as a result of or in association with the conduct of this study must be kept confidential.
Unpublished information contained herein, as well as any information received from the Sponsor

for the purposes of this study, may not be disclosed to any third party without the prior written approval of the Sponsor.
No data will be used for scientific meetings and/or publication in scientific journals without prior written authorization from the Sponsor.
Any publication of data from an individual center in this trial will be allowed only after the study is concluded and data are published and upon written ISS approval.

ISS reserves, the right to use the results obtained as documentary and scientific backing in proceedings regarding the Regulatory Authorities and/or for updating their own staff.
ISS is committed to publication of results after study conclusion.

14. STUDY TIMETABLE

The enrolment of the first subject is planned on April 2008.
The enrolment period will last until case number is reached.
The investigators are expected to make every reasonable effort to recruit suitable subjects to the study.
If the enrolment rate will be low, if a significant number of protocol violators are recruited or if no subjects are recruited within a reasonable period, it will be ISS faculty to prematurely close the enrolment or interrupt the study at one or more clinical sites.

15. INVESTIGATOR'S AGREEMENT

A PHASE II RANDOMIZED, OPEN LABEL, IMMUNOGENICITY AND SAFETY TRIAL OF THE VACCINE BASED ON THE RECOMBINANT BIOLOGICALLY ACTIVE HIV-1 TAT PROTEIN IN ANTI-TAT NEGATIVE HIV-1 INFECTED HAART-TREATED ADULT SUBJECTS

Protocol Number: ISS T-002

Issue Date: 12th February 2008

Sponsor Representatives

Barbara Ensoli, MD, PhD		
**AIDS National Center**	Signature	Date

Istituto Superiore di Sanità
Viale Regina Elena, 299
00161 - Rome, Italy

Clinical site - Principal Investigator

I have carefully read this protocol and agree to conduct the study in accordance with GCP, Declaration of Helsinki, local laws and regulations relevant to the use of new and approved therapeutic agents in human subjects.
I agree that ISS, its delegates and Regulatory Authorities have direct access to all study documentation.
I agree to obtain Written Informed Consent from all participating subjects or their legal representative.
I agree to maintain the confidentiality of all information received or developed in connection with this protocol.

Name	Signature	Date

(typed or printed)
Institute address:
(typed or printed)

16. REFERENCES

Arora VK, Fredericksen BL, Garcia JV. Nef: agent of cell subversion. Microbes and Infections 2002, 4:189-199.

Arya SK, Guo C, Josephs SF, Wong-Staal F. Trans-activator gene of human T-lymphotropic virus type III (HTLV-III). Science 1985, 229:69-73.

Barillari G, Gendelman R, Gallo RC, Ensoli B. The Tat protein of human immunodeficiency virus type 1, a growth factor for AIDS Kaposi sarcoma and cytokine-activated vascular cells, induces adhesion of the same cell types by using integrin receptors recognizing the RGD amino acid sequence. Proc Natl Acad Sci USA 1993, 90:7941-7945.

Barillari G, Sgadari C, Palladino C, Gendelman R, Caputo A, Morris CB, Nair BC, Markham P, Nel A, Stürzl M, Ensoli B. Inflammatory cytokines synergize with the HIV-1 Tat protein to promote angiogenesis and Kaposi's sarcoma via induction of basic fibroblast growth factor and the alpha v beta 3 integrin. J Immunol 1999a, 163:1929-1935.

Barillari G, Sgadari C, Fiorelli V, Samaniego F, Colombini S, Manzari V, Modesti A, Nair BC, Cafaro A, Stürzl M, Ensoli B. The Tat protein of human immunodeficiency virus type-1 promotes vascular cell growth and locomotion by engaging the alpha5beta1 and alphavbeta3 integrins and by mobilizing sequestered basic fibroblast growth factor. Blood 1999b, 94:663-672.

Burton DR. A vaccine for HIV type 1: the antibody perspective. Proc Natl Acad Sci USA 1997, 94:10018-10023.

Buttò S, Fiorelli V, Tripiciano A, Ruiz-Alvarez MJ, Scoglio A, Ensoli F, Ciccozzi M, Collacchi B, Sabbatucci M, Cafaro A, Guzmán CA, Borsetti A, Aiuti F, Vardas E, Colvin M, Lukwyia M, Rezza G, Ensoli B, the Tat Multicentric Study Group. Recognition of a clade B-derived HIV-1 Tat protein by antibodies of infected individuals from Uganda, South Africa and Italy is associated with conservation of Tat epitopes in the infecting viruses Cross-Recognition of the Clade B HIV-1 Tat Protein Vaccine Candidate by Antibodies from HIV-1-Infected Italian, Ugandan and South African Individuals. J Infect Dis 2003, 188:1171-1180.

Cafaro A, Caputo A, Fracasso C, Maggiorella MT, Goletti D, Baroncelli S, Pace M, Sernicola L, Koanga-Mogtomo ML, Betti M, Borsetti A, Belli R, Akerblom L, Corrias F, Buttò S, Heeney J, Verani P, Titti F, Ensoli B. Control of SHIV-89.6P-infection of cynomolgus monkeys by HIV-1 Tat protein vaccine. Nat Med 1999, 5:643-650.

Cafaro A, Caputo A, Maggiorella MT, Baroncelli S, Fracasso C, Pace M, Borsetti A, Sernicola L, Negri DR, Ten Haaft P, Betti M, Michelini Z, Macchia I, Fanales-Belasio E, Belli R, Corrias F, Buttò S, Verani P, Titti F, Ensoli B. SHIV89.6P pathogenicity in cynomolgus monkeys and control of viral replication and disease onset by human immunodeficiency virus type 1 Tat vaccine. J Med Primatol 2000, 29:193-208.

Cafaro A, Titti F, Fracasso C, Maggiorella MT, Baroncelli S, Caputo A, Goletti D, Borsetti A, Pace M, Fanales-Belasio E, Ridolfi B, Negri DR, Sernicola L, Belli R, Corrias F, Macchia I, Leone P, Michelini Z, Ten Haaft P, Buttò S, Verani P, Ensoli B. Vaccination with DNA containing tat coding sequences and unmethylated CpG motifs protects cynomolgus monkeys upon infection with simian/human immunodeficiency virus (SHIV89.6P). Vaccine 2001, 19:2862-2877.

Caputo A, Gavioli R, Ensoli B. Recent advances in the development of HIV-1 Tat-based vaccines. Curr HIV Res 2004, 2:357-376.

Casanova-Sotolongo P, Casanova-Carrillo P. Association of peripheral facial paralysis in patients with human immunodeficiency virus infection. Rev Neurol 2001, 32(4):327-330.

Caselli E, Betti M, Grossi MP, Balboni PG, Rossi C, Boarini C, Cafaro A, Barbanti-Brodano G, Ensoli B, Caputo A. DNA immunization with HIV-1 tat mutated in the trans

activation domain induces humoral and cellular immune responses against wild-type Tat. J Immunol 1999, 162:5631-5638.

Chang HC, Samaniego F, Nair BC, Buonaguro L, Ensoli B. HIV-1 Tat protein exits from cells via a leaderless secretory pathway and binds to extracellular matrix-associated heparan sulfate proteoglycans through its basic region. AIDS 1997, 11:1421-1431.

Demirhan I, Chandra A, Sarin PS, Hasselmayer O, Hofmann D, Chandra P. Inhibition of tat-mediated HIV-1-LTR transactivation and virus replication by sulfhydryl compounds with chelating properties. Anticancer Res 2000, 20:2513-2517.

Doutre MS, Beylot C, Beylot J, Long P, Royer P, Lacoste D. Peripheral facial paralysis and HIV infection. 2 case reports. Rev Med Interne 1992, 13(5):354-358.

Ensoli B, Barillari G, Salahuddin SZ, Gallo RC, Wong-Staal F. Tat protein of HIV-1 stimulates growth of cells derived from Kaposi's sarcoma lesions of AIDS patients. Nature 1990, 345:84-86.

Ensoli B, Barillari G, Gallo RC. Cytokines and growth factors in the pathogenesis of AIDS-associated Kaposi's sarcoma. Immunol Review 1992, 127:147-155.

Ensoli B, Buonaguro L, Barillari G, Fiorelli V, Gendelman R, Morgan RA, Wingfield P, Gallo RC. Release, uptake, and effects of extracellular human immunodeficiency virus type1 Tat protein on cell growth and viral transactivation. J Virol 1993, 67:277-287.

Ensoli B, Gendelman R, Markham P, Fiorelli V, Colombini S, Raffeld M, Cafaro A, Chang HK, Brady JN, Gallo RC. Synergy between basic fibroblast growth factor and HIV-1 Tat protein in induction of Kaposi's sarcoma. Nature 1994, 371:674-680.

Ensoli B, Fiorelli V, Ensoli F, Cafaro A, Titti F, Buttò S, Monini P, Magnani M, Caputo A, Garaci E. Candidate HIV-1 Tat vaccine development: from basic science to clinical trials. AIDS 2006, 20:2245-2261.

Fanales-Belasio E, Moretti S, Nappi F, Barillari G, Micheletti F, Cafaro A, Ensoli B. Native HIV-1 Tat protein targets monocyte-derived dendritic cells and enhances their maturation, function and antigen-specific T cell responses. J Immunol 2002, 168:197-206.

Ferrantelli F, Cafaro A, Ensoli B. Nonstructural HIV proteins as targets for prophylactic or therapeutic vaccines. Curr Opin Biotechnol. 2004 Dec,15(6):543-556.

Fiorelli V, Gendelman R, Samaniego F, Markham PD, Ensoli B. Cytokines from activated T cells induce normal endothelial cells to acquire the phenotypic and functional features of AIDS-Kaposi's sarcoma spindle cells. J Clin Invest 1995, 95:1723-1734.

Fisher AG, Feinberg MB, Josephs SF, Harper ME, Marselle LM, Reyes G, Gonda MA, Aldovini A, Debouk C, Gallo RC, et al. The trans-activator gene of HTLV-III is essential for virus replication. Nature 1986, 320:367-371.

Frankel AD, Pabo CO. Cellular uptake of the tat protein from human immunodeficiency virus. Cell 1988, 55:1189-1193.

Hinkula J, Svanholm C, Schwartz S, Lundholm P, Brytting M, Engstrom G, Benthin R, Glaser H, Sutter G, Kohleisen B, Erfle V, Okuda K, Wigzell H, Wahren B. Recognition of prominent viral epitopes induced by immunization with human immunodeficiency virus type 1 regulatory genes. J Virol 1997, 71:5528-5539.

James CO, Huang MB, Khan M, Garcia-Barrio M, Powell MD, Bond VC. Extracellular Nef protein targets CD4+ T cells for apoptosis by interacting with CXCR4 surface receptors. J Virol 2004 Mar, 78(6):3099-3109.

Kim DT, Mitchell DJ, Brockstedt DG, Fong L, Nolan GP, Fathman CG, Engleman EG, Rothbard JB. Introduction of soluble proteins into the MHC class I pathway by conjugation to an HIV tat peptide. J Immunol 1997, 159:1666-1668.

Koedel U, Kohleisen B, Sporer B et al. HIV Type 1 Nef protein is a viral Factor for Leukocyte Recruitment into the Central Nervous System. Journal of Immunology 1999, 163:1237-1245.

Krone WJ, Debouck C, Epstein LG, Heutink P, Meloen R, Goudsmit J. Natural antibodies to HIV-tat epitopes and expression of HIV-1 genes in vivo. J Med Virol 1988, 26:261-270.

Merck/HVTN News release. Vaccination and Enrolment Are Discontinued in Phase II Trials of Merck's Investigational HIV Vaccine Candidate-Interim Analysis of STEP Study Shows Vaccine was not Effective, 2007.

Moy P, Daikh Y, Pepinsky B, Thomas D, Fawell S, Barsoum J. Tat-mediated protein delivery can facilitate MHC class I presentation of antigens. Mol Biotechnol 1996, 6:105-113.

Osterhaus AD, van Baalen CA, Gruters RA, Schutten M, Siebelink CH, Hulskotte EG, Tijhaar EJ, Randall RE, van Amerongen G, Fleuchaus A, Erfle V, Sutter G. Vaccination with Rev and Tat against AIDS. Vaccine 1999, 17:2713-2714.

Ratner L, Haseltine W, Patarca R, Livak KJ, Starcich B, Josephs SF, Doran ER, Rafalski JA, Whitehorn EA, Baumeister K, Ivanoff L, Petteway SR Jr, Pearson ML, Lautenberger JA, Papas TS, Ghrayeb J, Chang NT, Gallo RC, Wong-Staal F. Complete nucleotide sequence of the AIDS virus, HTLV-III. Nature 1985, 313:277-284.

Re MC, Furlini G, Vignoli M, Ramazzotti E, Roderigo G, De Rosa V, Zauli G, Lolli S, Capitani S, La Placa M. J Acquired Immune Defic Syndr Hum Retrovirol 1995, 10:408-416.

Re MC, Vignoli M, Furlini G, Gibellini D, Colangeli V, Vitone F, La Placa M. Antibodies against full-lenght Tat protein and some low-molecular-weight Tat-peptides correlate with low or undetectable viral load in HIV-1 seropositive patients. J Clin Virol 2001, 21:81-89.

Reiss P, Lange JM, de Ronde A, de Wolf F, Dekker J, Debouck C, Goudsmit J. Speed of progression to AIDS and degree of antibody response to accessory gene products of HIV-1. J Med Virol 1990, 30:163-168.

Rezza G, Fiorelli V, Dorrucci M, Ciccozzi M, Tripiciano A, Scoglio A, Collacchi B, Ruiz-Alvarez M, Giannetto C, Caputo A, Tomasoni L, Castelli F, Sciandra M, Sinicco A, Ensoli F, Butto S, Ensoli B. The presence of anti-Tat antibodies is predictive of long-term nonprogression to AIDS or severe immunodeficiency: findings in a cohort of HIV-1 seroconverters. J Infect Dis 2005, 15:1321-1324.

Rodman TC, To SE, Hashish H, Manchester K. Epitopes for natural antibodies of human immunodeficiency virus (HIV)-negative (normal) and HIV-positive sera are coincident with two key functional sequences of HIV Tat protein. Proc Natl Acad Sci USA 1993, 90:7719-7723.

Shutt DC, Soll DR. HIV-induced T-cell syncytia release a two component T-helper cell chemoattractant composed of Nef and Tat. J Cell Sci 1999, 112 (Pt 22):3931-3941.

Tyagi M, Rusnati M, Presta M, Giacca M. Internalization of HIV-1 tat requires cell surface heparan sulfate proteoglycans. J Biol Chem 2001, 276:3254-3261.

Wahren B, Ljungberg K, Rollman E, Levi M, Zuber B, Kjerrstrom Zuber A, Hinkula J, Leandersson AC, Calarota S, Hejdeman B, Bratt G, Sandstrom E. HIV subtypes and recombination strains--strategies for induction of immune responses in man. Vaccine 2002, 20(15):1988-1993.

WHO. HIV/AIDS Programme, Antiretroviral therapy for HIV infection in adults and adolescents: recommendations for a public health approach – Revision 2006 – Annex 7.

Wu Y, Marsh JW. Selective transcription and modulation of resting T cell activity by preintegrated HIV DNA. Science 2001, 293:1503-1506.

Zagury D, Lachgar A, Chams V, Fall LS, Bernard J, Zagury JF, Bizzini B, Gringeri A, Santagostino E, Rappaport J, Feldman M, Burny A, Gallo RC. Interferon alpha and Tat involvement in the immuno suppression of uninfected T cells and C-C chemokine decline in AIDS. Proc Natl Acad Sci USA 1998a, 95:3851-3856.

Zagury JF, Sill A, Blattner W, Lachgar A, Le Buanec H, Richardson M, Rappaport J, Hendel H, Bizzini B, Gringeri A, Carcagno M, Criscuolo M, Burny A, Gallo RC,

Zagury D. Antibodies to the HIV-1 Tat protein correlated with nonprogression to AIDS: a rationale for the use of Tat toxoid as an HIV-1 vaccine. J Hum Virol 1998b, 1:282-292.

APPENDIX I

WHO - Division of AIDS - Table for Grading Severity of Adult Adverse Experiences

PARAMETER	GRADE 1<br>MILD	GRADE 2<br>MODERATE	GRADE 3<br>SEVERE	GRADE 4<br>POTENTIALLY LIFE THREATING
**HEMATOLOGY**				
Hemoglobin	8.0 g/dL-9.4 g/dL	7.0 g/dL-7.9 g/dL	6.5 g/dL-6.9 g/dL	<6.5 g/dL
Absolute neutrophil count	1000-1500/mm^3	750-999/mm^3	500-749/mm^3	<500/mm^3
Platelets	75,000-99,000/mm^3	50,000-74,999/mm^3	20,000-49,999/ mm^3	<20,000/mm^3
Prothrombin time (PT)	>1.0-1.25 X ULN	>1.25-1.5 X ULN	>1.5-3.0 X ULN	>3 X ULN
PTT	>1.0-1.66 X ULN	>1.66-2.33 X ULN	>2.33-3.0 X ULN	>3.0 X ULN
Methemoglobin	5.0-10.0%	10.1-15.0%	15.1-20.0%	>20%
**CHEMISTRIES**				
SODIUM				
Hyponatremia	130-135 meq/L	123-129 meq/L	116-122 meq/L	<116 meq/L
Hypernatremia	146-150 meq/L	151-157 meq/L	158-165 meq/L	>165 meq/L
**POTASSIUM**				
Hypokalemia	3.0-3.4 meq/L	2.5-2.9 meq/L	2.0-2.4 meq/L	<2.0 meq/L
Hyperkalemia	5.6-6.0 meq/L	6.1-6.5 meq/L	6.6-7.0 meq/L	>7.0 meq/L
**PHOSPHATE**				
Hypophosphatemia	2.0-2.4 mg/dL	1.5-1.9 mg/dL	1.0-1.4 mg/dL	<1.0 mg/dL
**CALCIUM-(Corrected for albumin)**				
Hypocalcemia	7.8-8.4 mg/dL	7.0-7.7 mg/dL	6.1-6.9 mg/dL	<6.1 mg/dL
Hypercalcemia	10.6-11.5 mg/dL	11.6-12.5 mg/dL	12.6-13.5 mg/dL	>13.5 mg/dL
**MAGNESIUM**				
Hypomagnesemia	1.2-1.4 meq/L	0.9-1.1 meq/L	0.6-0.8 meq/L	<0.6 meq/L
**BILIRUBIN**				
Hyperbilirubinemia	>1.0-1.5 x ULN	>1.5-2.5 x ULN	>2.5-5.0 x ULN	>5 x ULN
**GLUCOSE**				
Hypoglycemia	55-64 mg/dL	40-54 mg/dL	30-39 mg/dL	<30 mg/dL
Hyperglycemia (nonfasting and no prior diabetes)	116-160 mg/dL	161-250 mg/dL	251-500 mg/dL	>500 mg/dL
Triglycerides	________	400-750 mg/dL	751-1200 mg/dL	>1200
Creatinine	>1.0-1.5 x ULN	>1.5-3.0 x ULN	>3.0-6.0 x ULN	>6.0 x ULN
**URIC ACID**				
Hyperuricemia	7.5-10.0 mg/dL	10.1-12.0 mg/dL	12.1-15.0 mg/dL	>15.0 mg/dL
**LIVER TRANSAMINASE (LFTs)**				
AST (SGOT)	1.25-2.5 x ULN	>2.5-5.0 x ULN	>5.0-10.0 x ULN	>10.0 x ULN
ALT (SGPT)	1.25-2.5 x ULN	>2.5-5.0 x ULN	>5.0-10.0 x ULN	>10.0 x ULN
GGT	1.25-2.5 x ULN	>2.5-5.0 x ULN	>5.0-10.0 x ULN	>10.0 x ULN
Alk Phos	1.25-2.5 x ULN	>2.5-5.0 x ULN	>5.0-10.0 x ULN	>10.0 x ULN
**PANCREATIC ENZYMES**				
Amylase	1.0-1.5 x ULN	>1.5-2.0 x ULN	>2.0-5.0 x ULN	>5.0 x ULN
Pancreatic amylase	1.0-1.5 x ULN	>1.5-2.0 x ULN	>2.0-5.0 x ULN	>5.0 x ULN
Lipasi	1.0-1.5 x ULN	>1.5-2.0 x ULN	>2.0-5.0 x ULN	>5.0 x ULN
**CARDIOVASCULAR**				
Cardiac Arrythmia	________	Asymptomatic; transient dysrhythmia, no Rx req	Recurrent/persistent dysrhythmia; symptomatic Rx req	Unstable dysrhythmia, hospitalisation and Rx req
Hypotension	Transient orthostatic hypotension, no Rx	Symptoms correctable with oral fluid Rx	IV fluid req, no hospitalisation req	Hospitalisation req
Hypertension	Transient, increase >20 mm/Hg; no Rx	Recurrent; chronic increase >20 mm/Hg	Acute Rx req; outpatient hospitalisation possible	Hospitalization req
Pericarditis	Minimal effusion	Mild/mod	Symptomatic	Tamponade or

		asymptomatic effusion, no Rx	effusion, pain, EKG changes	periocardiocentesis or surgery req
Hemorrhage, blood loss	-----------------------	Mildly symptomatic, no Rx required	Gross blood loss OR 1-2 units transfused	Massive blood loss OR >2 units transfused
**GASTROINTESTINAL**				
Nausea	Mild or transient; reasonable intake maintained	Mod discomfort OR intake decreased for <3 days	Severe discomfort OR minimal intake for ≥3 days	Hospitalisation req
Vomiting	Mild or transient; 2-3 episodes per day OR mild vomiting lasting <1 week	Mod OR persistent; 4-5 episodes per day OR vomiting lasting ≥1 week	Severe vomiting of all food/fluids in 24 hrs OR orthostatic hypotension IV Rx req	Hypotensive shock OR hospitalisation req for IV Rx req
Diarrhea	Mild or transient; 3-4 loose stools per day OR mild diarrhea lasting <1 week	Mod OR persistent; 5-7 loose stools per day OR diarrhea lasting ≥1 week	Bloody diarrhea OR orthostatic hypotension OR >7 loose stools/day OR IV Rx required	Hypotensive shock OR hospitalisation req
Oral discomfort/dysphagia	Mild discomfort, no difficulty swallowing	Difficulty swallowing but able to eat and drink	Unable to swallow solids	Unable to drink fluids; IV fluids req
Constipation	Mild	Moderate	Severe	Distention with vomiting
**RESPIRATORY**				
Cough (foraerosol studies)	Transient; no Rx	Treatment associated cough; inhaled bronchodilator	Uncontrolled cough; systemic Rx req	--------------------------------
Broncospasm Acute	Transient; no Rx; FEV1 70%-<80% (or peak flow)	Rx req; normalizes with bronchodilator; FEV1 <50%-70% (or peak flow)	No normalization with bronchodilator; FEV1 <25%-50% (or peak flow), retractions	Cyanosis; FEV1 25% (or peak flow) or intubated
Dyspnea	Dyspnea onexertion	Dyspnea with normal activity	Dyspnea at rest	Dyspnea requiring O_2 therapy
**NEUROLOGIC**				
Neuro-cerebellar	Slight incoordination OR dysdiadochokinesia	Intention tremor OR dysmetria OR slurredspeech OR nystagmus	Ataxia requiring assistance to walk or arm incoordination interfering with ADLs	Unable to stand
Neuro-psych/mood	-----------------------	--------------------------	Severe mood changes requiring medical intervention	Acute psychosis req hospitalization
Paresthesia	Mild discomfort; no Rx req	Mod discomfort; non-narcotic analgesia req	Severe discomfort; OR narcotic analgesia req with improvement	Incapaciting; OR not responsive narcotic analgesia
Neuro-motor	Mild weakness in muscle or feet but able to walk and/or mild increase or decrease in reflexes	Mod weakness in feet (unable to walk on heels and/or toes), mild weakness in hands, still able to do most tasks and/or loss of previously present reflex or development of hyperrflexia and/or unable to deep knee bends due to weakness	Marked distal weakness (unable to dorsiflex toes or foot drop), and proximal weakness e.g., in hands, with ADLs and/or requiring assistance to walk and/or unable to rise from chair unassisted	Confined to bed orwheel chair because of muscle weakness
Neuro-sensory	Mild impairment (dec sensation, e.g.,	Mod impairment (mod dec sensation e.g.,	Severe impairment (dec or loss of)	Sensory loss involves limbs and trunk sensation

	vibratory, pinprick, hot/cold in great toes) in focal area or symmetrical distribution)	vibratory, pinprick, hot/cold to anklesand/or joint position or mild impairment that is not symmetrical		to knees or wrists) or loss of sensation of at least mod degree in multiple different body areas (i.e., upper and lower extremities)
**URINALYSIS**				
Proteinuria				
Spot urine	1+	2-3+	4+	Nephrotic syndrome
24 hour urine	200 mg-1 g loss/day OR <0.3% OR <3 g/l	>1-2 g loss/day OR 0.3-1.0% OR 3-10 g/l	>2-3.5 g loss/day OR >1.0% OR 10 g/l	Nephrotic syndrome OR >3.5 g loss/day
Gross Hematuria	Microscopic only	Gross, no clots	Gross plus clots	Obstructive OR transfusion req
**MISCELLANEOUS**				
Fever	37.7-38.5C OR 100.0-101.5F	38.6-39.5C OR 101.6-102.9F	39.6-40.5C OR 103-105F	>40.5C OR >105F
Oral > 12 hours				
Headache	Mild, No Rx req	Mod; or non-narcoticanalgesia Rx	Severe; OR responds initial narcotic Rx	Intractable; OR requiring repeated narcotic Rx
Allergic Reaction	Pruritus without rash	Localized urticaria	Generalized urticaria angioedema	Anaphylaxis
Cutaneous/Rash/Dermatitis	Erythema, pruritus	Diffuse maculopapular rash OR dry desquamation	Vesiculation OR moist desquamation OR ulceration	ANY ONE: mucous membrane involvment, suspected Stevens-Johnson (TEN), erythema multiforma, necrosis req surgery, exfoliative dermatitis
Local Reaction (2° parenteral Rx-not vaccination or skin test)	Erythema	Induration <10 mm OR inflammation OR phlebitis	Induration > 10 mm OR ulceration	Necrosis of skin
Fatigue	Normal activity reduced <25%	Normal activity reduced <25-50%	Normal activity reduced <50% cannot work	Unable to care for self

APPENDIX II

FLOW-CHART

WEEK	Screening		0	4	8	12	16	20	24	48
**DAY**	**-21**	**-14/ -7**	**0**	**28**	**56**	**84**	**112**	**140**	**168**	**336**
STUDY VISIT	*01*		*02*	*03*	*04*	*05*	*06*	*07*	*08*	*09*
**Immunization Arm A**			**X**	**X**	**X**	**X**	**X**			
**Immunization Arm B**			**X**	**X**	**X**					
Signed Informed Consent	X									
Medical History – Previous Medications	X									
**CLINICAL AND SAFETY EVALUATIONS**										
Physical Examination – Vital Signs	X		X	X	X	X	X	X	X	X
HIV1/2 ELISA	X									
Hematology	X		X	X	X	X	X	X	X	X
Coagulation Assessment	X		X	X	X	X	X	X	X	X
Blood Chemistry	X		X	X	X	X	X	X	X	X
Urine Dipstick	X		X	X	X	X	X	X	X	X
$CD4^{+}$ T Cell counts	X	X	X	X	X	X	X	X	X	X
Pregnancy Test (females)	X		X	X	X	X^{A}	X^{A}			
12-lead ECG	X						X^{B}		X^{A}	
PPD Test / X-Ray	X									
Serum IgE levels	X									
Syphilis Serology Test	X									
Psychological assessment and tests	X	X	X		X		X			X
Counseling on pregnancy avoidance	X		X	X	X	X^{A}	X^{A}			
Assessment of Adverse Events			X	X	X	X	X	X	X	X
Concomitant Medications	X		X	X	X	X	X	X	X	X
**IMMUNOLOGICAL AND VIROLOGICAL EVALUATIONS**										
Anti-Tat antibodies (IgG, IgM, IgA)	X		X	X	X	X	X	X	X	X
Neutralization of Tat activity			X			X*			X*	
Lymphoproliferative response to Tat			X		X	X		X		X
γIFN, IL-4, IL-2 production in response to Tat			X		X	X		X		X
HIV-1 Plasma Viremia	X		X	X	X	X	X	X	X	X
PBMC, plasma and sera freezing	X			X			X		X	
Whole blood shipment	X		X	X	X	X	X	X	X	X

Blood sample collection for all tests will be performed just before the immunization.

X^{A} = to be done only for Arm A.
X^{B} = to be done only for Arm B.
* The test will be performed at Study Visit 2 (Day 0). The time-line of the following determinations could be rescheduled in relationship to the trend of the humoral immune response to Tat.

APPENDIX III

Facsimile of drug labels

ISTITUTO SUPERIORE DI SANITA'/CNAIDS
Protocollo: ISS T-002
Flacone da 0,5 ml di Tat (7,5 µg)
Da somministrarsi per via intradermica
Data di scadenza 1009 Lotto n.: T2051007
Kit n.:____________
Centro n.:___________ Paziente n.:_/_/_/__
Visita n.:____________________

ISS/CNAIDS
Protocollo: ISS T-002
Fl. da 0,5 ml di Tat (7,5 µg)
Da somministrarsi per via intradermica
Data scadenza 1009
Lotto n. : T2051007
Kit n.: __________
Centro n.: __________
Paziente n.: __________

Primary Container (Single Vial)

Promotore della sperimentazione:
ISTITUTO SUPERIORE
DI SANITA'/CNAIDS
Protocollo N: ISS T-002
Contiene: 1 flacone da 0,5 ml di Tat (7,5 µg)
Soluzione iniettabile da somministrarsi per via intradermica (ID) secondo le procedure descritte nel protocollo di studio
Data di scadenza:1009
Lotto n.: T2051007
Kit n.: ________
Centro n.:______________
Paziente n.:__/__/__/__
Visita n.:__________
Nome sperimentatore:_________

Conservare a -80°C protetto dalla luce
DA USARSI SOLO PER
SPERIMENTAZIONE CLINICA

Secondary Container (Single Vial Box)

Promotore della sperimentazione:
ISTITUTO SUPERIORE DI SANITA'/CNAIDS
Protocollo No: ISS T-002
Contiene: 5 flaconi da 0,5 ml di Tat (7,5 µg)
Soluzione iniettabile da somministrarsi per via Intradermica (ID) secondo le procedure descritte nel protocollo di studio
Data scadenza 1009 Lotto n.: T2051007
Kit n.:________
Centro n.:_____________________
Paziente n.: __/__/__/__
Nome Sperimentatore:____________________
Conservare a – 80°C protetto dalla luce
DA USARSI SOLO PER SPERIMENTAZIONE CLINICA

Tertiary Container (Patient Kit)

Promotore della sperimentazione: ISTITUTO SUPERIORE DI SANITA'/CNAIDS
Viale Regina Elena, 299 00161 Roma
Tel. 06 49903209
Protocollo No: ISS T-002
Contiene: 4 scatole di N° 3/5 flaconi da 0,5 ml di Tat (7,5/30 µg)
Soluzione iniettabile da somministrarsi per via intradermica (ID) secondo le procedure descrittte nel protocollo di studio
DA USARSI SOLO PER SPERIMENTAZIONE CLINICA
Contiene i kit n.:______________________
Lotto n.: T2051007 / T3051007 Data di scadenza: 1009
Nome Sperimentatore: ______________________ Centro n.__________
Conservare a temperatura -80° C protetto dalla luce

Quaternary Container (Shipment Box)

APPENDIX IV

Declaration of Helsinki

Policy
WORLD MEDICAL ASSOCIATION DECLARATION OF HELSINKI
Ethical Principles for Medical Research Involving Human Subjects
Adopted by the 18th WMA General Assembly, Helsinki, Finland, June 1964, and amended by the
29th WMA General Assembly, Tokyo, Japan, October 1975
35th WMA General Assembly, Venice, Italy, October 1983
41st WMA General Assembly, Hong Kong, September 1989
48th WMA General Assembly, Somerset West, Republic of South Africa, October 1996
and the 52nd WMA General Assembly, Edinburgh, Scotland, October 2000
Note of Clarification on Paragraph 29 added by the WMA General Assembly, Washington 2002
Note of Clarification on Paragraph 30 added by the WMA General Assembly, Tokyo 2004

A. INTRODUCTION

1. The World Medical Association has developed the Declaration of Helsinki as a statement of ethical principles to provide guidance to physicians and other participants in medical research involving human subjects. Medical research involving human subjects includes research on identifiable human material or identifiable data.

2. It is the duty of the physician to promote and safeguard the health of the people. The physician's knowledge and conscience are dedicated to the fulfillment of this duty.

3. The Declaration of Geneva of the World Medical Association binds the physician with the words, "The health of my patient will be my first consideration," and the International Code of Medical Ethics declares that, "A physician shall act only in the patient's interest when providing medical care which might have the effect of weakening the physical and mental condition of the patient."

4. Medical progress is based on research which ultimately must rest in part on experimentation involving human subjects.

5. In medical research on human subjects, considerations related to the well-being of the human subject should take precedence over the interests of science and society.

6. The primary purpose of medical research involving human subjects is to improve prophylactic, diagnostic and therapeutic procedures and the understanding of the aetiology and pathogenesis of disease. Even the best proven prophylactic, diagnostic, and therapeutic methods must continuously be challenged through research for their effectiveness, efficiency, accessibility and quality.

7. In current medical practice and in medical research, most prophylactic, diagnostic and therapeutic procedures involve risks and burdens.

8. Medical research is subject to ethical standards that promote respect for all human beings and protect their health and rights. Some research populations are vulnerable and need special protection. The particular needs of the economically and medically disadvantaged must be recognized. Special attention is also required for those who cannot give or refuse consent for themselves, for those who may be subject to giving consent under duress, for those who will not benefit personally from the research and for those for whom the research is combined with care.

9. Research Investigators should be aware of the ethical, legal and regulatory requirements for research on human subjects in their own countries as well as applicable international requirements. No national ethical, legal or regulatory requirement should be allowed to reduce or eliminate any of the protections for human subjects set forth in this Declaration.

B. BASIC PRINCIPLES FOR ALL MEDICAL RESEARCH

10. It is the duty of the physician in medical research to protect the life, health, privacy, and dignity of the human subject.

11. Medical research involving human subjects must conform to generally accepted scientific principles, be based on a thorough knowledge of the scientific literature, other relevant sources of information, and on adequate laboratory and, where appropriate, animal experimentation.

12. Appropriate caution must be exercised in the conduct of research which may affect the environment, and the welfare of animals used for research must be respected.

13. The design and performance of each experimental procedure involving human subjects should be clearly formulated in an experimental protocol. This protocol should be submitted for consideration, comment, guidance, and where appropriate, approval to a specially appointed ethical review committee, which must be independent of the investigator, the sponsor or any other kind of undue influence. This independent committee should be in conformity with the laws and regulations of the country in which the research experiment is performed. The committee has the right to monitor ongoing trials. The researcher has the obligation to provide monitoring information to the committee, especially any serious adverse events. The researcher should also submit to the committee, for review, information regarding funding, sponsors, institutional affiliations, other potential conflicts of interest and incentives for subjects.

14. The research protocol should always contain a statement of the ethical considerations involved and should indicate that there is compliance with the principles enunciated in this Declaration.

15. Medical research involving human subjects should be conducted only by scientifically qualified persons and under the supervision of a clinically competent medical person. The responsibility for the human subject must always rest with a medically qualified person and never rest on the subject of the research, even though the subject has given consent.

16. Every medical research project involving human subjects should be preceded by careful assessment of predictable risks and burdens in comparison with foreseeable benefits to the subject or to others. This does not preclude the participation of healthy volunteers in medical research. The design of all studies should be publicly available.

17. Physicians should abstain from engaging in research projects involving human subjects unless they are confident that the risks involved have been adequately assessed and can be satisfactorily managed. Physicians should cease any investigation if the risks are found to outweigh the potential benefits or if there is conclusive proof of positive and beneficial results.

18. Medical research involving human subjects should only be conducted if the importance of the objective outweighs the inherent risks and burdens to the subject. This is especially important when the human subjects are healthy volunteers.

19. Medical research is only justified if there is a reasonable likelihood that the populations in which the research is carried out stand to benefit from the results of the research.

20. The subjects must be volunteers and informed participants in the research project.

21. The right of research subjects to safeguard their integrity must always be respected. Every precaution should be taken to respect the privacy of the subject, the confidentiality of the patient's information and to minimize the impact of the study on the subject's physical and mental integrity and on the personality of the subject.

22. In any research on human beings, each potential subject must be adequately informed of the aims, methods, sources of funding, any possible conflicts of interest, institutional affiliations of the researcher, the anticipated benefits and potential risks of the study and the discomfort it may entail. The subject should be informed of the right to abstain from participation in the study or to withdraw consent to participate at any time without reprisal. After ensuring that the subject has understood the information, the physician should then obtain the subject's freely-given informed consent, preferably in writing. If the consent cannot be obtained in writing, the non-written consent must be formally documented and witnessed.

23. When obtaining informed consent for the research project the physician should be particularly cautious if the subject is in a dependent relationship with the physician or may consent under duress. In that case the informed consent should be obtained by a well-informed physician who is not engaged in the investigation and who is completely independent of this relationship.

24. For a research subject who is legally incompetent, physically or mentally incapable of giving consent or is a legally incompetent minor, the investigator must obtain informed consent from the legally authorized representative in accordance with applicable law. These groups should not be included in research unless the research is necessary to promote the health of the population represented and this research cannot instead be performed on legally competent persons.

25. When a subject deemed legally incompetent, such as a minor child, is able to give assent to decisions about participation in research, the investigator must obtain that assent in addition to the consent of the legally authorized representative.

26. Research on individuals from whom it is not possible to obtain consent, including proxy or advance consent, should be done only if the physical/mental condition that prevents obtaining informed consent is a necessary characteristic of the research population. The specific reasons for involving research subjects with a condition that renders them unable to give informed consent should be stated in the experimental protocol for consideration and approval of the review committee. The protocol should state that consent to remain in the research should be obtained as soon as possible from the individual or a legally authorized surrogate.

27. Both authors and publishers have ethical obligations. In publication of the results of research, the investigators are obliged to preserve the accuracy of the results. Negative as well as positive results should be published or otherwise publicly available. Sources of funding, institutional affiliations and any possible conflicts of interest should be declared in the publication. Reports of experimentation not in accordance with the principles laid down in this Declaration should not be accepted for publication.

C. ADDITIONAL PRINCIPLES FOR MEDICAL RESEARCH COMBINED WITH MEDICAL CARE

28. The physician may combine medical research with medical care, only to the extent that the research is justified by its potential prophylactic, diagnostic or therapeutic value. When medical research is combined with medical care, additional standards apply to protect the patients who are research subjects.

29. The benefits, risks, burdens and effectiveness of a new method should be tested against those of the best current prophylactic, diagnostic, and therapeutic methods. This does not exclude the use of placebo, or no treatment, in studies where no proven prophylactic, diagnostic or therapeutic method exists.[1]

30. At the conclusion of the study, every patient entered into the study should be assured of access to the best proven prophylactic, diagnostic and therapeutic methods identified by the study.[2]

31. The physician should fully inform the patient which aspects of the care are related to the research. The refusal of a patient to participate in a study must never interfere with the patient-physician relationship.

32. In the treatment of a patient, where proven prophylactic, diagnostic and therapeutic methods do not exist or have been ineffective, the physician, with informed consent from the patient, must be free to use unproven or new prophylactic, diagnostic and therapeutic measures, if in the physician's judgement it offers hope of saving life, re-establishing health or alleviating suffering. Where possible, these measures should be made the object of research, designed to evaluate their safety and efficacy. In all cases, new information should be recorded and, where appropriate, published. The other relevant guidelines of this Declaration should be followed.

---

1 **Note of clarification on paragraph 29 of the WMA Declaration of Helsinki**
The WMA hereby reaffirms its position that extreme care must be taken in making use of a placebo-controlled trial and that in general this methodology should only be used in the absence of existing proven therapy. However, a placebo-controlled trial may be ethically acceptable, even if proven therapy is available, under the following circumstances:

- Where for compelling and scientifically sound methodological reasons its use is necessary to determine the efficacy or safety of a prophylactic, diagnostic or therapeutic method; or

- Where a prophylactic, diagnostic or therapeutic method is being investigated for a minor condition and the patients who receive placebo will not be subject to any additional risk of serious or irreversible harm.

All other provisions of the Declaration of Helsinki must be adhered to, especially the need for appropriate ethical and scientific review.

2 **Note of clarification on paragraph 30 of the WMA Declaration of Helsinki**
The WMA hereby reaffirms its position that it is necessary during the study planning process to identify post-trial access by study participants to prophylactic, diagnostic and therapeutic procedures identified as beneficial in the study or access to other appropriate care. Post-trial access arrangements or other care must be described in the study protocol so the ethical review committee may consider such arrangements during its review.
9.10.2004

APPENDIX V

Psychological Evaluation

Sponsor:
AIDS National Center
Istituto Superiore di Sanità (ISS)
Viale Regina Elena, 299
00161 – Rome, Italy

Psychological evaluation for subjects participating in the trial:

ISS T-002 - "A phase II randomized, open label, immunogenicity and safety trial of the vaccine based on the recombinant biologically active HIV-1 Tat protein in anti-Tat negative HIV-1 infected HAART-treated adult subjects"

1. PROJECT IDENTIFICATION 877
2. BACKGROUND 87
   2.1 Introduction 877
   2.2 Phase-I-related background 888
3. RATIONALE 899
4. HYPOTHESIS AND TARGET 899
   4.1 Key Targets 899
   4.2 Secondary Targets 899
5. METHODS 90
   5.1 MINI International Neuropsychiatric Interview -Plus (M.I.N.I. –Plus) 90
   5.2 SCL-90-R (Symptom Checklist-90-Revised) 90
   5.3 State-Trait Anxiety Inventory (STAI) 911
   5.4 Beck Depression Inventory (BDI) 922
   5.5 International Personality Item Pool Representation of the NEO PI-R (IPIP-Neo) 922
   5.6 World Health Organization Quality of Life, Short Form (WHOQoL-SF) 933
   5.7 Temperament and Character Inventory - Revised (TCI-R) 944
   5.8 Anamnestic Standard Form Administration and Clinical Interview 955
6. PROCEDURES 955
7. STATISTICAL METHODOLOGY AND SAMPLE SIZE 966
8. REFERENCES 977
9. LIST OF ABREVIATIONS AND SYMBOLS 999

1. PROJECT IDENTIFICATION

TITLE: Psychological evaluation for subjects participating in the trial ISS T-002 "A phase II randomized, open label, immunogenicity and safety trial of the vaccine based on the recombinant biologically active HIV-1 Tat protein in anti-Tat negative HIV-1 infected HAART-treated adult subjects".

AUTHORS: Visintini R., Cattaneo E., Carretta I., Balestra S.

VERSION: 12th February 2008

2. BACKGROUND

2.1 Introduction

Data collected by the WHO and Joint United Nation Programme on HIV/AIDS (UNAIDS) show that in 2007 33,2 millions of people have been living with HIV and that in the same year over two thirds (68%) of the new infections occurred in sub-Saharan Africa.

New anti-retroviral therapies resulted very successful in controlling the disease; their application is however rather impossible, due to the high cost of drugs, to the need for well-equipped labs, as well as for clinical monitoring.

The production of a vaccine against HIV is the latest outcome of existing prevention strategies, and actually stands for the real unique possibility to control virus diffusion in population (Excler J.L., 2005).

HIV-vaccine trial literature does not include any specific protocol dealing with volunteer subjects's psychology. Only few data are available to date, mainly focusing on subjects' motivation to participate in the trial, on the subsequent difficulty to recruit volunteers as well as on drop-out risk in follow up. Recently, USA and other countries started trials on HIV-vaccines: some of them are going towards phase II and III, whereas others stopped at phase I. One of the main reasons for such a failure seems to to be the difficult recruitment of new volunteer subjects (Jenkins R.A., Temoshok L.R., Virochsiri K., 1995).

In previous vaccine trial experiences many potential candidates subscribed to participate, although their active participation to phase-I studies on a specific vaccine did not result as satisfactory as expected (Jenkins R.A., Temoshok L.R., Virochsiri K., 1995).

Loss of subjects during follow up is also critical. "Time" is identified as major factor of loss: subjects seem to lose their motivation along the trial inspite of their original will to commit and be part of the vaccine trial (Koblin B.A., Holte S., Lenderking B., Heagerty P., 2000; Halpern S.D., Metzger D., Berlin J., Ubel I.A., 2001).

Drop-out phenomena seem to depend on the volunteers' perception of being menaced and risking to lose their own safety, as well as of feeling "used as a guinea-pig" (Jenkins R.A., Temoshok L.R., Virochsiri K., 1995).

2.2 Phase-I-related background

The introduction of psycho-social monitoring derives from the need to limit the above mentioned problems, tailor the-trial on such subjects' needs, explore motivation and feelings associated to trial participation, in order to prevent drop-out and risk-behaviours.

Experiences made in Phase-I-trials with the Tat vaccine have showed the importance of such an evaluation even from an ethical point of view, since the psychological support may help in elaborating conscious and unconscious fantasies on the vaccine and allows volunteers to assess adequately their own expectations.

Previous experiences evidenced that subjects approaching such trials have a good social and economical background, a superior education and do not show any pathological personality traits (in press). With reference to general populations' psychological variables, they can be included in a "normal" range. For such reasons volunteers' recruitment can be carried out through several channels, such as biological parameters investigation, TVA (Telefono Verde AIDS [AIDS toll free telephone number) counselling as well as direct clinical evaluation.

Since the volunteers' sample created for this T-002 study can be considered as an "average" range, reference can be made to the literature stressing on the relationship between HIV infection stage and volunteers' anxiety/depression. Such data are probably influenced by AIDS physical symptoms: high scores in MMPI-2 scales have actually been registered in HIV-positive patients with neuropsychological and physical symptoms (Drebing et al., 1994; Inman et al., 2002; Kalichman et al., 1995).

The inclusion of a major number of subjects in Phase II trial and the need to widen the biological inclusion criteria set for Phase I, will probably result in a different composition of the volunteers' sample. Methods[1] used in Phase I, as oulined in the psychosocial protocol, were mainly oriented to psycho-diagnostic analysis. Together with quality life monitoring and motivation, already included in Phase I, this experience outlined the opportunity of widening volunteers' psychological evaluation.

[1] MMPI-2 (Minnesota Multiphasic Personality Inventory, 1996); Questionnaire based on the Information-Motivation-Behavioral Skills Model (IMB, Fisher & Fisher, 1999); Quality of Life Questionnaire created by World Health Organization (WHOQOL Group, 2001, 2002); Self Rating Anxiety Scale (Zung, 1971); Subjective Cognitive Symptoms Questionnaire (Maj, 1991).

3. RATIONALE

The introduction of an articulated psychological evaluation within an anti-HIV-vaccine trial is very innovative, since only few studies included a volunteers' psychological investigation. The experience made during ISS Phase I studies actually outlines the need for a psychological evaluation that goes much further than the mere psychopathological aspects and, by identifying volunteer's common features, could thus contribute to define adequate guidelines.

This Phase II trial will therefore pay special attention to the assessment of particular aspects limiting volunteers' inclusion (Mood Disorders, Suicidal Ideation, Psychotics Disorders, substance abuse) as well as to the variables able to influence trial involvement, such as motivation, frustration tolerability, interpersonal style and life quality.

4. HYPOTHESIS AND TARGET

4.1 Key Targets

This psychological evaluation has the following key-targets:

1. Psycho-diagnostic screening (following DSM-IV criteria) of all subjects, to exclude those presenting psychopathological symptoms, as defined by exclusion criteria, and to widen volunteers' tutelage.
2. Volunteers' psychological evaluation, to rule out subjects whose psychologicy might influence protocol adherence.
3. Psychopathological symptoms identification during the trial, to prevent trial interruption.
4. Psychological monitoring to identify specific personality profiles and problematic areas which need psychological support in order to prevent drop outs or risk behaviors.
5. Life quality assessment and monitoring during the trial, to verify the subject's mental and physical state, the environmental support and his way to behave in interpersonal relationships.

4.2 Secondary Targets

This psychological evaluation has the following secondary-targets:

1. Quantify trial experience impact on subjects' psychic status.
2. Obtain useful information for the development of further psychological guidelines in clinical trials.
3. Investigate on subjects' motivation, for a more accurate rule-in phase in future vaccine trials.

5. METHODS

Self-administered questionnaires will be offered to each subject. The administration schedule, shown below (fig. 1 – Flow Chart), is not to interfere with vaccine trial procedures.

Foreign subjects, or with a different cultural background, who might not fit to the standard psychological assessment, shall undergo clinical interviews only.

5.1 MINI International Neuropsychiatric Interview -Plus (M.I.N.I. –Plus)

The M.I.N.I. is a short (15-30 minutes) structured diagnostic interview complying with DSM-III-R/IV (Diagnostic and Statistic Manual – III version Revised) and ICD-10 criteria. It was designed for clinical practice and research in psychiatric and primary care settings. A short training (1-3 hours) will teach clinicians on how to learn it. The M.I.N.I. -Plus is a fully structured instrument able to detect DSM-IV mood, anxiety, somatoform, substance use, psychotic, eating, conduct and adjustment disorders. For each disorder the M.I.N.I. lists an ordered series of 6-12 questions, with immediate scoring. Different time frames - current, past, or lifetime – are proposed, according to the disorder type. Psychometric examination shows acceptable test-retest and inter-rater reliability (Sheehan et al., 1998). The M.I.N.I.-Plus has been preferred to other screening tools due to its easy administration, the relatively short training for use, its broad coverage and its reported quick administration time.

This test will provide for psycho diagnostic screening (as for the DSM-IV criteria), aiming at assessing psychopathological symptoms as identified in the exclusion criteria (Key-Target nr. 1). Moreover, it will also identify new psychopathological symptoms which might develop during the trial and could then lead to trial interruption (Key-Target nr. 3).

M.I.N.I. -Plus has already been used HIV-positive patients studies to identify Axis I disorders (Olley B.O., Gxamza F., et al., 2003; Olley B.O., Seedat S., Nei D.G., Stein D.J., 2004).

5.2 SCL-90-R (Symptom Checklist-90-Revised)

The SCL-90-R is a brief, multidimensional self-report inventory developed in the 1980s by Derogatis and designed to screen a broad range of psychological problems and psychopathology symptoms. The SCL-90-R is also a useful progress or outcome measurement tool, adopted by clinical psychologists and psychiatrists in mental health, medical and educational settings as well as for research purposes. It can be useful in both the initial evaluation of patients and for measuring patient progress during treatment.

Each item is rated on a five-point distress scale (0-4), ranging from "not at all" to "extremely." Questions should be answered in terms of symptoms or feelings "over the last week, including today."

The nine primary symptom dimensions are labeled as: SOM – Somatisation; O-C – Obsessive-Compulsive; I-S – Interpersonal Sensitivity; DEP – Depression; ANX – Anxiety; HOS – Hostility; PHOB – Phobic Anxiety; PAR – Paranoid Ideation; PSY – Psychoticism.

Global Indices: GSI – Global Severity Index; PSDI – Positive Symptom Distress Index; PST – Positive Symptom Total.

High test-retest and internal consistency have been demonstrated, and no problem seem to be related to practice effects.

Administration time is of 12-15 minutes (90 items). A degree in psychology is not a prerequisite for any of such applications.

5.3 State-Trait Anxiety Inventory (STAI)

STAI measures adults' anxiety. A clear distinction is made between the temporary condition of "state-anxiety" and the more general and long-standing quality of "trait-anxiety". Being very simple, this inventory is ideal for the evaluation of individuals with lower educational backgrounds.

It consists of two sub-scales STAI T-Anxiety Scale (X-2) and STAI S-Anxiety Scale (X-1) aiming at evaluating "trait-anxiety" and "state-anxiety"; they both propose a series of twenty questions, each one offering a range of four possible responses. Full completion takes about ten minutes.

Based on a ten-year-experience X Form underwent a substantial revision, that changed 30% of its items. It improved psychometric characteristics (now called Y) and provides now a clearer distinction between the two anxiety types (Spielberger, 1983).

State anxiety is considered as a specific experience, a lack of confidence and a feeling of, helplessness before a perceived damage, leading to worry or to flight and avoidance. Trait anxiety leads to consider stressful experience as harmful and dangerous and consequently to react with intensity.

Available in more than forty languages, the STAI stands for the leading measure of personal anxiety worldwide. Italian translation and validation by Predabissi L. and Santinello M. (1989).

This inventory will be used in the psychological monitoring stage, to identify problematic areas which might need psychological support in order to prevent drop-outs and risk behaviors (Key-Target nr. 4).

5.4 Beck Depression Inventory (BDI)

The Beck Depression Inventory (BDI, BDI-II), created by Dr. Aaron T. Beck, is a twenty-one question multiple choice self-report inventory. It is one of the most widely used instruments for measuring the severity of depression. The most updated version is designed for 13-year-old individuals and includes items relating to depression symptoms such as hopelessness and irritability, cognitions such as guilt or feelings of being punished, as well as physical symptoms such as fatigue, weight loss, and lack of interest.

The original BDI, first published in 1961, consisted of twenty-one questions about how the subject has been feeling in the last week.

Items involving changes in body image, hypochondria, and difficulty working were replaced. Each question has a set of at least four possible answer choices, ranging in intensity.

BDI asks participants to rate how they have been feeling for the past week.

Each answer is being scored on a scale value from 0 to 3 and the total score is compared to a key to determine the depression's severity. The standard cut-offs are as follows: 0–10 indicates that a person is not depressed, 11–17 indicates mild-moderate depression, 18–23 indicates moderate-severe depression and 24–63 indicates severe depression. Higher total scores indicate more severe depressive symptoms.

BDI shows high internal consistency, with alpha coefficients of .86 and .81 for psychiatric and non-psychiatric populations, respectively. The BDI is positively correlated with the Hamilton Depression Rating Scale (Pearson r = 0.73) and the Zung Self Reported Depression Scale (r = .76), showing good agreement.

5.5 International Personality Item Pool Representation of the NEO PI-R (IPIP-Neo)

The IPIP-NEO Personality Inventory is a personality test based on the Five-Factor Theory of Personality. It proposes five different domains, which include Extraversion, Agreeableness, Conscientiousness, Neuroticism, and Openness to Experience. Each domain is further divided to include thirty sub-domains. For example, the Extraversion domain is divided into six sub-domains: friendliness, gregariousness, assertiveness, activity level, excitement-seeking, and cheerfulness. IPIP-NEO is based on factor analysis.

The Big Five are a descriptive model of personality, and their main features can be summarized as follows:

- Openness to Experience - appreciation for art, emotion, adventure, unusual ideas, imagination, and curiosity.

- Conscientiousness - a tendency to show self-discipline, act dutifully and aim for achievement; planned rather than spontaneous behaviour.
- Extraversion - energy, positive emotions, surgency and the tendency to seek stimulation and the company of others.
- Agreeableness - a tendency to be compassionate and cooperative rather than suspicious and antagonistic towards others.
- Neuroticism - a tendency to experience unpleasant emotions easily, such as anger, anxiety, depression, or vulnerability; sometimes called emotional instability.

The short version of the IPIP-NEO inventory includes 120 items from the original inventory; completion normally takes 15-25 minutes.

This inventory will provide volunteers' psychological assessment able to rule out subjects presenting adherence-influencing psychological functioning (Key-Target nr. 2).

5.6 World Health Organization Quality of Life, Short Form (WHOQoL-SF)

The questionnaire described hereafter will be used during trial phases for the subject' Life Quality evaluation and monitoring. Observation will focus on the subject's perception of his physical and mental health, his level of social support and his way to interact in personal relationships.

The World Health Organization Quality of Life- Short form (The WHOQoL Group, 1998) is a questionnaire proposed by World Health Organization with the purpose of evaluating the subject perception of his life quality. It's a self-administered questionnaire consisting of 26 items, divided in four categories: physical health, psychological health, social relationships, environment.

The WHOQoL short form, composed by a limited number of items is taken from a 100 items version, the WHOQoL-100. An item selection procedure has been carried out, with the goal of preserving the questionnaire structural integrity.

Each Category is composed by subdimensions:

- Physical Health: Pain and Bodily discomfort, Energy and Tiredness, Sleep and Rest.
- Psychological Health: Positive emotions, Reasoning skills, Learning skills, Memory skills, Concentration, Self-esteem, Bodily image and Appearance. Negative emotions.
- Social Relationships: Personal relationships, Social support, Sexual activities.
- Environment: Safety, Domestic environment, Economic resources, Social and Health insurance, Chances to learn new abilities, Social participation and recreational occasions, Natural environment, Transportations.

5.7 Temperament and Character Inventory - Revised (TCI-R)

This tool will be used for volunteers' psychological evaluation, with the purpose to rule out the trial the subjects whose psychological functions might interfere with protocol adherence needs, as well as for volunteers' defense.

Temperament and Character Inventory (TCI, Cloninger C.R., Przybeck T.R, e Svrakic D.M., 1994) is a self-administered questionnaire consisting of 240 items measured on a true-false dichotomous scale.

The questionnaire leads to an evaluation of temperament and character individual differences, referring to Cloninger personality theory.

Cloninger defines normal personality development as an adaptive complex system with seven factors: four temperamental and three character ones.

The term *Temperament* refers to behavioural dispositions present up to birth. Cloninger identified three temperamental dimensions, genetically independent:

- *Novelty seeking:* behavioural activation, with high stimulation levels;
- *Harm avoidance:* worry for possible consequences of one' own actions, it represents the opposite of impulsivity;
- *Reward dependence:* worry for other people's reaction to one'actions and behaviour maintenance in response to a positive reinforce.

Character refers to the individual differences as for targets and values. Its development is influenced by rules, cultural expectations as well as personal experience; it occurs in a step-by-step development from childhood to adulthood, refers to long-term target identification and uses logics.

Cloninger defined three dimensions:

- *Self Directedness:* people's ability to set goals and act accordingly in a disciplined way;
- *Cooperativeness:* ability to be open to other's needs and to share his own work;
- *Self-Transcendence:* ability to tolerate conceptual idiosyncrasies and be spiritual.

With respect to the possible combination of temperamental and character dimensions, TCI allows for different clusters influencing individuals to qualitatively distinct emotional and behavioural patterns. For example, different temperamental assets are associated to different behavioural risks, as violence or charity or extreme love for others.

5.8 Anamnestic Standard Form Administration and Clinical Interview

It's a brief form summarizing the main information collected during clinical interviews.

In order to standardize all the clinical interviews in the different clinical sites, some important areas have been highlighted:

- Anamnestic Information
- HIV course
- Motivations to Trial
- Personal History (Sexuality, Relationships)
- Social Support
- Expectations to Trial
- HIV Impact on QoL
- Coping Strategies.

Both during pre-screening and clinical trial:

- in case of diagnostic doubt, the psychologist shall be authorized to require more clinical interviews with volunteers.
- in the presence of psychopathological volunteers, the psychologist will be always authorized to ask for psychiatric or psychotherapeutic intervention, including the possibility to address the volunteer to a specialized clinical structure.

6. PROCEDURES

Once the Informed Consent is obtained in writing, volunteers will begin the pre-screening phase and evaluations.

In addition to the present psychological evaluation, the psychologist/psychiatrist at the clinical site will support volunteers throughout critical points during the study (screening failure, enrolment, treatment phase, conclusion of the study, adverse events and follow-up).

Figure 1: Flow Chart

**WEEK**	**Screening**		**0**	**4**	**8**	**12**	**16**	**20**	**24**	**48**
**DAY**	**-21**	**-14 /- 7**	**0**	**28**	**56**	**84**	**112**	**140**	**168**	**336**
STUDY VISIT	*01*		*02*	*03*	*04*	*05*	*06*	07	*08*	*09*
Immunization Arm A			X	X	X	X	X			
Immunization Arm B			X	X	X					
Signed Informed Consent	X									
Medical History	X									
**Psychological Assessments**										
MINI - Plus	X									
SCL – 90 - R	X									
STAI (y - x)		X	X		X		X			X
BDI		X	X		X		X			X
IPIP - Neo - R	X									
TCI - R	X									
WHOQoL - sf		X	X		X		X			X
Clinical Interview		X								
Anamnestic Schedule		X								

7. STATISTICAL METHODOLOGY AND SAMPLE SIZE

Descriptive statistics (mean scores, frequencies) will be used. χ^2 and Independent Sample *t*-Test statistics will be performed to compare different subgroups. Based on the division between sub-groups as Factors, Multivariate Analyses of Variance (MANOVA) will be applied on the different groups of dependent variables.

Finally, based on the total scores resulting from all administered questionnaires as dependent variables, Repeated Measures Analyses of Variance (RM ANOVA) will be applied to the subjects who will complete the trial.

REFERENCES

- Beck A.T., Ward C., Mendelson M. (1961) *"Beck Depression Inventory (BDI)"*. Arch Gen Psychiatry, 4: 561-571.
- Beck A.T., Steer R.A., & Brown G.K. (1996) *"Manual for the Beck Depression Inventory-II"*. San Antonio, TX: Psychological Corporation.
- Cloninger, C. R., Przybeck, T. R., Svrakic, D. M., Wetzel, R. D. (1994). The Temperament and Character Inventory (TCI): a guide to its development and use. St. Louis, Missouri: Center for Psychobiology of Personality.
- Derogatis L.R., Melisataros N., *The Brief Symptom Inventory: an introductory report*, Psychol Med, 1983 Aug;13(3): 595-605.
- Drebing C.E., Van Gorp W.G., Hinkin C., Miller E.N., Satz P., Kim D.S., Holston S., D'Elia L.F., *Confounding factors in the measurement of depression in HIV*, J Pers Assess, Feb,62(1): 68-83,1994.
- Excler J.L., *AIDS vaccine development: Perspectives, challenges & hopes*, Indian Journal of Medical Research, Apr. 2005.
- Halpern S.D., Metzger D., Berlin J., Ubel P.A., *Who will enroll? Predicting partecipation in a phase II AIDS vaccine trial*, JAIDS, 27: 281-8, 2001.
- Inman T.H., Esther J.K., Robertson W.T., Hall C.D., Robertson K.R., *The Minnesota Multiphasic Personality Inventory-2 across the human immunodeficiency virus spectrum*, Assessment, Mar,9(1): 24-30, 2002.
- Jenkins R.A., Temoshok L.R., Virochsiri K., *Incentives and Disincentives to participate in prophylactic HIV vaccine research*, J of Acquired Immune deficiency Syndrome and Human Retrovirology, 9: 36-42, 1995.
- Kalichman S.C., Sikkema K.J, Somlai A., *Assessing persons with human immunodeficiency virus (HIV) infection using the Beck Depression Inventory: disease processes and other potential confounds*, J Pers Assess, Feb, 64(1): 86-100, 1995.
- Koblin B.A., Holte S., Lenderking B., Heagerty P., *Readiness for HIV vaccine efficacy trials: concerns elicited from gay men at increased risk for HIV infection,* JAIDS, 10: 61-4, 2000.
- Olley B.O., Gxamza F., et al., *Psychopathology and coping in recently diagnosed HIV/AIDS patients—the role of gender*, S Afr Med J, 2003 Dec; 93(12): 928-31.
- Olley B.O., Seedat S., Nei D.G., Stein D.J., *Predictors of major depression in recently diagnosed patients with HIV/AIDS in South Africa*, AIDS Patients Care STDS, 2004 Aug; 18 (8): 481-7.

- Predabissi L. and Santinello M., *Verification of the validity of the STAI, form Y, by Spielberger,* Bollettino di psicologia applicata, No 191-192, Jul-Dec 1989, pp. 11-14.
- Sheehan B.V., Lecrubier Y., Sheehan K.H., et al., *The Mini International Neuropsychiatric Interview (MINI): The development and validation of structured diagnostic psychiatric interview for DSM-IV and ICD-10*, J Clin Psychiatry, 59(Suppl 20): 22-33, 1998.
- Spielberger C.D., *Manual for the State-Trait Anxiety Inventory. Revised Edition,* Consulting, Psychologists Press, Palo Alto, CA, 1983.
- Spielberger C. D., *S.T.A.I. Inventario per l'Ansia di Stato e di Tratto, Forma Y.* Firenze, Organizzazioni Speciali, 1991.
- UNAIDS-WHO. AIDS epidemic update, December 2007. *http://data.unaids.org/pub/EPISlides/2007/2007_epiupdate_en.pdf,* downloaded on January, 8th 2008.
- The WHOQoL Group, *Development of the World Health Organisation WHOQOL-BREF Quality of Life Assessment,* Psychological Medicine, 28, 551-558 (1998b).

9. LIST OF ABREVIATIONS AND SYMBOLS

AIDS	Acquired ImmunoDeficiency Syndrome
BDI	Beck Depression Inventory
DSM-III-R	Diagnostic and Statistic Manual of Mental Disorders (Third Version Revised)
DSM-IV	Diagnostic and Statistic Manual of Mental Disorders (Fourth Version)
HAART	Highly Active Antiretroviral Therapy
HIV	Human Immunodeficiency Virus
HIV-1 Tat	Transactivator (regulatory gene which accelerates the production of more HIV virus)
ICD-10	International Statistical Classification of Diseases and Related Health Problems
IPIP-Neo	International Personality Item Pool Representation of the NEO PI-R
ISS	Istituto Superiore di Sanità
MANOVA	Multivariate Analysis of Variance
M.I.N.I.–Plus	MINI International Neuropsychiatric Interview-Plus
MMPI-2	Minnesota Multiphasic Personality Inventory (Second Version)
Pearson r	Pearson product-moment correlation coefficient
QoL	Quality of Life
RM ANOVA	Repeated Measures Analysis of Variance
SCL-90-R	Symptom Checklist-90-Revised
STAI	State-Trait Anxiety Inventory
t-Test	is any of a number of tests based on the t distribution
TCI-R	Temperament and Character Inventory - Revised
TVA	Telefono Verde AIDS
UNAIDS	Joint United Nation Programme on HIV/AIDS
WHO	World Health Organization
WHOQoL-SF	World Health Organizations Quality of Life- Short Form
$\chi 2$	test based on the $\chi 2$ distribution

APPENDIX VI

List of Abbreviations and Symbols

Abs	Absorbance
ADCC	Antibody-mediated Cellular Cytotoxicity
ADR	Adverse Drug Reaction
AE	Adverse Event
Ag	Antigen
AIDS	Acquired Immune Deficiency Syndrome
ALT	Alanine amino Transferase
ANOVA	Analysis of Variance
APCs	Antigen-Presenting Cells
APTT	Activated Partial Thromboplastin Time
AST	Aspartate amino Transferase
AVIP	AIDS Vaccine Integrated Programme
BAER	Brainstem Auditory Evoked Response
CAB	Community Advisory Board
CCR-5	Chemokine Receptor 5
CD	Cluster of Differentiation
CDC	Center of Disease Control
CEC	Central Ethical Committee
CFSE	CarboxyFluoroscein Succinimidyl Ester
CL-CRF	Core Laboratory Case Report Form
cm	Centimeter
CNS	Central Nervous System
CRF	Case Report Form
CRO	Contract Research Organization
CSF	CerebroSpinal Fluid
CTLs	Cytotoxic T Lymphocytes
CTM	Clinical Trial Monitor
DEAE	Diethylaminoethyl
dl	Deciliter
DNA	Deoxyribonucleic Acid
DSMB	Data Safety Monitoring Board
ECG	Electrocardiogram
e-CRF	Electronic Case Report Form
ELISA	Enzyme-Linked Immunosorbent Assay
EudraCT	European Clinical Trials Database
FP6	Framework Programme 6
g	Gram
GCP	Good Clinical Practice
GGT (γGT)	Gamma Glutamyl Transferase
GMP	Good Manufacturing Practice
GMT	Geometric Mean Titers
HAART	Highly Active Antiretroviral Therapy
Hb	Hemoglobin
Hbe Ag	Hepatitis B e Antigen
Hbs Ag	Hepatitis B surface Antigen
HBV	Hepatitis B Virus

HCT	Hematocrit
HCV	Hepatitis C Virus
HHV-8	Human Herpes Virus 8
HIV-1	Human Immunodeficiency Virus type 1
HIV-1 LTR	Human Immunodeficiency Virus type 1 Long Terminal Repeat
HIV-2	Human Immunodeficiency Virus type 2
HLA	Human Leukocyte Antigen
HPLC	High Performance Liquid Chromatography
HTLV-I	Human T-Lymphotropic Virus I
HTLV-II	Human T-Lymphotropic Virus II
HTLV-III	Human T-Lymphotropic Virus III
HSA	Human Serum Albumin
ICAV	Italian Concerted Action on HIV/AIDS Vaccine Development
ICH	International Conference on Harmonization
ICS	Intracellular Cytokine Staining
ID	Identification number
id	Intradermally
IFO	Istituti Fisioterapici Ospitalieri
IgA	Immunoglobulin A
IgE	Immunoglobulin E
IgG	Immunoglobulin G
IgM	Immunoglobulin M
IL-2	Interleukin-2
IL-4	Interleukin-4
IL-12	Interleukin-12
INMI	Istituto Nazionale Malattie Infettive
ISS	Istituto Superiore di Sanità
Kg	Kilogram
LEC	Local Ethical Committee
MCV	Mean Corpuscolar Volume
MDDCs	Monocyte-derived dendritic cells
MedDRA	Medical Dictionary for Regulatory Activities
mg	Milligram
MHC	Major Histocompatibility Complex
MID_{50}	Monkey 50% Infectious Doses
min	Minute
MIP-1α	Macrophage Inflammatory Protein 1α
MIP-1β	Macrophage Inflammatory Protein-1β
ml	Milliliter
mm^3	Millimeter cube
NA	Neutralizing Antibodies
ng	Nanogram
NIH	National Institutes of Health
NK	Natural Killer
nm	Nanometer
NMR	Nuclear Magnetic Resonance
PBMC	Peripheral Blood Mononuclear Cell
PPD	Purified Protein Derivative
RANTES	Regulated on Activation Normal T cell Expressed and Secreted
RBC	Red blood cell
RD	Relationship to Drug

RNA	Ribonucleic Acid
RPR	Rapid Plasma Reagin
SADR	Serious Adverse Drug Reaction
SAE	Serious Adverse Event
SAS	Statistical and data Analysis Software
sc	Subcutaneous
SHIV	Simian/Human Immunodeficiency Virus
SIV	Simian Immunodeficiency Virus
SOC	System Organ Classes
SOP	Standard Operating Procedure
SUSAR	Suspected Unexpected Serious Adverse Reaction
Th1	Type 1 helper T Cells
Th2	Type 2 helper T Cells
TM	Trade Mark
TNF-α	Tumor Necrosis Factor-α
UI	International Unit
WBC	White Blood Cell
WHO	World Health Organization
WMA	World Medical Association
γIFN	γ Interferon
μg	Microgram
μl	Microliter
°C	Temperature in degrees Celsius
