# Supplementary material for: Therapeutic Immunization with HIV-1 Tat Reduces Immune Activation and Loss of Regulatory T-Cells and Improves Immune Function in Subjects on HAART
Source: PLoS One. 2010 Nov 11;5(11):e13540. doi: 10.1371/journal.pone.0013540 (PMC2978690; doi:10.1371/journal.pone.0013540)
Supplement: Protocol S2 — ISS OBS T-002 Protocol. (0.15 MB PDF) [file pone.0013540.s018.pdf]

OBSERVATIONAL STUDY WITH ADDITIONAL DIAGNOSTIC PROCEDURES ON ANTI-TAT IMMUNE RESPONSE IN HIV-1-INFECTED HAART-TREATED ADULT SUBJECTS

Protocol Number: ISS OBS T-002

Study sponsored by Istituto Superiore di Sanità

Data collection and analysis National AIDS Center
Istituto Superiore di Sanità
V.le Regina Elena, 299
00161 Rome, Italy

LIST OF CLINICAL SITES AND PRINCIPAL INVESTIGATORS:

Ospedale A. di Savoia
Prof. G. Di Perri
C.so Svizzera, 164
10149 Torino

Ospedale S. Raffaele
Prof. A.Lazzarin/G. Tambussi
Via Stamira d'Ancona, 20
20127 Milano

Ospedale Sacco
Prof. M. Galli
Via G.B. Grassi, 74
20157 Milano

Policlinico di Modena
Prof. R. Esposito
L.go del Pozzo, 71
41100 Modena

Spedali Civili
Prof. G. Carosi
Piazzale Spedali Civili, 1
25123 Brescia

Ospedali Riuniti
Prof. F.Suter
Via Tito Livio, 2
24123 Bergamo

Arcispedale S. Anna
Prof. F. Ghinelli
Corso Giovecca, 203
44100 Ferrara

Ospedale S. Maria Annunziata
Prof. F. Mazzotta
Via dell'Antella 58
50011 Antella, Firenze

IFO-San Gallicano
Prof. G. Palamara
Via di S. Gallicano, 25/a
00153 Roma

INMI "L. Spallanzani"
Prof. P. Narciso, Prof. A. Antinori
Via Portuense, 292
00149 Roma

Ospedale S.M. Goretti
Prof. F. Soscia
Via Guido Reni, 1
04100 Latina

Policlinico di Bari
Prof. G. Pastore
Piazza Giulio Cesare, 11
70124 Bari

OBSERVATIONAL STUDY WITH ADDITIONAL DIAGNOSTIC PROCEDURES ON ANTI-TAT IMMUNE RESPONSE IN HIV-1-INFECTED HAART-TREATED ADULT SUBJECTS

Protocol Number: ISS OBS T-002
Date: June 14, 2007

PROTOCOL SUMMARY

Protocol : A prospective observational study on the clinical, immunological and virological outcome of HAART-receiving HIV-1 infected adult subjects. The primary endpoint of this study is to evaluate the frequency, magnitude, quality and persistence of the anti-Tat humoral and cellular immune response in HIV-1 infected individuals under successful HAART. The secondary endpoint is to prospectively evaluate the immunological, virological and clinical outcome of anti-Tat positive versus anti-Tat negative subjects under successful HAART in order to define novel prognostic markers for the clinical management of HIV-1 infected HAART-treated subjects. In addition, the study will promote the creation of a network of clinical centers working according to standard operating procedures for the harmonization of the conduction of future vaccine trials.

Subjects: HIV-1 infected adult subjects of either gender, ≥ 18 years old, under successful HAART (chronic suppression of HIV infection with a plasma viremia <50 copies/ml in the last 6 months and without a history of virologic rebound) and a known nadir level of $CD4^+$ T cells.

Number of subjects: Enrollment will be open to all HIV-1 infected adult subjects under HAART regimens at the participating clinical centers.

Time Period: 5 years with possibility of extension.

Sponsoring Agency: Istituto Superiore di Sanità (ISS)
Viale Regina Elena, 299
00161 Rome, Italy

1. PURPOSE OF THE STUDY

The present study is designed as a prospective observational study directed to evaluate the frequency, magnitude, quality and persistence (primary endpoint) of the anti-Tat immune response in highly active antiretroviral therapy (HAART)-receiving HIV-1 infected individuals, and to prospectively evaluate the immunological, virological and clinical outcome of anti-Tat positive versus anti-Tat negative subjects under successful HAART (secondary endpoint) in order to determine the impact of anti-Tat immunity on HIV disease progression as well as the potential use of anti-Tat immune response assessment for the clinical and therapeutic management of HAART-treated infected patients. This survey, that will evaluate a large number of subjects, will provide important information for the design, planning and conduction of future therapeutic vaccine trials based on the HIV-1 Tat protein in HAART-treated patients.

2. BACKGROUND AND RATIONALE

The HIV-1 Tat is a very early regulatory protein that plays a major role in HIV-1 replication and AIDS pathogenesis. Tat is produced early after infection, even prior to HIV integration, being necessary for viral gene expression (Arya, Science 1985; Fisher, Nature 1986; Ensoli, J Virol. 1993; Wu, Science 2001), cell-to-cell virus transmission and disease progression. Tat therefore represents a key HIV-1 target protein for the host immune response and an optimal candidate for the development of a vaccine against HIV/AIDS (Ensoli, Nature 1990, J. Virol. 1993, Nature 1994; Chang, AIDS 1997, Ensoli, AIDS 2006). Several studies suggest that in natural HIV infection an immune response to Tat (humoral and cellular) exerts a protective role upon disease progression. In particular, the presence of anti-Tat antibodies strongly correlates with a slower progression to the disease (Reiss, 1990; Rodman, 1993; Re, 1995; Zagury, 1998; Re, 2001; Rezza 2005). However, anti-Tat antibodies are produced by only a small fraction (around 20%) of asymptomatic HIV infected individuals while, in contrast, the majority (50% to 70 %) of these individuals generates an adaptive γ-IFN T-cell response against Tat, as well as a wide spectrum of antibodies against virtually all other virus components, including other regulatory gene products (Rev, Nef). This indicates the existence of a strong Th1 T cell response against Tat in asymptomatic HIV infection, as compared to the limited (frequency, titre and repertoire) anti-Tat Th2 B-cell response. In addition, most anti-Tat antibodies recognize only conformational Tat epitopes, and in most cases fail to recognize linear epitopes (Ensoli, AIDS 2006). Overall, the anti-Tat humoral response appears to be clustered in a fraction of asymptomatic HIV-infected patients (Stage A HIV disease) with peak frequencies in Long Term Non Progressor (LTNP) individuals (around 30 % to 40 %), and is lost in more advanced stages of the disease (Krone 1988, Demirhan 2000, Re 2001) as well as in fast progressors to AIDS (Zagury 1998). Results of a cross-sectional and longitudinal study recently performed in a cohort of 252 individuals with accurately estimated dates of seroconversion and a medium follow-up of 7.2 years, revealed a strong association between the presence of anti-Tat antibodies and a slower progression to overt disease (Rezza, 2005). In fact, the risk of developing AIDS or severe immunodeficiency was 60% lower for anti-Tat positive individuals than for anti-Tat negative individuals. The longitudinal analysis also indicated that none of the individuals who were persistently anti-Tat positive progressed to AIDS, whereas 53 events (AIDS or severe immunodeficiency) occurred among those who were persistently anti-Tat negative (Rezza, 2005). Thus, a strong correlation exists between the presence of anti-Tat antibodies and non-progression to AIDS. This suggests that the presence of anti-Tat antibodies may predict the clinical outcome and that the induction of an immune response against Tat either during the course of the natural infection or by vaccination, may contrast the progression to the disease. Thus, the anti-Tat immune

response represents an immune correlate of protection which may have an important prognostic value and direct implications for the clinical and therapeutic management of infected patients. This notion also suggests that therapeutic vaccination with a Tat-based vaccine should be targeted only at those individuals that do not develop a humoral immune response to Tat in the natural course of the infection. On this basis, the knowledge of the impact (and the potential for a reciprocal influence) that the implementation of different HAART regimens may exert upon the preservation or the generation of Ag-specific immune responses might be of critical importance in the clinical and therapeutic management of infected subjects as well as for therapeutic vaccine development. Although HAART usually induces a potent suppression of viral replication with a net improvement of immunologic parameters (i.e. sustained increases in $CD4^+$ T cell number), very little is known about the characteristics of the adaptive immune response against Tat as well as other viral antigens during HAART. In addition, it is unknown whether the different class of antiretroviral drugs may play a part in influencing the quality of the immune reconstitution. Indeed, HIV protease inhibitors (HIV-PI) have been shown to interfere with the maturation and function of dendritic cells and cytotoxic T lymphocytes and to alter the apoptotic pathway of T cells and hematopoietic cell precursors (André, 1998; Schmidtke, 1999; Gruber, 2001; Kobayashi, 1999; Sloand, 1999 & 2000; Weichold, 1999; Lu & Andrieu 2000, Phenix, 2001). On the other hand, nucleoside reverse-transcriptase inhibitors (NRTI) and non-nucleoside reverse-transcriptase inhibitors (NNRTI) have also been shown to interfere with the growth of hematopoietic cell precursors, T cells and peripheral blood mononuclear cells (Somadossi, 1987; Chitnis, 2002; Viora, 1995 & 1997; Pilon, 2002; Hashimoto, AIDS Res Hum Retrov 1997; Mangiacasale, 2003). Altogether, these data suggest that, by directly or indirectly interfering with cells of the immune system, all the different combination regimens of antiretroviral drugs may concur in altering the immune response in infected patients as well as potentially in vaccinees.
It is, therefore, of utmost importance to collect data on the frequency, magnitude, quality and persistence of the anti-Tat immune response in different cohorts of HIV-1 infected individuals under different HAART regimens. These data will be essential to prospectively evaluate the immunological, virological and clinical outcome of anti-Tat positive versus anti-Tat negative patients which are treated by different antiretroviral intervention, and to define the potential application of this novel immunologic analysis in the clinical staging and therapeutic management of infected patients. In addition, by creating a network of clinical centers working according to standard operating procedures (SOPs), identifying potential vaccinee's cohorts and simplifying all the pre-screening procedures for enrollment, this study will be key for the design and conduction of future therapeutic Phase II vaccine trials based on the HIV-1 Tat protein in HAART-treated subjects. In particular, the results of the Phase I Safety and Immunogenicity Trials of Recombinant HIV-1 Tat (ISS P-001 and ISS T-001) demonstrated that the vaccine based on the recombinant Tat protein is well tolerated and immunogenic in both seronegative (Preventative trial) and seropositive (Therapeutic trial) individuals. Based on these data, phase II therapeutic trials will be targeted on individuals with no detectable humoral immune response against Tat and will be conducted in parallel by two distinct multicentric studies, which are going to start soon in Italy and will address two different clinical categories consisting of patients undergoing HAART regimens and asymptomatic drug-naïve individuals respectively.

3. STUDY DESIGN AND OBJECTIVES

The goal of this observational prospective study is to collect information on the frequency, magnitude, quality and persistence of anti-Tat immunity in HIV-1 infected subjects under successful HAART, to evaluate the immunological, virological and clinical outcome of anti-Tat positive versus the anti-Tat negative HAART-treated subjects, and to define whether the assessment

of anti-Tat immunity in HAART-treated patients may prove useful for the clinical and therapeutic management of infected patients.

Since these evaluations require assessments that go beyond the routine clinical monitoring, this protocol represents an observational study with additional diagnostic procedures.

3.1 Primary endpoint

The primary endpoint of the study is to evaluate the frequency, magnitude, quality and persistence of the anti-Tat humoral and cellular immune response in HAART-receiving individuals with chronically suppressed HIV-1 infection. Patients will be stratified according to PI-based or NNRTI-based HAART regimens.

3.2 Secondary endpoint

The secondary endpoint of the study is to prospectively evaluate the immunological, virological and clinical outcome of anti-Tat positive versus anti-Tat negative subjects under successful HIV-PI- or NNRTI-HAART in order to define immunologic markers of potential prognostic value for the clinical and therapeutic management of the disease.

The aim of this study is also to create a network of clinical centers working according SOPs for the conduction of future vaccine trials and for the management of HIV-1 infected HAART-treated subjects.

3.3 Clinical and immunological determinations

The presence of specific humoral and cellular immune responses will be monitored by determining anti-Tat antibodies in the sera of subjects, and by assessing the proliferative response (CFSE) and the production of γIFN, IL-4 and IL-2 (Elispot) by peripheral blood mononuclear cells (PBMC) in response to Tat. Refer to section 6 for details of the laboratory assays to be performed.

The decline of $CD4^+$ T cell counts, the increase of HIV plasma viral load or the occurrence of AIDS-defining events will be assessed to determine disease progression.

3.4 Study population

Enrollment will be open to all HIV-1 infected adult subjects of either gender, under any HAART regimen with a known nadir level of $CD4^+$ T cells and chronically suppressed HIV infection (plasma viremia <50 copies/ml in the last 6 months and without a history of virologic rebound).

No sample size limitation is defined and the number of the subjects enrolled will depend on the accrual capability of participating clinical centers.

3.5 Selection criteria

Informed consent will be obtained prior to assessments of eligibility. Subsequently, investigators will proceed with medical history, physical examination, and blood collection for routinary and extra-routinary tests.

3.5.1 Inclusion criteria

All the following criteria have to be met for the patients to be eligible for the study:

- Diagnosis of HIV-1 infection
- To be under successful HAART treatment with plasma viremia <50 copies/ml in the last 6 months prior to initiation of the study, without a history of virologic rebound
- Known $CD4^+$ T cells nadir
- Age ≥ 18 years old
- Signed informed consent

Subjects with concomitant viral infections will be included in the study.

3.5.2 Exclusion criteria

Any of the following criteria will exclude the subjects from the study:

- Current therapy with immunomodulators or immunosuppressive drugs, or chemotherapy for neoplastic disorders
- Concomitant treatment for HBV or HCV infection

3.6 Enrollment and study duration

Enrollment will start at study initiation. In order to collect sufficient information on the anti-Tat immune response and on the immunological, virological and clinical outcome of the disease, the follow up of the enrolled subjects will be continued for up to 5 years with the possibility to extend the survey. No enrollment period is planned.

Enrolled subjects may elect to discontinue their participation at any time.

3.7 Lost to follow-up

If a participant misses a scheduled study visit, the study staff will try to establish communication with the participant through all possible means (e.g., writing and telephoning the participants and his/her contacts). The need to attend all scheduled follow-up visits will be emphasized at each visit. If a participant misses a scheduled visit the study staff will try to reschedule the visit within 4 weeks.

4.0 CLINICAL SITES

A network of 12 clinical sites in Italy will participate to this study. The list of all the participating clinical centers is reported below.

**City**	***Clinical Site***
Torino	*Ospedale A. di Savoia*
Milano	*Ospedale S. Raffaele*
Milano	*Ospedale Sacco*
Modena	*Policlinico di Modena*
Brescia	*Spedali Civili*

Bergamo	*Ospedali Riuniti*
Ferrara	*Arcispedale S. Anna*
Firenze	*Ospedale S. Maria Annunziata*
Roma	*IFO-San Gallicano*
Roma	*INMI "L. Spallanzani"*
Latina	*Ospedale S.M. Goretti*
Bari	*Policlinico di Bari*

5.0 CORE LABORATORY

A Core Laboratory of Immunology and Virology will centralize all the immunologic and virological assessments that go beyond the routine clinical monitoring of the patients applying SOPs developed within the AIDS Vaccine Integrated Programme (AVIP) founded within the FP6 program of the European Community and implemented also in the context of the Italian Concerted Action on HIV/AIDS Vaccine Development (ICAV), founded within the Italian AIDS National Program (Ministry of Health).

**City**	***Laboratory Site***
Rome	*Ospedale S.Gallicano IFO*

6.0 STUDY ASSESSMENTS

The following sections provide a detailed listing of the clinical, immunological and virological determinations to be performed in this protocol.

The visits, during which the clinical and laboratory parameters and biological samples necessary to conduct the study are collected, will adhere to the timing of routine clinical monitoring, therefore, visits are scheduled every 3 ± 1 months (see study schedule at section 6.5 for details).

Most of the immunological assessment included in the study is not performed at routine evaluation of the patients, however the amount of blood to be collected for the specific purposes of this study will be reduced to a minimum volume and will be performed, at least in part, on the same samples collected for routine patient monitoring, as detailed in section 6.3.
The first line of testing includes the minimal panel of assays required to identify the presence of an immune response to Tat, which correspond to the primary endpoint of the study; the second line of testing will allow a more detailed exploration of the immune response against Tat and will be performed according to specimens (PBMC, sera, plasma) availability. Following the same principle of "minimal necessary testing", the blood samples (other than those collected for routine clinical monitoring) required in this study will be scheduled every three months only during the first year of conduction of the study, and will be performed every six months thereafter.
SOPs for all these assays have been developed and validated within the FP6-AVIP European Community funded AIDS vaccine program and the Italian Concerted Action on HIV/AIDS Vaccine Development (ICAV), and were employed to determine the immunogenicity induced by the Tat-vaccine in phase I preventative (P-001) and therapeutic (T-001) clinical trials (Ensoli, 2006).

The aim of this study is also to contribute to the ongoing process of standardization, qualification and harmonization of the procedures already developed for future vaccine trials.

6.1 Immunological evaluation

Immunological evaluations will be performed by following a "first line" and a "second line" laboratory testing by the Core Laboratory.

First line immunologic testing

Assessment of anti-Tat humoral immune response:
- determination of IgM, IgG and IgA anti-Tat antibodies in sera
- titration of IgM, IgG and IgA anti-Tat antibodies

Assessment of anti-Tat cellular immune response:
- lymphoproliferative response to Tat (CFSE staining)
- *in vitro* γIFN, IL-4 and IL-2 production in response to Tat (Elispot)

Second line immunologic testing

These studies will be performed, according to specimens (PBMC, sera, plasma) availability:

Characterization of lymphocyte subsets (CD3, CD4, CD8, CD16, CD56, CD19)
Anti-Tat IgG (IgG1, IgG2, IgG3, IgG4) subclasses
Epitope mapping of IgM and IgG anti-Tat antibodies
Anti-HIV regulatory and structural proteins antibodies
Antibody-mediated cellular cytotoxicity (ADCC)
Neutralization of Tat activity by rescue assay
Neutralization of primary isolates (all clades)
Anti-CCR5 antibodies
Anti-CD4 antibodies
Lymphoproliferative response to mitogens and recall antigens
Lymphoproliferative response to HIV-1 Env (CFSE staining)
In vitro γIFN, IL-4 and IL-2 production in response to Tat (ICS)
In vitro γIFN, IL-4 and IL-2 production in response to Env (ICS/Elispot)
B cells phenotype (naïve and memory)
Phenotype and functional characterization of regulatory T cells
Intracellular PBMC staining for granzyme, perforin, cytokines and chemokines
Analysis of Th1 and Th2 cytokines in sera and in PBMC supernatants
Analysis of chemokines in sera and in PBMC supernatants
Lymphocytes spontaneous cell death
B cell cloning
Functional and molecular characterization of clono-specific antibodies
Serum/plasma determination of soluble CD4
HLA typing

6.2 Virological evaluation

Virological evaluations will be performed by the Core Laboratory as for routine monitoring by determining:

HIV-1 plasma viremia (viral RNA copies)

Additional (second line) virologic studies will be performed, according to specimens (PBMC, sera, plasma) availability and will include:

HIV-1 sequencing and virus phylogenetic analysis
Genotypic resistance
Viral tropism
Anti-HTLV-I antibodies
Anti-HTLV-II antibodies
Anti-HBV antibodies
HBV antigens (HbsAg, HbeAg)
Anti-HCV antibodies
HHV-8 antibodies and plasma viremia
HIV-1 Proviral DNA copies

6.3 Sample collection, storage and shipment

The volume of blood to be taken at each visit will be about 40 mL in addition to the volume needed for routine testing (which will require 7 to 10 mL of blood, according to the panel of routinary assays performed in the different clinical centers).
Each sample will be stored with the following information: protocol number, center number, subject number, visit number, sample collection date. PBMC, serum and plasma samples which are unused after performing the tests indicated in sections 6.1 and 6.2 (first line testing) will be utilized for the additional tests (second line) indicated in the same sections or frozen for later analysis.
The shipment of blood samples from the Clinical Centers to Core Laboratory will be organized and performed directly by the Sponsor through a dedicated courier. Samples shipment and storage costs as well as samples examination costs for extra-routine analysis to be performed at the Core Laboratory will be covered by the Sponsor.
A specific manual with a complete description of procedures to be followed for samples collection and management will be provided to investigational sites before study initiation.

6.4 Physical and clinical evaluation

Physical examination will monitor the clinical status and record the appearance of clinical signs and symptoms of disease progression as well as AIDS-defining events. Clinical evaluation will also include the collection of hematological and blood chemistry (including liver and kidney functional parameters) data as performed for routine monitoring at each participating clinical center.

6.5 Visit schedule

The following section provide a listing of the determinations and examinations to be performed in this protocol yearly at the designated time points.
The visits and clinical evaluations, which will follow the timing of routine clinical monitoring, are scheduled every 3 ± 1 months.
The immunological and virological assessments will be performed according to the same schedule during the first year of observation in order to fully characterize the patients. However, in the absence of signs of disease progression, the additional evaluations will be performed every 6 months during the second year of follow-up and thereafter.

VISITS SCHEDULE

(Visits for the third**,** fourth and fifth year of the study will be scheduled as for the second year)

	**First Year**					**Second Year ***			
**MONTH (±1)**	**0**	**3**	**6**	**9**	**12**	**15**	**18**	**21**	**24**
**STUDY VISIT**	**01**	**02**	**03**	**04**	**05**	**06**	**07**	**08**	**09**
Signed Informed Consent	x								
Medical History	x								
Anti-Tat antibodies (IgG, IgM, IgA)	x	x	x	x	x	x	x	x	x
**Clinical Evaluations**									
Physical examination	x	x	x	x	x	x	x	x	x
Hematology	x	x	x	x	x	x	x	x	x
Blood chemistry	x	x	x	x	x	x	x	x	x
Concomitant medications	x	x	x	x	x	x	x	x	x
**Immunological-Virological Evaluations**									
Lymphocyte phenotype	x	x	x	x	x	x	x	x	x
HLA typing	x								
CD4+ T cell count	x	x	x	x	x	x	x	x	x
HIV-1 plasma viremia	x	x	x	x	x	x	x	x	x
Lymphoproliferative response	x	x	x	x	x		x		x
γ-IFN, IL-4, IL-2 production	x	x	x	x	x		x		x
PBMC, plasma and sera freezing	x	x	x	x	x		x		x

* The serological assessment after the first year of the study will be performed on sera frozen obtained at each routine visit (about every 3 months), without additional blood withdrawals.

6.6 Statistical considerations

Sample size

For this observational study no sample size will be predefined due to the fact that the study has the objective of providing information about the frequency, magnitude, quality and persistence of the anti-Tat immune response and of the immunological, virological and clinical outcome in HAART-receiving HIV-1 infected individuals.

Primary variables

- Anti-Tat humoral immune response in terms of IgM, IgG, IgA anti-Tat antibodies
- Epitope mapping of IgM and IgG anti-Tat antibodies
- Neutralization of Tat activity by rescue assay
- Anti-Tat cellular-mediated immune response, in terms of lymphoproliferative response to Tat (CFSE staining), and in vitro γIFN, IL-4, IL-2 production in response to Tat (Elispot, Intracellular staining)

Secondary variables

- Change of $CD4^+$ T cells and HIV viral load

- Trend over time of lymphocytes populations
- Time to AIDS defining clinical condition (according to the CDC criteria)

Other immunological and virological evaluations will be performed and analyzed during the study period.

Statistical analysis

Anti-Tat humoral-cellular immune response will be evaluated by the percentage of anti-Tat positive subjects with the relative 95% confidence interval; geometric mean antibody titers (GMT) will be also performed.
The immune response will be compared between subjects groups stratified by NNRTI-based or PI-based HAART regimens, using a Logistic regression model (odds ratio estimates).
$CD4^{+}$ T cells and HIV viral load will be evaluated and compared between anti-Tat positive and negative subjects using an Analysis of variance model (ANOVA).
The time to AIDS will be determined and compared between groups by the Log-Rank test.
A Multivariate analysis will be applied to the immunological and virological parameters in order to carry out potential prognostic factors for the clinical outcome.

Statistical analysis and data processing will be performed using SAS® software for Windows.
All descriptive statistics used to summarize numeric data will include mean values, standard deviation, median, minimum and maximum. Frequency distributions will be presented for categorical variables.
95% confidence intervals will be determined for all data.
All statistical tests will be performed at two-sided with a 5% significance level.

7.0 DATA COLLECTION AND MANAGEMENT

A CRO, Opera Srl, will perform the Sites initiation visits. Clinical, immunological, virological and laboratory data will be recorded through an electronic Case Report Form (e-CRF).

7.1 Clinical and laboratory assessment

Any clinically relevant event and all laboratory assessments will be recorded and described on the CLINICAL ASSESSMENT page of the e-CRF.

7.2 Concomitant treatment

All concomitant treatments will be recorded on the e-CRF, including the name of the procedure or drug (trade name or generic name), indication, route, total daily dose and start/stop dates.

7.3 Antiretroviral treatment

Patients will receive standard antiretroviral therapy according to clinical conditions and current guidelines.
All antiretroviral treatments and every change of therapy will be recorded on the e-CRF, including the name of the procedure or drug (trade name or generic name), indication, route, total daily dose and start/stop dates.

8.0 ETHICS

This study will be conducted in accordance with the ethical principles that have their origin in the Declaration of Helsinki (as amended by the 52nd World Medical Association (WMA) General Assembly in October 2000 with Note of Clarification on paragraph 29 added by the WMA General Assembly, Washington 2002 and Note of Clarification on paragraph 30 added by the WMA General Assembly, Tokyo 2004) and will be consistent with Good Clinical Practice (GCP) and applicable regulatory requirements.

The study will be conducted in compliance with the protocol. The protocol and any Amendment and the subject informed consent form must receive approval by the Central Ethics Committee (CEC) and by the Local Ethics Committees (LEC) prior to study initiation. Freely given written informed consent must be obtained from every subject prior to enrollment into the study.

Personnel involved in conducting this study will be qualified by education, training, and experience to perform their respective tasks.

For the protection of the enrolled subjects, this study will be conducted respecting the privacy and confidentiality rules, in accordance with the applicable regulatory requirements.

9. ADMINISTRATIVE SECTION

9.1 Informed consent

Preparation of the consent form is the responsibility of the Sponsor. The informed consent includes all the elements required by ICH, GCP and applicable regulatory requirements, and adheres to GCP and to the ethical principles that have their origin in the Declarations of Helsinki. The consent form also includes a statement that ISS and regulatory authorities have direct access to subject records.

The Investigator must provide the subject with a copy of the consent form and written information sheet about the study in a format that is non-technical and easily understood. The Investigator should allow time necessary for the subject to inquire about the details of the study, then informed consent must be signed and personally dated by the subject and by the person who conducted the informed consent discussion. The subject should receive a copy of the signed informed consent and any other written information provided to study subjects prior to subject's participation in the observational study.

The informed consent and any other information provided to subjects, will be revised whenever important new information becomes available that is relevant to the subject's consent, and will receive LEC approval/favorable opinion prior to use. The Investigator, or a person designated by the Investigator, should fully inform the subject of all pertinent aspects of the study and of any new information relevant to the subject's willingness to continue participation in the study. This communication should be documented.

During a subject's participation in the study, any update to the consent form and any update to the written information will be provided to the subject.

9.2 Records and reports

An Investigator is required to prepare and maintain adequate and accurate case histories designed to record all observations and other data pertinent to the investigation on each individual included in the present study.
The confidentiality of records that could identify subjects must be protected, respecting the privacy and confidentiality rules in accordance with the applicable regulatory requirements.

The final e-CRF data entry must be reviewed, by a qualified physician who is an Investigator or Subinvestigator (identified by the Investigators and defined as additional employees involved in the project).

9.3 Central and Local Ethics Committee (CEC/LEC)

Before study initiation, the Investigator must receive written and dated approval/favorable opinion from the CEC/LEC for the protocol, consent form, and any other written information to be provided to subjects.

9.4 Record retention

The Investigator must retain all source documents for the maximum period required by applicable regulations and guidelines. The Investigator must contact ISS prior to destroy any records associated with the study. ISS will notify the Investigator when the study records are no longer needed.
If the Investigator withdraws from the study (e.g., relocation, retirement), the records shall be transferred to a mutually agreed upon designee (e.g., another Investigator, IRB). Notice of such transfer will be given in writing to ISS.

9.5 Confidentiality

The Sponsor compels to maintain the confidentiality upon all scientific information gathered during the entire duration of the study.

9.6 Insurance and refunds

All medical costs for routine clinical monitoring (including new tests ordered as a result of abnormal results) will be covered by the Public Health System.

Being an observational study, no specific insurance coverage is required.

LIST OF ABBREVIATIONS AND SYMBOLS

Ab	Antibody
ADCC	Antibody-Dependent Cell-mediated Cytotoxicity
AE	Adverse Event
Ag	Antigen
AIDS	Acquired Immune Deficiency Syndrome
ANOVA	Analysis of Variance
AVIP	AIDS Vaccine Integrated Programme
CCR-5	Chemokine receptor
CD	Cluster of Designation
CDC	Center of Disease Control
CEC	Central Ethical Committee
CFSE	CarboxyFluorescein Succinimidyl Ester
CRO	Contract Research Organization
DNA	Deoxyribonucleic Acid
e-CRF	Electronic Case Report Form
FP6	Framework Programme 6
GCP	Good Clinical Practice
GMT	Geometric Mean Titers
HAART	Highly Active Antiretroviral Therapy
Hbe Ag	Hepatitis b e Antigen
Hbs Ag	Hepatitis b superficial Antigen
HBV	Hepatitus B Virus
HCV	Hepatitis C Virus
HHV-8	Human Herpes Virus-8
HIV	Human Immunodeficiency Virus
HIV-PI	HIV Protease Inhibitor
HLA	Human Leucocyte Antigens
HTLV-I	Human T-Lymphotropic Virus I
HTLV-II	Human T-Lymphotropic Virus II
ICAV	Italian Concerted Action on HIV/AIDS Vaccine Development
ICH	International Conference on Harmonization
ICS	IntraCellular Staining
IgA	Immunoglobulin A
IgG	Immunoglobulin G
IgM	Immunoglobulin M
IL-2	Interleukin-2
IL-4	Interleukin-4
LTNP	Long Term Non Progressor
IRB	Institutional Review Board
ISS	Istituto Superiore di Sanità
LEC	Local Ethics Committee
ml	Milliliters
NRTI	Nucleosides Retro-Transcriptase Inhibitor
NNRTI	Non Nucleosides Retro-Transcriptase Inhibitor
OBS	Observational Study
PBMC	Peripheral Blood Mononuclear Cells

RNA	Ribonucleic Acid
SAS	Statistical and data Analysis Software
SOPs	Standard Operating Procedures
γIFN	γ interferon
Th1	Type 1 helper T Cells
Th2	Type 2 helper T Cells
TM	Trade Mark
WMA	World Medical Assembly
μL	Micro Liter

REFERENCES

André P, Groettrup M, Klenerman P, de Giuli R, Booth BL, Cerundolo V, Bonneville M, Jotereau F, Zinkernagel RM, Lotteau V. An inhibitor of HIV-1 protease modulates proteasome activity, antigen presentation, and T cell responses. Proc Natl Acad Sci USA 1998, 95:13120-4.

Arya SK, Guo C, Josephs SF, Wong-Staal F. Trans-activator gene of human T-lymphotropic virus type III (HTLV-III). Science 1985, 229:69-73.

Chang, H. C., F. Samaniego, B. C. Nair, L. Buonaguro, and B. Ensoli. HIV-1 Tat protein exits from cells via a leaderless secretory pathway and binds to extracellular matrix-associated heparan sulfate proteoglycans through its basic region. AIDS 1997, 11:1421-1431.

Chitnis S, Mondal D, Agrawal KC. Zidovudine (AZT) treatment suppresses granulocyte-monocyte colony stimulating factor receptor type alpha (GM-CSFR alpha) gene expression in murine bone marrow cells. Life Sci 2002, 71:967-78.

Demirhan I, **Chandra A**, **Sarin PS**, **Hasselmayer O**, **Hofmann D**, **Chandra P**. Inhibition of tat-mediated HIV-1-LTR transactivation and virus replication by sulfhydryl compounds with chelating properties. Anticancer Res. 2000, 20:2513-7

Ensoli B, Barillari G, Salahuddin SZ, Gallo RC, Wong-Staal F. Tat protein of HIV-1 stimulates growth of cells derived from Kaposi's sarcoma lesions of AIDS patients. Nature 1990, 345:84-6.

Ensoli B, Buonaguro L, Barillari G, Fiorelli V, Gendelman R, Morgan RA, Wingfield P, Gallo RC. Release, uptake, and effects of extracellular human immunodeficiency virus type1 Tat protein on cell growth and viral transactivation. J Virol 1993, 67:277-87.

Ensoli B, Gendelman R, Markham P, Fiorelli V, Colombini S, Raffeld M, Cafaro A, Chang HK, Brady JN, Gallo RC. Synergy between basic fibroblast growth factor and HIV-1 Tat protein in induction of Kaposi's sarcoma. Nature 1994, 371:674-80.

Ensoli B, Fiorelli V, Ensoli F, Cafaro A, Titti F, Buttò S, Monini P, Magnani M, Caputo A and Garaci E. Candidate HIV-1 Tat vaccine development: from basic science to clinical trials. AIDS 2006, 20: 2245-2261.

Fisher AG, Feinberg MB, Josephs SF, Harper ME, Marselle LM, Reyes G, Gonda MA, Aldovini A, Debouk C, Gallo RC, et al. The trans-activator gene of HTLV-III is essential for virus replication. Nature 1986, 320:367-71.

Gruber A, Wheat JC, Kuhen KL, Looney DJ, Wong-Staal, F. Differential effects of HIV-1 protease inhibitors on dendritic cell immunophenotype and function. J Biol Chem 2001, 276:47840-3.

Hashimoto KI, Tsunoda R, Okamoto M, Shigeta S, Baba M. Stavudine selectively induces apoptosis in HIV type 1-infected cells. AIDS Res Hum Retroviruses 1997, 13:193-9.

Kobayashi Y, Matsumoto M, Kotani M, Makino T. Possible involvement of matrix metalloproteinase-9 in Langerhans cell migration and maturation. J Immunol 1999, 163:5989-93.

Krone WJ, **Debouck C**, **Epstein LG**, **Heutink P**, **Meloen R**, **Goudsmit J**. Natural antibodies to HIV-tat epitopes and expression of HIV-1 genes in vivo. J. Med. Virol. 1988, 26:261-70

Lu W, Andrieu JM. HIV protease inhibitors restore impaired T-cell proliferative response in vivo and in vitro: a viral-suppression-independent mechanism. Blood 2000, 96:250-8.

Mangiacasale R, Pittoggi C, Sciamanna I, Careddu A, Mattei E, Lorenzini R, Travaglini L, Landriscina M, Barone C, Nervi C, Lavia P, Spadafora C. Exposure of normal and transformed cells to nevirapine, a reverse transcriptase inhibitor, reduces cell growth and promotes differentiation. Oncogene 2003, 22:2750-61.

Phenix BN, Lum JJ, Nie Z, Sanchez-Dardon J, Badley AD. Antiapoptotic mechanism of HIV protease inhibitors: preventing mitochondrial transmembrane potential loss. Blood 2001, 98:1078-85.

Pilon AA, Lum JJ, Sanchez-Dardon J, Phenix BN, Douglas R, Badley AD. Induction of apoptosis by a nonnucleoside human immunodeficiency virus type 1 reverse transcriptase inhibitor. Antimicrob Agents Chemother 2002, 46:2687-91.

Re MC, Furlini G, Vignoli M, Ramazzotti E, Roderigo G, De Rosa V, Zauli G, Lolli S, Capitani S, La Placa M. J. Acquired Immune Defic. Syndr. Hum. Retrovirol 1995, 10:408-416

Re MC, Vignoli M, Furlini G, Gibellini D, Colangeli V, Vitone F, La Placa M. Antibodies against full-lenght Tat protein and some low-molecular-weight Tat-peptides correlate with low or undetectable viral load in HIV-1 seropositive patients. J Clin Virol 2001, 21:81-89.

Reiss P, Lange JM, de Ronde A, de Wolf F, Dekker J, Debouck C, Goudsmit J. Speed of progression to AIDS and degree of antibody response to accessory gene products of HIV-1. J Med Virol 1990, 30:163-8.

Rezza G, **Fiorelli V**, **Dorrucci M**, **Ciccozzi M**, **Tripiciano A**, **Scoglio A**, **Collacchi B**, **Ruiz-Alvarez M**, **Giannetto C**, **Caputo A**, **Tomasoni L**, **Castelli F**, **Sciandra M**, **Sinicco A**, **Ensoli F**, **Butto S**, **Ensoli B**. The presence of anti-Tat antibodies is predictive of long-term nonprogression to AIDS or severe immunodeficiency: findings in a cohort of HIV-1 seroconverters. J Infect Dis. 2005, 15:1321-4.

Rodman TC, To SE, Hashish H, Manchester K. Epitopes for natural antibodies of human immunodeficiency virus (HIV)-negative(normal) and HIV-positive sera are coincident with two key functional sequences of HIV Tat protein. Proc Natl Acad Sci USA 1993, 90:7719-23.

Schmidtke G, Holzhütter HG, Bogyo M, Kairies N, Groll M, de Giuli R, Emch S, Groettrup M. How an inhibitor of the HIV-I protease modulates proteasome activity. J Biol Chem 1999, 274:35734-40.

Sloand EM, Kumar PN, Kim S, Chaudhuri A, Weichold FF, Young NS. Human immunodeficiency virus type 1 protease inhibitor modulates activation of peripheral blood

CD4(+) T cells and decreases their susceptibility to apoptosis in vitro and in vivo. Blood 1999, 94:1021-7.

Sloand EM, Maciejewski J, Kumar P, Kim S, Chaudhuri A, Young N. Protease inhibitors stimulate hematopoiesis and decrease apoptosis and ICE expression in CD34(+) cells. Blood 2000, 96:2735-9.

Sommadossi JP, Carlisle R. Toxicity of 3'-azido-3'-deoxythymidine and 9-(1,3-dihydroxy-2-propoxymethyl)guanine for normal human hematopoietic progenitor cells in vitro. Antimicrob Agents Chemother 1987, 31:452-4.

Viora M, Di Genova G., Rivabene R, Malorni W, Fattorossi A. Interference with cell cycle progression and induction of apoptosis by dideoxynucleoside analogs. Int J Immunopharmacol 1997, 19:311-21.

Viora M, Camponeschi B. Down-regulation of interleukin-2 receptor gene activation and protein expression by dideoxynucleoside analogs. Cell Immunol 1995, 163:289-95.

Weichold FF, Bryant JL, Pati S, Barabitskaya O, Gallo RC, Reitz MS. HIV-1 protease inhibitor ritonavir modulates susceptibility to apoptosis of uninfected T cells. J Hum Virol 1999, 2:261-9.

Wu Y, Marsh JW. Selective transcription and modulation of resting T cell activity by preintegrated HIV DNA. Science 2001, 293:1503-6.

Zagury JF, Sill A, Blattner W, Lachgar A, Le Buanec H, Richardson M, Rappaport J, Hendel H, Bizzini B, Gringeri A, Carcagno M, Criscuolo M, Burny A, Gallo RC, Zagury D. Antibodies to the HIV-1 Tat protein correlated with nonprogression to AIDS: a rationale for the use of Tat toxoid as an HIV-1 vaccine. J Hum Virol 1998, 1:282-92.

OBSERVATIONAL STUDY WITH ADDITIONAL DIAGNOSTIC PROCEDURES ON ANTI-TAT IMMUNE RESPONSE IN HIV-1-INFECTED HAART-TREATED ADULT SUBJECTS

(Protocol Number : ISS OBS T-002)

Issue Date : June 14, 2007

Sponsor Representatives

Name	Signature	Date

Barbara Ensoli, MD, PhD
National AIDS Center

Istituto Superiore di Sanità
Viale Regina Elena, 299
00161 - Rome, Italy

Clinical/Laboratory site - Principal Investigator
I declare to have read and understood the above mentioned protocol and I agree to conduct this trial in accordance with all stipulations of the protocol and in accordance with the declaration of Helsinki.

Name	Signature	Date

(typed or printed)
Institute address:
(typed or printed)
